# Correction: Salivary biomarkers of tactical athlete readiness: A systematic review

**DOI:** 10.1371/journal.pone.0353341

**Published:** 2026-07-07

**Authors:** Bryndan Lindsey, Yosef Shaul, Joel Martin

In the Review and registration subsection of the Materials and methods, there is an error in the second sentence. The correct sentence is: The systematic review was registered in the International Prospective Register of Systematic Reviews (PROSPERO; registration #: CRD42022370388).
