# Supplementary material for: Salivary biomarkers of tactical athlete readiness: A systematic review
Source: PLoS One. 2025 Apr 29;20(4):e0321223. doi: 10.1371/journal.pone.0321223 (PMC12040155; doi:10.1371/journal.pone.0321223)
Supplement: S1 Table — (DOCX) [file pone.0321223.s001.docx]

**S1 Table.** Search strategy including list of search terms and filters.

| **Databases** | | | | | | | |
| --- | --- | --- | --- | --- | --- | --- | --- |
|  | ***PubMed*** |  | ***SportDiscus (Ebsco)*** |  | ***CINAHL PLUS (Ebsco)*** |  | ***Google Scholar*** |
| **Filters** | 30 years, Human subjects, Full text, English |  | 30 years, Peer review, English, Academic journal |  | 30 years, Peer review, English, Academic journal, Human subjects |  | 30 years, English. |
| **Category** |  |  |  |  |  |  |  |
| Saliva | (saliv*[Title/Abstract]) |  | AB(saliv*) |  | AB(saliv*) |  | saliva |
| Operational Readiness | AND ( "athletic performance"[Title/Abstract] OR fitness[Title/Abstract] OR training[Title/Abstract] or conditioning OR preparedness[Title/Abstract] OR "muscle strength"[Title/Abstract] OR aerobic[Title/Abstract] OR mental[Title/Abstract] OR cognitive[Title/Abstract] OR resilience[Title/Abstract] OR exercise[Title/Abstract] OR readiness[Title/Abstract]) |  | AND( AB("athletic performance") OR AB(fitness) OR AB(training) OR AB(conditioning) OR AB(preparedness) OR AB("muscle strength") OR AB(aerobic) OR AB(mental) OR AB(cognitive) OR AB(resilience) OR AB(exercise) OR AB(readiness)) |  | AND( AB("athletic performance") OR AB(fitness) OR AB(training) OR AB(conditioning) OR AB(preparedness) OR AB("muscle strength") OR AB(aerobic) OR AB(mental) OR AB(cognitive) OR AB(resilience) OR AB(exercise) OR AB(readiness)) |  | AND (athletic \| fitness \| strength \| aerobic \| cognitive \| psych* \| exercise) |
| Testing | AND (assess* OR test* OR task OR perform* OR measure*) |  | AND (assess* OR test* OR task OR perform* OR measure*) |  | AND (assess* OR test* OR task OR perform* OR measure*) |  | AND performance |
| Biomarkers | AND (metabolites OR hormon* OR cognitive OR neur* OR endoc* OR immun* OR mental OR cognitive OR enzym* OR inflamm* OR cortisol OR interleukin OR cytokines or interferon) |  | AND (metabolites OR hormon* OR cognitive OR neur* OR endoc* OR immun* OR mental OR cognitive OR enzym* OR inflamm* or cortisol OR interleukin OR cytokines or interferon) |  | AND (metabolites OR hormon* OR cognitive OR neur* OR endoc* OR immun* OR mental OR cognitive OR enzym* OR inflamm* or cortisol OR interleukin OR cytokines or interferon) |  | AND marker |
| Acute stressors | AND ("sleep" OR "heat" OR dehydration OR "hypoxia" OR "hypothermia" OR anxiety OR acute OR stress OR Injury) |  | AND ("sleep" OR "heat" OR dehydration OR "hypoxia" OR "hypothermia" OR anxiety OR acute OR stress OR Injury) |  | AND ("sleep" OR "heat" OR dehydration OR "hypoxia" OR "hypothermia" OR anxiety OR acute OR stress OR Injury) |  | AND (sleep \| injury \| dehydration \| "acute stress*") |
| Exclude  diseased populations | NOT (Alzheimer[Title/Abstract] OR Parkinson[Title/Abstract] OR dementia[Title/Abstract] OR Insomnia[Title/Abstract] OR dental[Title/Abstract] OR disease[Title/Abstract] OR cancer[Title/Abstract] OR diabetes[Title/Abstract] OR patient*[Title/Abstract] ) |  | NOT (AB(Alzheimer) OR AB(Parkinson) OR AB(dementia) OR AB(Insomnia) OR AB(dental) OR AB(disease) OR AB(cancer) OR AB(diabetes) OR AB(patient*) ) |  | NOT (AB(Alzheimer) OR AB(Parkinson) OR AB(dementia) OR AB(Insomnia) OR AB(dental) OR AB(disease) OR AB(cancer) OR AB(diabetes) OR AB(patient*) ) |  | -Alzheimer -Parkinson -dementia -Insomnia -cancer -diabetes -patient |
| Exclude population by age | NOT (elderly[Title/Abstract] OR frail[Title/Abstract] OR "older adults"[Title/Abstract] OR children[Title/Abstract] OR boys[Title/Abstract] OR girls[Title/Abstract] OR adolesc*[Title/Abstract]) |  | NOT (AB(elderly) OR AB(frail) OR AB("older adults") OR AB(children) OR AB(boys) OR AB(girls) OR AB(adolesc*)) |  | NOT (AB(elderly) OR AB(frail) OR AB("older adults") OR AB(children) OR AB(boys) OR AB(girls) OR AB(adolesc*)) |  | -elderly -child |
