# Supplementary material for: Salivary biomarkers of tactical athlete readiness: A systematic review
Source: PLoS One. 2025 Apr 29;20(4):e0321223. doi: 10.1371/journal.pone.0321223 (PMC12040155; doi:10.1371/journal.pone.0321223)
Supplement: S2 Table — (DOCX) [file pone.0321223.s002.docx]

**S2 Table.** Table of all articles identified during literature search process with inclusion/exclusion decision and justification

| Title | Year | Authors | Inclusion/Exclusion Status |
| --- | --- | --- | --- |
| Elevated hair cortisol concentrations in endurance athletes | 2012 | Skoluda, Nadine; Dettenborn, Lucia; Stalder, Tobias; Kirschbaum, Clemens | Not applicable based on title |
| Cortisol awakening response prospectively predicts peritraumatic and acute stress reactions in police officers | 2011 | Inslicht, Sabra S; Otte, Christian; McCaslin, Shannon E; Apfel, Brigitte A; Henn-Haase, Clare; Metzler, Thomas; Yehuda, Rachel; Neylan, Thomas C; Marmar, Charles R | Not applicable based on title |
| Self-control and its relation to emotions and psychobiology: evidence from a Day Reconstruction Method study | 2014 | Daly, M; Baumeister, R F; Delaney, L; MacLachlan, M | Not applicable based on title |
| Acute stress alters auditory selective attention in humans independent of HPA: a study of evoked potentials | 2011 | Elling, Ludger; Steinberg, Christian; Brˆckelmann, Ann-Kathrin; Dobel, Christan; Bˆlte, Jens; Junghofer, Markus | Not applicable based on title |
| Association of serotonin transporter gene polymorphisms with poststroke depression | 2008 | Kohen, Ruth; Cain, Kevin C; Mitchell, Pamela H; Becker, Kyra; Buzaitis, Ann; Millard, Steven P; Navaja, Grace P; Teri, Linda; Tirschwell, David; Veith, Richard | Not applicable based on title |
| Effects of light on cognitive brain responses depend on circadian phase and sleep homeostasis | 2011 | Vandewalle, Gilles; Archer, Simon N; Wuillaume, Catherine; Balteau, Evelyne; Degueldre, Christian; Luxen, AndrÈ; Dijk, Derk-Jan; Maquet, Pierre | Not applicable based on title |
| Vitamin E and immunity after the Kona Triathlon World Championship | 2004 | Nieman, David C; Henson, Dru A; McAnulty, Steven R; McAnulty, Lisa S; Morrow, Jason D; Ahmed, Alaa; Heward, Chris B | Not applicable based on title |
| L-Theanine reduces psychological and physiological stress responses | 2007 | Kimura, Kenta; Ozeki, Makoto; Juneja, Lekh Raj; Ohira, Hideki | Not applicable based on title |
| Acute caffeine ingestion's increase of voluntarily chosen resistance-training load after limited sleep | 2012 | Cook, Christian; Beaven, C Martyn; Kilduff, Liam P; Drawer, Scott | Not applicable based on title |
| Skill execution and sleep deprivation: effects of acute caffeine or creatine supplementation - a randomized placebo-controlled trial | 2011 | Cook, Christian J; Crewther, Blair T; Kilduff, Liam P; Drawer, Scott; Gaviglio, Chris M | Included |
| Salivary reactivity in restrained and unrestrained eaters and women with bulimia nervosa | 1996 | Bulik, C M; Lawson, R H; Carter, F A | Not applicable based on title |
| Attenuated cortisol response to psychological stress but not to CRH or ergometry in young habitual smokers | 1993 | Kirschbaum, C; Strasburger, C J; Langkr‰r, J | Not applicable based on title |
| Neuroendocrine and psychometric evaluation of a placebo version of the 'Trier Social Stress Test' | 2009 | Het, S; Rohleder, N; Schoofs, D; Kirschbaum, C; Wolf, O T | Not applicable based on title |
| Extinction of conditioned fear is better learned and recalled in the morning than in the evening | 2013 | Pace-Schott, Edward F; Spencer, Rebecca M C; Vijayakumar, Shilpa; Ahmed, Nafis A K; Verga, Patrick W; Orr, Scott P; Pitman, Roger K; Milad, Mohammed R | Not applicable based on title |
| Effect of chronic training on heart rate variability, salivary IgA and salivary alpha-amylase in elite swimmers with a disability | 2015 | Edmonds, Rohan; Burkett, Brendan; Leicht, Anthony; McKean, Mark | Not applicable based on title |
| Entrainment of the human circadian clock to the natural light-dark cycle | 2013 | Wright, Kenneth P; McHill, Andrew W; Birks, Brian R; Griffin, Brandon R; Rusterholz, Thomas; Chinoy, Evan D | Not applicable based on title |
| Bovine colostrum supplementation attenuates the decrease of salivary lysozyme and enhances the recovery of neutrophil function after prolonged exercise | 2010 | Davison, Glen; Diment, Bethany C | Not applicable based on title |
| The effects of exercise in forest and urban environments on sympathetic nervous activity of normal young adults | 2006 | Yamaguchi, M; Deguchi, M; Miyazaki, Y | Not applicable based on title |
| The Montreal Imaging Stress Task: using functional imaging to investigate the effects of perceiving and processing psychosocial stress in the human brain | 2005 | Dedovic, Katarina; Renwick, Robert; Mahani, Najmeh Khalili; Engert, Veronika; Lupien, Sonia J; Pruessner, Jens C | Not applicable based on title |
| Cognitive emotion regulation fails the stress test | 2013 | Raio, Candace M; Orederu, Temidayo A; Palazzolo, Laura; Shurick, Ashley A; Phelps, Elizabeth A | Not applicable based on title |
| Green space and stress: evidence from cortisol measures in deprived urban communities | 2013 | Roe, Jenny J; Thompson, Catharine Ward; Aspinall, Peter A; Brewer, Mark J; Duff, Elizabeth I; Miller, David; Mitchell, Richard; Clow, Angela | Not applicable based on title |
| Gender difference in neural response to psychological stress | 2007 | Wang, Jiongjiong; Korczykowski, Marc; Rao, Hengyi; Fan, Yong; Pluta, John; Gur, Ruben C; McEwen, Bruce S; Detre, John A | Not applicable based on title |
| Anxiety and stress can predict pain perception following a cognitive stress | 2010 | Hoeger Bement, Marie; Weyer, Andy; Keller, Manda; Harkins, April L; Hunter, Sandra K | Not applicable based on title |
| No effect of caloric restriction on salivary cortisol levels in overweight men and women | 2014 | Tam, Charmaine S; Frost, Elizabeth A; Xie, Wenting; Rood, Jennifer; Ravussin, Eric; Redman, Leanne M; Pennington CALERIE Team | Not applicable based on title |
| Psychoneuroendocrine effects of resource-activating stress management training | 2007 | Storch, Maja; Gaab, Jens; K¸ttel, Yvonne; St¸ssi, Ann-Christin; Fend, Helmut | Not applicable based on title |
| Perceived and measured physical activity and mental stress levels in obstetricians | 2013 | Martinez de Tejada, BegoÒa; Jastrow, Nicole; Poncet, Antoine; Le Scouezec, Iona; Irion, Olivier; Kayser, Bengt | Not applicable based on title |
| Effect of 6 Weeks of n-3 fatty-acid supplementation on oxidative stress in Judo athletes | 2010 | Filaire, Edith; Massart, Alain; Portier, Hugues; Rouveix, Matthieu; Rosado, Fatima; Bage, Anne S; Gobert, Mylene; Durand, Denys | Not applicable based on title |
| Increased salivary cortisol reliably induced by a protein-rich midday meal | 2005 | Gibson, E L; Checkley, S; Papadopoulos, A; Poon, L; Daley, S; Wardle, J | Not applicable based on title |
| Sources of biological and methodological variation in salivary cortisol and their impact on measurement among healthy adults: a review | 2005 | Hansen, Ase Marie; Garde, Anne Helene; Persson, Roger | Not applicable based on title |
| Stress in pregnancy and infant HPA axis function: conceptual and methodological issues relating to the use of salivary cortisol as an outcome measure | 2007 | Egliston, Kerry-Ann; McMahon, Catherine; Austin, Marie-Paule | Not applicable based on title |
| Psychological and cortisol reactivity to experimentally induced stress in adults with ADHD | 2015 | Raz, Sivan; Leykin, Dmitry | Not applicable based on title |
| Stress management: a randomized study of cognitive behavioural therapy and yoga | 2005 | Granath, Jens; Ingvarsson, Sara; von Thiele, Ulrica; Lundberg, Ulf | Not applicable based on title |
| Effects of caffeine, sleep loss, and stress on cognitive performance and mood during US Navy SEAL training Sea-Air-Land | 2002 | Lieberman, Harris R; Tharion, William J; Shukitt-Hale, Barbara; Speckman, Karen L; Tulley, Richard | Not applicable based on title |
| Self perceived work related stress and the relation with salivary IgA and lysozyme among emergency department nurses | 2002 | Yang, Y; Koh, D; Ng, V; Lee, C Y; Chan, G; Dong, F; Goh, S H; Anantharaman, V; Chia, S E | Not applicable based on title |
| The type 5 phosphodiesterase inhibitor tadalafil influences salivary cortisol, testosterone, and dehydroepiandrosterone sulphate responses to maximal exercise in healthy men | 2008 | Di Luigi, Luigi; Baldari, Carlo; SgrÚ, Paolo; Emerenziani, Gian Pietro; Gallotta, Maria Chiara; Bianchini, Serena; Romanelli, Francesco; Pigozzi, Fabio; Lenzi, Andrea; Guidetti, Laura | Not applicable based on title |
| Longitudinal measurement of cortisol in association with mental health and experience of domestic violence and abuse: study protocol | 2013 | Lokhmatkina, Natalia V; Feder, Gene; Blake, Sarah; Morris, Richard; Powers, Victoria; Lightman, Stafford | Not applicable based on title |
| The impact of a week of simulated night work on sleep, circadian phase, and performance | 2003 | Lamond, N; Dorrian, J; Roach, G D; McCulloch, K; Holmes, A L; Burgess, H J; Fletcher, A; Dawson, D | Not applicable based on title |
| Predicting posttraumatic stress symptoms from pretraumatic risk factors: a 2-year prospective follow-up study in firefighters | 2005 | Heinrichs, Markus; Wagner, Dieter; Schoch, Walter; Soravia, Leila M; Hellhammer, Dirk H; Ehlert, Ulrike | Not applicable based on title |
| Salivary alpha-amylase in biobehavioral research: recent developments and applications | 2007 | Granger, Douglas A; Kivlighan, Katie T; el-Sheikh, Mona; Gordis, Elana B; Stroud, Laura R | Not applicable based on title |
| Electroencephalogram, cognitive state, psychological disorders, clinical symptom, and oxidative stress in horticulture farmers exposed to organophosphate pesticides | 2012 | Bayrami, Mansour; Hashemi, Touraj; Malekirad, Ali Akbar; Ashayeri, Hassan; Faraji, Fardin; Abdollahi, Mohammad | Not applicable based on title |
| Effects of caffeine and caffeine withdrawal on mood and cognitive performance degraded by sleep restriction | 2005 | Rogers, Peter J; Heatherley, Susan V; Hayward, Robert C; Seers, Helen E; Hill, Joanne; Kane, Marian | Not applicable based on title |
| Sexual thoughts: links to testosterone and cortisol in men | 2012 | Goldey, Katherine L; van Anders, Sari M | Not applicable based on title |
| Sexy thoughts: effects of sexual cognitions on testosterone, cortisol, and arousal in women | 2011 | Goldey, Katherine L; van Anders, Sari M | Not applicable based on title |
| Effects of stress and sex on acquisition and consolidation of human fear conditioning | 2006 | Zorawski, Michael; Blanding, Nineequa Q; Kuhn, Cynthia M; LaBar, Kevin S | Not applicable based on title |
| Sex differences in negative affective response during nicotine withdrawal | 2006 | Hogle, Joanne M; Curtin, John J | Not applicable based on title |
| Hormone profiles in humans experiencing military survival training | 2000 | Morgan, C A; Wang, S; Mason, J; Southwick, S M; Fox, P; Hazlett, G; Charney, D S; Greenfield, G | No acute stressor |
| Neuropeptide-Y, cortisol, and subjective distress in humans exposed to acute stress: replication and extension of previous report | 2002 | Morgan, Charles A; Rasmusson, Ann M; Wang, Sheila; Hoyt, Gary; Hauger, Richard L; Hazlett, Gary | Not applicable based on title |
| Impairments of spatial working memory and attention following acute psychosocial stress | 2015 | Olver, James S; Pinney, Myra; Maruff, Paul; Norman, Trevor R | Not applicable based on title |
| The time course of psychological stress as revealed by event-related potentials | 2012 | Yang, Juan; Qi, Mingming; Guan, Lili; Hou, Yan; Yang, Yu | Not applicable based on title |
| Acute exposure to stress improves performance in trace eyeblink conditioning and spatial learning tasks in healthy men | 2007 | Duncko, Roman; Cornwell, Brian; Cui, Lihong; Merikangas, Kathleen R; Grillon, Christian | Not applicable based on title |
| Enhancing effects of acute psychosocial stress on priming of non-declarative memory in healthy young adults | 2012 | Hidalgo, Vanesa; Villada, Carolina; Almela, Mercedes; EspÌn, Laura; GÛmez-Amor, Jes˙s; Salvador, Alicia | Not applicable based on title |
| The influence of acute stress on attention mechanisms and its electrophysiological correlates | 2014 | S‰nger, Jessica; Bechtold, Laura; Schoofs, Daniela; Blaszkewicz, Meinolf; Wascher, Edmund | Not applicable based on title |
| The "Trier Social Stress Test": A tool for investigating psychobiological stress responses in a laboratory setting | 2005 | Kirschbaum, C; Pirke, K M; Hellhammer, D H | Not applicable based on title |
| Oxytocin facilitates the sensation of social stress | 2014 | Eckstein, Monika; Scheele, Dirk; Weber, Kristina; Stoffel-Wagner, Birgit; Maier, Wolfgang; Hurlemann, RenÈ | Not applicable based on title |
| The role of trait emotional intelligence in emotion regulation and performance under pressure | 2014 | Laborde, Sylvain; Lautenbach, Franziska; Allen, Mark S.; Herbert, Cornelia; Achtzehn, Silvia | Not applicable based on title |
| Acute threat to the social self: shame, social self-esteem, and cortisol activity | 2005 | Gruenewald, Tara L; Kemeny, Margaret E; Aziz, Najib; Fahey, John L | Not applicable based on title |
| Preliminary evidence for reduced cortisol responsivity to psychological stress in women using oral contraceptive medication | 2005 | Kirschbaum, C; Pirke, K M; Hellhammer, D H | Not applicable based on title |
| Menstrual cycle variation in spatial ability: relation to salivary cortisol levels | 2001 | McCormick, C M; Teillon, S M | Not applicable based on title |
| Measurement of testosterone in human sexuality research: methodological considerations | 2014 | van Anders, Sari M; Goldey, Katherine L; Bell, Sarah N | Not applicable based on title |
| Corticotroph axis sensitivity after exercise in endurance-trained athletes | 1998 | Duclos, M; Corcuff, J B; Arsac, L; Moreau-Gaudry, F; Rashedi, M; Roger, P; Tabarin, A; Manier, G | Not applicable based on title |
| Stress differentially modulates fear conditioning in healthy men and women | 2006 | Jackson, Eric D; Payne, Jessica D; Nadel, Lynn; Jacobs, W Jake | Not applicable based on title |
| The relationship between cortisol, stress and psychiatric illness: New insights using hair analysis | 2015 | Herane Vives, A; De Angel, V; Papadopoulos, A; Strawbridge, R; Wise, T; Young, A H; Arnone, D; Cleare, A J | Not applicable based on title |
| Sleep and circadian rhythms in mining operators: limited evidence of adaptation to night shifts | 2012 | Ferguson, Sally A; Kennaway, David J; Baker, Angela; Lamond, Nicole; Dawson, Drew | Not applicable based on title |
| The effects of stress on cognitive aging, physiology and emotion (ESCAPE) project | 2015 | Scott, Stacey B; Graham-Engeland, Jennifer E; Engeland, Christopher G; Smyth, Joshua M; Almeida, David M; Katz, Mindy J; Lipton, Richard B; Mogle, Jacqueline A; Munoz, Elizabeth; Ram, Nilam; Sliwinski, Martin J | Not applicable based on title |
| Cortisol level modulated by integrative meditation in a dose-dependent fashion | 2014 | Fan, Yaxin; Tang, Yi-Yuan; Posner, Michael I | Not applicable based on title |
| Stress improves task processing efficiency in dual-tasks | 2013 | Beste, Christian; Yildiz, Ali; Meissner, Tobias W; Wolf, Oliver T | Not applicable based on title |
| Dispositional emotion coping styles and physiological responses to expressive writing | 2013 | Tamagawa, Rie; Moss-Morris, Rona; Martin, Alexandra; Robinson, Elizabeth; Booth, Roger J | Not applicable based on title |
| Stress impairs acquisition of delay eyeblink conditioning in men and women | 2009 | Wolf, Oliver T; Minnebusch, Denise; Daum, Irene | Not applicable based on title |
| Maternal mood and concordant maternal and infant salivary cortisol during heel lance while in kangaroo care | 2015 | Castral, T C; Warnock, F; Dos Santos, C B; DarÈ, M F; Moreira, A C; Antonini, S R R; Scochi, C G S | Not applicable based on title |
| Salivary antimicrobial protein response to prolonged running | 2013 | Gillum, T L; Kuennen, M; Gourley, C; Schneider, S; Dokladny, K; Moseley, P | Not applicable based on title |
| A framework for understanding the training process leading to elite performance | 2005 | Smith, David J | Not applicable based on title |
| Severe decrements in cognition function and mood induced by sleep loss, heat, dehydration, and undernutrition during simulated combat | 2005 | Lieberman, Harris R; Bathalon, Gaston P; Falco, Christina M; Kramer, F Matthew; Morgan, Charles A; Niro, Philip | Included |
| Examining the effectiveness of psychological strategies on physiologic markers: evidence-based suggestions for holistic care of the athlete | 2014 | Dawson, Michelle A; Hamson-Utley, Jennifer Jordan; Hansen, Rodney; Olpin, Michael | Not applicable based on title |
| Are salivary gonadal steroid concentrations influenced by acute psychosocial stress? A study using the Trier Social Stress Test (TSST) | 2011 | Schoofs, Daniela; Wolf, Oliver T | Not applicable based on title |
| Yoga as a complementary treatment for smoking cessation in women | 2012 | Bock, Beth C; Fava, Joseph L; Gaskins, Ronnesia; Morrow, Kathleen M; Williams, David M; Jennings, Ernestine; Becker, Bruce M; Tremont, Geoffrey; Marcus, Bess H | Not applicable based on title |
| Placebo-mediated, Naloxone-sensitive suggestibility of short-term memory performance | 2011 | Stern, Jair; Candia, Victor; Porchet, Roseline I; Krummenacher, Peter; Folkers, Gerd; Schedlowski, Manfred; Ettlin, Dominik A; Schˆnb‰chler, Georg | Not applicable based on title |
| A single dose of melatonin prevents the phase delay associated with a delayed weekend sleep pattern | 2001 | Yang, C M; Spielman, A J; D'Ambrosio, P; Serizawa, S; Nunes, J; Birnbaum, J | Not applicable based on title |
| Intimate male partner violence impairs immune control over herpes simplex virus type 1 in physically and psychologically abused women | 2005 | Garcia-Linares, M Isabel; Sanchez-Lorente, Segunda; Coe, Christopher L; Martinez, Manuela | Not applicable based on title |
| Biochemical and immunological markers of over-training | 2002 | Gleeson, Michael | Not applicable based on title |
| Monitoring training loads, stress, immune-endocrine responses and performance in tennis players | 2013 | Gomes, R V; Moreira, A; Lodo, L; Nosaka, K; Coutts, A J; Aoki, M S | Not applicable based on title |
| Noninvasive biochemical monitoring of physiological stress by Fourier transform infrared saliva spectroscopy | 2010 | Khaustova, Svetlana; Shkurnikov, Maxim; Tonevitsky, Evgeny; Artyushenko, Viacheslav; Tonevitsky, Alexander | Not applicable based on title |
| Monitoring internal training load and mucosal immune responses in futsal athletes | 2013 | Moreira, Alexandre; de Moura, Nivaldo Ribeiro; Coutts, Aaron; Costa, Eduardo Caldas; Kempton, Thomas; Aoki, Marcelo Saldanha | Not applicable based on title |
| Salivary hormone and immune responses to three resistance exercise schemes in elite female athletes | 2011 | Nunes, Jo„o A; Crewther, Blair T; Ugrinowitsch, Carlos; Tricoli, Valmor; Viveiros, LuÌs; de Rose, Dante; Aoki, Marcelo S | Not applicable based on title |
| Monitoring training load, recovery-stress state, immune-endocrine responses, and physical performance in elite female basketball players during a periodized training program | 2014 | Nunes, Jo„o A; Moreira, Alexandre; Crewther, Blair T; Nosaka, Ken; Viveiros, Luis; Aoki, Marcelo S | Not applicable based on title |
| Saliva as a tool for monitoring steroid, peptide and immune markers in sport and exercise science | 2011 | Papacosta, Elena; Nassis, George P | Not applicable based on title |
| Modification of the association between serotonin transporter genotype and risk of posttraumatic stress disorder in adults by county-level social environment | 2009 | Koenen, Karestan C; Aiello, Allison E; Bakshis, Erin; Amstadter, Ananda B; Ruggiero, Kenneth J; Acierno, Ron; Kilpatrick, Dean G; Gelernter, Joel; Galea, Sandro | Not applicable based on title |
| Paradoxical effects of stress and an executive task on decisions under risk | 2013 | Pabst, Stephan; Schoofs, Daniela; Pawlikowski, Mirko; Brand, Matthias; Wolf, Oliver T | Not applicable based on title |
| Salivary stress markers and psychological stress in simulated microgravity: 21 days in 6∞ head-down tilt | 2011 | Rai, Balwant; Kaur, Jasdeep | Not applicable based on title |
| An acute, double-blind, placebo-controlled cross-over study of 320 mg and 640 mg doses of Bacopa monnieri (CDRI 08) on multitasking stress reactivity and mood | 2014 | Benson, Sarah; Downey, Luke A; Stough, Con; Wetherell, Mark; Zangara, Andrea; Scholey, Andrew | Not applicable based on title |
| Effect of Tongkat Ali on stress hormones and psychological mood state in moderately stressed subjects | 2013 | Talbott, Shawn M; Talbott, Julie A; George, Annie; Pugh, Mike | Not applicable based on title |
| Reduction of rise in blood pressure and cortisol release during stress by Ginkgo biloba extract (EGb 761) in healthy volunteers | 2002 | Jezova, D; Duncko, R; Lassanova, M; Kriska, M; Moncek, F | Not applicable based on title |
| The effects of acute physical exercise on memory, peripheral BDNF, and cortisol in young adults | 2016 | Hˆtting, Kirsten; Schickert, Nadine; Kaiser, Jochen; Rˆder, Brigitte; Schmidt-Kassow, Maren | Not applicable based on title |
| Sensitivity to intranasal oxytocin in adult men with early parental separation | 2007 | Meinlschmidt, Gunther; Heim, Christine | Not applicable based on title |
| Altered States of Consciousness during an Extreme Ritual | 2016 | Lee, Ellen M; Klement, Kathryn R; Ambler, James K; Loewald, Tonio; Comber, Evelyn M; Hanson, Sarah A; Pruitt, Bria; Sagarin, Brad J | Not applicable based on title |
| Acute stress affects risk taking but not ambiguity aversion | 2014 | Buckert, Magdalena; Schwieren, Christiane; Kudielka, Brigitte M; Fiebach, Christian J | Not applicable based on title |
| Effects of Group Drumming Interventions on Anxiety, Depression, Social Resilience and Inflammatory Immune Response among Mental Health Service Users | 2016 | Fancourt, Daisy; Perkins, Rosie; Ascenso, Sara; Carvalho, Livia A; Steptoe, Andrew; Williamon, Aaron | Not applicable based on title |
| Local modulation of human brain responses by circadian rhythmicity and sleep debt | 2016 | Muto, Vincenzo; Jaspar, Mathieu; Meyer, Christelle; KussÈ, Caroline; Chellappa, Sarah L; Degueldre, Christian; Balteau, Evelyne; Shaffii-Le Bourdiec, Anahita; Luxen, AndrÈ; Middleton, Benita; Archer, Simon N; Phillips, Christophe; Collette, Fabienne; Vandewalle, Gilles; Dijk, Derk-Jan; Maquet, Pierre | Not applicable based on title |
| Salivary dehydroepiandrosterone secretion in response to acute psychosocial stress and its correlations with biological and psychological changes | 2008 | Izawa, Shuhei; Sugaya, Nagisa; Shirotsuki, Kentaro; Yamada, Kosuke Chris; Ogawa, Namiko; Ouchi, Yuko; Nagano, Yuichiro; Suzuki, Katsuhiko; Nomura, Shinobu | Not applicable based on title |
| Coping with Work-Related Stress through Guided Imagery and Music (GIM): Randomized Controlled Trial | 2015 | Beck, Bolette Daniels; Hansen, ≈se Marie; Gold, Christian | Not applicable based on title |
| A study of the effect of relaxing music on heart rate recovery after exercise among healthy students | 2014 | Tan, Fuitze; Tengah, Asrin; Nee, Lo Yah; Fredericks, Salim | Not applicable based on title |
| The effect of music on the human stress response | 2013 | Thoma, Myriam V; La Marca, Roberto; Brˆnnimann, Rebecca; Finkel, Linda; Ehlert, Ulrike; Nater, Urs M | Not applicable based on title |
| Psychosocial and physiological correlates of self-reported hearing problems in male and female musicians in symphony orchestras | 2009 | Hasson, Dan; Theorell, Tˆres; Liljeholm-Johansson, Yvonne; Canlon, Barbara | Not applicable based on title |
| The clinical effectiveness of healing touch | 2002 | Wilkinson, Dawn S; Knox, Pamela L; Chatman, James E; Johnson, Terrance L; Barbour, Nilufer; Myles, Yvonne; Reel, Antonio | Not applicable based on title |
| Relaxing music prevents stress-induced increases in subjective anxiety, systolic blood pressure, and heart rate in healthy males and females | 2005 | Knight, W E; Rickard PhD, N S | Not applicable based on title |
| Modulation of secretory immunoglobulin A in saliva; response to manipulation of mood | 2000 | Hucklebridge, F; Lambert, S; Clow, A; Warburton, D M; Evans, P D; Sherwood, N | Not applicable based on title |
| Risk Impact of having a first-degree relative with affective disorder: a 7-year follow-up study | 2016 | Vinberg, Maj | Not applicable based on title |
| The validity of scopolamine as a pharmacological model for cognitive impairment: a review of animal behavioral studies | 2010 | Klinkenberg, Inge; Blokland, Arjan | Not applicable based on title |
| Is a dark virtual environment scary? | 2009 | Toet, Alexander; van Welie, Marloes; Houtkamp, Joske | Not applicable based on title |
| The Netherlands Study of Depression and Anxiety (NESDA): rationale, objectives and methods | 2005 | Penninx, Brenda W J H; Beekman, Aartjan T F; Smit, Johannes H; Zitman, Frans G; Nolen, Willem A; Spinhoven, Philip; Cuijpers, Pim; De Jong, Peter J; Van Marwijk, Harm W J; Assendelft, Willem J J; Van Der Meer, Klaas; Verhaak, Peter; Wensing, Michel; De Graaf, Ron; Hoogendijk, Witte J; Ormel, Johan; Van Dyck, Richard; NESDA Research Consortium | Not applicable based on title |
| Effects of intranasal oxytocin on steroid hormones in men and women | 2015 | Wirth, Michelle M; Gaffey, Allison E; Martinez, Brandy S | Not applicable based on title |
| Relationships between training load indicators and training outcomes in professional soccer | 2017 | Jaspers, Arne; Brink, Michel S; Probst, Steven G M; Frencken, Wouter G P; Helsen, Werner F | Not applicable based on title |
| Training Load and Fatigue Marker Associations with Injury and Illness: A Systematic Review of Longitudinal Studies | 2017 | Jones, Christopher M; Griffiths, Peter C; Mellalieu, Stephen D | Not applicable based on title |
| Chronic stress and sexual function in women | 2013 | Hamilton, Lisa Dawn; Meston, Cindy M | Not applicable based on title |
| Training and overtraining markers in selected sport events | 2000 | Hartmann, U; Mester, J | Not applicable based on title |
| Menstrual cycle modulation of the relationship between cortisol and long-term memory | 2008 | Andreano, Joseph M; Arjomandi, Hamidreza; Cahill, Larry | Not applicable based on title |
| Exposure to acute stress enhances decision-making competence: Evidence for the role of DHEA | 2016 | Shields, Grant S; Lam, Jovian C W; Trainor, Brian C; Yonelinas, Andrew P | Not applicable based on title |
| Psychological traits and the cortisol awakening response: results from the Netherlands Study of Depression and Anxiety | 2011 | van Santen, Aafke; Vreeburg, Sophie A; Van der Does, A J Willem; Spinhoven, Philip; Zitman, Frans G; Penninx, Brenda W J H | Not applicable based on title |
| Basal testosterone moderates responses to anger faces in humans | 2007 | Wirth, Michelle M; Schultheiss, Oliver C | Not applicable based on title |
| Blunted endocrine and cardiovascular reactivity in young healthy women reporting a history of childhood adversity | 2015 | Voellmin, Annette; Winzeler, Katja; Hug, Evelin; Wilhelm, Frank H; Schaefer, ValÈrie; Gaab, Jens; La Marca, Roberto; Pruessner, Jens C; Bader, Klaus | Not applicable based on title |
| Sex differences in cognitive regulation of psychosocial achievement stress: brain and behavior | 2015 | Kogler, Lydia; Gur, Ruben C; Derntl, Birgit | Not applicable based on title |
| Psychological determinants of the cortisol stress response: the role of anticipatory cognitive appraisal | 2005 | Gaab, J; Rohleder, N; Nater, U M; Ehlert, U | Not applicable based on title |
| Mood and autonomic responses to repeated exposure to the Trier Social Stress Test for Groups (TSST-G) | 2014 | Boesch, Maria; Sefidan, Sandra; Ehlert, Ulrike; Annen, Hubert; Wyss, Thomas; Steptoe, Andrew; La Marca, Roberto | Not applicable based on title |
| Identification of a narrow post-ovulatory window of vulnerability to distressing involuntary memories in healthy women | 2013 | Soni, Mira; Curran, Valerie H; Kamboj, Sunjeev K | Not applicable based on title |
| Neuroimaging evidence for a role of neural social stress processing in ethnic minority-associated environmental risk | 2014 | Akdeniz, Ceren; Tost, Heike; Streit, Fabian; Haddad, Leila; W¸st, Stefan; Sch‰fer, Axel; Schneider, Michael; Rietschel, Marcella; Kirsch, Peter; Meyer-Lindenberg, Andreas | Not applicable based on title |
| Effects of acute psychosocial stress on working memory related brain activity in men | 2010 | Weerda, Riklef; Muehlhan, Markus; Wolf, Oliver T; Thiel, Christiane M | Not applicable based on title |
| Time course of neurobehavioral alertness during extended wakefulness in morning- and evening-type healthy sleepers | 2011 | Taillard, Jacques; Philip, Pierre; Claustrat, Bruno; Capelli, Aurore; Coste, Olivier; Chaumet, Guillaume; Sagaspe, Patricia | Not applicable based on title |
| Effects of the implementation of an 84-hour workweek on neurobehavioral test performance and cortisol responsiveness during testing | 2003 | Persson, Roger; Orbaek, Palle; Ursin, Holger; Kecklund, Gˆran; Osterberg, Kai; Akerstedt, Torbjˆrn | Not applicable based on title |
| Creatine supplementation, sleep deprivation, cortisol, melatonin and behavior | 2007 | McMorris, T; Harris, R C; Howard, A N; Langridge, G; Hall, B; Corbett, J; Dicks, M; Hodgson, C | Not applicable based on title |
| Resilience of the immune system in healthy young students to 30-hour sleep deprivation with psychological stress | 2013 | Matzner, Pini; Hazut, Ofir; Naim, Reut; Shaashua, Lee; Sorski, Liat; Levi, Ben; Sadeh, Avi; Wald, Ilan; Bar-Haim, Yair; Ben-Eliyahu, Shamgar | Not applicable based on title |
| Chronotype, sleep loss, and diurnal pattern of salivary cortisol in a simulated daylong driving | 2010 | Oginska, Halszka; Fafrowicz, Magdalena; Golonka, Krystyna; Marek, Tadeusz; Mojsa-Kaja, Justyna; Tucholska, Kinga | Not applicable based on title |
| The influence of sleep deprivation and oscillating motion on sleepiness, motion sickness, and cognitive and motor performance | 2017 | Kaplan, Janna; Ventura, Joel; Bakshi, Avijit; Pierobon, Alberto; Lackner, James R; DiZio, Paul | Not applicable based on title |
| Is there more to yoga than exercise? | 2011 | Smith, J Andy; Greer, Tammy; Sheets, Timothy; Watson, Sheree | Not applicable based on title |
| Attenuated adrenocorticotropic responses to psychological stress are associated with early smoking relapse | 2005 | al'Absi, Mustafa; Hatsukami, Dorothy; Davis, Gary L | Not applicable based on title |
| Improving strength and power in trained athletes with 3 weeks of occlusion training | 2014 | Cook, Christian J; Kilduff, Liam P; Beaven, C Martyn | Not applicable based on title |
| Saliva metabolomics by NMR for the evaluation of sport performance | 2014 | Santone, C; Dinallo, V; Paci, M; D'Ottavio, S; Barbato, G; Bernardini, S | Not applicable based on title |
| Sleep recalibrates homeostatic and associative synaptic plasticity in the human cortex | 2016 | Kuhn, Marion; Wolf, Elias; Maier, Jonathan G; Mainberger, Florian; Feige, Bernd; Schmid, Hanna; B¸rklin, Jan; Maywald, Sarah; Mall, Volker; Jung, Nikolai H; Reis, Janine; Spiegelhalder, Kai; Klˆppel, Stefan; Sterr, Annette; Eckert, Anne; Riemann, Dieter; Normann, Claus; Nissen, Christoph | Not applicable based on title |
| The effects of L-theanine, caffeine and their combination on cognition and mood | 2008 | Haskell, Crystal F; Kennedy, David O; Milne, Anthea L; Wesnes, Keith A; Scholey, Andrew B | Not applicable based on title |
| Mindfulness meditation improves emotion regulation and reduces drug abuse | 2016 | Tang, Yi-Yuan; Tang, Rongxiang; Posner, Michael I | Not applicable based on title |
| Predictors and moderators of biopsychological social stress responses following brief self-compassion meditation training | 2016 | Arch, Joanna J; Landy, Lauren N; Brown, Kirk Warren | Not applicable based on title |
| Stress and decision making: a few minutes make all the difference | 2013 | Pabst, Stephan; Brand, Matthias; Wolf, Oliver T | Not applicable based on title |
| Cortisol, Chromogranin A, and Pupillary Responses Evoked by Speech Recognition Tasks in Normally Hearing and Hard-of-Hearing Listeners: A Pilot Study | 2016 | Kramer, Sophia E; Teunissen, Charlotte E; Zekveld, Adriana A | Not applicable based on title |
| The integrative effects of cognitive reappraisal on negative affect: associated changes in secretory immunoglobulin A, unpleasantness and ERP activity | 2012 | Zhang, Wencai; Li, Fan; Qin, Shaozheng; Luo, Jing | Not applicable based on title |
| Cortisol and self-report measures of anxiety as predictors of neuropsychological performance | 2012 | Leininger, Shelley; Skeel, Reid | Not applicable based on title |
| Tattooing to "Toughen up": Tattoo experience and secretory immunoglobulin A | 2016 | Lynn, Christopher D; Dominguez, Johnna T; DeCaro, Jason A | Not applicable based on title |
| Stress disrupts the reconsolidation of fear memories in men | 2017 | Meir Drexler, Shira; Wolf, Oliver T | Not applicable based on title |
| One night of partial sleep deprivation impairs recovery from a single exercise training session | 2017 | Rae, Dale E; Chin, Tayla; Dikgomo, Kagiso; Hill, Lee; McKune, Andrew J; Kohn, Tertius A; Roden, Laura C | Not applicable based on title |
| Lower cortisol response in high-resilient caregivers of people with autism: the role of anger | 2017 | Ruiz-Robledillo, N; Romero-MartÌnez, A; Moya-Albiol, L | Not applicable based on title |
| The GoMo study: a randomized clinical trial assessing neonatal pain with Gomco vs Mogen clamp circumcision | 2015 | Sinkey, Rachel G; Eschenbacher, Michaela A; Walsh, Peggy M; Doerger, Rita G; Lambers, Donna S; Sibai, Baha M; Habli, Mounira A | Not applicable based on title |
| Mindfulness-based stress reduction and physiological activity during acute stress: a randomized controlled trial | 2013 | NyklÌ ek, Ivan; Mommersteeg, Paula M C; Van Beugen, Sylvia; Ramakers, Christian; Van Boxtel, Geert J | Not applicable based on title |
| Stress biomarkers as predictors of transition to psychosis in at-risk mental states: roles for cortisol, prolactin and albumin | 2015 | Labad, Javier; Stojanovic-PÈrez, Alexander; Montalvo, Itziar; SolÈ, Montse; Cabezas, ¡ngel; Ortega, Laura; Moreno, Irene; Vilella, Elisabet; Martorell, Lourdes; Reynolds, Rebecca M; GutiÈrrez-Zotes, Alfonso | Not applicable based on title |
| The hippocampal response to psychosocial stress varies with salivary uric acid level | 2016 | Goodman, Adam M; Wheelock, Muriah D; Harnett, Nathaniel G; Mrug, Sylvie; Granger, Douglas A; Knight, David C | Not applicable based on title |
| Salivary nerve growth factor response to stress related to resilience | 2014 | Laurent, Heidemarie K; Laurent, Sean M; Granger, Douglas A | Not applicable based on title |
| How does yoga reduce stress? A systematic review of mechanisms of change and guide to future inquiry | 2015 | Riley, Kristen E; Park, Crystal L | Not applicable based on title |
| The use of testosterone/cortisol ratio in response to acute stress as an indicator of propensity to anger in informal caregivers | 2016 | Romero-MartÌnez, ¡ngel; Moya-Albiol, Luis | Not applicable based on title |
| Perceived stress and cortisol levels predict speed of wound healing in healthy male adults | 2004 | Ebrecht, Marcel; Hextall, Justine; Kirtley, Lauren-Grace; Taylor, Alice; Dyson, Mary; Weinman, John | Not applicable based on title |
| The Research Doesn't Always Apply: Practical Solutions to Evidence-Based Training-Load Monitoring in Elite Team Sports | 2017 | Burgess, Darren J | Not applicable based on title |
| Monitoring Fatigue Status in Elite Team-Sport Athletes: Implications for Practice | 2017 | Thorpe, Robin T; Atkinson, Greg; Drust, Barry; Gregson, Warren | Not applicable based on title |
| Respiratory inflammation and infections in high-performance athletes | 2016 | Gleeson, Maree; Pyne, David B | Not applicable based on title |
| Monitoring training status with HR measures: do all roads lead to Rome? | 2014 | Buchheit, Martin | Not applicable based on title |
| Changes in salivary testosterone concentrations and subsequent voluntary squat performance following the presentation of short video clips | 2012 | Cook, Christian J; Crewther, Blair T | Not applicable based on title |
| Attention-deficit hyperactivity disorder and the stress response | 1998 | King, J A; Barkley, R A; Barrett, S | Not applicable based on title |
| Tear fluid siga as a noninvasive biomarker of mucosal immunity and common cold risk | 2016 | Hanstock, Helen G; Walsh, Neil P; Edwards, Jason P; Fortes, Matthew B; Cosby, Sara L; Nugent, Aaron; Curran, Tanya; Coyle, Peter V; Ward, Mark D; Yong, Xin Hui Aw | No salivary markers |
| Overnight urinary cortisol and cortisone add new insights into adaptation to training | 2005 | GouarnÈ, Caroline; Groussard, Carole; Gratas-Delamarche, Arlette; Delamarche, Paul; Duclos, Martine | Not applicable based on title |
| Female social and sexual interest across the menstrual cycle: the roles of pain, sleep and hormones | 2010 | Guillermo, Chrisalbeth J; Manlove, Heidi A; Gray, Peter B; Zava, David T; Marrs, Chandler R | Not applicable based on title |
| Chronic stress induces a hyporeactivity of the autonomic nervous system in response to acute mental stressor and impairs cognitive performance in business executives | 2015 | Teixeira, Renata Roland; DÌaz, Miguel Mauricio; Santos, Tatiane Vanessa da Silva; Bernardes, Jean Tofoles Martins; Peixoto, Leonardo Gomes; Bocanegra, Olga Lucia; Neto, Morun Bernardino; Espindola, Foued Salmen | Not applicable based on title |
| Low endogenous fibroblast growth factor 2†levels are associated with heightened conditioned fear expression in rats and humans | 2017 | Graham, Bronwyn M; Zagic, Dino; Richardson, Rick | Not applicable based on title |
| Pharmacological interventions for those who have sexually offended or are at risk of offending | 2015 | Khan, Omer; Ferriter, Michael; Huband, Nick; Powney, Melanie J; Dennis, Jane A; Duggan, Conor | Not applicable based on title |
| Reduced stress and inflammatory responsiveness in experienced meditators compared to a matched healthy control group | 2016 | Rosenkranz, Melissa A; Lutz, Antoine; Perlman, David M; Bachhuber, David R W; Schuyler, Brianna S; MacCoon, Donal G; Davidson, Richard J | Not applicable based on title |
| High self-perceived stress and many stressors, but normal diurnal cortisol rhythm, in adults with ADHD (attention-deficit/hyperactivity disorder) | 2009 | Hirvikoski, Tatja; Lindholm, Torun; Nordenstrˆm, Anna; Nordstrˆm, Anna-Lena; Lajic, Svetlana | Not applicable based on title |
| Acute stress impairs the retrieval of extinction memory in humans | 2014 | Raio, Candace M; Brignoni-Perez, Edith; Goldman, Rachel; Phelps, Elizabeth A | Not applicable based on title |
| The scanner as a stressor: evidence from subjective and neuroendocrine stress parameters in the time course of a functional magnetic resonance imaging session | 2011 | Muehlhan, Markus; Lueken, Ulrike; Wittchen, Hans-Ulrich; Kirschbaum, Clemens | Not applicable based on title |
| Markers of biological stress in response to a single session of high-intensity interval training and high-volume training in young athletes | 2016 | Kilian, Yvonne; Engel, Florian; Wahl, Patrick; Achtzehn, Silvia; Sperlich, Billy; Mester, Joachim | Not applicable based on title |
| Psychoendocrine validation of a short measure for assessment of perceived stress management skills in different non-clinical populations | 2013 | Wirtz, Petra H; Thomas, Livia; Domes, Gregor; Penedo, Frank J; Ehlert, Ulrike; Nussbeck, Fridtjof W | Not applicable based on title |
| Sex and stress: Men and women show different cortisol responses to psychological stress induced by the Trier social stress test and the Iowa singing social stress test | 2017 | Reschke-Hern·ndez, Alaine E; Okerstrom, Katrina L; Bowles Edwards, Angela; Tranel, Daniel | Not applicable based on title |
| Perfusion functional MRI reveals cerebral blood flow pattern under psychological stress | 2005 | Wang, Jiongjiong; Rao, Hengyi; Wetmore, Gabriel S; Furlan, Patricia M; Korczykowski, Marc; Dinges, David F; Detre, John A | Not applicable based on title |
| Major depressive disorder and hypothalamic-pituitary-adrenal axis activity: results from a large cohort study | 2009 | Vreeburg, Sophie A; Hoogendijk, Witte J G; van Pelt, Johannes; Derijk, Roel H; Verhagen, Jolanda C M; van Dyck, Richard; Smit, Johannes H; Zitman, Frans G; Penninx, Brenda W J H | Not applicable based on title |
| Influence of early trauma on features of schizophrenia | 2017 | Ruby, Eugene; Rothman, Karen; Corcoran, Cheryl; Goetz, Raymond R; Malaspina, Dolores | Not applicable based on title |
| Preliminary evidence of salivary cortisol predicting performance in a controlled setting | 2014 | Lautenbach, Franziska; Laborde, Sylvain; Achtzehn, Silvia; Raab, Markus | Not applicable based on title |
| Familial social support predicts a reduced cortisol response to stress in sexual minority young adults | 2014 | Burton, C L; Bonanno, G A; Hatzenbuehler, M L | Not applicable based on title |
| Salivary cortisol, heart rate, electrodermal activity and subjective stress responses to the Mannheim Multicomponent Stress Test (MMST) | 2012 | Reinhardt, Tatyana; Schmahl, Christian; W¸st, Stefan; Bohus, Martin | Not applicable based on title |
| Anticipatory sensitization to repeated stressors: the role of initial cortisol reactivity and meditation/emotion skills training | 2015 | Turan, Bulent; Foltz, Carol; Cavanagh, James F; Wallace, B Alan; Cullen, Margaret; Rosenberg, Erika L; Jennings, Patricia A; Ekman, Paul; Kemeny, Margaret E | Not applicable based on title |
| Stress-related and basic determinants of hair cortisol in humans: A meta-analysis | 2017 | Stalder, Tobias; Steudte-Schmiedgen, Susann; Alexander, Nina; Klucken, Tim; Vater, Annika; Wichmann, Susann; Kirschbaum, Clemens; Miller, Robert | Not applicable based on title |
| Brief mindfulness meditation training alters psychological and neuroendocrine responses to social evaluative stress | 2014 | Creswell, J David; Pacilio, Laura E; Lindsay, Emily K; Brown, Kirk Warren | Not applicable based on title |
| Psychological and hormonal features of smokers at risk to gain weight after smoking cessation--results of a multicenter study | 2011 | Koopmann, Anne; Dinter, Christina; Grosshans, Martin; von der Goltz, Christoph; Hentschel, Rahel; Dahmen, Norbert; Gallinat, J¸rgen; Wagner, Michael; Gr¸nder, Gerd; Th¸rauf, Norbert; Wienker, Thomas; Brinkmeyer, J¸rgen; Mobascher, Arian; Spreckelmeyer, Katja N; Clepce, Marion; de Millas, Walter; Wiedemann, Klaus; Winterer, Georg; Kiefer, Falk | Not applicable based on title |
| Effect of prenatal exposure to maternal cortisol and psychological distress on infant development in Bengaluru, southern India: a prospective cohort study | 2017 | Nath, Anita; Murthy, Gudlavalleti Venkata Satyanarayana; Babu, Giridhara R; Di Renzo, Gian Carlo | Not applicable based on title |
| Effects of artificial dawn and morning blue light on daytime cognitive performance, well-being, cortisol and melatonin levels | 2013 | Gabel, Virginie; Maire, Micheline; Reichert, Carolin F; Chellappa, Sarah L; Schmidt, Christina; Hommes, Vanja; Viola, Antoine U; Cajochen, Christian | Not applicable based on title |
| Nurses' experiences, expectations, and preferences for mind-body practices to reduce stress | 2011 | Kemper, Kathi; Bulla, Sally; Krueger, Deborah; Ott, Mary Jane; McCool, Jane A; Gardiner, Paula | Not applicable based on title |
| Salutary effects of an attention bias modification mobile application on biobehavioral measures of stress and anxiety during pregnancy | 2017 | Dennis-Tiwary, Tracy A; Denefrio, Samantha; Gelber, Shari | Not applicable based on title |
| Alterations in Salivary Proteome following Single Twenty-Minute Session of Yogic Breathing | 2015 | Balasubramanian, Sundaravadivel; Janech, Michael G; Warren, Graham W | Not applicable based on title |
| Representational similarity analysis offers a preview of the noradrenergic modulation of long-term fear memory at the time of encoding | 2015 | Visser, RenÈe M; Kunze, Anna E; Westhoff, Bianca; Scholte, H Steven; Kindt, Merel | Not applicable based on title |
| Dose-related effects of delta-9-THC on emotional responses to acute psychosocial stress | 2017 | Childs, Emma; Lutz, Joseph A; de Wit, Harriet | Not applicable based on title |
| Anxiety mediates the effect of acute stress on working memory performance when cortisol levels are high: a moderated mediation analysis | 2015 | Hood, Anna; Pulvers, Kim; Spady, Thomas J; Kliebenstein, Alexa; Bachand, Jennifer | Not applicable based on title |
| Simultaneous measurement of salivary cortisol and alpha-amylase: Application and recommendations | 2017 | Strahler, Jana; Skoluda, Nadine; Kappert, Mattes B; Nater, Urs M | Not applicable based on title |
| Functional ADA polymorphism increases sleep depth and reduces vigilant attention in humans | 2012 | Bachmann, ValÈrie; Klaus, Federica; Bodenmann, Sereina; Sch‰fer, Nikolaus; Brugger, Peter; Huber, Susanne; Berger, Wolfgang; Landolt, Hans-Peter | Not applicable based on title |
| Cognitive function, stress hormones, heart rate and nutritional status during simulated captivity in military survival training | 2016 | Lieberman, Harris R; Farina, Emily K; Caldwell, John; Williams, Kelly W; Thompson, Lauren A; Niro, Philip J; Grohmann, Kyle A; McClung, James P | Undefined stressor in criteria |
| Learning and Motivational Processes Contributing to Pavlovian-Instrumental Transfer and Their Neural Bases: Dopamine and Beyond | 2016 | Corbit, Laura H; Balleine, Bernard W | Not applicable based on title |
| Sex differences in HPA axis activity in response to a meal | 2012 | Martens, Eveline A P; Lemmens, Sofie G T; Adam, Tanja C M; Westerterp-Plantenga, Margriet S | Not applicable based on title |
| Apolipoprotein e genotype, cortisol, and cognitive function in community-dwelling older adults | 2008 | Lee, Brian K; Glass, Thomas A; Wand, Gary S; McAtee, Matthew J; Bandeen-Roche, Karen; Bolla, Karen I; Schwartz, Brian S | Not applicable based on title |
| Mindfulness training affects attention--or is it attentional effort? | 2012 | Jensen, Christian Gaden; Vangkilde, Signe; Frokjaer, Vibe; Hasselbalch, Steen G | Not applicable based on title |
| Caffeine stimulation of cortisol secretion across the waking hours in relation to caffeine intake levels | 2005 | Lovallo, William R; Whitsett, Thomas L; al'Absi, Mustafa; Sung, Bong Hee; Vincent, Andrea S; Wilson, Michael F | Not applicable based on title |
| Stress-induced changes in human salivary alpha-amylase activity -- associations with adrenergic activity | 2006 | Nater, Urs Markus; La Marca, Roberto; Florin, Ladina; Moses, Anthony; Langhans, Wolfgang; Koller, Markus M; Ehlert, Ulrike | Not applicable based on title |
| Assessing daily stress processes in social surveys by combining stressor exposure and salivary cortisol | 2005 | Almeida, David M; McGonagle, Katherine; King, Heather | Not applicable based on title |
| Associations of trait optimism with diurnal neuroendocrine activity, cortisol responses to mental stress, and subjective stress measures in healthy men and women | 2011 | Endrighi, Romano; Hamer, Mark; Steptoe, Andrew | Not applicable based on title |
| Optimism, positive affectivity, and salivary cortisol | 2005 | Lai, Julian C L; Evans, Phil D; Ng, Sik Hung; Chong, Alice M L; Siu, Oswald T; Chan, Cecilia L W; Ho, Samuel M Y; Ho, Rainbow T H; Chan, Plato; Chan, Charles C | Not applicable based on title |
| Mental health outcomes in emerging adults exposed to childhood maltreatment: the moderating role of stress reactivity | 2014 | Hagan, Melissa J; Roubinov, Danielle S; Mistler, Amy Kraft; Luecken, Linda J | Not applicable based on title |
| Effects of Ramadan fasting on physical performance and metabolic, hormonal, and inflammatory parameters in middle-distance runners | 2009 | Chennaoui, Mounir; Desgorces, FranÁois; Drogou, Catherine; Boudjemaa, Bechir; Tomaszewski, Armand; Depiesse, FrÈdÈric; Burnat, Pascal; Chalabi, Hakim; Gomez-Merino, Danielle | Included |
| Where's the impairment: an examination of factors that impact sustained attention following a stressor | 2005 | Banks, Jonathan B; Tartar, Jaime L; Welhaf, Matthew S | Not applicable based on title |
| Acute social stress increases biochemical and self report markers of stress without altering spatial learning in humans | 2005 | Klopp, Christine; Garcia, Carlos; Schulman, Allan H; Ward, Christopher P; Tartar, Jaime L | Not applicable based on title |
| High testosterone levels and sensitivity to acute stress in perpetrators of domestic violence with low cognitive flexibility and impairments in their emotional decoding process: a preliminary study | 2013 | Romero-MartÌnez, Angel; Lila, Marisol; SariÒana-Gonz·lez, Patricia; Gonz·lez-Bono, Esperanza; Moya-Albiol, Luis | Not applicable based on title |
| Occupational role stress is associated with higher cortisol reactivity to acute stress | 2013 | Wirtz, Petra H; Ehlert, Ulrike; Kottwitz, Maria U; La Marca, Roberto; Semmer, Norbert K | Not applicable based on title |
| Elevated salivary cortisol levels as a result of sleep deprivation in a shift worker | 2003 | Lac, G; Chamoux, A | Not applicable based on title |
| Biobehavioral indices of emotion regulation relate to school attitudes, motivation, and behavior problems in a low-income preschool sample | 2006 | Miller, Alison L; Seifer, Ronald; Stroud, Laura; Sheinkopf, Stephen J; Dickstein, Susan | Not applicable based on title |
| Independent component analysis and source localization on mobile EEG data can identify increased levels of acute stress | 2017 | Schlink, Bryan R; Peterson, Steven M; Hairston, W D; Kˆnig, Peter; Kerick, Scott E; Ferris, Daniel P | Not applicable based on title |
| Associations of salivary cortisol with cognitive function in the Baltimore memory study | 2007 | Lee, Brian K; Glass, Thomas A; McAtee, Matthew J; Wand, Gary S; Bandeen-Roche, Karen; Bolla, Karen I; Schwartz, Brian S | Not applicable based on title |
| Stress reactivity and personality in extreme sport athletes: The psychobiology of BASE jumpers | 2016 | Monasterio, Erik; Mei-Dan, Omer; Hackney, Anthony C; Lane, Amy R; Zwir, Igor; Rozsa, Sandor; Cloninger, C Robert | Not applicable based on title |
| Experiencing Lifetime Domestic Violence: Associations with Mental Health and Stress among Pregnant Women in Rural Bangladesh: The MINIMat Randomized Trial | 2016 | Ziaei, Shirin; Frith, Amy Lynn; Ekstrˆm, Eva-Charlotte; Naved, Ruchira Tabassum | Not applicable based on title |
| Effect of interpersonal and cognitive stressors on habituation and the utility of heart rate variability to measure habituation | 2016 | Feda, Denise M; Roemmich, James N | Not applicable based on title |
| Regulatory skill as a resilience factor for adults with a history of foster care: a pilot study | 2015 | Johnson, Angela J; Tottenham, Nim | Not applicable based on title |
| Biofeedback-based training for stress management in daily hassles: an intervention study | 2014 | Kotozaki, Yuka; Takeuchi, Hikaru; Sekiguchi, Atsushi; Yamamoto, Yuki; Shinada, Takamitsu; Araki, Tsuyoshi; Takahashi, Kei; Taki, Yasuyuki; Ogino, Takeshi; Kiguchi, Masashi; Kawashima, Ryuta | Not applicable based on title |
| Effects of suckling on hypothalamic-pituitary-adrenal axis responses to psychosocial stress in postpartum lactating women | 2001 | Heinrichs, M; Meinlschmidt, G; Neumann, I; Wagner, S; Kirschbaum, C; Ehlert, U; Hellhammer, D H | Not applicable based on title |
| Adrenal hormone response and psychophysiological correlates under psychosocial stress in individuals with irritable bowel syndrome | 2012 | Sugaya, Nagisa; Izawa, Shuhei; Kimura, Kenta; Ogawa, Namiko; Yamada, Kosuke C; Shirotsuki, Kentaro; Mikami, Ikuyo; Hirata, Kanako; Nagano, Yuichiro; Nomura, Shinobu; Shimada, Hironori | Not applicable based on title |
| Posttraumatic Growth in Populations with Posttraumatic Stress Disorder-A Systematic Review on Growth-Related Psychological Constructs and Biological Variables | 2016 | Schubert, Christine F; Schmidt, Ulrike; Rosner, Rita | Not applicable based on title |
| A comparison of changes in secretory immunoglobulin A following a stress-inducing and stress-reducing task | 2009 | Benham, Grant; Nash, Michael R.; Baldwin, Debora R. | Not applicable based on title |
| A randomized trial on mineralocorticoid receptor blockade in men: effects on stress responses, selective attention, and memory | 2011 | Cornelisse, Sandra; JoÎls, Marian; Smeets, Tom | Not applicable based on title |
| Hormonal and neuromuscular responses during a singles match in male professional tennis players | 2018 | LÛpez-Samanes, ¡lvaro; G PallarÈs, Jes˙s; PÈrez-LÛpez, Alberto; Mora-RodrÌguez, Ricardo; Ortega, Juan F | age |
| Childhood poverty and stress reactivity are associated with aberrant functional connectivity in default mode network | 2014 | Sripada, Rebecca K; Swain, James E; Evans, Gary W; Welsh, Robert C; Liberzon, Israel | Not applicable based on title |
| Non-invasive mouthguard biosensor for continuous salivary monitoring of metabolites | 2014 | Kim, Jayoung; ValdÈs-RamÌrez, Gabriela; Bandodkar, Amay J; Jia, Wenzhao; Martinez, Alexandra G; RamÌrez, Julian; Mercier, Patrick; Wang, Joseph | Not applicable based on title |
| DHEA, physical exercise and doping | 2015 | Collomp, K; Buisson, C; Lasne, F; Collomp, R | Not applicable based on title |
| Perfectionism and the cortisol response to psychosocial stress in men | 2007 | Wirtz, Petra H; Elsenbruch, Sigrid; Emini, Luljeta; R¸dis¸li, Katharina; Groessbauer, Sara; Ehlert, Ulrike | Not applicable based on title |
| The serotonin transporter promoter polymorphism is associated with cortisol response to psychosocial stress | 2010 | Way, Baldwin M; Taylor, Shelley E | Not applicable based on title |
| Delayed response and lack of habituation in plasma interleukin-6 to acute mental stress in men | 2006 | von K‰nel, Roland; Kudielka, Brigitte M; Preckel, Daniel; Hanebuth, Dirk; Fischer, Joachim E | Not applicable based on title |
| Stress reactivity in 15-month-old infants: links with infant temperament, cognitive competence, and attachment security | 2004 | van Bakel, Hedwig J A; Riksen-Walraven, J Marianne | Not applicable based on title |
| Do social disadvantage and early family adversity affect the diurnal cortisol rhythm in infants? The Generation R Study | 2010 | Saridjan, Nathalie S; Huizink, Anja C; Koetsier, Jitske A; Jaddoe, Vincent W; Mackenbach, Johan P; Hofman, Albert; Kirschbaum, Clemens; Verhulst, Frank C; Tiemeier, Henning | Not applicable based on title |
| Heritability of cortisol responses to human corticotropin-releasing hormone, ergometry, and psychological stress in humans | 1992 | Kirschbaum, C; W¸st, S; Faig, H G; Hellhammer, D H | Not applicable based on title |
| Social hierarchy and adrenocortical stress reactivity in men | 1997 | Hellhammer, D H; Buchtal, J; Gutberlet, I; Kirschbaum, C | Not applicable based on title |
| Circadian effects on the acute responses of salivary cortisol and IgA in well trained swimmers | 2002 | Dimitriou, L; Sharp, N C C; Doherty, M | Not applicable based on title |
| Cortisol, dehydroepiandrosterone sulphate and dehydroepiandrosterone sulphate/cortisol ratio responses to physical stress in males are influenced by pubertal development | 2006 | Di Luigi, L; Guidetti, L; Baldari, C; Gallotta, M C; SgrÚ, P; Perroni, F; Romanelli, F; Lenzi, A | Not applicable based on title |
| Salivary extracellular heat shock protein 70 (eHSP70) levels increase after 59 min of intense exercise and correlate with resting salivary secretory immunoglobulin A (SIgA) levels at rest | 2016 | Murase, Yosuke; Shimizu, Kazuhiro; Tanimura, Yuko; Hanaoka, Yukichi; Watanabe, Koichi; Kono, Ichiro; Miyakawa, Shumpei | Not applicable based on title |
| Effects of acute social stress on alcohol consumption in healthy subjects | 2003 | de Wit, Harriet; Sˆderpalm, Anna H V; Nikolayev, Lilia; Young, Elizabeth | Not applicable based on title |
| The low single nucleotide polymorphism heritability of plasma and saliva cortisol levels | 2017 | Neumann, Alexander; Direk, Nese; Crawford, Andrew A; Mirza, Saira; Adams, Hieab; Bolton, Jennifer; Hayward, Caroline; Strachan, David P; Payne, Erin K; Smith, Jennifer A; Milaneschi, Yuri; Penninx, Brenda; Hottenga, Jouke J; de Geus, Eco; Oldehinkel, Albertine J; van der Most, Peter J; de Rijke, Yolanda; Walker, Brian R; Tiemeier, Henning | Not applicable based on title |
| Increased sympathetic and decreased parasympathetic activity rather than changes in hypothalamic-pituitary-adrenal axis activity is associated with metabolic abnormalities | 2010 | Licht, Carmilla M M; Vreeburg, Sophie A; van Reedt Dortland, Arianne K B; Giltay, Erik J; Hoogendijk, Witte J G; DeRijk, Roel H; Vogelzangs, Nicole; Zitman, Frans G; de Geus, Eco J C; Penninx, Brenda W J H | Not applicable based on title |
| The Very Low-Dose Dexamethasone Suppression Test in the General Population: A Cross-Sectional Study | 2016 | Direk, Nese; Dekker, Marieke J H J; Luik, Annemarie I; Kirschbaum, Clemens; de Rijke, Yolanda B; Hofman, Albert; Hoogendijk, Witte J G; Tiemeier, Henning | Not applicable based on title |
| A novel biomarker associated with distress in humans: calcium-binding protein, spermatid-specific 1 (CABS1) | 2017 | Ritz, Thomas; Rosenfield, David; St Laurent, Chris D; Trueba, Ana F; Werchan, Chelsey A; Vogel, Pia D; Auchus, Richard J; Reyes-Serratos, Eduardo; Befus, A Dean | Not applicable based on title |
| Two novel genetic variants in the mineralocorticoid receptor gene associated with spontaneous preterm birth | 2015 | Christiaens, Inge; Ang, Q Wei; Gordon, Lindsay N; Fang, Xin; Williams, Scott M; Pennell, Craig E; Olson, David M | Not applicable based on title |
| Interindividual differences in stress sensitivity: basal and stress-induced cortisol levels differentially predict neural vigilance processing under stress | 2016 | Henckens, Marloes J A G; Klumpers, Floris; Everaerd, Daphne; Kooijman, Sabine C; van Wingen, Guido A; Fern·ndez, GuillÈn | Not applicable based on title |
| Acute stress and cardiovascular health: is there an ACE gene connection? | 2012 | Holman, E Alison | Not applicable based on title |
| Changes in cortisol and dehydroepiandrosterone in women victims of physical and psychological intimate partner violence | 2004 | Pico-Alfonso, Maria A; Garcia-Linares, M Isabel; Celda-Navarro, Nuria; Herbert, Joe; Martinez, Manuela | Not applicable based on title |
| Sympathetic and hypothalamic-pituitary-adrenal asymmetry in generalized anxiety disorder | 2016 | Reeves, Jonathan W; Fisher, Aaron J; Newman, Michelle G; Granger, Douglas A | Not applicable based on title |
| Acute stress, memory, attention and cortisol | 2000 | Vedhara, K; Hyde, J; Gilchrist, I D; Tytherleigh, M; Plummer, S | Not applicable based on title |
| Low social support and poor emotional regulation are associated with increased stress hormone reactivity to mental stress in systemic hypertension | 2006 | Wirtz, Petra H; von K‰nel, Roland; Mohiyeddini, Changiz; Emini, Luljeta; Ruedisueli, Katharina; Groessbauer, Sara; Ehlert, Ulrike | Not applicable based on title |
| Circulating cytokine signatures in healthy medical students exposed to academic examination stress | 2012 | Kamezaki, Yoshiko; Katsuura, Sakurako; Kuwano, Yuki; Tanahashi, Toshihito; Rokutan, Kazuhito | Not applicable based on title |
| Acute and chronic stress increase salivary cortisol: a study in the real-life setting of a national examination undertaken by medical graduates | 2014 | Gonz·lez-Cabrera, J; Fern·ndez-Prada, M; Iribar-Ibabe, C; Peinado, J M | Not applicable based on title |
| Neuroendocrine stress responses to an oral academic examination: No strong influence of sex, repeated participation and personality traits | 2008 | Schoofs, D; Hartmann, R; Wolf, O T | Not applicable based on title |
| The quantification of training load, the training response and the effect on performance | 2005 | Borresen, Jill; Lambert, Michael Ian | Not applicable based on title |
| An experimental investigation into the extent social evaluation anxiety impairs performance in simulation-based learning environments amongst final-year undergraduate nursing students | 2016 | Mills, Brennen; Carter, Owen; Rudd, Cobie; Claxton, Louise; O'Brien, Robert | Not applicable based on title |
| Monitoring training load to understand fatigue in athletes | 2014 | Halson, Shona L | Not applicable based on title |
| Stress management training for surgeons-a randomized, controlled, intervention study | 2011 | Wetzel, Cordula M; George, Akram; Hanna, George B; Athanasiou, Thanos; Black, Stephen A; Kneebone, Roger L; Nestel, Debra; Woloshynowych, Maria | Not applicable based on title |
| High-fidelity simulator technology may not be superior to traditional low-fidelity equipment for neonatal resuscitation training | 2012 | Finan, E; Bismilla, Z; Whyte, H E; Leblanc, V; McNamara, P J | Not applicable based on title |
| Cortisol awakening response, internalizing symptoms, and life satisfaction in emerging adults | 2017 | Chong, Li Shen; Thai, Michelle; Cullen, Kathryn R; Lim, Kelvin O; Klimes-Dougan, Bonnie | Not applicable based on title |
| The Efficacy of Vigorous-Intensity Exercise as an Aid to Smoking Cessation in Adults With High Anxiety Sensitivity: A Randomized Controlled Trial | 2016 | Smits, Jasper A J; Zvolensky, Michael J; Davis, Michelle L; Rosenfield, David; Marcus, Bess H; Church, Timothy S; Powers, Mark B; Frierson, Georita M; Otto, Michael W; Hopkins, Lindsey B; Brown, Richard A; Baird, Scarlett O | Not applicable based on title |
| Human herpesvirus 6 and 7 are biomarkers for fatigue, which distinguish between physiological fatigue and pathological fatigue | 2016 | Aoki, Ryo; Kobayashi, Nobuyuki; Suzuki, Go; Kuratsune, Hirohiko; Shimada, Kazuya; Oka, Naomi; Takahashi, Mayumi; Yamadera, Wataru; Iwashita, Masayuki; Tokuno, Shinichi; Nibuya, Masashi; Tanichi, Masaaki; Mukai, Yasuo; Mitani, Keiji; Kondo, Kazuhiro; Ito, Hiroshi; Nakayama, Kazuhiko | Not applicable based on title |
| Mindfulness-based resilience training to reduce health risk, stress reactivity, and aggression among law enforcement officers: A feasibility and preliminary efficacy trial | 2018 | Christopher, Michael S; Hunsinger, Matthew; Goerling, Lt Richard J; Bowen, Sarah; Rogers, Brant S; Gross, Cynthia R; Dapolonia, Eli; Pruessner, Jens C | Not applicable based on title |
| Intense physical exercise increases systemic 11beta-hydroxysteroid dehydrogenase type 1 activity in healthy adult subjects | 2010 | Dovio, Andrea; Roveda, Eliana; Sciolla, Chiara; Montaruli, Angela; Raffaelli, Andrea; Saba, Alessandro; Calogiuri, Giovanna; De Francia, Silvia; Borrione, Paolo; Salvadori, Piero; Carandente, Franca; Angeli, Alberto | Not applicable based on title |
| Psychological and physiological markers of stress in concussed athletes across recovery milestones | 2005 | Hutchison, Michael G; Mainwaring, Lynda; Senthinathan, Arrani; Churchill, Nathan; Thomas, Scott; Richards, Doug | Not applicable based on title |
| Lost in translation? The potential psychobiotic Lactobacillus rhamnosus (JB-1) fails to modulate stress or cognitive performance in healthy male subjects | 2017 | Kelly, John R; Allen, Andrew P; Temko, Andriy; Hutch, William; Kennedy, Paul J; Farid, Niloufar; Murphy, Eileen; Boylan, Geraldine; Bienenstock, John; Cryan, John F; Clarke, Gerard; Dinan, Timothy G | Not applicable based on title |
| Salivary cortisol concentrations, stress and quality of life in women with endometriosis and chronic pelvic pain | 2008 | Petrelluzzi, K F S; Garcia, M C; Petta, C A; Grassi-Kassisse, D M; Spadari-Bratfisch, R C | Not applicable based on title |
| Altered functioning of the HPA axis in depressed postpartum women | 2016 | de Rezende, Marcos GonÁalves; Garcia-Leal, Cybele; de Figueiredo, Felipe Pinheiro; Cavalli, Ricardo de Carvalho; Spanghero, Maristela Schaufelberger; Barbieri, Marco Antonio; Bettiol, Heloisa; de Castro, Margaret; Del-Ben, Cristina Marta | Not applicable based on title |
| Interaction between genetic variants and exposure to Hurricane Katrina on post-traumatic stress and post-traumatic growth: a prospective analysis of low income adults | 2014 | Dunn, Erin C; Solovieff, Nadia; Lowe, Sarah R; Gallagher, Patience J; Chaponis, Jonathan; Rosand, Jonathan; Koenen, Karestan C; Waters, Mary C; Rhodes, Jean E; Smoller, Jordan W | Not applicable based on title |
| Elevated salivary IgA, decreased anxiety, and an altered oral microbiota are associated with active participation on an undergraduate athletic team | 2017 | Lamb, Ashley L; Hess, Debra E; Edenborn, Sherie; Ubinger, Elizabeth; Carrillo, Andres E; Appasamy, Pierette M | Not applicable based on title |
| Peripheral DNA methylation of HPA axis-related genes in humans: Cross-tissue convergence, two-year stability and behavioural and neural correlates | 2018 | Di Sante, Jessica; Ismaylova, Elmira; Nemoda, Zsofia; Gouin, Jean-Philippe; Yu, Wei-Jo; Caldwell, Warren; Vitaro, Frank; Szyf, Moshe; Tremblay, Richard E; Booij, Linda | Not applicable based on title |
| Cortisol response to an experimental stress paradigm prospectively predicts long-term distress and resilience trajectories in response to active police service | 2014 | Galatzer-Levy, Isaac R; Steenkamp, Maria M; Brown, Adam D; Qian, Meng; Inslicht, Sabra; Henn-Haase, Clare; Otte, Christian; Yehuda, Rachel; Neylan, Thomas C; Marmar, Charles R | Not applicable based on title |
| Stressful military training: endocrine reactivity, performance, and psychological impact | 2007 | Taylor, Marcus K; Sausen, Kenneth P; Potterat, Eric G; Mujica-Parodi, Lilianne R; Reis, Jared P; Markham, Amanda E; Padilla, Genieleah A; Taylor, Deborah L | Included |
| Accelerated muscle contractility and decreased muscle steadiness following sauna recovery do not induce greater neuromuscular fatigability during sustained submaximal contractions | 2019 | Cernych, Margarita; Baranauskiene, Neringa; Vitkauskiene, Astra; Satas, Andrius; Brazaitis, Marius | Not applicable based on title |
| Psychosocial stress reactivity is associated with decreased whole-brain network efficiency and increased amygdala centrality | 2018 | Wheelock, Muriah D; Rangaprakash, Deshpande; Harnett, Nathaniel G; Wood, Kimberly H; Orem, Tyler R; Mrug, Sylvie; Granger, Douglas A; Deshpande, Gopikrishna; Knight, David C | Not applicable based on title |
| Effects of internet-based stress management on acute cortisol stress reactivity: Preliminary evidence using the Trier Social Stress Test for Groups (TSST-G) | 2019 | Domes, Gregor; St‰chele, Tobias; von Dawans, Bernadette; Heinrichs, Markus | Not applicable based on title |
| Salivary markers of inflammation in response to acute stress | 2015 | Slavish, Danica C; Graham-Engeland, Jennifer E; Smyth, Joshua M; Engeland, Christopher G | Not applicable based on title |
| Mental fatigue impairs technical performance and alters neuroendocrine and autonomic responses in elite young basketball players | 2018 | Moreira, Alexandre; Aoki, Marcelo Saldanha; Franchini, Emerson; da Silva Machado, Daniel Gomes; Paludo, Ana Carolina; Okano, Alexandre Hideki | Not applicable based on title |
| Salivary Cortisol Responses and Perceived Exertion during High Intensity and Low Intensity Bouts of Resistance Exercise | 2004 | McGuigan, Michael R; Egan, Alison D; Foster, Carl | Not applicable based on title |
| Force-on-Force handgun practice: an intra-individual exploration of stress effects, biomarker regulation, and behavioral changes | 2014 | Taverniers, John; De Boeck, Pieter | Not applicable based on title |
| Psychobiological stress response to a simulated school shooting in police officers | 2015 | Strahler, Jana; Ziegert, Thomas | Not applicable based on title |
| The Relationship between Cortisol Activity during Cognitive Task and Posttraumatic Stress Symptom Clusters | 2015 | Duan, Hongxia; Wang, Li; Zhang, Liang; Liu, Jing; Zhang, Kan; Wu, Jianhui | Not applicable based on title |
| Physiological and self-assessed psychological stress induced by a high fidelity simulation course among third year anesthesia and critical care residents: An observational study | 2017 | Geeraerts, Thomas; Roulleau, Philippe; Cheisson, GaÎlle; Marhar, Fouad; Aidan, Karl; Lallali, Karim; Leguen, Morgan; Schnell, David; Trabold, Fabien; Fauquet-Alekhine, Philippe; Duranteau, Jacques; Benhamou, Dan | Not applicable based on title |
| Age-related changes in the time course of vigilant attention during 40 hours without sleep in men | 2006 | Adam, Martin; RÈtey, Julia V; Khatami, Ramin; Landolt, Hans-Peter | Not applicable based on title |
| Prospective Assessment of Acute Blood Markers of Brain Injury in Sport-Related Concussion | 2017 | Meier, Timothy B; Nelson, Lindsay D; Huber, Daniel L; Bazarian, Jeffrey J; Hayes, Ronald L; McCrea, Michael A | Not applicable based on title |
| Acute stress alters the 'default' brain processing | 2019 | Zhang, Wei; Hashemi, Mahur M; Kaldewaij, Reinoud; Koch, Saskia B J; Beckmann, Christian; Klumpers, Floris; Roelofs, Karin | Not applicable based on title |
| Awakening effects of blue-enriched morning light exposure on university students' physiological and subjective responses | 2019 | Choi, Kyungah; Shin, Cheong; Kim, Taesu; Chung, Hyun Jung; Suk, Hyeon-Jeong | Not applicable based on title |
| Salivary cortisol concentration after high-intensity interval exercise: Time of day and chronotype effect | 2017 | Bonato, Matteo; La Torre, Antonio; Saresella, Marina; Marventano, Ivana; Merati, Giampiero; Vitale, Jacopo Antonino | Not applicable based on title |
| Effects of Mindfulness Practice on Performance-Relevant Parameters and Performance Outcomes in Sports: A Meta-Analytical Review | 2017 | B¸hlmayer, Lucia; Birrer, Daniel; Rˆthlin, Philipp; Faude, Oliver; Donath, Lars | Not applicable based on title |
| Chewing gum alleviates negative mood and reduces cortisol during acute laboratory psychological stress | 2009 | Scholey, Andrew; Haskell, Crystal; Robertson, Bernadette; Kennedy, David; Milne, Anthea; Wetherell, Mark | Not applicable based on title |
| Chewing gum moderates multi-task induced shifts in stress, mood, and alertness A re-examination | 2011 | Johnson, Andrew J; Jenks, Rebecca; Miles, Christopher; Albert, Michael; Cox, Martin | Not applicable based on title |
| The role of coach-athlete relationship quality in team sport athletes' psychophysiological exhaustion: implications for physical and cognitive performance | 2018 | Davis, Louise; Appleby, Ralph; Davis, Paul; Wetherell, Mark; Gustafsson, Henrik | Not applicable based on title |
| The effects of acute stress on human prefrontal working memory systems | 2008 | Porcelli, Anthony J; Cruz, Daniel; Wenberg, Karen; Patterson, Michael D; Biswal, Bharat B; Rypma, Bart | Not applicable based on title |
| Cognitive performance in high-altitude Andean residents compared with low-altitude populations: from childhood to older age | 2014 | Hill, Catherine M; Dimitriou, Dagmara; Baya, Ana; Webster, Rebecca; Gavlak-Dingle, Johanna; Lesperance, Veline; Heathcote, Kate; Bucks, Romola S | Not applicable based on title |
| The COMT Val158Met polymorphism modulates working memory performance under acute stress | 2012 | Buckert, Magdalena; Kudielka, Brigitte M; Reuter, Martin; Fiebach, Christian J | Not applicable based on title |
| Unhealthy lifestyle in early psychoses: the role of life stress and the hypothalamic-pituitary-adrenal axis | 2014 | Manzanares, N˙ria; Monseny, Rosa; Ortega, Laura; Montalvo, Itziar; Franch, Joan; GutiÈrrez-Zotes, Alfonso; Reynolds, Rebecca M; Walker, Brian R; Vilella, Elisabet; Labad, Javier | Not applicable based on title |
| Chronotype, physical activity, and sport performance: A systematic review | 2017 | Vitale, Jacopo Antonino; Weydahl, Andi | Not applicable based on title |
| The effects of time of day and chronotype on cognitive and physical performance in healthy volunteers | 2018 | Facer-Childs, Elise R; Boiling, Sophie; Balanos, George M | Not applicable based on title |
| Epigenetic alterations and prenatal maternal depression | 2017 | Nemoda, Zsofia; Szyf, Moshe | Not applicable based on title |
| Scheduling of training and recovery during the in-season weekly micro-cycle: Insights from team sport practitioners | 2019 | Cross, Rebecca; Siegler, Jason; Marshall, Paul; Lovell, Ric | Not applicable based on title |
| Trait and state rumination interact to prolong cortisol activation to psychosocial stress in females | 2016 | Shull, Alexa; Mayer, Stefanie E; McGinnis, Ellen; Geiss, Elisa; Vargas, Ivan; Lopez-Duran, Nestor L | Not applicable based on title |
| Effects of simulated domestic and international air travel on sleep, performance, and recovery for team sports | 2015 | Fowler, P; Duffield, R; Vaile, J | Not applicable based on title |
| The relationship between the menstrual cycle and cortisol secretion: Daily and stress-invoked cortisol patterns | 2018 | Montero-LÛpez, Eva; Santos-Ruiz, Ana; GarcÌa-RÌos, M Carmen; RodrÌguez-Bl·zquez, Manuel; Rogers, Heather L; Peralta-RamÌrez, MarÌa Isabel | Not applicable based on title |
| Using biofeedback while immersed in a stressful videogame increases the effectiveness of stress management skills in soldiers | 2012 | Bouchard, StÈphane; Bernier, FranÁois; Boivin, Eric; Morin, Brian; Robillard, GeneviËve | Not applicable based on title |
| Cortisol and politics: variance in voting behavior is predicted by baseline cortisol levels | 2014 | French, Jeffrey A; Smith, Kevin B; Alford, John R; Guck, Adam; Birnie, Andrew K; Hibbing, John R | Not applicable based on title |
| Protein intake during training sessions has no effect on performance and recovery during a strenuous training camp for elite cyclists | 2016 | Hansen, Mette; Bangsbo, Jens; Jensen, J¯rgen; Krause-Jensen, Matilde; Bibby, Bo Martin; Sollie, Ove; Hall, Ulrika Andersson; Madsen, Klavs | Not applicable based on title |
| Stress system response and decision making in heavy episodic users of alcohol and online video games | 2019 | Gilbertson, Rebecca J; Leff, Dustyn J; Young, Nathan A | Not applicable based on title |
| Exploration of psychological mechanisms of the reduced stress response in long-term meditation practitioners | 2019 | Gamaiunova, Liudmila; Brandt, Pierre-Yves; Bondolfi, Guido; Kliegel, Matthias | Not applicable based on title |
| Salivary biomarkers and training load during training and competition in paralympic swimmers | 2018 | Sinnott-O'Connor, Ciara; Comyns, Thomas M; Nevill, Alan M; Warrington, Giles D | Not applicable based on title |
| The Trier Social Stress Test and the Trier Social Stress Test for groups: Qualitative investigations | 2018 | Vors, Olivier; Marqueste, Tanguy; Mascret, Nicolas | Not applicable based on title |
| Effects of oral contraceptive use on the salivary testosterone and cortisol responses to training sessions and competitions in elite women athletes | 2015 | Crewther, Blair T; Hamilton, Dave; Casto, Kathleen; Kilduff, Liam P; Cook, Christian J | Not applicable based on title |
| Transdermal nicotine attenuates depression symptoms in nonsmokers: a double-blind, placebo-controlled trial | 2006 | McClernon, F Joseph; Hiott, F Berry; Westman, Eric C; Rose, Jed E; Levin, Edward D | Not applicable based on title |
| Recovery and performance in sport: consensus statement | 2018 | Kellmann, Michael; Bertollo, Maurizio; Bosquet, Laurent; Brink, Michel; Coutts, Aaron J; Duffield, Rob; Erlacher, Daniel; Halson, Shona L; Hecksteden, Anne; Heidari, Jahan; Kallus, K Wolfgang; Meeusen, Romain; Mujika, IÒigo; Robazza, Claudio; Skorski, Sabrina; Venter, Ranel; Beckmann, J¸rgen | Not applicable based on title |
| The effects of energy balance, obesity-proneness and sex on the neuronal response to sweet taste | 2015 | Cornier, Marc-Andre; Shott, Megan E; Thomas, Elizabeth A; Bechtell, Jamie L; Bessesen, Daniel H; Tregellas, Jason R; Frank, Guido K | Not applicable based on title |
| Reactivity to television food commercials in overweight and lean adults: Physiological, cognitive and behavioural responses | 2017 | Boyland, Emma J; Burgon, Rachel H; Hardman, Charlotte A | Not applicable based on title |
| Pre-scan cortisol is differentially associated with enhanced connectivity to the cognitive control network in young adults with a history of depression | 2019 | Peters, Amy T; Jenkins, Lisanne M; Stange, Jonathan P; Bessette, Katie L; Skerrett, Kristy A; Kling, Leah R; Welsh, Robert C; Milad, Mohammed R; Phan, Kinh L; Langenecker, Scott A | Not applicable based on title |
| Space motion sickness: A common neurovestibular dysfunction in microgravity | 2005 | Russomano, Thais; da Rosa, Michele; Dos Santos, Marlise A | Not applicable based on title |
| Dose-dependent effects of endotoxin on neurobehavioral functions in humans | 2011 | Grigoleit, Jan-Sebastian; Kullmann, Jennifer S; Wolf, Oliver T; Hammes, Florian; Wegner, Alexander; Jablonowski, Stephanie; Engler, Harald; Gizewski, Elke; Oberbeck, Reiner; Schedlowski, Manfred | Not applicable based on title |
| Sleep deprivation and a non-24-h working schedule lead to extensive alterations in physiology and behavior | 2019 | Ma, Huan; Li, Yunzhen; Liang, Haojian; Chen, Shijunyin; Pan, Siyu; Chang, Lulin; Li, Silin; Zhang, Ying; Liu, Xinyan; Xu, Yanwen; Shao, Yongcong; Yang, Yebing; Guo, Jinhu | Not applicable based on title |
| Cortisol affects pain sensitivity and pain-related emotional learning in experimental visceral but not somatic pain: a randomized controlled study in healthy men and women | 2019 | Benson, Sven; Siebert, Carsten; Koenen, Laura R; Engler, Harald; Kleine-Borgmann, Julian; Bingel, Ulrike; Icenhour, Adriane; Elsenbruch, Sigrid | Not applicable based on title |
| Stress during pregnancy is associated with developmental outcome in infancy | 2003 | Huizink, Anja C.; Robles de Medina, Pascale G.; Mulder, Eduard J.H.; Visser, Gerard H.A.; Buitelaar, Jan K. | Not applicable based on title |
| Increased systolic blood pressure reactivity to acute stress is related with better self-reported health | 2014 | Wright, Bradley J; O'Brien, Shaun; Hazi, Agnes; Kent, Stephen | Not applicable based on title |
| Distress intolerance moderation of neurophysiological markers of response inhibition after induced stress: Relations with cannabis use disorder | 2018 | Macatee, Richard J; Albanese, Brian J; Crane, Natania A; Okey, Sarah A; Cougle, Jesse R; Schmidt, Norman B | Not applicable based on title |
| Early father-infant skin-to-skin contact and its effect on the neurodevelopmental outcomes of moderately preterm infants in China: study protocol for a randomized controlled trial | 2018 | Deng, Qingqi; Li, Qiufang; Wang, Hua; Sun, Huilian; Xu, Xinfen | Not applicable based on title |
| A continuous mental task decreases the physiological response to soccer-specific intermittent exercise | 2007 | Greig, Matt; Marchant, David; Lovell, Richard; Clough, Peter; McNaughton, Lars | Not applicable based on title |
| Longer-term increased cortisol levels in young people with mental health problems | 2016 | Heinze, Kareen; Lin, Ashleigh; Reniers, Renate L E P; Wood, Stephen J | Not applicable based on title |
| Central slowing during the night | 2007 | Bratzke, Daniel; Rolke, Bettina; Ulrich, Rolf; Peters, Maren | Not applicable based on title |
| Why egalitarianism might be good for your health: physiological thriving during stressful intergroup encounters | 2007 | Mendes, Wendy Berry; Gray, Heather M; Mendoza-Denton, Rodolfo; Major, Brenda; Epel, Elissa S | Not applicable based on title |
| A neurobiological correlate of stress-induced nicotine-seeking behavior among cigarette smokers | 2020 | Woodcock, Eric A; Stanley, Jeffrey A; Diwadkar, Vaibhav A; Khatib, Dalal; Greenwald, Mark K | Not applicable based on title |
| Increased cortisol levels in cognitively challenging situations are beneficial in young but not older subjects | 2008 | Kukolja, Juraj; Thiel, Christiane M; Wolf, Oliver T; Fink, Gereon R | Not applicable based on title |
| Trans-generational stress regulation: Mother-infant cortisol and maternal mental health across the perinatal period | 2019 | Galbally, Megan; van Rossum, Elisabeth F C; Watson, Stuart J; de Kloet, Edo Ronald; Lewis, Andrew J | Not applicable based on title |
| [development and application of an overcoming compassion fatigue program for emergency nurses] | 2016 | Kim, Yeong Ah; Park, Jeong Sook | Not applicable based on title |
| Associations of monoamine oxidase A gene first exon methylation with sexual abuse and current depression in women | 2018 | Checknita, David; Ekstrˆm, Tomas J; Comasco, Erika; Nilsson, Kent W; Tiihonen, Jari; Hodgins, Sheilagh | Not applicable based on title |
| Patterns of cortisol and alpha-amylase reactivity to psychosocial stress in maltreated women | 2017 | Mielock, Alyssa S; Morris, Matthew C; Rao, Uma | Not applicable based on title |
| Socially oriented thinking and the biological stress response: Thinking of friends and family predicts trajectories of salivary cortisol decline | 2019 | Vine, Vera; Hilt, Lori M; MarroquÌn, Brett; Gilbert, Kirsten E | Not applicable based on title |
| High volume exercise training in older athletes influences inflammatory and redox responses to acute exercise | 2017 | Estrela, AndrÈ L; Zaparte, Aline; da Silva, Jeferson D; Moreira, JosÈ Cl·udio; Turner, James E; Bauer, MoisÈs E | Not applicable based on title |
| Individual differences in the cortisol response to stress in young healthy men: testing the roles of perceived stress reactivity and threat appraisal using multiphase latent growth curve modeling | 2011 | Schlotz, Wolff; Hammerfald, Karin; Ehlert, Ulrike; Gaab, Jens | Not applicable based on title |
| Persistent effects of cognitive-behavioral stress management on cortisol responses to acute stress in healthy subjects--a randomized controlled trial | 2006 | Hammerfald, K; Eberle, C; Grau, M; Kinsperger, A; Zimmermann, A; Ehlert, U; Gaab, J | Not applicable based on title |
| Stress management skills, cortisol awakening response, and post-exertional malaise in Chronic Fatigue Syndrome | 2014 | Hall, Daniel L; Lattie, Emily G; Antoni, Michael H; Fletcher, Mary Ann; Czaja, Sara; Perdomo, Dolores; Klimas, Nancy G | Not applicable based on title |
| Prenatal stress and cognitive development and temperament in infants | 2003 | Buitelaar, Jan K; Huizink, Anja C; Mulder, Edu J; de Medina, Pascalle G Robles; Visser, Gerard H A | Not applicable based on title |
| Reductions in salivary cortisol are associated with mood improvement during relaxation training among HIV-seropositive men | 2000 | Cruess, D G; Antoni, M H; Kumar, M; Schneiderman, N | Not applicable based on title |
| Concurrent levels of maternal salivary cortisol are unrelated to self-reported psychological measures in low-risk pregnant women | 2013 | Voegtline, Kristin M; Costigan, Kathleen A; Kivlighan, Katie T; Laudenslager, Mark L; Henderson, Janice L; DiPietro, Janet A | Not applicable based on title |
| Cardiovascular responses to an acute psychological stressor are associated with the cortisol awakening response in individuals with chronic neck pain | 2015 | Shahidi, Bahar; Sannes, Timothy; Laudenslager, Mark; Maluf, Katrina S | Not applicable based on title |
| Opposite effects of stress on pain modulation depend on the magnitude of individual stress response | 2018 | Geva, Nirit; Defrin, Ruth | Not applicable based on title |
| Adult cognitive ability and socioeconomic status as mediators of the effects of childhood disadvantage on salivary cortisol in aging adults | 2013 | Franz, Carol E; Spoon, Kelly; Thompson, Wesley; Hauger, Richard L; Hellhammer, Dirk H; Jacobson, Kristen C; Lupien, Sonia; Lyons, Michael J; McCaffery, Jeanne; McKenzie, Ruth; Mendoza, Sally P; Panizzon, Matthew S; Ramundo, Ana; Shahroudi, Afrand; Kremen, William S | Not applicable based on title |
| Affective responses to self-selected and imposed walking in inactive women with high stress: a pilot study | 2013 | Wardwell, K K; Focht, B C; Courtney Devries, A; O'connell, A A; Buckworth, J | Not applicable based on title |
| Association of secondhand smoke exposure with mental health in men and women: cross-sectional and prospective analyses using the UK Health and Lifestyle Survey | 2013 | Lam, E; Kvaavik, E; Hamer, M; Batty, G D | Not applicable based on title |
| Behavioral and physiological correlates of stress related to examination performance in college chemistry students | 2011 | Bardi, M; Koone, T; Mewaldt, S; O'Connor, K | Not applicable based on title |
| Copeptin as a stress marker prior and after a written examination--the CoEXAM study | 2015 | Urwyler, Sandrine A; Schuetz, Philipp; Sailer, Clara; Christ-Crain, Mirjam | Not applicable based on title |
| Cortisol and cognitive function in midlife: the role of childhood cognition and educational attainment | 2014 | Gaysina, Darya; Gardner, Michael P; Richards, Marcus; Ben-Shlomo, Yoav | Not applicable based on title |
| Early calibration of the HPA axis by maternal psychopathology | 2017 | Laurent, Heidemarie | Not applicable based on title |
| The effect of communication skills training on residents' physiological arousal in a breaking bad news simulated task | 2013 | Meunier, Julie; Merckaert, Isabelle; Libert, Yves; Delvaux, Nicole; Etienne, Anne-Marie; LiÈnard, Aurore; Bragard, Isabelle; Marchal, Serge; Reynaert, Christine; Slachmuylder, Jean-Louis; Razavi, Darius | Not applicable based on title |
| Effects of a psychosocial couple-based prevention program on adverse birth outcomes | 2015 | Feinberg, Mark E; Roettger, Michael E; Jones, Damon E; Paul, Ian M; Kan, Marni L | Not applicable based on title |
| Effects of simulation versus traditional tutorial-based training on physiologic stress levels among clinicians: a pilot study | 2010 | Bong, Choon Looi; Lightdale, Jenifer R; Fredette, Meghan E; Weinstock, Peter | Not applicable based on title |
| Effects of two dominance manipulations on the stress response: Cognitive and embodied influences | 2016 | Deuter, Christian Eric; Sch‰chinger, Hartmut; Best, Daniel; Neumann, Roland | Not applicable based on title |
| Infant hair cortisol: associations with salivary cortisol and environmental context | 2005 | Flom, Megan; St John, Ashley M; Meyer, Jerrold S; Tarullo, Amanda R | Not applicable based on title |
| Low subjective social status in the police is linked to health-relevant changes in diurnal salivary alpha-amylase activity in Swiss police officers | 2018 | Habersaat, StÈphanie; Abdellaoui, Sid; Geiger, Ashley M; Urben, SÈbastien; Wolf, Jutta M | Not applicable based on title |
| Men with chronic occupational stress benefit from behavioural/psycho-educational group training: a randomized, prospective, controlled trial | 2007 | Nickel, C; Tanca, S; Kolowos, S; Pedrosa-Gil, F; Bachler, E; Loew, T H; Gross, M; Rother, W K; Nickel, M K | Not applicable based on title |
| Morning salivary cortisol and cognitive function in mid-life: evidence from a population-based birth cohort | 2012 | Geoffroy, M C; Hertzman, C; Li, L; Power, C | Not applicable based on title |
| Parental stress management using relaxation techniques in a neonatal intensive care unit: A randomised controlled trial | 2016 | Fotiou, Catherine; Vlastarakos, Petros V; Bakoula, Chrysa; Papagaroufalis, Konstantinos; Bakoyannis, George; Darviri, Christine; Chrousos, George | Not applicable based on title |
| Recovery of rescuers from a 24-h shift and its association with aerobic fitness | 2017 | Lyytik‰inen, Katariina; Toivonen, Leena; Hynynen, Esa; Lindholm, Harri; Kyrˆl‰inen, Heikki | Not applicable based on title |
| Relationship between self-reported mental stressors at the workplace and salivary cortisol | 2008 | Maina, Giovanni; Palmas, Antonio; Filon, Francesca Larese | Not applicable based on title |
| Salivary cortisol and depression in public sector employees: cross-sectional and short term follow-up findings | 2014 | Vammen, Marianne Agergaard; Mikkelsen, Sigurd; Hansen, ≈se Marie; Grynderup, Matias Br¯dsgaard; Andersen, Johan Hviid; Bonde, Jens Peter; Buttensch¯n, Henriette N¯rm¯lle; Kolstad, Henrik Albert; KÊrgaard, Anette; KÊrlev, Linda; Mors, Ole; Rugulies, Reiner; Thomsen, Jane Fr¯lund | Not applicable based on title |
| Salivary cortisol levels and work-related stress among emergency department nurses | 2001 | Yang, Y; Koh, D; Ng, V; Lee, F C; Chan, G; Dong, F; Chia, S E | Not applicable based on title |
| Salivary steroid response and competitive anxiety in elite basketball players: Effect of opponent level | 2017 | Arruda, Ademir F S; Aoki, Marcelo S; Paludo, Ana Carolina; Moreira, Alexandre | Not applicable based on title |
| "Sex differences in a real academic stressor, cognitive appraisal and the cortisol response" | 2017 | Helbig, Susanne; Backhaus, Jutta | Not applicable based on title |
| Stress and cortisol in disaster evacuees: an exploratory study on associations with social protective factors | 2015 | Thompson, David Javier; Weissbecker, Inka; Cash, Elizabeth; Simpson, David M; Daup, Meagan; Sephton, Sandra E | Not applicable based on title |
| Stress and cortisol responses in men: differences according to facial symmetry | 2017 | Borr·z-LeÛn, Javier I; Cerda-Molina, Ana Lilia; Mayagoitia-Novales, Lilian | Not applicable based on title |
| Test-enhanced learning may be a gender-related phenomenon explained by changes in cortisol level | 2011 | Kromann, Charles B; Jensen, Morten L; Ringsted, Charlotte | Not applicable based on title |
| Threat and challenge: cognitive appraisal and stress responses in simulated trauma resuscitations | 2010 | Harvey, Adrian; Nathens, Avery B; Bandiera, Glen; Leblanc, Vicki R | Not applicable based on title |
| Associations between Health Behaviors and Factors on Markers of Healthy Psychological and Physiological Functioning: a Daily Diary Study | 2020 | Strahler, Jana; Nater, Urs M; Skoluda, Nadine | Not applicable based on title |
| Prospective examination of synthetic 5-methoxy-N,N-dimethyltryptamine inhalation: effects on salivary IL-6, cortisol levels, affect, and non-judgment | 2020 | Uthaug, Malin V; Lancelotta, Rafael; Szabo, Attila; Davis, Alan K; Riba, Jordi; Ramaekers, Johannes G | Not applicable based on title |
| Physical fitness and prior physical activity are both associated with less cortisol secretion during psychosocial stress | 2018 | Wood, Carly J; Clow, Angela; Hucklebridge, Frank; Law, Robin; Smyth, Nina | Not applicable based on title |
| Working memory-related frontal theta activity is decreased under acute stress | 2014 | G‰rtner, Matti; Rohde-Liebenau, Lea; Grimm, Simone; Bajbouj, Malek | Not applicable based on title |
| Secretory immunoglobulin A reactions to prolonged mental arithmetic stress: inter-session and intra-session reliability | 2002 | Ring, Christopher; Drayson, Mark; Walkey, Duncan G; Dale, Sarah; Carroll, Douglas | Not applicable based on title |
| Realising the potential of urine and saliva as diagnostic tools in sport and exercise medicine | 2017 | Lindsay, Angus; Costello, Joseph T | Not applicable based on title |
| Stress and stressors of medical student near-peer tutors during courses: a psychophysiological mixed methods study | 2019 | Hundertmark, Jan; Alvarez, Simone; Loukanova, Svetla; Schultz, Jobst-Hendrik | Not applicable based on title |
| Cortisol response to stress: The role of expectancy and anticipatory stress regulation | 2020 | Pulopulos, Matias M; Baeken, Chris; De Raedt, Rudi | Not applicable based on title |
| Resting amygdala connectivity and basal sympathetic tone as markers of chronic hypervigilance | 2019 | Kleshchova, Olena; Rieder, Jenna K; Grinband, Jack; Weierich, Mariann R | Not applicable based on title |
| Inflammatory biomarkers, microbiome, depression, and executive dysfunction in alcohol users | 2020 | RodrÌguez-Rabassa, Mary; LÛpez, Pablo; S·nchez, Raphael; Hern·ndez, Cyanela; RodrÌguez, Cesarly; RodrÌguez-Santiago, Ronald E; Orengo, Juan C; Green, Vivian; Yamamura, Yasuhiro; Rivera-Amill, Vanessa | Not applicable based on title |
| Trait rumination predicts elevated evening cortisol in sexual and gender minority young adults | 2017 | Zoccola, Peggy M; Manigault, Andrew W; Figueroa, Wilson S; Hollenbeck, Cari; Mendlein, Anna; Woody, Alex; Hamilton, Katrina; Scanlin, Matt; Johnson, Ryan C | Not applicable based on title |
| Pathophysiology behind prolonged whiplash associated disorders: study protocol for an experimental study | 2019 | Peolsson, Anneli; Karlsson, Anette; Ghafouri, Bijar; Ebbers, Tino; Engstrˆm, Maria; Jˆnsson, Margaretha; WÂhlÈn, Karin; Romu, Thobias; Borga, Magnus; Kristjansson, Eythor; Bahat, Hilla Sarig; German, Dmitry; Zsigmond, Peter; Peterson, Gunnel | Not applicable based on title |
| Basolateral Amygdala Connectivity With Subgenual Anterior Cingulate Cortex Represents Enhanced Fear-Related Memory Encoding in Anxious Humans | 2020 | Hakamata, Yuko; Mizukami, Shinya; Izawa, Shuhei; Moriguchi, Yoshiya; Hori, Hiroaki; Kim, Yoshiharu; Hanakawa, Takashi; Inoue, Yusuke; Tagaya, Hirokuni | Not applicable based on title |
| Magic Moments: Determinants of Stress Relief and Subjective Wellbeing from Visiting a Cultural Heritage Site | 2019 | Grossi, Enzo; Tavano Blessi, Giorgio; Sacco, Pier Luigi | Not applicable based on title |
| Changes in resting salivary testosterone, cortisol and interleukin-6 as biomarkers of overtraining | 2005 | Anderson, Travis; Haake, Simon; Lane, Amy R; Hackney, Anthony C | Not applicable based on title |
| The cortisol awakening response: association with training load in endurance runners | 2018 | Anderson, Travis; Lane, Amy R; Hackney, Anthony C | Not applicable based on title |
| Differences in relaxation by means of guided imagery in a healthy community sample | 2006 | Watanabe, Eri; Fukuda, Sanae; Hara, Hisako; Maeda, Yuko; Ohira, Hideki; Shirakawa, Taro | Not applicable based on title |
| Minimal effect of acute caffeine ingestion on intense resistance training performance | 2011 | Astorino, Todd A; Martin, Brian J; Schachtsiek, Lena; Wong, Keau; Ng, Karno | Not applicable based on title |
| Nucleus accumbens neurochemistry in human anxiety: A 7 T 1H-MRS study | 2019 | Strasser, Alina; Xin, Lijing; Gruetter, Rolf; Sandi, Carmen | Not applicable based on title |
| Neurophysiological effects of sleep deprivation in healthy adults, a pilot study | 2015 | Klumpers, Ursula M H; Veltman, Dick J; van Tol, Marie-Jose; Kloet, Reina W; Boellaard, Ronald; Lammertsma, Adriaan A; Hoogendijk, Witte J G | Not applicable based on title |
| A single session of hatha yoga improves stress reactivity and recovery after an acute psychological stress task-A counterbalanced, randomized-crossover trial in healthy individuals | 2017 | Benvenutti, Mateus J; Alves, Eduardo da Sliva; Michael, Scott; Ding, Ding; Stamatakis, Emmanuel; Edwards, Kate M | Not applicable based on title |
| Acute psychological and physical stress transiently enhances brachial artery flow-mediated dilation stimulated by exercise-induced increases in shear stress | 2014 | Szijgyarto, Ingrid C; Poitras, Veronica J; Gurd, Brendon J; Pyke, Kyra E | Not applicable based on title |
| Neuroendocrine activation during combined mental and physical stress in women depends on trait anxiety and the phase of the menstrual cycle | 2008 | Hlavacova, Natasa; Wawruch, Martin; Tisonova, Jana; Jezova, Daniela | Not applicable based on title |
| Anabolic hormone profiles in elite military men: Robust associations with age, stress, and fatigue | 2017 | Taylor, Marcus K; Padilla, Genieleah A; Hern·ndez, Lisa M | Included |
| Cardiorespiratory fitness, sleep, and physiological responses to stress in women | 2020 | Crowley, Shannon K; Rebellon, Julia; Huber, Christina; Leonard, Abigail J; Henderson, Daniel; Magal, Meir | Not applicable based on title |
| Psychophysiological responses to the Stroop Task after a maximal cycle ergometry in elite sportsmen and physically active subjects | 2001 | Moya-Albiol, L; Salvador, A; Costa, R; MartÌnez-Sanchis, S; Gonz·lez-Bono, E; Ricarte, J; Arnedo, M | Not applicable based on title |
| Acute effects of power and resistance exercises on hemodynamic measurements of older women | 2017 | Coelho-J˙nior, HÈlio JosÈ; Irigoyen, Maria-Cl·udia; Aguiar, Samuel da Silva; GonÁalves, Ivan de Oliveira; C‚mara, Niels Olsen Saraiva; Cenedeze, Marco Antonio; Asano, Ricardo Yukio; Rodrigues, Bruno; Uchida, Marco Carlos | Not applicable based on title |
| Acute psychosocial stress alters thalamic network centrality | 2019 | Reinelt, Janis; Uhlig, Marie; M¸ller, Karsten; Lauckner, Mark E; Kumral, Deniz; Schaare, H Lina; Baczkowski, Blazej M; Babayan, Anahit; Erbey, Miray; Roebbig, Josefin; Reiter, Andrea; Bae, Yoon-Ju; Kratzsch, Juergen; Thiery, Joachim; Hendler, Talma; Villringer, Arno; Gaebler, Michael | Not applicable based on title |
| Pythagorean self-awareness intervention: A novel cognitive stress management technique for body weight control | 2019 | Simos, Dimitrios S; Kokkinos, Alexandros; Tentolouris, Nikolaos; Dimosthenopoulos, Charilaos; Mantzou, Emily; Artemiadis, Artemios; Bacopoulou, Flora; Nicolaides, Nicolas C; Kosta, Ourania; Chrousos, George P; Darviri, Christina | Not applicable based on title |
| Measuring resilience to operational stress in canadian armed forces personnel | 2018 | Hellewell, Sarah C; Cernak, Ibolja | undefined stressor in criteria |
| Dehydroepiandrosterone and dehydroepiandrosterone sulfate: anabolic, neuroprotective, and neuroexcitatory properties in military men | 2013 | Taylor, Marcus K | Not applicable based on title |
| Effects of dehydroepiandrosterone supplementation during stressful military training: a randomized, controlled, double-blind field study | 2012 | Taylor, Marcus K; Padilla, Genieleah A; Stanfill, Katherine E; Markham, Amanda E; Khosravi, Jasmine Y; Ward, Michael D Dial; Koehler, Matthew M | Not applicable based on title |
| Sex differences in stress and immune responses during confinement in Antarctica | 2019 | Strewe, C; Moser, D; Buchheim, J-I; Gunga, H-C; Stahn, A; Crucian, B E; Fiedel, B; Bauer, H; Gˆssmann-Lang, P; Thieme, D; Kohlberg, E; ChoukËr, A; Feuerecker, M | Not applicable based on title |
| Infant video viewing and salivary cortisol responses: a randomized experiment | 2013 | Christakis, Dimitri A; Liekweg, Kimberly; Garrison, Michelle M; Wright, Jeffrey A | Not applicable based on title |
| Effectiveness of active versus passive recovery strategies after futsal games | 2008 | Tessitore, Antonio; Meeusen, Romain; Pagano, Rita; Benvenuti, Cinzia; Tiberi, Monica; Capranica, Laura | Not applicable based on title |
| Maternal early pregnancy body mass index and diurnal salivary cortisol in young adult offspring | 2019 | Kumpulainen, Satu M; Heinonen, Kati; Kaseva, Nina; Andersson, Sture; Lano, Aulikki; Reynolds, Rebecca M; Wolke, Dieter; Kajantie, Eero; Eriksson, Johan G; R‰ikkˆnen, Katri | Not applicable based on title |
| Effects of exercise on S-IGA and URS in postmenopausal women | 2013 | Sloan, C A; Engels, H J; Fahlman, M M; Yarandi, H E; Davis, J E | Not applicable based on title |
| Exercise versus no exercise for the occurrence, severity, and duration of acute respiratory infections | 2020 | Grande, Antonio Jose; Keogh, Justin; Silva, Valter; Scott, Anna M | Not applicable based on title |
| Cortisol response to repeated psychosocial stress | 2012 | Petrowski, Katja; Wintermann, Gloria-Beatrice; Siepmann, Martin | Not applicable based on title |
| Time-Course of Changes in Physiological, Psychological, and Performance Markers following a Functional-Fitness Competition | 2019 | Tibana, Ramires Alsamir; Prestes, Jonato; DE Sousa, Nuno Manuel Frade; DE Souza, Vinicius Carolino; DE TolÍdo Nobrega, Ot·vio; Baffi, Matheus; Ferreira, Carlos Ernesto S; Cunha, Gabriel Veloso; Navalta, James W; Trombeta, Joice Cristina Dos Santos; Cavaglieri, Cl·udia R; Voltarelli, FabrÌcio Azevedo | Not applicable based on title |
| Special feature for the Olympics: effects of exercise on the immune system: overtraining effects on immunity and performance in athletes | 2000 | MacKinnon, L T | Not applicable based on title |
| Effects of a single session of SMR neurofeedback training on anxiety and cortisol levels | 2020 | Gadea, Marien; AliÒo, Marta; Hidalgo, Vanesa; Espert, Raul; Salvador, Alicia | Not applicable based on title |
| Effects of Different Chair-Based Exercises on Salivary Biomarkers and Functional Autonomy in Institutionalized Older Women | 2019 | Rieping, TaÌs; Furtado, Guilherme Eust·quio; Letieri, Rubens VinÌcius; Chupel, Matheus Uba; Colado, Juan C; Hogervorst, Eef; Filaire, Edith; Teixeira, Ana Maria Miranda Botelho; Ferreira, JosÈ Pedro | Not applicable based on title |
| Influence of a montmorency cherry juice blend on indices of exercise-induced stress and upper respiratory tract symptoms following marathon running--a pilot investigation | 2015 | Dimitriou, Lygeri; Hill, Jessica A; Jehnali, Ahmed; Dunbar, Joe; Brouner, James; McHugh, Malachy P; Howatson, Glyn | Not applicable based on title |
| Multidimensional monitoring of recovery status and implications for performance | 2018 | Heidari, Jahan; Beckmann, J¸rgen; Bertollo, Maurizio; Brink, Michel; Kallus, Wolfgang; Robazza, Claudio; Kellmann, Michael | Not applicable based on title |
| The impact of essential fatty acid, B vitamins, vitamin C, magnesium and zinc supplementation on stress levels in women: a systematic review | 2005 | McCabe, Delia; Lisy, Karolina; Lockwood, Craig; Colbeck, Marc | Not applicable based on title |
| How do stress, sleep quality, and chronotype associate with clinically significant depressive symptoms? A study of young male military recruits in compulsory service | 2020 | Tonon, AndrÈ C; Carissimi, Alicia; Schimitt, Regina L; de Lima, LetÌcia S; Pereira, Fernanda Dos S; Hidalgo, Maria Paz | Not applicable based on title |
| A metacognitive perspective on the cognitive deficits experienced in intellectually threatening environments | 2009 | Schmader, Toni; Forbes, Chad E; Zhang, Shen; Mendes, Wendy Berry | Not applicable based on title |
| Rethinking butterflies: the affective, physiological, and performance effects of reappraising arousal during social evaluation | 2014 | Beltzer, Miranda L; Nock, Matthew K; Peters, Brett J; Jamieson, Jeremy P | Not applicable based on title |
| Efficacy of exercise in reducing depressive symptoms across 5-HTTLPR genotypes | 2010 | Rethorst, Chad D; Landers, Daniel M; Nagoshi, Craig T; Ross, Julianna T D | Not applicable based on title |
| Delayed sleep timing and circadian rhythms in pregnancy and transdiagnostic symptoms associated with postpartum depression | 2020 | Obeysekare, Jessica L; Cohen, Zachary L; Coles, Meredith E; Pearlstein, Teri B; Monzon, Carmen; Flynn, E Ellen; Sharkey, Katherine M | Not applicable based on title |
| Salivary BDNF and Cortisol Responses During High-Intensity Exercise and Official Basketball Matches in Sedentary Individuals and Elite Players | 2018 | Moreira, Alexandre; Aoki, Marcelo Saldanha; de Arruda, Ademir Felipe Schultz; Machado, Daniel Gomes da Silva; Elsangedy, Hassan Mohamed; Okano, Alexandre Hideki | Not applicable based on title |
| Mindfulness versus Physical Exercise: Effects of Two Recovery Strategies on Mental Health, Stress and Immunoglobulin A during Lunch Breaks A Randomized Controlled Trial | 2020 | DÌaz-Silveira, Cintia; Alcover, Carlos-MarÌa; Burgos, Francisco; Marcos, Alberto; Santed, Miguel A | Not applicable based on title |
| Autonomic and hypothalamic-pituitary-adrenal stress resilience: Impact of cardiac vagal tone | 2010 | Smeets, Tom | Not applicable based on title |
| Stress levels during emergency care: A comparison between reality and simulated scenarios | 2016 | Daglius Dias, Roger; Scalabrini Neto, Augusto | Not applicable based on title |
| Loneliness and stress-related inflammatory and neuroendocrine responses in older men and women | 2012 | Hackett, Ruth A; Hamer, Mark; Endrighi, Romano; Brydon, Lena; Steptoe, Andrew | Not applicable based on title |
| Physical activity as treatment for alcohol use disorders (FitForChange): study protocol for a randomized controlled trial | 2018 | Hallgren, Mats; Andersson, Victoria; Ekblom, ÷rjan; AndrÈasson, Sven | Not applicable based on title |
| Toxic stress history and hypothalamic-pituitary-adrenal axis function in a social stress task: Genetic and epigenetic factors | 2005 | Lapp, Hannah E; Ahmed, Sarah; Moore, Celia L; Hunter, Richard G | Not applicable based on title |
| Efficacy of heat-killed Lactococcus lactis JCM 5805 on immunity and fatigue during consecutive high intensity exercise in male athletes: a randomized, placebo-controlled, double-blinded trial | 2018 | Komano, Yuta; Shimada, Kazunori; Naito, Hisashi; Fukao, Kosuke; Ishihara, Yoshihiko; Fujii, Toshio; Kokubo, Takeshi; Daida, Hiroyuki | Not applicable based on title |
| Acute effects of energy drinks in medical students | 2017 | GarcÌa, AndrÈs; Romero, CÈsar; Arroyave, Cristhian; Giraldo, Fabi·n; S·nchez, Leidy; S·nchez, Julio | Not applicable based on title |
| Intake of green tea inhibited increase of salivary chromogranin A after mental task stress loads | 2014 | Yoto, Ai; Murao, Sato; Nakamura, Yoriyuki; Yokogoshi, Hidehiko | Not applicable based on title |
| Pre-germinated brown rice could enhance maternal mental health and immunity during lactation | 2007 | Sakamoto, Shigeko; Hayashi, Takashi; Hayashi, Keiko; Murai, Fumie; Hori, Miyo; Kimoto, Koichi; Murakami, Kazuo | Not applicable based on title |
| Disordered eating behaviour is associated with blunted cortisol and cardiovascular reactions to acute psychological stress | 2012 | Ginty, Annie T; Phillips, Anna C; Higgs, Suzanne; Heaney, Jennifer L J; Carroll, Douglas | Not applicable based on title |
| Pilot Study of a Telehealth-Delivered Medication-Augmented Exposure Therapy Protocol for PTSD | 2017 | Olden, Megan; Wyka, Katarzyna; Cukor, Judith; Peskin, Melissa; Altemus, Margaret; Lee, Francis S; Finkelstein-Fox, Lucy; Rabinowitz, Terry; Difede, JoAnn | Not applicable based on title |
| Altering meal timing to improve cognitive performance during simulated nightshifts | 2019 | Gupta, Charlotte C; Centofanti, Stephanie; Dorrian, Jillian; Coates, Alison; Stepien, Jacqueline M; Kennaway, David; Wittert, Gary; Heilbronn, Leonie; Catcheside, Peter; Noakes, Manny; Coro, Daniel; Chandrakumar, Dilushi; Banks, Siobhan | Not applicable based on title |
| Effects of strategic early-morning caffeine gum administration on association between salivary alpha-amylase and neurobehavioural performance during 50/ h of sleep deprivation | 2019 | Pajcin, Maja; White, Jason M; Banks, Siobhan; Dorrian, Jill; Paech, Gemma M; Grant, Crystal L; Johnson, Kayla; Tooley, Katie; Aidman, Eugene; Fidock, Justin; Kamimori, Gary H; Della Vedova, Chris B | Not applicable based on title |
| The effect of intermittent fasting during Ramadan on sleep, sleepiness, cognitive function, and circadian rhythm | 2017 | Qasrawi, Shaden O; Pandi-Perumal, Seithikurippu R; BaHammam, Ahmed S | Not applicable based on title |
| Emotion regulation as a predictor of the endocrine, autonomic, affective, and symptomatic stress response and recovery | 2018 | Krkovic, Katarina; Clamor, Annika; Lincoln, Tania M | Not applicable based on title |
| Psychoendocrine and physical performance responses in male Tunisian rugby players during an international competitive season | 2008 | Elloumi, Mohamed; Ben Ounis, Omar; Tabka, Zouhair; Van Praagh, Emmanuel; Michaux, Odile; Lac, GÈrard | Not applicable based on title |
| The digit ratio (2D:4D) relationship with testosterone is moderated by physical training: Evidence of prenatal organizational influences on activational patterns of adult testosterone in physically-active women | 2019 | Crewther, Blair T; Cook, Christian J | Not applicable based on title |
| Effects of oral contraceptive use on female sexual salivary hormones and indirect markers of muscle damage following eccentric cycling in women | 2019 | Mackay, Karen; Gonz·lez, Cristopher; Zbinden-Foncea, Hermann; PeÒailillo, Luis | Not applicable based on title |
| Effect of dietary intake on immune function in athletes | 2005 | Venkatraman, Jaya T; Pendergast, David R | Not applicable based on title |
| Leveraging the experience of stressors: the role of adaptive systematic self-reflection | 2020 | Crane, Monique F; Kangas, Maria; Karin, Eyal; Searle, Ben; Chen, Diana | Not applicable based on title |
| Effect of Supplementation with Saccharomyces Boulardii on Academic Examination Performance and Related Stress in Healthy Medical Students: A Randomized, Double-Blind, Placebo-Controlled Trial | 2020 | Karbownik, MichaB Seweryn; KrczyDska, Joanna; Kwarta, Paulina; Cybula, Magdalena; Wiktorowska-Owczarek, Anna; Kowalczyk, Edward; Pietras, Tadeusz; Szemraj, Janusz | Not applicable based on title |
| Examining the Impact of a University-driven Exercise Programming Event on End-of-semester Stress in Students | 2017 | Koschel, Tessa L; Young, John C; Navalta, James W | Not applicable based on title |
| Salivary alpha amylase and salivary cortisol response to fluid consumption in exercising athletes | 2015 | Backes, T P; Horvath, P J; Kazial, K A | Not applicable based on title |
| Worldview under stress: preliminary findings on cardiovascular and cortisol stress responses predicted by secularity, religiosity, spirituality, and existential search | 2020 | Schnell, Tatjana; Fuchs, Dietmar; Hefti, RenÈ | Not applicable based on title |
| The effect of short-term and long-term coronavirus quarantine on physical performance and injury incidence in high-level soccer | 2020 | Guerrero-CalderÛn, Berni | Not applicable based on title |
| A randomized controlled trial of high dose ascorbic acid for reduction of blood pressure, cortisol, and subjective responses to psychological stress | 2002 | Brody, Stuart; Preut, Ragnar; Schommer, Kerstin; Sch¸rmeyer, Thomas H | Not applicable based on title |
| The influence of vitamin C on the interaction between acute mental stress and endothelial function | 2017 | Plotnick, Meghan D; D'Urzo, Katrina A; Gurd, Brendon J; Pyke, Kyra E | Not applicable based on title |
| Vitamin C supplementation and salivary immune function following exercise-heat stress | 2008 | Carrillo, Andres E; Murphy, RenÈ J L; Cheung, Stephen S | Not applicable based on title |
| Caffeine and theanine exert opposite effects on attention under emotional arousal | 2017 | Giles, Grace E; Mahoney, Caroline R; BrunyÈ, Tad T; Taylor, Holly A; Kanarek, Robin B | Not applicable based on title |
| The effect of blue-enriched white light on cognitive performances and sleepiness of night-shift workers: A field study | 2017 | Motamedzadeh, Majid; Golmohammadi, Rostam; Kazemi, Reza; Heidarimoghadam, Rashid | Not applicable based on title |
| Brief training of psychoneuroendocrinoimmunology-based meditation (PNEIMED) reduces stress symptom ratings and improves control on salivary cortisol secretion under basal and stimulated conditions | 2014 | Bottaccioli, Francesco; Carosella, Antonia; Cardone, Raffaella; Mambelli, Monica; Cemin, Marisa; D'Errico, Marcello M; Ponzio, Elisa; Bottaccioli, Anna Giulia; Minelli, Andrea | Not applicable based on title |
| Exercise and the cortisol awakening response: A systematic review | 2017 | Anderson, Travis; Wideman, Laurie | Not applicable based on title |
| Stress effects on mood, HPA axis, and autonomic response: comparison of three psychosocial stress paradigms | 2014 | Giles, Grace E; Mahoney, Caroline R; BrunyÈ, Tad T; Taylor, Holly A; Kanarek, Robin B | Not applicable based on title |
| The imaging Maastricht Acute Stress Test (iMAST): a neuroimaging compatible psychophysiological stressor | 2013 | Quaedflieg, Conny W E M; Meyer, T; Smeets, T | Not applicable based on title |
| Genetic and environmental modulation of neurotrophic and anabolic stress response: Counterbalancing forces | 2015 | Taylor, Marcus K; Carpenter, Jennifer; Stone, Michael; Hernandez, Lisa M; Rauh, Mitchell J; Laurent, Heidemarie K; Granger, Douglas A | Not applicable based on title |
| The impact of attentional training on the salivary cortisol and alpha amylase response to psychosocial stress: importance of attentional control | 2014 | Pilgrim, Kamala; Ellenbogen, Mark A; Paquin, Karine | Not applicable based on title |
| Salivary alpha-amylase as a measure of endogenous adrenergic activity | 1996 | Chatterton, R T; Vogelsong, K M; Lu, Y C; Ellman, A B; Hudgens, G A | Not applicable based on title |
| Psychoneuroendocrine effects of cognitive-behavioral stress management in a naturalistic setting--a randomized controlled trial | 2006 | Gaab, J; Sonderegger, L; Scherrer, S; Ehlert, U | Not applicable based on title |
| The burden of conscientiousness? Examining brain activation and cortisol response during social evaluative stress | 2017 | Dahm, Anne-Sophie; Schmierer, Phˆbe; Veer, Ilya M; Streit, Fabian; Gˆrgen, Anna; Kruschwitz, Johann; W¸st, Stefan; Kirsch, Peter; Walter, Henrik; Erk, Susanne | Not applicable based on title |
| Yoga for veterans with PTSD: Cognitive functioning, mental health, and salivary cortisol | 2020 | Zaccari, Belle; Callahan, Megan L; Storzbach, Daniel; McFarlane, Nancy; Hudson, Rebekah; Loftis, Jennifer M | Not applicable based on title |
| Training Reduces Stress in Human-Socialised Wolves to the Same Degree as in Dogs | 2016 | Vasconcellos, AngÈlica da Silva; Vir·nyi, ZsÛfia; Range, Friederike; Ades, CÈsar; Scheidegger, Jˆrdis Kristin; Mˆstl, Erich; Kotrschal, Kurt | Not applicable based on title |
| The relationship between serum and salivary cortisol levels in response to different intensities of exercise | 2011 | VanBruggen, Mitch D; Hackney, Anthony C; McMurray, Robert G; Ondrak, Kristin S | Not applicable based on title |
| Anxiety and depressive symptoms, and stress biomarkers in pregnant women after in vitro fertilization: a prospective cohort study | 2018 | GarcÌa-Blanco, Ana; Diago, Vicente; Herv·s, David; Ghosn, Farah; Vento, M·ximo; Ch·fer-Peric·s, Consuelo | Not applicable based on title |
| Women's mental health nursing: depression, anxiety and stress during pregnancy | 2010 | Parcells, D A | Not applicable based on title |
| The plasmatic and salivary levels of IL-1≤, IL-18 and IL-6 are associated to emotional difference during stress in young male | 2018 | La Fratta, I; Tatangelo, R; Campagna, G; Rizzuto, A; Franceschelli, S; Ferrone, A; Patruno, A; Speranza, L; De Lutiis, M A; Felaco, M; Grilli, A; Pesce, M | Not applicable based on title |
| Sex-specific association between functional neuropeptide S receptor gene (NPSR1) variants and cortisol and central stress responses | 2017 | Streit, Fabian; Akdeniz, Ceren; Haddad, Leila; Kumsta, Robert; Entringer, Sonja; Frank, Josef; Yim, Ilona S; Z‰nkert, Sandra; Witt, Stephanie H; Kirsch, Peter; Rietschel, Marcella; W¸st, Stefan | Not applicable based on title |
| Physiological responses during exposure to carbon dioxide and bioeffluents at levels typically occurring indoors | 2017 | Zhang, X; Wargocki, P; Lian, Z | Not applicable based on title |
| Acute stress and working memory: The role of sex and cognitive stress appraisal | 2016 | Zandara, M; Garcia-Lluch, M; Pulopulos, M M; Hidalgo, V; Villada, C; Salvador, A | Not applicable based on title |
| Psychoneuroendocrinoimmunology-based meditation (PNEIMED) training reduces salivary cortisol under basal and stressful conditions in healthy university students: Results of a randomized controlled study | 2005 | Bottaccioli, Anna Giulia; Bottaccioli, Francesco; Carosella, Antonia; Cofini, Vincenza; Muzi, Paola; Bologna, Mauro | Not applicable based on title |
| Anxiety and stress in live disaster exercises | 2019 | Farra, Sharon L; Smith, Sherrill J | Not applicable based on title |
| The impact of long-term confinement and exercise on central and peripheral stress markers | 2015 | Jacubowski, A; Abeln, V; Vogt, T; Yi, B; ChoukËr, A; Fomina, E; Str¸der, H K; Schneider, S | Not applicable based on title |
| The effects of cannabidiol on persecutory ideation and anxiety in a high trait paranoid group | 2018 | Hundal, Harneet; Lister, Rachel; Evans, Nicole; Antley, Angus; Englund, Amir; Murray, Robin M; Freeman, Daniel; Morrison, Paul D | Not applicable based on title |
| The effect of 20 minutes scuba diving on cognitive function of professional scuba divers | 2016 | Pourhashemi, Seyedeh Faezeh; Sahraei, Hedayat; Meftahi, Gholam Hossein; Hatef, Boshra; Gholipour, Bahareh | Not applicable based on title |
| The effects of acute work stress and appraisal on psychobiological stress responses in a group office environment | 2020 | Kerr, Jasmine I; Naegelin, Mara; Weibel, Raphael P; Ferrario, Andrea; La Marca, Roberto; von Wangenheim, Florian; Hoelscher, Christoph; Schinazi, Victor R | Not applicable based on title |
| Stress response and communication in surgeons undergoing training in endoscopic management of major vessel hemorrhage: a mixed methods study | 2017 | Jukes, Alistair K; Mascarenhas, Annika; Murphy, Jae; Stepan, Lia; MuÒoz, Tamara N; Callejas, Claudio A; Valentine, Rowan; Wormald, P J; Psaltis, Alkis J | Not applicable based on title |
| Maternal cortisol during pregnancy is related to infant cardiac vagal control | 2015 | Rash, Joshua A; Campbell, Tavis S; Letourneau, Nicole; Giesbrecht, Gerald F | Not applicable based on title |
| Effects of a 12-week endurance training program on the physiological response to psychosocial stress in men: a randomized controlled trial | 2014 | Klaperski, Sandra; von Dawans, Bernadette; Heinrichs, Markus; Fuchs, Reinhard | Not applicable based on title |
| The stressed prefrontal cortex and goal-directed behaviour: acute psychosocial stress impairs the flexible implementation of task goals | 2012 | Plessow, Franziska; Kiesel, Andrea; Kirschbaum, Clemens | Not applicable based on title |
| Salivary cortisol as a non-invasive window on the brain | 2020 | Clow, Angela; Smyth, Nina | Not applicable based on title |
| Problematic Internet use, excessive alcohol consumption, their comorbidity and cardiovascular and cortisol reactions to acute psychological stress in a student population | 2015 | Bibbey, Adam; Phillips, Anna C; Ginty, Annie T; Carroll, Douglas | Not applicable based on title |
| Neuromuscular, Biochemical, Endocrine, and Mood Responses to Small-Sided Games' Training in Professional Soccer | 2018 | Sparkes, William; Turner, Anthony; Weston, Matthew; Russell, Mark; Johnston, Michael; Kilduff, Liam | Not applicable based on title |
| The neuromuscular, endocrine and mood responses to a single versus double training session day in soccer players | 2020 | Sparkes, W; Turner, A N; Cook, C J; Weston, M; Russell, M; Johnston, M J; Kilduff, L P | Not applicable based on title |
| Decrements in neuromuscular performance and increases in creatine kinase impact training outputs in elite soccer players | 2018 | Malone, Shane; Mendes, Bruno; Hughes, Brian; Roe, Mark; Devenney, Simon; Collins, Kieran; Owen, Adam | Not applicable based on title |
| Self-Paced Team-Sport Match Simulation Results in Reductions in Voluntary Activation and Modifications to Biological, Perceptual, and Performance Measures at Halftime and for up to 96 Hours Postmatch | 2018 | Tofari, Paul J; Kemp, Justin G; Cormack, Stuart J | Not applicable based on title |
| The effect of lower limb occlusion on recovery following sprint exercise in academy rugby players | 2018 | Williams, N; Russell, M; Cook, C J; Kilduff, L P | No acute stressor |
| Training and competition workloads and fatigue responses of elite junior cricket players | 2013 | McNamara, Dean J; Gabbett, Tim J; Naughton, Geraldine; Farhart, Patrick; Chapman, Paul | Not applicable based on title |
| Time-Motion and Biological Responses in Simulated Mixed Martial Arts Sparring Matches | 2016 | Coswig, Victor S; Ramos, Solange de P; Del Vecchio, FabrÌcio B | Not applicable based on title |
| Neuromuscular function, hormonal, and mood responses to a professional rugby union match | 2014 | West, Daniel J; Finn, Charlotte V; Cunningham, Daniel J; Shearer, David A; Jones, Marc R; Harrington, Bradley J; Crewther, Blair T; Cook, Christian J; Kilduff, Liam P | Not applicable based on title |
| Identification of sensitive measures of recovery after external load from football match play | 2017 | Rowell, Amber E; Aughey, Robert J; Hopkins, Will G; Stewart, Andrew M; Cormack, Stuart J | Not applicable based on title |
| Salivary testosterone is related to self-selected training load in elite female athletes | 2013 | Cook, Christian J; Beaven, C Martyn | Not applicable based on title |
| Salivary testosterone responses to a physical and psychological stimulus and subsequent effects on physical performance in healthy adults | 2015 | Crewther, Blair T; Kilduff, Liam P; Finn, Charlie; Scott, Phil; Cook, Christian J | Included |
| Markers of postmatch fatigue in professional Rugby League players | 2011 | McLellan, Christopher P; Lovell, Dale I; Gass, Gregory C | age |
| The impact of neuromuscular electrical stimulation on recovery after intensive, muscle damaging, maximal speed training in professional team sports players | 2015 | Taylor, Tom; West, Daniel J; Howatson, Glyn; Jones, Chris; Bracken, Richard M; Love, Thomas D; Cook, Christian J; Swift, Eamon; Baker, Julien S; Kilduff, Liam P | Not applicable based on title |
| Acute neuromuscular and endocrine responses to two different compound exercises: squat vs deadlift | 2019 | Barnes, Matthew J; Miller, Adam; Reeve, Daniel; Stewart, Robin J C | Not applicable based on title |
| Basal and stress-induced salivary testosterone variation across the menstrual cycle and linkage to motivation and muscle power | 2018 | Cook, C J; Kilduff, L P; Crewther, B T | Not applicable based on title |
| Virtual and real-life ostracism and its impact on a subsequent acute stressor | 2021 | Kothgassner, Oswald D; Goreis, Andreas; Glenk, Lisa M; Kafka, Johanna Xenia; Beutl, Leon; Kryspin-Exner, Ilse; Hlavacs, Helmut; Palme, Rupert; Felnhofer, Anna | Not applicable based on title |
| Effects of Carbohydrate and Glutamine Supplementation on Oral Mucosa Immunity after Strenuous Exercise at High Altitude: A Double-Blind Randomized Trial | 2017 | Caris, Aline Venticinque; Da Silva, Edgar Tavares; Dos Santos, Samile Amorim; Tufik, Sergio; Dos Santos, Ronaldo Vagner Thomatieli | Not applicable based on title |
| Does the level of physical exercise affect physiological and psychological responses to psychosocial stress in women? | 2013 | Klaperski, Sandra; von Dawans, Bernadette; Heinrichs, Markus; Fuchs, Reinhard | Not applicable based on title |
| Physiological restitution after night-call duty in anaesthesiologists: impact on metabolic factors | 2007 | Malmberg, B; Persson, R; Jˆnsson, B A G; Erfurth, E-M; Flisberg, P; Ranklev, E; Orbaek, P | Not applicable based on title |
| Mental practice: effective stress management training for novice surgeons | 2011 | Arora, Sonal; Aggarwal, Rajesh; Moran, Aidan; Sirimanna, Pramudith; Crochet, Patrice; Darzi, Ara; Kneebone, Roger; Sevdalis, Nick | Not applicable based on title |
| Twelve weeks of exercise modality in hypoxia enhances health-related function in obese older Korean men: A randomized controlled trial | 2019 | Park, Hun-Young; Jung, Won-Sang; Kim, Jisu; Lim, Kiwon | Not applicable based on title |
| Therapeutic psychological and biological responses to mindfulness-based art therapy | 2020 | Beerse, Megan E; Van Lith, Theresa; Stanwood, Gregg | Not applicable based on title |
| A Randomized Crossover Trial on Acute Stress-Related Physiological Responses to Mountain Hiking | 2017 | Niedermeier, Martin; Grafetst‰tter, Carina; Hartl, Arnulf; Kopp, Martin | Not applicable based on title |
| Vasopressin needs an audience: neuropeptide elicited stress responses are contingent upon perceived social evaluative threats | 2011 | Shalev, Idan; Israel, Salomon; Uzefovsky, Florina; Gritsenko, Inga; Kaitz, Marsha; Ebstein, Richard P | Not applicable based on title |
| Cortisol and induced cognitive fatigue: effects on memory activation in healthy males | 2013 | Klaassen, Elissa B; de Groot, Renate H M; Evers, Elisabeth A T; Nicolson, Nancy A; Veltman, Dick J; Jolles, Jelle | Not applicable based on title |
| Rooming-in Reduces Salivary Cortisol Level of Newborn | 2018 | De Bernardo, Giuseppe; Riccitelli, Marina; Giordano, Maurizio; Proietti, Fabrizio; Sordino, Desiree; Longini, Mariangela; Buonocore, Giuseppe; Perrone, Serafina | Not applicable based on title |
| Vitamin D Supplementation during Winter: Effects on Stress Resilience in a Randomized Control Trial | 2020 | Hansen, Anita L; Ambroziak, Gina; Thornton, David; Mundt, James C; Kahn, Rachel E; Dahl, Lisbeth; Waage, Leif; Kattenbraker, Daniel; Araujo, Pedro; Murison, Robert; Rypdal, Knut; Grung, Bj¯rn | Not applicable based on title |
| Inflexibly focused under stress: acute psychosocial stress increases shielding of action goals at the expense of reduced cognitive flexibility with increasing time lag to the stressor | 2011 | Plessow, Franziska; Fischer, Rico; Kirschbaum, Clemens; Goschke, Thomas | Not applicable based on title |
| No relationship between the digit ratios (2D:4D) and salivary testosterone change: Study on men under an acute exercise | 2020 | Kowal, Marta; Sorokowski, Piotr; {elazniewicz, Agnieszka; Nowak, Judyta; Orzechowski, Sylwester; {urek, Grzegorz; {urek, Alina; Juszkiewicz, Anna; Wojtycka, Lidia; Sieniu, Wiktoria; Poniatowska, MaBgorzata; Tarnowska, Karolina; Kowalska, Kaja; Drabik, Katarzyna; Aukaszek, Patrycja; Krawczyk, Krzysztof; Stefaniak, Tadeusz; Danek, Natalia | Not applicable based on title |
| Acute stress and anxiety in medical residents on the emergency department duty | 2018 | Gonz·lez-Cabrera, JoaquÌn M; Fern·ndez-Prada, MarÌa; Iribar, ConcepciÛn; Molina-Ruano, Rogelio; Salinero-Bachiller, MarÌa; Peinado, JosÈ M | Not applicable based on title |
| Decision-making styles and physiological correlates of negative stress: is there a relation? | 2008 | Thunholm, Peter | No acute stressor |
| Delayed effects of acute stress on cognitive emotion regulation | 2021 | Langer, Katja; Wolf, Oliver T; Jentsch, Valerie L | Not applicable based on title |
| Acute stress in residents playing different roles during emergency simulations: a preliminary study | 2017 | Dias, Roger Daglius; Scalabrini-Neto, Augusto | Not applicable based on title |
| Sportomics in professional soccer players: metabolomics results during preseason | 2021 | Pintus, Roberta; Bongiovanni, Tindaro; Corbu, Sara; Francavilla, Vincenzo C; DessÃ, Angelica; Noto, Antonio; Corsello, Giovanni; Finco, Gabriele; Fanos, Vassilios; Cesare Marincola, Flaminia | Not applicable based on title |
| Circadian rhythms in exercise performance: implications for hormonal and muscular adaptation | 2011 | Teo, Weipeng; Newton, Michael J; McGuigan, Michael R | Not applicable based on title |
| The impact of sleeping with reduced glycogen stores on immunity and sleep in triathletes | 2016 | Louis, Julien; Marquet, Laurie-Anne; Tiollier, Eve; Bermon, StÈphane; Hausswirth, Christophe; Brisswalter, Jeanick | Not applicable based on title |
| Effect of pre-exercise ingestion of ±-lactalbumin on subsequent endurance exercise performance and mood states | 2019 | Qin, Lu; Sun, Feng-Hua; Huang, Yu; Sheridan, Sinead; Sit, Cindy Hui-Ping; Wong, Stephen Heung-Sang | Not applicable based on title |
| Marathon training and immune function | 2005 | Nieman, David C | Not applicable based on title |
| Hormonal contraceptive use diminishes salivary cortisol response to psychosocial stress and naltrexone in healthy women | 2013 | Roche, Daniel J O; King, Andrea C; Cohoon, Andrew J; Lovallo, William R | Not applicable based on title |
| Physiological stress markers, mental health and objective physical function | 2020 | Lever-van Milligen, Bianca A; Lamers, Femke; Smit, Johannes H; Penninx, Brenda W J H | Not applicable based on title |
| Methods for regulating and monitoring resistance training | 2020 | Helms, Eric R; Kwan, Kedric; Sousa, Colby A; Cronin, John B; Storey, Adam G; Zourdos, Michael C | Not applicable based on title |
| Acute psychosocial stress reduces pain modulation capabilities in healthy men | 2014 | Geva, Nirit; Pruessner, Jens; Defrin, Ruth | Not applicable based on title |
| The long and winding road: Effects of exercise intensity and type upon sustained attention | 2018 | Radel, RÈmi; Tempest, Gavin D; Brisswalter, Jeanick | Not applicable based on title |
| No effect of social exclusion on salivary cortisol secretion in women in a randomized controlled study | 2010 | Zˆller, C‰cilia; Maroof, Patrick; Weik, Ulrike; Deinzer, Renate | Not applicable based on title |
| DNA methylation and psychotherapy response in trauma-exposed men with appetitive aggression | 2021 | Xulu, Khethelo R; Womersley, Jacqueline S; Sommer, Jessica; Hinsberger, Martina; Elbert, Thomas; Weierstall, Roland; Kaminer, Debbie; Malan-M¸ller, Stefanie; Seedat, Soraya; Hemmings, Sian M J | Not applicable based on title |
| Does psychosocial stress impact cognitive reappraisal? behavioral and neural evidence | 2017 | Shermohammed, Maheen; Mehta, Pranjal H; Zhang, Joan; Brandes, Cassandra M; Chang, Luke J; Somerville, Leah H | Not applicable based on title |
| Cortisol and affective responses to exercise | 1998 | Rudolph, D L; McAuley, E | Not applicable based on title |
| Effect of novel recovery garments utilising nanodiamond- and nanoplatinum-coated materials (DPV576-C) on physical and psychological stress in baseball players: A randomised, placebo-controlled trial | 2019 | Choi, Youngju; Makita, Mizuho; Nakamura, Yuki; Yamamoto, Kousaku; Nara, Takaaki; Kawamura, Takashi; Fukuda, Hidehiro; Katano, Hideki; Miyakawa, Shumpei; Maeda, Seiji | Not applicable based on title |
| Stress-Reducing Function of Matcha Green Tea in Animal Experiments and Clinical Trials | 2018 | Unno, Keiko; Furushima, Daisuke; Hamamoto, Shingo; Iguchi, Kazuaki; Yamada, Hiroshi; Morita, Akio; Horie, Hideki; Nakamura, Yoriyuki | Not applicable based on title |
| Mind-body group treatment for women coping with infertility: a pilot study | 2005 | Psaros, Christina; Kagan, Leslee; Shifren, Jan L; Willett, Jessica; Jacquart, Jolene; Alert, Marissa D; Macklin, Eric A; Styer, Aaron K; Denninger, John W; LaRoche, Katie L; Park, Elyse R | Not applicable based on title |
| The Effect of Stress on Repeated Painful Stimuli with and Without Painful Conditioning | 2020 | Hoegh, Morten; Poulsen, Jeppe N; Petrini, Laura; Graven-Nielsen, Thomas | Not applicable based on title |
| Biomarkers of oxidative stress in saliva of sheep: Analytical performance and changes after an experimentally induced stress | 2019 | Rubio, Camila Peres; Contreras-Aguilar, Maria Dolores; Quiles, Alberto; LÛpez-Arjona, Marina; CerÛn, JosÈ Joaquin; MartÌnez-Subiela, Silvia; Hevia, Maria Luisa; Escribano, Dami·n; Tecles, Fernando | Not applicable based on title |
| Dose-Response and Mechanistic Issues in the Resistance Training and Affect Relationship | 2005 | Arent, Shawn M.; Landers, Daniel M.; Matt, Kathleen S.; Etnier, Jennifer L. | Not applicable based on title |
| Effect of one time coffee fragrance inhalation on working memory, mood, and salivary cortisol level in healthy young volunteers: a randomized placebo controlled trial | 2019 | Hawiset, Thaneeya | Not applicable based on title |
| Stress, allostatic load and mental health in Indigenous Australians | 2020 | Ketheesan, S; Rinaudo, M; Berger, M; Wenitong, M; Juster, R P; McEwen, B S; Sarnyai, Z | Not applicable based on title |
| The physiological and psychological effects of combat ration feeding during a 12-day training exercise in the tropics | 2003 | Booth, Christine K; Coad, Ross A; Forbes-Ewan, Christopher H; Thomson, Gary F; Niro, Philip J | Not applicable based on title |
| Didgeridoo health promotion method improves mood, mental stress, and stability of autonomic nervous system | 2019 | Lee, Suni; Yamamoto, Shoko; Kumagai-Takei, Naoko; Sada, Nagisa; Yoshitome, Kei; Nishimura, Yasumitsu; Kojima, Toshihiro; Otsuki, Takemi | Not applicable based on title |
| Neuroendocrine and cardiovascular correlates of positive affect measured by ecological momentary assessment and by questionnaire | 2007 | Steptoe, Andrew; Gibson, E Leigh; Hamer, Mark; Wardle, Jane | Not applicable based on title |
| Compared to self-immersion, mindful attention reduces salivation and automatic food bias | 2017 | Baquedano, Constanza; Vergara, Rodrigo; Lopez, Vladimir; Fabar, Catalina; Cosmelli, Diego; Lutz, Antoine | Not applicable based on title |
| Mindfulness training attenuates the increase in salivary cortisol concentration associated with competition in highly trained wheelchair-basketball players | 2018 | MacDonald, Luke A; Minahan, Clare L | Not applicable based on title |
| Acute responses to opioidergic blockade as a biomarker of hedonic eating among obese women enrolled in a mindfulness-based weight loss intervention trial | 2015 | Mason, Ashley E; Lustig, Robert H; Brown, Rashida R; Acree, Michael; Bacchetti, Peter; Moran, Patricia J; Dallman, Mary; Laraia, Barbara; Adler, Nancy; Hecht, Frederick M; Daubenmier, Jennifer; Epel, Elissa S | Not applicable based on title |
| The effects of mindfulness training on competition-induced anxiety and salivary stress markers in elite Wushu athletes: A pilot study | 2019 | Mehrsafar, Amir Hossien; Strahler, Jana; Gazerani, Parisa; Khabiri, Mohammad; S·nchez, JosÈ Carlos Jaenes; Moosakhani, Alireza; Zadeh, Ali Moghadam | Not applicable based on title |
| Immune nutrition and exercise: Narrative review and practical recommendations | 2019 | Williams, Neil C; Killer, Sophie C; Svendsen, Ida Siobhan; Jones, Arwel Wyn | Not applicable based on title |
| The impact of public transport on the health of work commuters: a systematic review | 2020 | Norgate, Sarah H; Cooper-Ryan, A M; Lavin, S; Stonier, C; Cooper, C L | Not applicable based on title |
| Non-pharmacological interventions during childbirth for pain relief, anxiety, and neuroendocrine stress parameters: A randomized controlled trial | 2018 | Henrique, Angelita JosÈ; Gabrielloni, Maria Cristina; Rodney, Patricia; Barbieri, M·rcia | Not applicable based on title |
| Dihydrotestosterone (DHT) rapidly increase after maximal aerobic exercise in healthy males: the lowering effect of phosphodiesterase's type 5 inhibitors on DHT response to exercise-related stress | 2021 | SgrÚ, P; Minganti, C; Lista, M; Antinozzi, C; Cappa, M; Pitsiladis, Y; Pigozzi, F; Di Luigi, L | No salivary markers |
| Effects of exercise intensity on salivary antimicrobial proteins and markers of stress in active men | 2008 | Allgrove, Judith E; Gomes, Elisa; Hough, John; Gleeson, Michael | Not applicable based on title |
| An update: salivary hormones and physical exercise | 2011 | Gatti, R; De Palo, E F | Not applicable based on title |
| Acute physical exercise promotes the consolidation of emotional material | 2020 | Jentsch, Valerie L; Wolf, Oliver T | Not applicable based on title |
| Immediate stress reduction effects of yoga during pregnancy: One group pre-post test | 2016 | Kusaka, Momoko; Matsuzaki, Masayo; Shiraishi, Mie; Haruna, Megumi | Not applicable based on title |
| Relationship between resilience and stress: Perceived stress, stressful life events, HPA axis response during a stressful task and hair cortisol | 2019 | GarcÌa-LeÛn, MarÌa ¡ngeles; PÈrez-M·rmol, JosÈ Manuel; Gonzalez-PÈrez, Raquel; GarcÌa-RÌos, MarÌa Del Carmen; Peralta-RamÌrez, MarÌa Isabel | Not applicable based on title |
| Monitoring Fatigue During the In-Season Competitive Phase in Elite Soccer Players | 2015 | Thorpe, Robin T; Strudwick, Anthony J; Buchheit, Martin; Atkinson, Greg; Drust, Barry; Gregson, Warren | Not applicable based on title |
| Neuromuscular, endocrine, and perceptual fatigue responses during different length between-match microcycles in professional rugby league players | 2010 | McLean, Blake D; Coutts, Aaron J; Kelly, Vince; McGuigan, Michael R; Cormack, Stuart J | Included |
| Exploring biobehavioral outcomes in mothers of preterm infants | 2011 | Howland, Lois C; Pickler, Rita H; McCain, Nancy L; Glaser, Dale; Lewis, Mary | Not applicable based on title |
| Beneficial effects of yoga stretching on salivary stress hormones and parasympathetic nerve activity | 2020 | Eda, Nobuhiko; Ito, Hironaga; Akama, Takao | Not applicable based on title |
| Salivary and serum cortisol levels during recovery from intense exercise and prolonged, moderate exercise | 2015 | Powell, J; DiLeo, T; Roberge, R; Coca, A; Kim, J-H | Not applicable based on title |
| Feasibility of a relaxation guided imagery intervention to reduce maternal stress in the NICU | 2017 | Howland, Lois C; Jallo, Nancy; Connelly, Cynthia D; Pickler, Rita H | Not applicable based on title |
| Psychophysiological responses to overloading and tapering phases in elite young soccer players | 2014 | Freitas, Camila G; Aoki, Marcelo S; Franciscon, Clovis A; Arruda, Ademir F S; Carling, Christopher; Moreira, Alexandre | Not applicable based on title |
| Salivary hormone response to 12-week block-periodized training in naval special warfare operators | 2015 | Oliver, Jonathan M; Abt, John P; Sell, Timothy C; Beals, Kim; Wood, Dallas E; Lephart, Scott M | Chronic/Prolonged stressor |
| The effects of mouthpiece use on cortisol levels during an intense bout of resistance exercise | 2011 | Garner, Dena P; Dudgeon, Wesley D; McDivitt, Erica J | Not applicable based on title |
| Effect of whey protein hydrolysate on performance and recovery of top-class orienteering runners | 2015 | Hansen, Mette; Bangsbo, Jens; Jensen, J¯rgen; Bibby, Bo Martin; Madsen, Klavs | Not applicable based on title |
| A 2 Week Cross-over Intervention with a Low Carbohydrate, High Fat Diet Compared to a High Carbohydrate Diet Attenuates Exercise-Induced Cortisol Response, but Not the Reduction of Exercise Capacity, in Recreational Athletes | 2021 | Terink, Rieneke; Witkamp, Renger F; Hopman, Maria T E; Siebelink, Els; Savelkoul, Huub F J; Mensink, Marco | Not applicable based on title |
| The Effects of a Single Whole-Body Cryotherapy Exposure on Physiological, Performance, and Perceptual Responses of Professional Academy Soccer Players After Repeated Sprint Exercise | 2017 | Russell, Mark; Birch, Jack; Love, Thomas; Cook, Christian J; Bracken, Richard M; Taylor, Tom; Swift, Eamon; Cockburn, Emma; Finn, Charlie; Cunningham, Daniel; Wilson, Laura; Kilduff, Liam P | Not applicable based on title |
| Baker's yeast beta glucan supplementation increases salivary IgA and decreases cold/flu symptomatic days after intense exercise | 2013 | McFarlin, Brian K; Carpenter, Katie C; Davidson, Tiffany; McFarlin, Meredith A | Not applicable based on title |
| Physiological and Performance Effects of Caffeine Gum Consumed During a Simulated Half-Time by Professional Academy Rugby Union Players | 2020 | Russell, Mark; Reynolds, Nicholas A; Crewther, Blair T; Cook, Christian J; Kilduff, Liam P | Not applicable based on title |
| Effects of vitamin D supplementation on salivary immune responses during Marine Corps basic training | 2019 | Scott, Jonathan M; Kazman, Josh B; Palmer, Jeremy; McClung, James P; Gaffney-Stomberg, Erin; Gasier, Heath G | Not applicable based on title |
| Effects of acute postexercise chocolate milk consumption during intensive judo training on the recovery of salivary hormones, salivary SIgA, mood state, muscle soreness, and judo-related performance | 2015 | Papacosta, Elena; Nassis, George P; Gleeson, Michael | Not applicable based on title |
| Resilience and hypothalamic-pituitary-adrenal axis reactivity under acute stress in young men | 2008 | Mikolajczak, MoÔra; Roy, Emmanuel; Luminet, Olivier; de Timary, Philippe | Not applicable based on title |
| Biomarkers of stress in music interventions: A systematic review | 2021 | Wong, Melanie Mitsui; Tahir, Talha; Wong, Michael Mitsui; Baron, Annilee; Finnerty, Rachael | Not applicable based on title |
| The effects of listening to music on breast milk production by mothers of premature newborns in the neonatal intensive care unit: A randomized controlled study | 2020 | Vari_o­lu, Yeliz; G¸ngˆr Satilmi_, Ilkay | Not applicable based on title |
| Pharmacology of Schisandra chinensis Bail: an overview of Russian research and uses in medicine | 2008 | Panossian, Alexander; Wikman, Georg | Not applicable based on title |
| Going the distance: The diurnal range of cortisol and its association with cognitive and physiological functioning | 2020 | Charles, Susan T; Mogle, Jacqueline; Piazza, Jennifer R; Karlamangla, Arun; Almeida, David M | Not applicable based on title |
| Acute and Chronic Stress in Daily Police Service: A Three-Week N-of-1 Study | 2020 | Giessing, Laura; Oudejans, RaÙul R D; Hutter, Vana; Plessner, Henning; Strahler, Jana; Frenkel, Marie Ottilie | Not applicable based on title |
| Chronic smoking, trait anxiety, and the physiological response to stress | 2016 | Wiggert, Nicole; Wilhelm, Frank H; Nakajima, Motohiro; al'Absi, Mustafa | Not applicable based on title |
| The Role of Anthropogenic Elements in the Environment for Affective States and Cortisol Concentration in Mountain Hiking-A Crossover Trial | 2019 | Niedermeier, Martin; Grafetst‰tter, Carina; Kopp, Martin; Huber, Daniela; Mayr, Michaela; Pichler, Christina; Hartl, Arnulf | Not applicable based on title |
| Changes in awakening cortisol response and midnight salivary cortisol are sensitive markers of strenuous training-induced fatigue | 2008 | Minetto, M A; Lanfranco, F; Tibaudi, A; Baldi, M; Termine, A; Ghigo, E | Not applicable based on title |
| Working memory performance after acute exposure to the cold pressor stress in healthy volunteers | 2009 | Duncko, Roman; Johnson, Linda; Merikangas, Kathleen; Grillon, Christian | Not applicable based on title |
| Trait anxiety and salivary cortisol during free living and military stress | 2008 | Taylor, Marcus K; Reis, Jared P; Sausen, Kenneth P; Padilla, Genieleah A; Markham, Amanda E; Potterat, Eric G; Drummond, Sean P A | Not applicable based on title |
| Seating type and cognitive performance after 3 hours travel by high-speed boat in sea states 2-3 | 2009 | McMorris, Terry; Myers, Stephen; Dobbins, Trevor; Hall, Ben; Dyson, Rosemary | Not applicable based on title |
| Physiological and psychological effects of escape from a sunken submarine on shore and at sea | 2009 | Trousselard, Marion; Cian, Corinne; Barraud, Pierre-Alain; Ferhani, Ouamar; Roux, Alain; Claverie, Damien; Canini, Frederic; Baert, Patrice | Not applicable based on title |
| High-intensity stress elicits robust cortisol increases, and impairs working memory and visuo-spatial declarative memory in Special Forces candidates: A field experiment | 2010 | Taverniers, John; Van Ruysseveldt, Joris; Smeets, Tom; von Grumbkow, Jasper | Not applicable based on title |
| Simultaneous transcutaneous electrical nerve stimulation mitigates simulator sickness symptoms in healthy adults: a crossover study | 2013 | Chu, Hsin; Li, Min-Hui; Huang, Yu-Cheng; Lee, Shih-Yu | Not applicable based on title |
| Stress reactivity and cognitive performance in a simulated firefighting emergency | 2013 | Robinson, Sarita J; Leach, John; Owen-Lynch, P Jane; S¸nram-Lea, Sandra I | Not applicable based on title |
| Medium-term effects of a two-desk sit/stand workstation on cognitive performance and workload for healthy people performing sedentary work: a secondary analysis of a randomised controlled trial | 2019 | Schwartz, Bernhard; Kapellusch, Jay M; Baca, Arnold; Wessner, Barbara | Not applicable based on title |
| Effects of workstation type on mental stress: FNIRS study | 2021 | Alyan, Emad; Saad, Naufal M; Kamel, Nidal | Not applicable based on title |
| Synergistic effects of edible plants with light environment on the emotion and sleep of humans in long-duration isolated environment | 2020 | Zhang, Wenzhu; Liu, Hui; Li, Zhaoming; Liu, Hong | Not applicable based on title |
| Physiological and Cognitive Performance in F-22 Pilots During Day and Night Flying | 2021 | Combs, Elizabeth K; Dahlman, Anna S; Shattuck, Nita L; Heissel, Jennifer A; Whitaker, Lyn R | Not applicable based on title |
| Effects of alpha-lactalbumin on emotional processing in healthy women | 2007 | Scrutton, Helen; Carbonnier, Anne; Cowen, Philip J; Harmer, Catherine J | Not applicable based on title |
| Fluid type influences acute hydration and muscle performance recovery in human subjects | 2019 | Harris, Preston R; Keen, Douglas A; Constantopoulos, Eleni; Weninger, Savanna N; Hines, Eric; Koppinger, Matthew P; Khalpey, Zain I; Konhilas, John P | Not applicable based on title |
| Effect of SunGold Kiwifruit and Vitamin C Consumption on Ameliorating Exercise-Induced Stress Response in Women | 2021 | Ali, Ajmol; Mehta, Sunali; Starck, Carlene; Wong, Marie; O'Brien, Wendy J; Haswell, Cameron; McNabb, Warren; Rutherfurd-Markwick, Kay; Ahmed Nasef, Noha | Not applicable based on title |
| Effect of a mindfulness exercise on stress in veterinary students performing surgery | 2019 | Stevens, Brenda S; Royal, Kenneth D; Ferris, Kelli; Taylor, Abigail; Snyder, Amy M | Not applicable based on title |
| Salivary cortisol in pregnant women suffering from blood and injection phobia | 2011 | Lilliecreutz, Caroline; Theodorsson, Elvar; Sydsjˆ, Gunilla; Josefsson, Ann | Not applicable based on title |
| Monitoring the swimmer's training load: A narrative review of monitoring strategies applied in research | 2020 | Feijen, Stef; Tate, Angela; Kuppens, Kevin; Barry, Lorna A; Struyf, Filip | Not applicable based on title |
| Relationship between Training Load Management and Immunoglobulin A to Avoid Immunosuppression after Soccer Training and Competition: A Theoretical Framework Based on COVID-19 for Athletes' Healthcare | 2021 | Rico-Gonz·lez, Markel; Pino-Ortega, JosÈ; Clemente, Filipe Manuel; Bustamante-Hern·ndez, Naia | Not applicable based on title |
| Updating futsal physiology, immune system, and performance | 2021 | Borges, Leandro; Dermargos, Alexandre; Gorj„o, Renata; Cury-Boaventura, Maria F; Hirabara, Sandro M; Abad, Cesar C; Pithon-Curi, Tania C; Curi, Rui; Barros, Marcelo P; Hatanaka, Elaine | Not applicable based on title |
| Monitoring training load and fatigue in soccer players with physiological markers | 2017 | Djaoui, LÈo; Haddad, Monoem; Chamari, Karim; Dellal, Alexandre | Not applicable based on title |
| Biomarkers of Physiological Responses to Periods of Intensified, Non-Resistance-Based Exercise Training in Well-Trained Male Athletes: A Systematic Review and Meta-Analysis | 2018 | Greenham, Grace; Buckley, Jonathan D; Garrett, Joel; Eston, Roger; Norton, Kevin | Not applicable based on title |
| Hematology, Hormones, Inflammation, and Muscle Damage in Elite and Professional Soccer Players: A Systematic Review with Implications for Exercise | 2021 | Saidi, Karim; Abderrahman, Abderraouf Ben; Hackney, Anthony C; Bideau, Benoit; Zouita, Sghaeir; Granacher, Urs; Zouhal, Hassane | Not applicable based on title |
| Monitoring the Athlete Match Response: Can External Load Variables Predict Post-match Acute and Residual Fatigue in Soccer? A Systematic Review with Meta-analysis | 2019 | Hader, Karim; Rumpf, Michael C; Hertzog, Maxime; Kilduff, Liam P; Girard, Olivier; Silva, Joao R | review |
| Applied sport science of australian football: A systematic review | 2018 | Johnston, Rich D; Black, Georgia M; Harrison, Peter W; Murray, Nick B; Austin, Damien J | Not applicable based on title |
| Stress perception following childhood adversity: Unique associations with adversity type and sex | 2020 | LoPilato, Allison M; Addington, Jean; Bearden, Carrie E; Cadenhead, Kristin S; Cannon, Tyrone D; Cornblatt, Barbara A; Mathalon, Daniel H; McGlashan, Thomas H; Perkins, Diana O; Tsuang, Ming T; Woods, Scott W; Walker, Elaine F | Not applicable based on title |
| The role of glucocorticoid and mineralocorticoid receptor DNA methylation in antenatal depression and infant stress regulation | 2020 | Galbally, Megan; Watson, Stuart J; van IJzendoorn, Marinus; Saffery, Richard; Ryan, Joanne; de Kloet, Edo Ronald; Oberlander, Tim F; Lappas, Martha; Lewis, Andrew J | Not applicable based on title |
| Immunosensor for assessing the welfare of trainee guide dogs | 2021 | Perkins, Hannah; Higgins, Michelle; Marcato, Marinara; Galvin, Paul; Teixeira, Sofia Rodrigues | Not applicable based on title |
| Differential associations between fatigue and psychobiological stress measures in women with depression and women with somatic symptom disorder | 2021 | Doerr, Johanna M; Nater, Urs M; Feneberg, Anja C; Mewes, Ricarda | Not applicable based on title |
| Driving home from the night shift: a bright light intervention study | 2017 | Weisgerber, Denise M; Nikol, Maria; Mistlberger, Ralph E | Not applicable based on title |
| Fetal programming pathway from maternal mental health to infant cortisol functioning: The role of placental 11≤-HSD2 mRNA expression | 2021 | Galbally, Megan; Watson, Stuart J; Lappas, Martha; de Kloet, E Ron; van Rossum, Elisabeth; Wyrwoll, Caitlin; Mark, Peter; Lewis, Andrew J | Not applicable based on title |
| Neuroendocrine and immune markers of maternal stress during pregnancy and infant cognitive development | 2020 | Nazzari, Sarah; Fearon, Pasco; Rice, Frances; Ciceri, Francesca; Molteni, Massimo; Frigerio, Alessandra | Not applicable based on title |
| Couples becoming parents: Trajectories for psychological distress and buffering effects of social support | 2020 | Hughes, Claire; T Devine, Rory; Foley, Sarah; D Ribner, Andrew; Mesman, Judi; Blair, Clancy | Not applicable based on title |
| The SMART Moms Program: A Randomized Trial of the Impact of Stress Management on Perceived Stress and Cortisol in Low-Income Pregnant Women | 2019 | Urizar, Guido G; Yim, Ilona S; Rodriguez, Anthony; Schetter, Christine Dunkel | Not applicable based on title |
| Evaluation of an antenatal acupuncture intervention as an adjunct therapy for antenatal depression (AcuAnteDep): study protocol for a pragmatic randomised controlled trial | 2016 | Ormsby, Simone M; Smith, Caroline A; Dahlen, Hannah G; Hay, Phillipa J; Lind, Joanne M | Not applicable based on title |
| Effects of an early intervention on perceived stress and diurnal cortisol in pregnant women with elevated stress, anxiety, and depressive symptomatology | 2012 | Richter, Judith; Bittner, Antje; Petrowski, Katja; Junge-Hoffmeister, Juliane; Bergmann, Sybille; Joraschky, Peter; Weidner, Kerstin | Not applicable based on title |
| Decreased salivary alpha-amylase levels are associated with performance deficits during sleep loss | 2017 | Pajcin, Maja; Banks, Siobhan; White, Jason M; Dorrian, Jill; Paech, Gemma M; Grant, Crystal; Johnson, Kayla; Tooley, Katie; Fidock, Justin; Kamimori, Gary H; Della Vedova, Chris B | Included |
| Caffeine may disrupt the impact of real-time drowsiness on cognitive performance: a double-blind, placebo-controlled small-sample study | 2021 | Aidman, E; Balin, M; Johnson, K; Jackson, S; Paech, G M; Pajcin, M; Yates, C; Mitchelson, E; Kamimori, G H; Fidock, J; Vedova, C Della; Banks, S | Not applicable based on title |
| Environmental hypoxia favors myoblast differentiation and fast phenotype but blunts activation of protein synthesis after resistance exercise in human skeletal muscle | 2018 | Gnimassou, Olouyomi; Fern·ndez-Verdejo, Rodrigo; Brook, Matthew; Naslain, Damien; Balan, Estelle; Sayda, Mariwan; Cegielski, Jessica; Nielens, Henri; Decottignies, Anabelle; Demoulin, Jean-Baptiste; Smith, Kenneth; Atherton, Philip J; Francaux, Marc; Deldicque, Louise | Not applicable based on title |
| Monitoring fatigue and recovery in rugby league players | 2013 | Twist, Craig; Highton, Jamie | Review |
| Nasal function and dysfunction in exercise | 2016 | Walker, A; Surda, P; Rossiter, M; Little, S | Not applicable based on title |
| The effects of stress on body weight: biological and psychological predictors of change in BMI | 2007 | Roberts, Cliff; Troop, Nicholas; Connan, Frances; Treasure, Janet; Campbell, Iain C | Not applicable based on title |
| Sleep spindle characteristics in adolescents | 2019 | Goldstone, AimÈe; Willoughby, Adrian R; de Zambotti, Massimiliano; Clark, Duncan B; Sullivan, Edith V; Hasler, Brant P; Franzen, Peter L; Prouty, Devin E; Colrain, Ian M; Baker, Fiona C | Not applicable based on title |
| The impact of abbreviated progressive muscle relaxation on salivary cortisol and salivary immunoglobulin A (sIgA) | 2005 | Pawlow, Laura A; Jones, Gary E | Not applicable based on title |
| Anxious attachment style predicts an enhanced cortisol response to group psychosocial stress | 2015 | Smyth, Nina; Thorn, Lisa; Oskis, Andrea; Hucklebridge, Frank; Evans, Phil; Clow, Angela | Not applicable based on title |
| Thinking of attachments reduces noradrenergic stress response | 2015 | Bryant, Richard A; Chan, Lilian | Not applicable based on title |
| Rumination, distraction and mindful self-focus: effects on mood, dysfunctional attitudes and cortisol stress response | 2009 | Kuehner, C; Huffziger, S; Liebsch, K | Not applicable based on title |
| The roles of noradrenergic and glucocorticoid activation in the development of intrusive memories | 2013 | Bryant, Richard A; McGrath, Chloe; Felmingham, Kim L | Not applicable based on title |
| Impaired empathy and increased anger following social exclusion in non-intoxicated opioid users | 2020 | Carlyle, Molly; Rowley, Megan; Stevens, Tobias; Karl, Anke; Morgan, Celia J A | Not applicable based on title |
| Implementing a structured exercise program for persistent concussion symptoms: a pilot study on the effects on salivary brain-derived neurotrophic factor, cognition, static balance, and symptom scores | 2018 | McGeown, Joshua P; Zerpa, Carlos; Lees, Simon; Niccoli, Sarah; Sanzo, Paolo | Not applicable based on title |
| Does training method matter? Evidence for the negative impact of aversive-based methods on companion dog welfare | 2020 | Vieira de Castro, Ana Catarina; Fuchs, Danielle; Morello, Gabriela Munhoz; Pastur, Stefania; de Sousa, Liliana; Olsson, I Anna S | Not applicable based on title |
| Exploring the effect of the apolipoprotein E (APOE) gene on executive function, working memory, and processing speed during the early recovery period following traumatic brain injury | 2016 | Padgett, Christine R; Summers, Mathew J; Vickers, James C; McCormack, Graeme H; Skilbeck, Clive E | Not applicable based on title |
| Comparison of serum and saliva miRNAs for identification and characterization of mTBI in adult mixed martial arts fighters | 2019 | LaRocca, Daria; Barns, Sarah; Hicks, Steven D; Brindle, Andrew; Williams, Jeremy; Uhlig, Richard; Johnson, Paul; Neville, Christopher; Middleton, Frank A | Not applicable based on title |
| Saliva RNA biomarkers predict concussion duration and detect symptom recovery: a comparison with balance and cognitive testing | 2021 | Fedorchak, Gregory; Rangnekar, Aakanksha; Onks, Cayce; Loeffert, Andrea C; Loeffert, Jayson; Olympia, Robert P; DeVita, Samantha; Leddy, John; Haider, Mohammad N; Roberts, Aaron; Rieger, Jessica; Uhlig, Thomas; Monteith, Chuck; Middleton, Frank; Zuckerman, Scott L; Lee, Timothy; Yeates, Keith Owen; Mannix, Rebekah; Hicks, Steven | Not applicable based on title |
| In search of positive mental health: Personality profiles and genetic polymorphisms | 2021 | Nestor, Paul G; Hasler, Victoria Choate; O'Donovan, Keira; Lapp, Hannah E; Boodai, Sara B; Hunter, Richard | Not applicable based on title |
| Immune status, well-being and gut microbiota in military supplemented with synbiotic ice cream and submitted to field training: a randomised clinical trial | 2021 | Valle, Maria C P R; Vieira, Isabel A; Fino, Luciana C; Gallina, Darlila A; Esteves, Andrea M; da Cunha, Diogo T; Cabral, LucÈlia; Benatti, Fabiana B; Marostica Junior, Mario R; Batista, ¬ngela G; Santos, Rosangela; Pastore, Glaucia M; Sartoratto, Adilson; Sivieri, Katia; Tizioto, Polyana C; Coutinho, Luiz L; Antunes, Adriane E C | Not applicable based on title |
| Advanced melatonin onset relative to sleep in women with unmedicated major depressive disorder | 2019 | Coleman, Michelle Y; McGlashan, Elise M; Vidafar, Parisa; Phillips, Andrew J K; Cain, Sean W | Not applicable based on title |
| Estradiol modulates neural and behavioral arousal in women with posttraumatic stress disorder during a fear learning and extinction task | 2020 | Sartin-Tarm, Anneliis; Ross, Marisa C; Privatsky, Anthony A; Cisler, Josh M | Not applicable based on title |
| Effect of laughter yoga on mental symptoms and salivary cortisol levels in first-year nursing students: A randomized controlled trial | 2021 | Ozturk, Fatma Ozlem; Tezel, Ayfer | Not applicable based on title |
| Effect of pre- and postcompetition emotional state on salivary cortisol in top-ranking wrestlers | 2010 | Coelho, Ricardo Weigert; Keller, Birgit; da Silva, Andressa Melina Becker | Not applicable based on title |
| Salivary ±-amylase and cortisol after exercise in menopause: influence of long-term HRT | 2015 | Patacchioli, F R; Ghiciuc, C M; Bernardi, M; Dima-Cozma, L C; Fattorini, L; Squeo, M R; Galoppi, P; Brunelli, R; Ferrante, F; Pasquali, V; Perrone, G | Not applicable based on title |
| Saliva testing as noninvasive way for monitoring exercise-dependent response in teenage elite water polo players: A cohort study | 2021 | Ferlazzo, Nadia; CurrÚ, Monica; Saija, Caterina; Naccari, Francesco; Ientile, Riccardo; Di Mauro, Debora; Trimarchi, Fabio; Caccamo, Daniela | Not applicable based on title |
| Psychological generators of stress-headaches | 2005 | Berry, Juanita Kay Miller; Drummond, Peter D | Not applicable based on title |
| Open and Calm--a randomized controlled trial evaluating a public stress reduction program in Denmark | 2015 | Jensen, Christian G; Lansner, Jon; Petersen, Anders; Vangkilde, Signe A; Ringk¯bing, Signe P; Frokjaer, Vibe G; Adamsen, Dea; Knudsen, Gitte M; Denninger, John W; Hasselbalch, Steen G | Not applicable based on title |
| Cognitive emotion regulation withstands the stress test: An fMRI study on the effect of acute stress on distraction and reappraisal | 2021 | Sandner, Magdalena; Zeier, Peter; Lois, Giannis; Wessa, MichËle | Not applicable based on title |
| Acute stress shapes creative cognition in trait anxiety | 2019 | Duan, Haijun; Wang, Xuewei; Wang, Zijuan; Xue, Wenlong; Kan, Yuecui; Hu, Weiping; Zhang, Fengqing | Not applicable based on title |
| Basic psychological need satisfaction, stress-related appraisals, and dancers' cortisol and anxiety responses | 2011 | Quested, Eleanor; Bosch, Jos A; Burns, Victoria E; Cumming, Jennifer; Ntoumanis, Nikos; Duda, Joan L | Not applicable based on title |
| Proteomic and metabolomic profiling of acute and chronic stress events associated with military exercises | 2022 | McKetney, Justin; Jenkins, Conor C; Minogue, Catie; Mach, Phillip M; Hussey, Erika K; Glaros, Trevor G; Coon, Joshua; Dhummakupt, Elizabeth S | Included |
| Neonatal procedural pain and preterm infant cortisol response to novelty at 8 months | 2004 | Grunau, Ruth E; Weinberg, Joanne; Whitfield, Michael F | Not applicable based on title |
| Perceived chronic stress influences the effect of acute stress on cognitive flexibility | 2021 | Knauft, Katherine; Waldron, Alexander; Mathur, Mishali; Kalia, Vrinda | Not applicable based on title |
| A short Mindfulness retreat can improve biological markers of stress and inflammation | 2022 | Gardi, Concetta; Fazia, Teresa; Stringa, Blerta; Giommi, Fabio | Not applicable based on title |
| Effects of d-amphetamine upon psychosocial stress responses | 2016 | Childs, Emma; Bershad, Anya K; de Wit, Harriet | Not applicable based on title |
| Investigating individual stress reactivity: High hair cortisol predicts lower acute stress responses | 2020 | Sandner, Magdalena; Lois, Giannis; Streit, Fabian; Zeier, Peter; Kirsch, Peter; W¸st, Stefan; Wessa, MichËle | Not applicable based on title |
| Effects of exercise and human contact on animal welfare in a dog shelter | 2011 | Menor-Campos, D J; Molleda-Carbonell, J M; LÛpez-RodrÌguez, R | Not applicable based on title |
| Contagious yawning, social cognition, and arousal: an investigation of the processes underlying shelter dogs' responses to human yawns | 2014 | Buttner, Alicia Phillips; Strasser, Rosemary | Not applicable based on title |
| Job interview training targeting nonverbal communication using an android robot for individuals with autism spectrum disorder | 2019 | Kumazaki, Hirokazu; Muramatsu, Taro; Yoshikawa, Yuichiro; Corbett, Blythe A; Matsumoto, Yoshio; Higashida, Haruhiro; Yuhi, Teruko; Ishiguro, Hiroshi; Mimura, Masaru; Kikuchi, Mitsuru | Not applicable based on title |
| Assisted physical exercise and stress in preterm neonates | 2018 | Shaw, Subhash Chandra; Sankar, M Jeeva; Thukral, Anu; Agarwal, Ramesh; Deorari, Ashok K; Paul, Vinod K | Not applicable based on title |
| Exercise-induced responses in salivary testosterone, cortisol, and their ratios in men: a meta-analysis | 2015 | Hayes, Lawrence D; Grace, Fergal M; Baker, Julien S; Sculthorpe, Nicholas | Not applicable based on title |
| Emotional memory in pregnant women at risk for postpartum depression | 2015 | Williams, Marissa E; Becker, Suzanna; McKinnon, Margaret C; Wong, Queenie; Cudney, Lauren E; Steiner, Meir; Frey, Benicio N | Not applicable based on title |
| Sleep quality is associated with vasopressin methylation in pregnant and postpartum women with a history of psychosocial stress | 2019 | Solomonova, E; Lee, Y E A; Robins, S; King, L; Feeley, N; Gold, I; Hayton, B; Libman, E; Nagy, C; Turecki, G; Zelkowitz, P | Not applicable based on title |
| Early-life stress and recurrent psychological distress over the lifecourse predict divergent cortisol reactivity patterns in adulthood | 2012 | Goldman-Mellor, Sidra; Hamer, Mark; Steptoe, Andrew | Not applicable based on title |
| Chronic Stress in Cognitive Processes: Cortisol Dynamic Range of Secretion Is Associated with Perception of Unsafety Environment in a Venezuelan Population | 2005 | Ayala-Grosso, Carlos; Torrico, F·tima; Ledezma-Ruiz, Margot; Busolo-Pons, Maria | Not applicable based on title |
| The effect of pilates training on hormonal and psychophysical function in older women | 2022 | Farzane, Arezu; Koushkie Jahromi, Maryam | Not applicable based on title |
| Salivary IgA response and upper respiratory tract infection symptoms during a 21-week competitive season in young soccer players | 2014 | Moreira, Alexandre; Mortatti, Arnaldo L; Arruda, Ademir F S; Freitas, Camila G; de Arruda, Miguel; Aoki, Marcelo S | Not applicable based on title |
| Salivary adiponectin levels are associated with training intensity but not with bone mass or reproductive function in elite Rhythmic Gymnasts | 2014 | Roupas, Nikolaos D; MaÔmoun, Laurent; Mamali, Irene; Coste, Olivier; Tsouka, Alexandra; Mahadea, Krishna Kunal; Mura, Thibault; Philibert, Pascal; Gaspari, Laura; Mariano-Goulart, Denis; Leglise, Michel; Sultan, Charles; Georgopoulos, Neoklis A | Not applicable based on title |
| Effect of menstrual cycle phase on exercise performance of high-altitude native women at 3600 m | 2002 | Brutsaert, Tom D; Spielvogel, Hilde; Caceres, Esperanza; Araoz, Mauricio; Chatterton, Robert T; Vitzthum, Virginia J | Not applicable based on title |
| The impact of cortisol reactivity to acute stress on memory: sex differences in middle-aged people | 2011 | Almela, Mercedes; Hidalgo, Vanesa; Villada, Carolina; EspÌn, Laura; GÛmez-Amor, Jes˙s; Salvador, Alicia | Not applicable based on title |
| Studying the perceptive and cognitive function under the stress of match in female futsal players | 2017 | Sepahvand, Hossein; Pirzad Jahromi, Gila; Sahraei, Hedayat; Meftahi, Gholam Hossein | Not applicable based on title |
| Epigenome-wide association study of posttraumatic stress disorder identifies novel loci in US military veterans | 2022 | Montalvo-Ortiz, Janitza L; Gelernter, Joel; Cheng, Zhongshan; Girgenti, Matthew J; Xu, Ke; Zhang, Xinyu; Gopalan, Shyamalika; Zhou, Hang; Duman, Ronald S; Southwick, Steven M; Krystal, John H; Traumatic Stress Brain Research Study Group; Pietrzak, Robert H | Not applicable based on title |
| Effects of milk-based phospholipids on cognitive performance and subjective responses to psychosocial stress: A randomized, double-blind, placebo-controlled trial in high-perfectionist men | 2019 | Boyle, Neil B; Dye, Louise; ArkbÂge, Karin; Thorell, Lars; Frederiksen, Pernille; Croden, Fiona; Lawton, Clare | Not applicable based on title |
| Investigating differential effects of socio-emotional and mindfulness-based online interventions on mental health, resilience and social capacities during the COVID-19 pandemic: The study protocol | 2021 | Godara, Malvika; Silveira, Sarita; Matth‰us, Hannah; Heim, Christine; Voelkle, Manuel; Hecht, Martin; Binder, Elisabeth B; Singer, Tania | Not applicable based on title |
| Sleep quality in young adult informal caregivers: understanding psychological and biological processes | 2021 | Hoyt, Michael A; Mazza, Mary Carol; Ahmad, Zeba; Darabos, Katie; Applebaum, Allison J | Not applicable based on title |
| Psychological well-being and ill-being: do they have distinct or mirrored biological correlates? | 2005 | Ryff, Carol D; Dienberg Love, Gayle; Urry, Heather L; Muller, Daniel; Rosenkranz, Melissa A; Friedman, Elliot M; Davidson, Richard J; Singer, Burton | Not applicable based on title |
| Crime and violence: Desensitization in victims to watching criminal events | 2019 | Di Tella, Rafael; Freira, LucÌa; G·lvez, Ramiro H.; Schargrodsky, Ernesto; Shalom, Diego; Sigman, Mariano | Not applicable based on title |
| Effects of cannabidiol on symptoms induced by the recall of traumatic events in patients with posttraumatic stress disorder | 2022 | Bolsoni, LÌvia Maria; Crippa, JosÈ Alexandre S; Hallak, Jaime Eduardo CecÌlio; Guimar„es, Francisco Silveira; Zuardi, Antonio Waldo | Not applicable based on title |
| A longitudinal investigation of bidirectional and time-dependent interrelationships between testosterone and training motivation in an elite rugby environment | 2020 | Crewther, Blair T; Hecht, Martin; Potts, Neill; Kilduff, Liam P; Drawer, Scott; Marshall, Elizabeth; Cook, Christian J | Not applicable based on title |
| Dawn simulation light: a potential cardiac events protector | 2015 | Viola, Antoine U; Gabel, Virginie; Chellappa, Sarah L; Schmidt, Christina; Hommes, Vanja; Tobaldini, Eleonora; Montano, Nicola; Cajochen, Christian | Not applicable based on title |
| Impact of maternal trauma-related psychopathology and life stress on HPA axis stress response | 2022 | Ramdas, Dawn L; Sbrilli, Marissa D; Laurent, Heidemarie K | Not applicable based on title |
| Decrease in Salivary Serotonin in Response to Probiotic Supplementation With Saccharomyces boulardii in Healthy Volunteers Under Psychological Stress: Secondary Analysis of a Randomized, Double-Blind, Placebo-Controlled Trial | 2005 | Karbownik, MichaB Seweryn; KrczyDska, Joanna; Wiktorowska-Owczarek, Anna; Kwarta, Paulina; Cybula, Magdalena; Stilinovi, Nebojaa; Pietras, Tadeusz; Kowalczyk, Edward | Not applicable based on title |
| Lower estradiol predicts increased reinstatement of fear in women | 2021 | Felmingham, Kim L; Caruana, Julia M; Miller, Lisa N; Ney, Luke J; Zuj, Daniel V; Hsu, Chia Ming K; Nicholson, Emma; To, Annie; Bryant, Richard A | Not applicable based on title |
| The effects of high intensity short rest resistance exercise on muscle damage markers in men and women | 2014 | Heavens, Kristen R; Szivak, Tunde K; Hooper, David R; Dunn-Lewis, Courtenay; Comstock, Brett A; Flanagan, Shawn D; Looney, David P; Kupchak, Brian R; Maresh, Carl M; Volek, Jeff S; Kraemer, William J | No salivary markers |
| Estradiol and progesterone as resilience markers? - Findings from the Swiss Perimenopause Study | 2021 | S¸ss, Hannah; Willi, Jasmine; Grub, Jessica; Ehlert, Ulrike | Not applicable based on title |
| Impact of a simulated stress training program on the tactical shooting performance of SWAT trainees | 2018 | Liu, Yuxin; Mao, Lida; Zhao, Yunan; Huang, Yufang | Not applicable based on title |
| Oxytocin, cortisol, and cognitive control during acute and naturalistic stress | 2021 | Young Kuchenbecker, Shari; Pressman, Sarah D; Celniker, Jared; Grewen, Karen M; Sumida, Kenneth D; Jonathan, Naveen; Everett, Brendan; Slavich, George M | Not applicable based on title |
| EEG Mental Stress Assessment Using Hybrid Multi-Domain Feature Sets of Functional Connectivity Network and Time-Frequency Features | 2021 | Hag, Ala; Handayani, Dini; Pillai, Thulasyammal; Mantoro, Teddy; Kit, Mun Hou; Al-Shargie, Fares | Not applicable based on title |
| Competitive anxiety or Coronavirus anxiety? The psychophysiological responses of professional football players after returning to competition during the COVID-19 pandemic | 2021 | Mehrsafar, Amir Hossien; Moghadam Zadeh, Ali; Jaenes S·nchez, JosÈ Carlos; Gazerani, Parisa | Not applicable based on title |
| Exercise time and intensity: how much is too much? | 2020 | Gottschall, Jinger S; Davis, Joshua J; Hastings, Bryce; Porter, Heather J | Not applicable based on title |
| Group hypnotherapy versus group relaxation for smoking cessation: an RCT study protocol | 2012 | Dickson-Spillmann, Maria; Kraemer, Thomas; Rust, Kristina; Schaub, Michael | Not applicable based on title |
| Sensitivity to stress among the offspring of parents with bipolar disorder: a study of daytime cortisol levels | 2011 | Ostiguy, C S; Ellenbogen, M A; Walker, C-D; Walker, E F; Hodgins, S | Not applicable based on title |
| Effects of an evidence-based parenting program on biobehavioral stress among at-risk mothers for child maltreatment: A pilot study | 2018 | Tiwari, Ashwini; Self-Brown, Shannon; Lai, Betty S; McCarty, Colleen; Carruth, Laura | Not applicable based on title |
| Acculturative stress, mental health symptoms, and the role of salivary inflammatory markers among a Latino sample | 2018 | Maldonado, Adriana; Preciado, Andrea; Buchanan, Melissa; Pulvers, Kim; Romero, Devan; D'Anna-Hernandez, Kimberly | Not applicable based on title |
| Cardiovascular and cortisol responses to a psychological stressor during pregnancy | 2007 | De Weerth, Carolina; Wied, Gispen-De; Jansen, Lucres M C; Buitelaar, Jan K | Not applicable based on title |
| Pregnant women's cortisol is elevated with anxiety and depression - but only when comorbid | 2008 | Evans, Lynn M; Myers, Michael M; Monk, Catherine | Not applicable based on title |
| Social-evaluative threat, cognitive load, and the cortisol and cardiovascular stress response | 2018 | Woody, Alex; Hooker, Emily D; Zoccola, Peggy M; Dickerson, Sally S | Not applicable based on title |
| Effects of Coping-Related Traits and Psychophysiological Stress Responses on Police Recruits' Shooting Behavior in Reality-Based Scenarios | 2019 | Giessing, Laura; Frenkel, Marie Ottilie; Zinner, Christoph; Rummel, Jan; Nieuwenhuys, Arne; Kasperk, Christian; Brune, Maik; Engel, Florian Azad; Plessner, Henning | Not applicable based on title |
| Heidelberg Risk Sport-Specific Stress Test: A Paradigm to Investigate the Risk Sport-Specific Psycho-Physiological Arousal | 2019 | Frenkel, Marie Ottilie; Laborde, Sylvain; Rummel, Jan; Giessing, Laura; Kasperk, Christian; Plessner, Henning; Heck, Robin-Bastian; Strahler, Jana | Not applicable based on title |
| Effects of Probiotic (Bacillus subtilis DE111) Supplementation on Immune Function, Hormonal Status, and Physical Performance in Division I Baseball Players | 2018 | Townsend, Jeremy R; Bender, David; Vantrease, William C; Sapp, Philip A; Toy, Ann M; Woods, Clint A; Johnson, Kent D | Not applicable based on title |
| [Evaluation of mental stress tests among medical students based on salivary sample collected just before the national license examination] | 2011 | Ushiki, Kazumi; Sato, Yuka; Arai, Katsuya; Ide, Norihumi; Matsui, Naoki; Handa, Hiroshi; Murakami, Hirokazu; Ogawara, Hatsue | Not applicable based on title |
| Monitoring fitness, fatigue and running performance during a pre-season training camp in elite football players | 2013 | Buchheit, M; Racinais, S; Bilsborough, J C; Bourdon, P C; Voss, S C; Hocking, J; Cordy, J; Mendez-Villanueva, A; Coutts, A J | Not applicable based on title |
| Associations between police lethal force errors, measures of diurnal and reactive cortisol, and mental health | 2022 | Chan, Jennifer F; Di Nota, Paula M; Planche, Kyle; Borthakur, Debanjan; Andersen, Judith P | Not applicable based on title |
| Cardiac autonomic modulation and sleepiness: physiological consequences of sleep deprivation due to 40 h of prolonged wakefulness | 2014 | Glos, Martin; Fietze, Ingo; Blau, Alexander; Baumann, Gert; Penzel, Thomas | Not applicable based on title |
| Testosterone and cortisol are more predictive of choice behavior than a social nudge in adult males on a simple gift give-get task | 2021 | Serpell, Benjamin G; Cook, Christian J | Not applicable based on title |
| The Intelligent Phenotypic Plasticity Platform (IP3) for Precision Medicine-Based Injury Prevention in Sport | 2005 | Kiefer, Adam W; Armitano-Lago, Cortney N; Sathyan, Anoop; MacPherson, Ryan; Cohen, Kelly; Silva, Paula L | Not applicable based on title |
| Oral contraception but not menstrual cycle phase is associated with increased free cortisol levels and low hypothalamo-pituitary-adrenal axis reactivity | 2013 | Boisseau, N; Enea, C; Diaz, V; DuguÈ, B; Corcuff, J B; Duclos, M | Not applicable based on title |
| Cognitive dietary restraint and cortisol: importance of pervasive concerns with appearance | 2006 | Putterman, Erin; Linden, Wolfgang | Not applicable based on title |
| Menstrual variation in the acute testosterone and cortisol response to laboratory stressors correlate with baseline testosterone fluctuations at a within- and between-person level | 2021 | Cook, Christian J; Fourie, Phillip; Crewther, Blair T | Not applicable based on title |
| The menstrual cycle and sexual behavior: relationship to eating, exercise, sleep, and health patterns | 2005 | Brown, Susan G; Morrison, Lynn A; Calibuso, Marites J; Christiansen, Tess M | Not applicable based on title |
| The menstrual cycle and its effect on the immune status of female endurance runners | 2002 | Burrows, M; Bird, S R; Bishop, N | Not applicable based on title |
| Immune Response in Women during Exercise in the Heat: A Spotlight on Oral Contraception | 2018 | Larsen, Brianna; Cox, Amanda J; Quinn, Karlee; Fisher, Rhiannon; Minahan, Clare | Not applicable based on title |
| Possible hormone predictors of physical performance in adolescent team sport athletes | 2019 | Martin, Alanna C; Heazlewood, Ian T; Kitic, Cecilia M; Lys, Isabelle; Johnson, Liam | Not applicable based on title |
| Stress hormones are associated with the neuronal correlates of instructed fear conditioning | 2013 | Merz, Christian Josef; Stark, Rudolf; Vaitl, Dieter; Tabbert, Katharina; Wolf, Oliver Tobias | Not applicable based on title |
| The impact of menstrual-cycle phase on basal and exercise-induced hormones, mood, anxiety and exercise performance in physically active women | 2021 | Paludo, Ana C; Cook, Christian J; Owen, Julian A; Woodman, Tim; Irwin, Jennifer; Crewther, Blair T | Not applicable based on title |
| Influence of test tasks with different cognitive demands on salivary cortisol concentrations in school students | 2012 | Minkley, N; Kirchner, W H | Not applicable based on title |
| Attachment status and salivary cortisol in a normal day and during simulated interpersonal stress in young men | 2008 | Rifkin-Graboi, Anne | Not applicable based on title |
| Dietary factors, time of the week, physical fitness and saliva cortisol: their modulatory effect on mental distress and mood | 2022 | Begdache, Lina; Sadeghzadeh, Saloumeh; Pearlmutter, Paul; Derose, Gia; Krishnamurthy, Pragna; Koh, Ahyeon | Not applicable based on title |
| The Effect of a Probiotic Complex on the Gut-Brain Axis: A Translational Study | 2005 | Nobile, Vincenzo; Giardina, Silvana; Puoci, Francesco | Not applicable based on title |
| Estradiol during (analogue-)trauma: Risk- or protective factor for intrusive re-experiencing? | 2022 | Franke, Laila K; Miedl, Stephan F; Danbˆck, Sarah K; Lohse, Johanna; Liedlgruber, Michael; B¸rkner, Paul-Christian; Pletzer, Belinda; Wilhelm, Frank H | Not applicable based on title |
| External and internal load measures during preseason training in men collegiate soccer athletes | 2021 | Fields, Jennifer B; Merigan, Justin M; Gallo, Sina; White, Jason B; Jones, Margaret T | chronic/Prolonged stressor |
| Achievement goal theory-based psychological skills training session buffers youth athletespsychophysiological responses to performance stress | 2020 | Hogue, Candace M. | Not applicable based on title |
| The effects of two equal-volume training protocols upon strength, body composition and salivary hormones in male rugby union players | 2016 | Crewther, B T; Heke, Tol; Keogh, Jwl | Not applicable based on title |
| "Seat of the soul"? The structure and function of the pineal gland in women with alleged spirit possession-Results of two experimental studies | 2020 | Bastos, Marco AurÈlio Vinhosa; Bastos, Paulo Roberto Haidamus de Oliveira; E Paez, Loyn· Eu· Flores; de Souza, Edna Oliveira; Bogo, Danielle; Perdomo, Renata Trentin; Portella, Renata Boschi; Ozaki, Jorge Guilherme Okanobo; Iandoli, DÈcio; Lucchetti, Giancarlo | Not applicable based on title |
| Does waterfall aerosol influence mucosal immunity and chronic stress? A randomized controlled clinical trial | 2017 | Grafetst‰tter, Carina; Gaisberger, Martin; Prossegger, Johanna; Ritter, Markus; Kolar~, Predrag; Pichler, Christina; Thalhamer, Josef; Hartl, Arnulf | Not applicable based on title |
| Recovery of the immune system after exercise | 2017 | Peake, Jonathan M; Neubauer, Oliver; Walsh, Neil P; Simpson, Richard J | Not applicable based on title |
| Course of ante- and postnatal depressive symptoms related to mothers' HPA axis regulation | 2018 | Laurent, Heidemarie; Goodman, Sherryl H; Stowe, Zachary N; Halperin, Meeka; Khan, Faaiza; Wright, Dorianne; Nelson, Benjamin W; Newport, D Jeffrey; Ritchie, James C; Monk, Catherine; Knight, Bettina | Not applicable based on title |
| Association of weight discrimination during pregnancy and postpartum with maternal postpartum health | 2019 | Incollingo Rodriguez, Angela C; Tomiyama, A Janet; Guardino, Christine M; Dunkel Schetter, Christine | Not applicable based on title |
| Higher body mass index (BMI) is associated with reduced glucocorticoid inhibition of inflammatory cytokine production following acute psychosocial stress in men | 2008 | Wirtz, Petra H; Ehlert, Ulrike; Emini, Luljeta; Suter, Tobias | Not applicable based on title |
| Stress-induced modulation of multiple memory systems during retrieval requires noradrenergic arousal | 2020 | Zerbes, Gundula; Kausche, Franziska Magdalena; M¸ller, Jana Christina; Wiedemann, Klaus; Schwabe, Lars | Not applicable based on title |
| Salivary cortisol changes in humans after winning or losing a dominance contest depend on implicit power motivation | 2006 | Wirth, Michelle M; Welsh, Kathryn M; Schultheiss, Oliver C | Not applicable based on title |
| Workplace bullying increases the risk of anxiety through a stress-induced ≤2-adrenergic receptor mechanism: a multisource study employing an animal model, cell culture experiments and human data | 2021 | Rajalingam, Dhaksshaginy; Nymoen, Ingeborg; Nyberg, Henriette; Nielsen, Morten Birkeland; Einarsen, StÂle Valvatne; Gjerstad, Johannes | Not applicable based on title |
| Effect of Oral Administration of Lactiplantibacillus plantarum SNK12 on Temporary Stress in Adults: A Randomized, Placebo-Controlled, Double-Blind, Parallel-Group Study | 2022 | Watanabe, Takumi; Hayashi, Kyoko; Takara, Tsuyoshi; Teratani, Takumi; Kitayama, Joji; Kawahara, Toshio | Not applicable based on title |
| Internal training load measures during a competitive season in collegiate women lacrosse athletes | 2020 | Fields, Jennifer B; Esco, Michael R; Merrigan, Justin J; White, Jason B; Jones, Margaret T | Not applicable based on title |
| Understanding associations between rumination and inflammation: A scoping review | 2022 | Szabo, Yvette Z; Burns, Christina M; Lantrip, Crystal | Not applicable based on title |
| Cognitive, neural and endocrine functioning during late pregnancy: An Event-Related Potentials study | 2019 | Fiterman, Ora; Raz, Sivan | Not applicable based on title |
| Salivary cortisol in longitudinal associations between affective symptoms and midlife cognitive function: A British birth cohort study | 2022 | John, Amber; Desai, Roopal; Saunders, Rob; Buckman, Joshua E J; Brown, Barbara; Nurock, Shirley; Michael, Stewart; Ware, Paul; Marchant, Natalie L; Aguirre, Elisa; Rio, Miguel; Cooper, Claudia; Pilling, Stephen; Richards, Marcus; Gaysina, Darya; Stott, Josh | Not applicable based on title |
| Islamic praying changes stress-related hormones and genes | 2022 | Sobhani, Vahid; Manshadi Mokari, Ehsan; Aghajani, Jafar; Hatef, Boshra | Not applicable based on title |
| Chronic, acute and protocol-dependent effects of exercise on psycho-physiological health during long-term isolation and confinement | 2022 | Abeln, V; Fomina, E; Popova, J; Braunsmann, L; Koschate, J; Mˆller, F; Fedyay, S O; Vassilieva, G Y; Schneider, S; Str¸der, H K; Klein, T | Not applicable based on title |
| Psychological resilience and diurnal salivary cortisol in young adulthood | 2022 | Nishimi, Kristen; Koenen, Karestan C; Coull, Brent A; Segerstrom, Suzanne C; Austin, S Bryn; Kubzansky, Laura D | Not applicable based on title |
| Behaviour of salivary testosterone and cortisol in men during an Ironman Triathlon | 2022 | Vaamonde, Diana; GarcÌa-Manso, Juan Manuel; Algar-Santacruz, Carolina; Abbasi, Asghar; Sarmiento, Samuel; Valverde-Esteve, Teresa | Not applicable based on title |
| Early life stress sensitizes youth to the influence of stress-induced cortisol on memory for affective words | 2021 | Kuhlman, Kate R; Mayer, Stefanie E; Vargas, Ivan; Lopez-Duran, Nestor L | Not applicable based on title |
| Summated training and match load predictors of salivary immunoglobulin-A, alpha-amylase, testosterone, cortisol and T:C profile changes in elite-level professional football players: A longitudinal analysis | 2022 | Springham, Matthew; Williams, Sean; Waldron, Mark; McLellan, Chris; Newton, Robert U | Not applicable based on title |
| Social dialogue triggers biobehavioral synchrony of partners' endocrine response via sex-specific, hormone-specific, attachment-specific mechanisms | 2021 | Djalovski, Amir; Kinreich, Sivan; Zagoory-Sharon, Orna; Feldman, Ruth | Not applicable based on title |
| Cognitive Resilience and Psychological Responses across a Collegiate Rowing Season | 2017 | Shields, Morgan R; Brooks, M Alison; Koltyn, Kelli F; Kim, Jee-Seon; Cook, Dane B | Chronic/Prolonged stressor |
| Effects of an offshore sailing competition on anthropometry, muscular performance, subjective wellness, and salivary cortisol in professional sailors | 2022 | Philippe, Kilian; Paillard, Thierry; Maurelli, Olivier; Moody, Jeremy; Prioux, Jacques | Not applicable based on title |
| Effects of Overtraining Status on the Cortisol Awakening Response-Endocrine and Metabolic Responses on Overtraining Syndrome (EROS-CAR) | 2021 | Anderson, Travis; Wideman, Laurie; Cadegiani, Flavio A; Kater, Claudio E | Not applicable based on title |
| Acute effects of mindfulness-based intervention on athlete cognitive function: An fNIRS investigation | 2022 | Zhu, Yuxin; Sun, Fenghua; Li, Chunxiao; Huang, Junhao; Hu, Min; Wang, Kangle; He, Shan; Wu, Jiarui | Not applicable based on title |
| Monitoring Internal Training Load, Stress-Recovery Responses, and Immune-Endocrine Parameters in Brazilian Jiu-Jitsu Training | 2022 | Campos, Flavia; Molina Correa, J˙lio C; Canevari, Vinicius C M; Branco, Braulio H M; Andreato, Leonardo V; de Paula Ramos, Solange | chronic/Prolonged stressor |
| Resistance training as an acute stressor in healthy young men: associations with heart rate variability, alpha-amylase, and cortisol levels | 2021 | Becker, Linda; Semmlinger, Lucas; Rohleder, Nicolas | Not applicable based on title |
| Identifying diurnal cortisol profiles among young adults: Physiological signatures of mental health trajectories | 2021 | Hoyt, Lindsay Till; Zeiders, Katharine H; Chaku, Natasha; Niu, Li; Cook, Stephanie H | Not applicable based on title |
| Hydrocortisone as an adjunct to brief cognitive-behavioural therapy for specific fear: Endocrine and cognitive biomarkers as predictors of symptom improvement | 2021 | Steudte-Schmiedgen, Susann; Fay, Emily; Capitao, Liliana; Kirschbaum, Clemens; Reinecke, Andrea | Not applicable based on title |
| Neuroendocrine Response and State Anxiety Due to Psychosocial Stress Decrease after a Training with Subject's Own (but Not Another) Virtual Body: An RCT Study | 2022 | Burin, Dalila; Cavanna, Gabriele; Rabellino, Daniela; Kotozaki, Yuka; Kawashima, Ryuta | Not applicable based on title |
| In sickness and in health: partner's physical and mental health predicts cortisol levels in couples | 2019 | Meyer, Dixie; Salas, Joanne; Barkley, Stephanie; Buchanan, Tony W | Not applicable based on title |
| Effects of A high intensity interval session on mucosal immune function and salivary hormones in male and female endurance athletes | 2020 | Monje, Camila; Rada, Isabel; Castro-Sepulveda, Mauricio; PeÒailillo, Luis; Deldicque, Louise; Zbinden-Foncea, Hermann | Not applicable based on title |
| Combined exercise modulates cortisol, testosterone, and immunoglobulin A levels in individuals living with HIV/AIDS | 2019 | Melo, Bruno P; Guariglia, DÈbora A; Pedro, Rafael E; Bertolini, Dennis A; de Paula Ramos, Solange; Peres, Sidney B; FranzÛi de Moraes, Solange M | Not applicable based on title |
| Social fear in US infants: the roles of hair and salivary cortisol | 2022 | Winebrake, Deaven A; Almeida, Carlos F; Tuladhar, Charu T; Kao, Katie; Meyer, Jerrold S; Tarullo, Amanda R | Not applicable based on title |
| Applied relaxation and cortisol secretion: findings from a randomized controlled indicated prevention trial in adults with stress, anxiety, or depressive symptoms | 2022 | Kische, Hanna; Zenker, Monique; Pieper, Lars; Beesdo-Baum, Katja; Asselmann, Eva | Not applicable based on title |
| Socio-demographic and psychosocial predictors of salivary cortisol from older male participants in the Speedwell prospective cohort study | 2022 | Spiga, Francesca; Lawton, Michael A; Lightman, Stafford L; Smith, George Davey; Ben-Shlomo, Yoav | Not applicable based on title |
| Impact of military training stress on hormone response and recovery | 2022 | Tait, Jamie L; Drain, Jace R; Corrigan, Sean L; Drake, Jeremy M; Main, Luana C | Included |
| Simultaneous quantification of endocannabinoids, oleoylethanolamide and steroid hormones in human plasma and saliva | 2020 | Ney, Luke J; Felmingham, Kim L; Bruno, Raimondo; Matthews, Allison; Nichols, David S | Not applicable based on title |
| Effects of 5-HTTLPR genotype and cognitive rumination on long-term cortisol reactivity measured in human hair | 2019 | Schepers, Robbie; Keulers, Esther H; Markus, C Rob | Not applicable based on title |
| Acute Effects of Exercise on Risk-Taking: Different Responses in Males and Females | 2022 | Thomson, Cynthia J; Gaetz, Michael; Rastad, Michael | Not applicable based on title |
| Acute inflammatory, cortisol, and soreness responses to supramaximal accentuated eccentric loading | 2021 | Merrigan, Justin J; Jones, Margaret T | Included |
| Acute psychosocial stress increases serum BDNF levels: an antagonistic relation to cortisol but no group differences after mental training | 2019 | Linz, R; Puhlmann, L M C; Apostolakou, F; Mantzou, E; Papassotiriou, I; Chrousos, G P; Engert, V; Singer, T | Not applicable based on title |
| Executive functioning and rumination as they relate to stress-induced cortisol curves | 2020 | Guevara, Jasmin E; Murdock, Kyle W | Not applicable based on title |
| Effects of regular sport activities on stress level in sporting and non-sporting university students | 2022 | Szmodis, M·rta; Zs·kai, Annam·ria; BlaskÛ, Gergely; FehÈr, Piroska; Ann·r, Dorina; Szir·ki, ZsÛfia; Alm·si, G·bor; Kemper, Han Cg | Not applicable based on title |
| Evidence of the Associations between Individual and Partner Autonomy Support and Physiological Stress in the Context of Conversations about Weight among Couples Who are Overweight or Obese during a 6-Month Intervention | 2022 | Denes, Amanda; Crowley, John P; Ponivas, Ambyre L P; Cornelius, Talea; Allred, Ryan J; Gettens, Katelyn M; Powers, Theodore A; Gorin, Amy A | Not applicable based on title |
| Effects of cognitive-behavioral stress management training in individuals with functional somatic symptoms - an exploratory randomized controlled trial | 2019 | Markert, Charlotte; Gomm, Claudia; Ehlert, Ulrike; Gaab, Jens; Nater, Urs M | Not applicable based on title |
| Response of muscle damage markers to an accentuated eccentric training protocol: do serum and saliva measurements agree? | 2022 | Gonz·lez-Hern·ndez, Jorge M; JimÈnez-Reyes, Pedro; CerÛn, JosÈ J; Tvarijonaviciute, Asta; Llorente-Canterano, Francisco J; MartÌnez-Aranda, Luis M; GarcÌa-Ramos, Amador | Included |
| Symmetric convolutional and adversarial neural network enables improved mental stress classification from EEG | 2022 | Fu, Ruiqi; Chen, Yi-Feng; Huang, Yongqi; Chen, Shuping; Duan, Feiyan; Li, Jiewei; Wu, Jianhui; Jiang, Dongmei; Gao, Junling; Gu, Jason; Zhang, Mingming; Chang, Chunqi | Not applicable based on title |
| Influence of periodizing dietary carbohydrate on iron regulation and immune function in elite triathletes | 2020 | McKay, Alannah K A; Heikura, Ida A; Burke, Louise M; Peeling, Peter; Pyne, David B; van Swelm, Rachel P L; Laarakkers, Coby M; Cox, Gregory R | Not applicable based on title |
| Relationships between Heart Rate Variability, Sleep Duration, Cortisol and Physical Training in Young Athletes | 2021 | Mishica, Christina; Kyrˆl‰inen, Heikki; Hynynen, Esa; Nummela, Ari; Holmberg, Hans-Christer; Linnamo, Vesa | Not applicable based on title |
| Acute hormonal response to kettlebell swing exercise differs depending on load, even when total work is normalized | 2021 | Raymond, Leanne M; Renshaw, Derek; Duncan, Michael J | Not applicable based on title |
| Stress Levels in Handball Coaching-Case Study: Preliminary Analysis of the Differences between Training and Match | 2022 | Foreti, Nikola; Nikolovski, Zoran; Mari, Dora; Gabrilo, Goran; Sekuli, Damir; Jaksi, Damjan; Drid, Patrik | Not applicable based on title |
| Effect of sleep efficiency on salivary metabolite profile and cognitive function during exercise in volleyball athletes | 2019 | Akazawa, Nobuhiko; Kobayashi, Naoko; Nakamura, Yuki; Kumagai, Hiroshi; Choi, Youngju; Maeda, Seiji | Included |
| Acute and chronic catabolic responses to crossfitÆ and resistance training in young males | 2020 | Faelli, Emanuela; Bisio, Ambra; Codella, Roberto; Ferrando, Vittoria; Perasso, Luisa; PanascÏ, Marco; Saverino, Daniele; Ruggeri, Piero | Not applicable based on title |
| The effect of training order on neuromuscular, endocrine and mood response to small-sided games and resistance training sessions over a 24-h period | 2020 | Sparkes, W; Turner, A N; Weston, M; Russell, M; Johnston, M J; Kilduff, L P | Included |
| Combining mental and physical stress: Synergy or interference? | 2021 | Finke, Johannes B; Zhang, Xinwei; Plein, Debora; Schilling, Thomas M; Sch‰chinger, Hartmut; Larra, Mauro F | Not applicable based on title |
| Integrative review of early life adversity and cortisol regulation in pregnancy | 2021 | Epstein, Crystal Modde; Houfek, Julia F; Rice, Michael J; Weiss, Sandra J | Not applicable based on title |
| Putting the squeeze on compression garments: current evidence and recommendations for future research: A systematic scoping review | 2022 | Weakley, Jonathon; Broatch, James; O'Riordan, Shane; Morrison, Matthew; Maniar, Nirav; Halson, Shona L | Not applicable based on title |
| Firefighter salivary cortisol responses following rapid heat stress | 2022 | Coehoorn, Cory J; Neary, J Patrick; Krigolson, Olave E; Service, Thomas W; Stuart-Hill, Lynneth A | Not applicable based on title |
| Are Pain Polymorphisms Associated with the Risk and Phenotype of Post-COVID Pain in Previously Hospitalized COVID-19 Survivors? | 2022 | Fern·ndez-de-Las-PeÒas, CÈsar; Giordano, Rocco; DÌaz-Gil, Gema; Gil-Crujera, Antonio; GÛmez-S·nchez, Stella M; Ambite-Quesada, Silvia; Arendt-Nielsen, Lars | Not applicable based on title |
| Loneliness and diurnal cortisol levels during COVID-19 lockdown: the roles of living situation, relationship status and relationship quality | 2022 | Hopf, Dora; Schneider, Ekaterina; Aguilar-Raab, Corina; Scheele, Dirk; Morr, Mitjan; Klein, Thomas; Ditzen, Beate; Eckstein, Monika | Not applicable based on title |
| Bright light increases alertness and not cortisol in healthy men: A forced desynchrony study under dim and bright light (I) | 2022 | Lok, R; Woelders, T; van Koningsveld, M J; Oberman, K; Fuhler, S G; Beersma, D G M; Hut, R A | Not applicable based on title |
| Psychophysiological stress markers during preseason among elite female soccer players | 2022 | Botelho, Renata; Abad, Cesar C C; Spadari, Regina C; Winckler, Ciro; Garcia, M·rcia C; Guerra, Ricardo L F | Chronic/Prolonged stressor |
| Effects of Lactococcus lactis subsp cremoris YRC3780 daily intake on the HPA axis response to acute psychological stress in healthy Japanese men | 2022 | Matsuura, Noriko; Motoshima, Hidemasa; Uchida, Kenji; Yamanaka, Yujiro | Not applicable based on title |
| Cognitive control and cortisol response to stress in generalised anxiety disorder: a study of working memory capacity with negative and neutral distractors | 2020 | LeMoult, Joelle; McCabe, Randi E; Hamedani, Atayeh; Yoon, K Lira | Not applicable based on title |
| The effects of exercise intensity on the cortisol response to a subsequent acute psychosocial stressor | 2021 | Caplin, A; Chen, F S; Beauchamp, M R; Puterman, E | Not applicable based on title |
| Physiological and cognitive responses to an antarctic expedition: A case report | 2016 | Anton-Solanas, Ana; O'Neill, Barry V; Morris, Tessa E; Dunbar, Joe | Not applicable based on title |
| Resistance exercise intensity does not influence neurotrophic factors response in equated volume schemes | 2020 | Lodo, Leandro; Moreira, Alexandre; Bacurau, Reury Frank P; Capitani, Carol D; Barbosa, Wesley P; Massa, Marcelo; Schoenfeld, Brad J; Aoki, Marcelo S | Not applicable based on title |
| Salivary cortisol response to psychosocial stress in the late evening depends on CRHR1 genotype | 2020 | Weeger, J; Ising, M; M¸ller-Myhsok, B; Uhr, M; Schmidt, U; Steiger, A | Not applicable based on title |
| The between-week reliability of neuromuscular, endocrine, and mood markers in soccer players and the repeatability of the movement demands during small-sided games | 2021 | Sparkes, William; Turner, Anthony N; Weston, Matthew; Russell, Mark; Johnston, Michael; Kilduff, Liam P | Not applicable based on title |
| Daily rumination about stress, sleep, and diurnal cortisol activity | 2020 | Sladek, Michael R; Doane, Leah D; Breitenstein, Reagan S | Not applicable based on title |
| Relationship between mental workload and salivary cortisol levels: A field study | 2005 | Zoaktafi, Mojgan; Kazemi, Reza; Choobineh, Alireza; SaboorYaraghi, AliAkbar; Nematolahi, Samane; Zakerian, Seyed Abolfazl | Not applicable based on title |
| Evaluation of the effect of a live interview in journalism students on salivary stress biomarkers and conventional stress scales | 2022 | Roca, Delfina; Escribano, Dami·n; Franco-MartÌnez, Lorena; Contreras-Aguilar, Maria D; Bernal, Luis J; Ceron, Jose J; Rojo-Villada, Pedro A; MartÌnez-Subiela, Silvia; Tvarijonaviciute, Asta | Not applicable based on title |
| Acute effects of nicotine on physiological responses and sport performance in healthy baseball players | 2022 | Fang, Shih-Hua; Lu, Chi-Cheng; Lin, Hua-Wei; Kuo, Kuan-Chen; Sun, Chen-Yu; Chen, Yi-Ying; Chang, Wen-Dien | Not applicable based on title |
| Salivary hormones and anxiety in winners and losers of an international judo competition | 2005 | Papacosta, Elena; Nassis, George P; Gleeson, Michael | Not applicable based on title |
| Effects of training with free weights versus machines on muscle mass, strength, free testosterone, and free cortisol levels | 2020 | Schwanbeck, Shane R; Cornish, Stephen M; Barss, Trevor; Chilibeck, Philip D | Not applicable based on title |
| Factors Predicting Training Delays and Attrition of Recruits during Basic Military Training | 2022 | Tait, Jamie L; Drain, Jace R; Bulmer, Sean; Gastin, Paul B; Main, Luana C | age |
| Cortisol stress reactivity in women, diurnal variations, and hormonal contraceptives: studies from the Family Health Patterns Project | 2019 | Lovallo, William R; Cohoon, Andrew J; Acheson, Ashley; Vincent, Andrea S; Sorocco, Kristen H | Not applicable based on title |
| A congested match schedule alters internal match load and affects salivary immunoglobulin A concentration in youth soccer players | 2022 | Mortatti, Arnaldo Luis; Oliveira, Romerito SÛstenes Canuto de; Pinto, J˙lio Cesar Barbosa de Lima; Galv„o-Coelho, Nicole Leite; de Almeida, RaÌssa NÛbrega; Aoki, Marcelo S; Moreira, Alexandre | Not applicable based on title |
| The Effect of Copaiba Oil Odor on Anxiety Relief in Adults under Mental Workload: A Randomized Controlled Trial | 2022 | Zhang, Nan; Chen, Jie; Dong, Wenyan; Yao, Lei | Not applicable based on title |
| Validation of a new method for saliva cortisol testing to assess stress in first responders | 2021 | Smeets, Math Mj; Vandenbossche, Piet; Duijst, Wilma Ljm; Mook, Walther Nka van; Leers, Mathie P G | Not applicable based on title |
| The physiological response to cold-water immersion following a mixed martial arts training session | 2017 | Lindsay, Angus; Carr, Sam; Cross, Sean; Petersen, Carl; Lewis, John G; Gieseg, Steven P | Not applicable based on title |
| Effects of psychosocial stress on prosociality: the moderating role of current life stress and thought control | 2022 | Hensel, Lisa; Rohleder, Nicolas; Niessen, Cornelia | Not applicable based on title |
| Diurnal cortisol dynamics, perceived stress, and language production in aphasia | 2019 | Laures-Gore, Jacqueline; Cahana-Amitay, Dalia; Buchanan, Tony W | Not applicable based on title |
| Differences in stress response between two altitudes assessed by salivary cortisol levels within circadian rhythms in long-distance runners | 2022 | Tsunekawa, Katsuhiko; Ushiki, Kazumi; Martha, Larasati; Nakazawa, Asuka; Hasegawa, Rika; Shimizu, Risa; Shimoda, Nozomi; Yoshida, Akihiro; Nakajima, Kiyomi; Kimura, Takao; Murakami, Masami | Included |
| Daily stressors and diurnal cortisol among sexual and gender minority young adults | 2021 | Figueroa, Wilson S; Zoccola, Peggy M; Manigault, Andrew W; Hamilton, Katrina R; Scanlin, Matt C; Johnson, Ryan C | Not applicable based on title |
| Investigation of the relationship between salivary cortisol, training load, and subjective markers of recovery in elite rugby union players | 2019 | Tiernan, Caoimhe; Lyons, Mark; Comyns, Tom; Nevill, Alan M; Warrington, Giles | Chronic/Prolonged stressor |
| Stress-Related Responses to Alternations between Repetitive Physical Work and Cognitive Tasks of Different Difficulties | 2020 | Mixter, Susanna; Mathiassen, Svend Erik; Lindfors, Petra; Dimberg, Kent; Jahncke, Helena; Lyskov, Eugene; Hallman, David M | Not applicable based on title |
| Dynamic behavior of cell-free mitochondrial DNA in human saliva | 2022 | Trumpff, Caroline; Rausser, Shannon; Haahr, Rachel; Karan, Kalpita R; Gouspillou, Gilles; Puterman, Eli; Kirschbaum, Clemens; Picard, Martin | Not applicable based on title |
| Joint associations of regular exercise and healthy diet with psychobiological stress reactivity in a healthy male sample | 2021 | Strahler, Jana; Wurst, Ramona; Fuchs, Reinhard; Wunsch, Kathrin | Not applicable based on title |
| Good vibrations: An observational study of real-life stress induced by a stage performance | 2020 | Everaerd, Daphne S; Henckens, Marloes J A G; Bloemendaal, Mirjam; Bovy, Leonore; Kaldewaij, Reinoud; Maas, Frederique M W M; Mulders, Peter C R; Niermann, Hannah C M; van de Pavert, Iris; Przezdzik, Izabela; Fern·ndez, GuillÈn; Klumpers, Floris; de Voogd, Lycia D | age |
| Adverse parenting is associated with blunted salivary cortisol awakening response and altered expression of glucocorticoid receptor ≤ and ≤2-adrenergic receptor mRNAs in leukocytes in Japanese medical students | 2017 | Kawai, Tomoko; Kuwano, Yuki; Masuda, Kiyoshi; Fujita, Kinuyo; Tanaka, Hiroki; Nishikawa, Tatsuya; Rokutan, Kazuhito; Nishida, Kensei | Not applicable based on title |
| A systematic review of non-motor symptom evaluation in clinical trials for amyotrophic lateral sclerosis | 2022 | Beswick, Emily; Forbes, Deborah; Hassan, Zack; Wong, Charis; Newton, Judith; Carson, Alan; Abrahams, Sharon; Chandran, Siddharthan; Pal, Suvankar | Not applicable based on title |
| Fatigue, Stress, and Performance during Alternating Physical and Cognitive Tasks-Effects of the Temporal Pattern of Alternations | 2021 | Mixter, Susanna; Mathiassen, Svend Erik; Bj‰rntoft, Sofie; Lindfors, Petra; Lyskov, Eugene; Hallman, David M | Not applicable based on title |
| Effect of cold water immersion performed on successive days on physical performance, muscle damage, and inflammatory, hormonal, and oxidative stress markers in volleyball players | 2019 | de Freitas, Victor H; Ramos, Solange P; Bara-Filho, MaurÌcio G; Freitas, Daniel G S; Coimbra, Danilo R; Cecchini, Rubens; Guarnier, Fl·via A; Nakamura, F·bio Y | Not applicable based on title |
| The cortisol awakening response predicts same morning executive function: results from a 50-day case study | 2015 | Law, Robin; Evans, Phil; Thorn, Lisa; Hucklebridge, Frank; Clow, Angela | Not applicable based on title |
| A single administration of 'microbial' D-alanine to healthy volunteers augments reaction to negative emotions: A comparison with D-serine | 2020 | Capit„o, Liliana P; Forsyth, Jessica; Thomaidou, Mia A; Condon, Mark D; Harmer, Catherine J; Burnet, Philip Wj | Not applicable based on title |
| Stress-related cortisol responsivity modulates prospective memory | 2017 | Glienke, K; Piefke, M | Not applicable based on title |
| Prolonged perceived stress and saliva cortisol in a large cohort of Danish public service employees: cross-sectional and longitudinal associations | 2017 | Mikkelsen, Sigurd; Forman, Julie Lyng; Fink, Samuel; Vammen, Marianne Agergaard; Thomsen, Jane Fr¯lund; Grynderup, Matias Br¯dsgaard; Hansen, ≈se Marie; Kaerlev, Linda; Kolstad, Henrik Albert; Rugulies, Reiner; Bonde, Jens Peter | Not applicable based on title |
| Sex-specific effects of low-dose hydrocortisone on threat detection in HIV | 2021 | Kamkwalala, Asante R; Maki, Pauline M; Langenecker, Scott A; Phan, K Luan; Weber, Kathleen M; Rubin, Leah H | Not applicable based on title |
| Endogenous oxytocin levels in extracted saliva elevates during breastfeeding correlated with lower postpartum anxiety in primiparous mothers | 2022 | Nagahashi-Araki, Miyuki; Tasaka, Makoto; Takamura, Tsunehiko; Eto, Hiromi; Sasaki, Noriko; Fujita, Wakako; Miyazaki, Asuka; Morifuji, Kanako; Honda, Naoko; Miyamura, Tunetake; Nishitani, Shota | Not applicable based on title |
| Seasonal differences in rhythmicity of salivary cortisol in healthy adults | 2019 | Kanikowska, Dominika; Roszak, Magdalena; Rutkowski, RafaB; Sato, Maki; Sikorska, Dorota; Orzechowska, Zofia; Brborowicz, Andrzej; Witowski, Janusz | Not applicable based on title |
| Brain-derived Neurotropic Factor val66met is a Strong Predictor of Decision Making and Attention Performance on the CONVIRT Virtual Reality Cognitive Battery | 2021 | Corrone, Michelle; Nanev, Aleshia; Amato, Isabella; Bicknell, Rowena; Wundersitz, Daniel William Taylor; van den Buuse, Maarten; Wright, Bradley James | Not applicable based on title |
| Salivary stress hormone response and performance in full competition after linear or undulating periodization training in elite powerlifters | 2020 | Landram, Michael J; Koch, Alexander J; Mayhew, Jerry L | Not applicable based on title |
| Introducing nature at the work floor: A nature-based intervention to reduce stress and improve cognitive performance | 2022 | Daniels, Silvie; Clemente, Diana B P; Desart, Steffie; Saenen, Nelly; Sleurs, Hanne; Nawrot, Tim S; Malina, Robert; Plusquin, Michelle | Not applicable based on title |
| Changes in acute biochemical markers of inflammatory and structural stress in rugby union | 2005 | Lindsay, Angus; Lewis, John; Scarrott, Carl; Draper, Nick; Gieseg, Steven P | Not applicable based on title |
| Effect of one night of sleep deprivation on the diurnal patterns of steroid hormones | 2017 | Labsy, Z.; Vibarel-Rebot, N.; Rieth, N.; Do, M.-C.; Gagey, O.; Zorgati, H.; Collomp, K. | Performance not measured |
| The association of the 5-HTTLPR polymorphism and the response to different stressors in healthy males | 2021 | Kuhn, Leandra; Noack, Hannes; Skoluda, Nadine; Wagels, Lisa; Rˆhr, Ann-Kristin; Schulte, Christina; Eisenkolb, Sana; Nieratschker, Vanessa; Derntl, Birgit; Habel, Ute | Not applicable based on title |
| Gay men's stress response to a†general and a†specific social stressor | 2021 | Sattler, Frank A; Nater, Urs M; Mewes, Ricarda | Not applicable based on title |
| Effectiveness and cost effectiveness of a stress management training for leaders of small and medium sized enterprises - study protocol for a randomized controlled-trial | 2021 | Lehmann, J A M; Schwarz, E; Rahmani Azad, Z; Gritzka, S; Seifried-D¸bon, T; Diebig, M; Gast, M; Kilian, R; Nater, U; Jarczok, M; Kessemeier, F; Braun, S; Balint, E; Rothermund, E; Junne, F; Angerer, P; G¸ndel, H | Not applicable based on title |
| The effect of HF-rTMS over the left DLPFC on stress regulation as measured by cortisol and heart rate variability | 2020 | Pulopulos, Matias M; Schmausser, Maximilian; De Smet, Stefanie; Vanderhasselt, Marie-Anne; Baliyan, Shishir; Venero, CÈsar; Baeken, Chris; De Raedt, Rudi | Not applicable based on title |
| Cortisol secretion predicts functional macro-scale connectivity of the visual cortex: A data-driven Multivoxel Pattern Analysis (MVPA) | 2020 | Muehlhan, Markus; Alexander, Nina; Trautmann, Sebastian; Weckesser, Lisa J; Vogel, Susanne; Kirschbaum, Clemens; Miller, Robert | Not applicable based on title |
| Association between the ACE I/D polymorphism and muscle injuries in Italian and Japanese elite football players | 2020 | Massidda, Myosotis; Miyamoto-Mikami, Eri; Kumagai, Hiroshi; Ikeda, Hayato; Shimasaki, Yu; Yoshimura, Masafumi; Cugia, Paolo; Piras, Francesco; Scorcu, Marco; Kikuchi, Naoki; CalÚ, Carla Maria; Fuku, Noriyuki | Not applicable based on title |
| Acute physiological and perceptual responses to a netball specific training session in professional female netball players | 2022 | Birdsey, Laurence P; Weston, Matthew; Russell, Mark; Johnston, Michael; Cook, Christian J; Kilduff, Liam P | Not applicable based on title |
| Psychological Distress and Personality Dimensions Associated with Romantic Orientation Among Japanese Adults | 2022 | Sasayama, Daimei; Chijiiwa, Miyuki; Nogawa, Shun; Saito, Kenji; Kunugi, Hiroshi | Not applicable based on title |
| Salivary free light chains as a new biomarker to measure psychological stress: the impact of a university exam period on salivary immunoglobulins, cortisol, DHEA and symptoms of infection | 2020 | Irshad, Lylah; Faustini, Sian; Evans, Lili; Drayson, Mark T; Campbell, John P; Heaney, Jennifer L J | Not applicable based on title |
| Noradrenergic Regulation of Cognitive Flexibility: No Effects of Stress, Transcutaneous Vagus Nerve Stimulation, and Atomoxetine on Task-switching in Humans | 2020 | Tona, Klodiana-Daphne; Revers, Hans; Verkuil, Bart; Nieuwenhuis, Sander | Not applicable based on title |
| Field-Based Assessments of Behavioral Patterns During Shiftwork in Police Academy Trainees Using Wearable Technology | 2022 | Erickson, Melissa L; Wang, Will; Counts, Julie; Redman, Leanne M; Parker, Daniel; Huebner, Janet L; Dunn, Jessilyn; Kraus, William E | Undefined stressor in criteria |
| Aerodynamic and acoustic voice measures before and after an acute public speaking stressor | 2020 | Perrine, Brittany L; Scherer, Ronald C | Not applicable based on title |
| Mixed-methods process evaluation of a residence-based SARS-CoV-2 testing participation pilot on a UK university campus during the COVID-19 pandemic | 2022 | Blake, H; Carlisle, S; Fothergill, L; Hassard, J; Favier, A; Corner, J; Ball, J K; Denning, C | Not applicable based on title |
| Cortisol levels decrease after acute tobacco abstinence in regular smokers | 2014 | Wong, Jordan A; Pickworth, Wallace B; Waters, Andrew J; al'Absi, Mustafa; Leventhal, Adam M | Not applicable based on title |
| Cortisol stress reactivity to the trier social stress test in obese adults | 2018 | Herhaus, Benedict; Petrowski, Katja | Not applicable based on title |
| Determinants and Mechanisms of the Renin-Aldosterone Stress Response | 2022 | Gideon, Angelina; Sauter, Christine; Pruessner, Jens C; Farine, Damien R; Wirtz, Petra H | Not applicable based on title |
| Food restriction alters salivary cortisol and ±-amylase responses to a simulated weightlifting competition without significant performance modification | 2018 | Durguerian, Alexandre; Filaire, Edith; Drogou, Catherine; Bougard, ClÈment; Chennaoui, Mounir | Not applicable based on title |
| Workplace design-related stress effects on prefrontal cortex connectivity and neurovascular coupling | 2021 | Alyan, Emad; Saad, Naufal M; Kamel, Nidal; Rahman, Mohammad Abdul | Not applicable based on title |
| Anticipatory salivary cortisol and state anxiety before competition predict match outcome in division I collegiate wrestlers | 2019 | Cintineo, Harry P; Arent, Shawn M | Not applicable based on title |
| No association between FKBP5 gene methylation and acute and long-term cortisol output | 2020 | Alexander, Nina; Kirschbaum, Clemens; Stalder, Tobias; Muehlhan, Markus; Vogel, Susanne | Not applicable based on title |
| A new polymer lab-on-a-chip (LOC) based on a microfluidic capillary flow assay (MCFA) for detecting unbound cortisol in saliva | 2020 | T U, Vinitha; Ghosh, Sthitodhi; Milleman, Alexander; Nguyen, Thinh; Ahn, Chong H | Not applicable based on title |
| Female Volleyball Players Are More Prone to Cortisol Anticipatory Stress Response than Sedentary Women | 2019 | Dziembowska, Inga; WÛjcik, MaBgorzata; HoByDska-Iwan, Iga; Litwic-Kaminska, Kamila; SBomka, Artur; {ekanowska, Ewa | Not applicable based on title |
| Hair cortisol and cognitive performance in healthy older people | 2014 | Pulopulos, Matias M; Hidalgo, Vanesa; Almela, Mercedes; Puig-Perez, Sara; Villada, Carolina; Salvador, Alicia | Not applicable based on title |
| Hormonal Differences in Intimate Partner Violence Perpetrators When They Cope with Acute Stress: A Pilot Study | 2021 | Romero-MartÌnez, ¡ngel; Blanco-GandÌa, Mari-Carmen; Rodriguez-Arias, Marta; Lila, Marisol; Moya-Albiol, Luis | Not applicable based on title |
| Analysis of Occupational Stress and Its Relationship with Secretory Immunoglobulin A in the Xinjiang Plateau Young Military Recruits | 2020 | Tao, Ning; An, Hengqing; Zhang, Jianjiang; Zhang, Yuanyue; Jin, Lu; Xu, Lei; Liu, Jiwen; Xu, Xinjuan | undefined stressor in criteria |
| Sleep and stress hormone responses to training and competition in elite female athletes | 2018 | O'Donnell, Shannon; Bird, Steve; Jacobson, Gregory; Driller, Matthew | Not applicable based on title |
| Dynamic DNA methylation changes in the COMT gene promoter region in response to mental stress and its modulation by transcranial direct current stimulation | 2021 | Wiegand, Ariane; Blickle, Arne; Br¸ckmann, Christof; Weller, Simone; Nieratschker, Vanessa; Plewnia, Christian | Not applicable based on title |
| Stress regulation and cognitive control: evidence relating cortisol reactivity and neural responses to errors | 2013 | Compton, Rebecca J; Hofheimer, Julia; Kazinka, Rebecca | Not applicable based on title |
| Upper respiratory tract infection and mucosal immunity in young ice hockey players during the pretournament training period | 2019 | Orysiak, Joanna; Witek, Konrad; Malczewska-Lenczowska, Jadwiga; Zembron-Lacny, Agnieszka; Pokrywka, Andrzej; Sitkowski, Dariusz | Not applicable based on title |
| Diurnal variations of hormonal secretion, alertness and cognition in extreme chronotypes under different lighting conditions | 2016 | Maierova, L; Borisuit, A; Scartezzini, J-L; Jaeggi, S M; Schmidt, C; M¸nch, M | Not applicable based on title |
| Effect of match importance on salivary cortisol and immunoglobulin A responses in elite young volleyball players | 2013 | Moreira, Alexandre; Freitas, Camila G; Nakamura, F·bio Yuzo; Drago, Gustavo; Drago, Murilo; Aoki, Marcelo S | Not applicable based on title |
| Changes in salivary antimicrobial peptides, immunoglobulin A and cortisol after prolonged strenuous exercise | 2011 | Usui, Tatsuya; Yoshikawa, Takahiro; Orita, Keisuke; Ueda, Shin-Ya; Katsura, Yoshihiro; Fujimoto, Shigeo; Yoshimura, Mamiko | Not applicable based on title |
| Relationships among salivary immunoglobulin A, lactoferrin and cortisol in basketball players during a basketball season | 2010 | He, Cheng-Shiun; Tsai, Min-Lung; Ko, Miau-Hwa; Chang, Chen-Kang; Fang, Shih-Hua | Not applicable based on title |
| Blunted opioid regulation of the HPA stress response during nicotine withdrawal: therapeutic implications | 2021 | al'Absi, Mustafa; Nakajima, Motohiro; DeAngelis, Briana; Grant, Jon; King, Andrea; Grabowski, John; Hatsukami, Dorothy; Allen, Sharon | Not applicable based on title |
| Effect of repetitive transcranial magnetic stimulation on the cognitive impairment induced by sleep deprivation: a randomized trial | 2021 | Li, Shangda; Zhou, Hetong; Yu, Yueran; Lyu, Hailong; Mou, Tingting; Shi, Gongde; Hu, Shaohua; Huang, Manli; Hu, Jianbo; Xu, Yi | Not applicable based on title |
| Acute Effects of 24-h Sleep Deprivation on Salivary Cortisol and Testosterone Concentrations and Testosterone to Cortisol Ratio Following Supplementation with Caffeine or Placebo | 2017 | Donald, Ciaran Mc; Moore, Joss; McIntyre, Alan; Carmody, Kevin; Donne, Bernard | Included |
| Reduced professional efficacy is associated with a blunted salivary alpha-amylase awakening response | 2019 | Landolt, Kathleen; Maruff, Paul; Horan, Ben; Kingsley, Michael; Kinsella, Glynda; O'Halloran, Paul D; Hale, Matthew W; Wright, Bradley J | Not applicable based on title |
| Racial/Ethnic Differences in Physiological Stress and Relapse among Treatment Seeking Tobacco Smokers | 2019 | Webb Hooper, Monica | Not applicable based on title |
| The "warrior" COMT val/met genotype occurs in greater frequencies in mixed martial arts fighters relative to controls | 2020 | Tartar, Jaime L; Cabrera, Dominick; Knafo, Sarah; Thomas, Julius D; Antonio, Jose; Peacock, Corey A | Not applicable based on title |
| Risk factors associated with acute respiratory illnesses in athletes: a systematic review by a subgroup of the IOC consensus on 'acute respiratory illness in the athlete' | 2022 | Derman, Wayne; Badenhorst, Marelise; Eken, Maaike; Gomez-Ezeiza, Josu; Fitzpatrick, Jane; Gleeson, Maree; Kunorozva, Lovemore; Mjosund, Katja; Mountjoy, Margo; Sewry, Nicola; Schwellnus, Martin | Not applicable based on title |
| Assessment of fatigue and recovery in sport: narrative review | 2022 | Bestwick-Stevenson, Thomas; Toone, Rebecca; Neupert, Emma; Edwards, Kimberley; Kluzek, Stefan | Not applicable based on title |
| Effects of Training Loads on Stress Tolerance and Mucosal Immunity in High-Intensity Functional Fitness Athletes | 2022 | Batista, Elias Dos Santos; Ribeiro, Bruno Laerte Lopes; Leite Galv„o-Coelho, Nicole; Almeida, RaÌssa NÛbrega de; Teixeira, RÙmulo Vasconcelos; Silveira, Jonatas Cardoso Da; Ferreira, Ayrton Bruno de Morais; Mortatti, Arnaldo Luis | Not applicable based on title |
| Stress and recovery in sports: Effects on heart rate variability, cortisol, and subjective experience | 2019 | Vacher, P; Filaire, E; Mourot, L; Nicolas, M | Not applicable based on title |
| Influence of two functional polymorphisms in NOS1 on baseline cortisol and working memory in healthy subjects | 2019 | Roth, N J; Zipperich, S; Kopf, J; Deckert, J; Reif, A | Not applicable based on title |
| Effects of prenatal education and type of delivery on stress axes | 2021 | UÁar, Cihat; B¸lb¸l, Mehmet; Yildiz, Sedat | Not applicable based on title |
| Can salivary testosterone and cortisol reactivity to a mid-week stress test discriminate a match outcome during international rugby union competition? | 2018 | Crewther, Blair T; Potts, Neil; Kilduff, Liam P; Drawer, Scott; Cook, Christian J | Included |
| Salivary biomarker responses to two final matches in women's professional football | 2016 | Maya, Javiera; Marquez, Pablo; PeÒailillo, Luis; Contreras-Ferrat, Ariel; Deldicque, Louise; Zbinden-Foncea, Hermann | Not applicable based on title |
| Effect of High-Intensity Interval Training Versus Small-Sided Games Training on Sleep and Salivary Cortisol Level | 2020 | Bonato, Matteo; La Torre, Antonio; Saresella, Marina; Marventano, Ivana; Merati, Giampiero; Banfi, Giuseppe; Vitale, Jacopo A | Included |
| Frontal control over automatic emotional action tendencies predicts acute stress responsivity | 2019 | Kaldewaij, Reinoud; Koch, Saskia B J; Zhang, Wei; Hashemi, Mahur M; Klumpers, Floris; Roelofs, Karin | Not applicable based on title |
| Brief Report: Low-Dose Hydrocortisone Has Acute Enhancing Effects on Verbal Learning in HIV-Infected Men | 2017 | Rubin, Leah H; Phan, K Luan; Keating, Sheila M; Weber, Kathleen M; Maki, Pauline M | Not applicable based on title |
| Higher body fat percentage is associated with increased cortisol reactivity and impaired cognitive resilience in response to acute emotional stress | 2009 | Mujica-Parodi, L R; Renelique, R; Taylor, M K | Not applicable based on title |
| An Impact of Psychological Stress on the Interplay between Salivary Oxidative Stress and the Classic Psychological Stress-Related Parameters | 2021 | Stojanovi, Nikola M; Randjelovi, Pavle J; Pavlovi, Dragana; Stojiljkovi, Nenad I; Jovanovi, Ivan; Sokolovi, Duaan; Radulovi, Niko S | Not applicable based on title |
| The effect of Chlorella pyrenoidosa supplementation on immune responses to 2†days of intensified training | 2018 | Chidley, Corinna; Davison, Glen | Not applicable based on title |
| Salivary testosterone and cortisol concentrations after two different resistance training exercises | 2019 | Geisler, Stephan; Aussieker, Thorben; Paldauf, Sven; Scholz, Sˆhnke; Kurz, Markus; Jungs, Sebastian; Rissmeyer, Matthias; Achtzehn, Silvia; Zinner, Christoph | Not applicable based on title |
| Physical Activity versus Psychological Stress: Effects on Salivary Cortisol and Working Memory Performance | 2019 | Ponce, Pamela; Del Arco, Alberto; Loprinzi, Paul | Not applicable based on title |
| A complementary intervention to promote wellbeing and stress management for early career teachers | 2021 | Hepburn, Stevie-Jae; Carroll, Annemaree; McCuaig-Holcroft, Louise | Not applicable based on title |
| Coping strategies influence cardiometabolic risk factors in chronic psychological stress: A post hoc analysis of A randomized pilot study | 2021 | Armborst, Deborah; Bitterlich, Norman; Alteheld, Birgit; Rˆsler, Daniela; Metzner, Christine; Siener, Roswitha | Not applicable based on title |
| The acute readiness monitoring scale: assessing predictive and concurrent validation | 2021 | Summers, Simon J; Keegan, Richard J; Flood, Andrew; Martin, Kristy; McKune, Andrew; Rattray, Ben | Included |
| Impact of menstrual cycle phase and oral contraceptives on sleep and overnight memory consolidation | 2021 | Plamberger, Christina Paula; Van Wijk, Helen Elisabeth; Kerschbaum, Hubert; Pletzer, Belinda Angela; Gruber, Georg; Oberascher, Karin; Dresler, Martin; Hahn, Michael Andreas; Hoedlmoser, Kerstin | Not applicable based on title |
| The SF-36 component summary scales and the daytime diurnal cortisol profile | 2010 | Hagger-Johnson, Gareth Edward; Whiteman, Martha C; Wawrzyniak, Andrew J; Holroyd, Warren G | Not applicable based on title |
| High cortisol awakening response is associated with impaired error monitoring and decreased post-error adjustment | 2015 | Zhang, Liang; Duan, Hongxia; Qin, Shaozheng; Yuan, Yiran; Buchanan, Tony W; Zhang, Kan; Wu, Jianhui | Not applicable based on title |
| Effect of a kickboxing match on salivary cortisol and immunoglobulin A | 2010 | Moreira, Alexandre; Arsati, Franco; Lima-Arsati, Ynara Bosco De Oliveira; Franchini, Emerson; De Ara˙jo, Vera Cavalcanti | Not applicable based on title |
| Salivary diagnostic markers in males and females during rest and exercise | 2017 | Rutherfurd-Markwick, Kay; Starck, Carlene; Dulson, Deborah K; Ali, Ajmol | Included |
| Probiotic supplementation elicits favourable changes in muscle soreness and sleep quality in rugby players | 2021 | Harnett, Joanna E; Pyne, David B; McKune, Andrew J; Penm, Jonathan; Pumpa, Kate L | Not applicable based on title |
| Cortisol and cardiovascular reactions to mental stress and antibody status following hepatitis B vaccination: a preliminary study | 2002 | Burns, Victoria E; Ring, Christopher; Drayson, Mark; Carroll, Douglas | Not applicable based on title |
| ≤-Adrenergic Contributions to Emotion and Physiology During an Acute Psychosocial Stressor | 2021 | MacCormack, Jennifer K; Armstrong-Carter, Emma L; Gaudier-Diaz, Monica M; Meltzer-Brody, Samantha; Sloan, Erica K; Lindquist, Kristen A; Muscatell, Keely A | Not applicable based on title |
| Can haematological and hormonal biomarkers predict fitness parameters in youth soccer players? A pilot study | 2020 | Perroni, Fabrizio; Migliaccio, Silvia; Borrione, Paolo; Vetrano, Mario; Amatori, Stefano; Sisti, Davide; Rocchi, Marco B L; Salerno, Gerardo; Vescovo, Riccardo Del; Cavarretta, Elena; Guidetti, Laura; Baldari, Carlo; Visco, Vincenzo | Not applicable based on title |
| A comparison of the acute effects of different forms of yoga on physiological and psychological stress: A pilot study | 2020 | Marshall, Mallory; McClanahan, McKenzie; McArthur Warren, Sarah; Rogers, Rebecca; Ballmann, Christopher | Not applicable based on title |
| The impact of a short burst of exercise on sleep inertia | 2021 | Kovac, Katya; Vincent, Grace E; Paterson, Jessica L; Reynolds, Amy; Aisbett, Brad; Hilditch, Cassie J; Ferguson, Sally A | Not applicable based on title |
| Salivary cortisol and alpha-amylase: Is there consistency between psychosocial stress test and burdensome work shifts? | 2017 | Karhula, Kati; H‰rm‰, Mikko; Sallinen, Mikael; Lindholm, Harri; Hirvonen, Ari; Elovainio, Marko; Kivim‰ki, Mika; Vahtera, Jussi; Puttonen, Sampsa | Not applicable based on title |
| Probiotic Mixture Containing Lactobacillus helveticus, Bifidobacterium longum and Lactiplantibacillus plantarum Affects Brain Responses to an Arithmetic Stress Task in Healthy Subjects: A Randomised Clinical Trial and Proof-of-Concept Study | 2022 | Edebol Carlman, Hanna M T; Rode, Julia; Kˆnig, Julia; Repsilber, Dirk; Hutchinson, Ashley N; Thunberg, Per; Persson, Jonas; Kiselev, Andrey; Pruessner, Jens C; Brummer, Robert J | Not applicable based on title |
| Partial-Body Cryotherapy Exposure 2†Hours Prior to a Shuttle Run Does Not Enhance Running Performance | 2022 | Partridge, Emily M; Cooke, Julie; McKune, Andrew J; Pyne, David B | Not applicable based on title |
| Effect of alterations in whole-body cryotherapy (WBC) exposure on post-match recovery markers in elite Premier League soccer players | 2022 | Malone, James J; Hodges, Daniel; Roberts, Craig; Sinclair, Jonathan K; Page, Richard M; Allan, Robert | Not applicable based on title |
| The effects of a high carbohydrate diet on cortisol and salivary immunoglobulin A (s-IgA) during a period of increase exercise workload amongst Olympic and Ironman triathletes | 2005 | Costa, R J S; Jones, G E; Lamb, K L; Coleman, R; Williams, J H H | Not applicable based on title |
| Sleep and salivary testosterone and cortisol during a short preseason camp: A study in professional rugby union | 2019 | Serpell, Benjamin G; Horgan, Barry G; Colomer, Carmen M E; Field, Byron; Halson, Shona L; Cook, Christian J | Included |
| Telomere length and ADHD symptoms in young adults | 2021 | Momany, Allison M; Lussier, Stephanie; Nikolas, Molly A; Stevens, Hanna | Not applicable based on title |
| The Effect of Acute Intense Exercise on Activity of Antioxidant Enzymes in Smokers and Non-Smokers | 2021 | Nobari, Hadi; Nejad, Hamzeh Abdi; Kargarfard, Mehdi; Mohseni, Soghra; Suzuki, Katsuhiko; Carmelo Adsuar, JosÈ; PÈrez-GÛmez, Jorge | Not applicable based on title |
| Effects of training and competition load on neuromuscular recovery, testosterone, cortisol, and match performance during a season of professional football | 2018 | Rowell, Amber E; Aughey, Robert J; Hopkins, William G; Esmaeili, Alizera; Lazarus, Brendan H; Cormack, Stuart J | Not applicable based on title |
| Salivary cortisol and nitrite concentrations in school teachers: A longitudinal pilot study | 2018 | de Oliveira Silva, Regisnei Aparecido; Agricola, Nestor Persio Alvim; Guillo, Lidia Andreu | Not applicable based on title |
| Anticipated next-day demand affects the magnitude of the cortisol awakening response, but not subjective or objective sleep | 2018 | Elder, Greg J; Barclay, Nicola L; Wetherell, Mark A; Ellis, Jason G | Not applicable based on title |
| Effects of Wearing a Jaw-repositioning Intra-oral Device in Synchronized Swimming Athletes | 2020 | Solana-Tramunt, Monica; Busc‡, Bernat; Morales, Jose; MirÛ, Adri‡; Aguilera-Castells, Joan; Arboix-AliÛ, Jordi | Not applicable based on title |
| A short humorous intervention protects against subsequent psychological stress and attenuates cortisol levels without affecting attention | 2021 | Froehlich, Eva; Madipakkam, Apoorva Rajiv; Craffonara, Barbara; Bolte, Christina; Muth, Anne-Katrin; Park, Soyoung Q | Not applicable based on title |
| Frontal Electroencephalogram Alpha Asymmetry during Mental Stress Related to Workplace Noise | 2021 | Alyan, Emad; Saad, Naufal M; Kamel, Nidal; Yusoff, Mohd Zuki; Zakariya, Mohd Azman; Rahman, Mohammad Abdul; Guillet, Christophe; Merienne, Frederic | Not applicable based on title |
| Impact of Cognitive Tasks on CO2 and Isoprene Emissions from Humans | 2021 | Gall, Elliott T; Mishra, Asit Kumar; Li, Jiayu; Schiavon, Stefano; Laguerre, AurÈlie | Not applicable based on title |
| The stress of competing: cortisol and amylase response to training and competition | 2021 | De Pero, Roberta; Minganti, Carlo; Cibelli, Giuseppe; Cortis, Cristina; Piacentini, Maria Francesca | Not applicable based on title |
| Estradiol therapy after menopause mitigates effects of stress on cortisol and working memory | 2017 | Herrera, Alexandra Ycaza; Hodis, Howard N; Mack, Wendy J; Mather, Mara | Not applicable based on title |
| Changes in salivary hormones, immunoglobulin A, and C-reactive protein in response to ultra-endurance exercises | 2014 | Tauler, Pedro; Martinez, Sonia; Moreno, Carlos; MartÌnez, Pau; Aguilo, Antoni | Not applicable based on title |
| Caffeine alters emotion and emotional responses in low habitual caffeine consumers | 2018 | Giles, Grace E; Spring, Alexander M; Urry, Heather L; Moran, Joseph M; Mahoney, Caroline R; Kanarek, Robin B | Not applicable based on title |
| Do dogs rescue their owners from a stressful situation? A behavioral and physiological assessment | 2020 | Carballo, Fabricio; Dzik, Victoria; Freidin, Esteban; Dami·n, Juan Pablo; Casanave, Emma B; Bentosela, Mariana | Not applicable based on title |
| Transcendental meditation and hypothalamic-pituitary-adrenal axis functioning: a pilot, randomized controlled trial with young adults | 2020 | Klimes-Dougan, Bonnie; Chong, Li Shen; Samikoglu, Ali; Thai, Michelle; Amatya, Palistha; Cullen, Kathryn R; Lim, Kelvin O | Not applicable based on title |
| Blunting of exercise-induced salivary testosterone in elite-level triathletes with a 10-day training camp | 2015 | Hough, John; Robertson, Caroline; Gleeson, Michael | Not applicable based on title |
| Stress-induced bias of multiple memory systems during retrieval depends on training intensity | 2021 | Zerbes, Gundula; Schwabe, Lars | Not applicable based on title |
| FKBP5 polymorphisms influence pre-learning stress-induced alterations of learning and memory | 2017 | Zoladz, Phillip R; Dailey, Alison M; Nagle, Hannah E; Fiely, Miranda K; Mosley, Brianne E; Brown, Callie M; Duffy, Tessa J; Scharf, Amanda R; Earley, McKenna B; Rorabaugh, Boyd R | Not applicable based on title |
| Integrative psycho-biophysiological markers in predicting psychological resilience | 2021 | Lau, Way K W; Tai, Alan P L; Chan, Jackie N M; Lau, Benson W M; Geng, Xiujuan | Not applicable based on title |
| No Effect of Chronotype on Sleepiness, Alertness, and Sustained Attention during a Single Night Shift | 2021 | Reiter, Andrew M; Sargent, Charli; Roach, Gregory D | Not applicable based on title |
| Comparison of peroxidase response to mental arithmetic stress in saliva of smokers and non-smokers | 2007 | Goi, Nobuhiro; Hirai, Yuuko; Harada, Hitoshi; Ikari, Akira; Ono, Takahiko; Kinae, Naohide; Hiramatsu, Mitsuo; Nakamura, Kimitsugu; Takagi, Kuniaki | Not applicable based on title |
| Effect of 8-week n-3 fatty-acid supplementation on oxidative stress and inflammation in middle- and long-distance running athletes: a pilot study | 2020 | Buonocore, Daniela; Verri, Manuela; Giolitto, Andrea; Doria, Enrico; Ghitti, Michele; Dossena, Maurizia | Not applicable based on title |
| Salivary secretory immunoglobulin A variation between female varsity athletes and nonathletes | 2020 | Chaney, Carlye; Quinn, Elizabeth A | Not applicable based on title |
| Acupuncture and responses of immunologic and endocrine markers during competition | 2003 | Akimoto, Takayuki; Nakahori, Chikako; Aizawa, Katsuji; Kimura, Fuminori; Fukubayashi, Toru; Kono, Ichiro | Not applicable based on title |
| The cortisol awakening response is associated with performance of a serial sequence reaction time task | 2016 | Hodyl, Nicolette A; Schneider, Luke; Vallence, Ann-Maree; Clow, Angela; Ridding, Michael C; Pitcher, Julia B | Not applicable based on title |
| Intimate partner violence and hair cortisol concentration: A biomarker for HPA axis function | 2020 | Alhalal, Eman; Falatah, Rawaih | Not applicable based on title |
| Effects of soy lecithin phosphatidic acid and phosphatidylserine complex (PAS) on the endocrine and psychological responses to mental stress | 2004 | Hellhammer, J; Fries, E; Buss, C; Engert, V; Tuch, A; Rutenberg, D; Hellhammer, D | Not applicable based on title |
| The detection of cortisol in human sweat: implications for measurement of cortisol in hair | 2014 | Russell, Evan; Koren, Gideon; Rieder, Michael; Van Uum, Stan H M | Not applicable based on title |
| Effects of resistance exercise on the HPA axis response to psychological stress during short-term smoking abstinence in men | 2014 | Ho, Jen-Yu; Kraemer, William J; Volek, Jeff S; Vingren, Jakob L; Fragala, Maren S; Flanagan, Shawn D; Maladouangdock, Jesse; Szivak, Tunde K; Hatfield, Disa L; Comstock, Brett A; Dunn-Lewis, Courtenay; Ciccolo, Joseph T; Maresh, Carl M | Not applicable based on title |
| Effects of high intensity interval training on cardiorespiratory fitness and salivary levels of IL-8, IL-1ra, and IP-10 in adults with asthma and non-asthma controls | 2022 | O'Neill, Carley D; Patlan, Ilana; Jeffery, Michael; Lewis, Danielle; Jenkins, Michael; Jones-Taggart, Holly; Green-Johnson, Julia; Dogra, Shilpa | Not applicable based on title |
| Unique diagnostic signatures of concussion in the saliva of male athletes: the Study of Concussion in Rugby Union through MicroRNAs (SCRUM) | 2021 | Di Pietro, Valentina; O'Halloran, Patrick; Watson, Callum N; Begum, Ghazala; Acharjee, Animesh; Yakoub, Kamal M; Bentley, Conor; Davies, David J; Iliceto, Paolo; Candilera, Gabriella; Menon, David K; Cross, Matthew J; Stokes, Keith A; Kemp, Simon Pt; Belli, Antonio | Not applicable based on title |
| The effects of acute stress on attentional networks and working memory in females | 2021 | Stone, Caleb; Ney, Luke; Felmingham, Kim; Nichols, David; Matthews, Allison | Not applicable based on title |
| Use of saliva stress biomarkers to estimate novice male endoscopist's stress during training in a high-end simulator | 2021 | Boyanov, Nikola; Georgiou, Konstantinos; Thanasas, Dimitrios; Deneva, Tanya; Oussi, Ninos; Marinov, Blagoi; Enochsson, Lars | Not applicable based on title |
| Are beards honest signals of male dominance and testosterone? | 2021 | Kowal, Marta; Sorokowski, Piotr; {elazniewicz, Agnieszka; Nowak, Judyta; Orzechowski, Sylwester; {urek, Grzegorz; {urek, Alina; Nawrat, Magdalena | Not applicable based on title |
| Intensified training period increases salivary iga responses but does not affect the severity of upper respiratory tract infection symptoms in prepuberal rhythmic gymnasts | 2018 | Antualpa, Kizzy; Aoki, Marcelo Saldanha; Moreira, Alexandre | Not applicable based on title |
| The Effects of a 4-Week, Intensified Training, and Competition Period on Salivary Hormones, Immunoglobulin A, Illness Symptoms, and Mood State in Elite Synchronised Swimmers | 2017 | Tanner, Amy; Day, Shannon | Not applicable based on title |
| The effects of captivity survival training on mood, dissociation, PTSD symptoms, cognitive performance and stress hormones | 2017 | Suurd Ralph, Cindy; Vartanian, Oshin; Lieberman, Harris R; Morgan, Charles A; Cheung, Bob | Undefined stressor in criteria |
| Impact of physical fitness on salivary stress markers in sedentary to low-active young to middle-aged men | 2016 | Strahler, Jana; Fuchs, Reinhard; Nater, Urs M; Klaperski, Sandra | Not applicable based on title |
| Effect of cortisol diurnal rhythm on emotional memory in healthy young adults | 2017 | Nagamine, Mitsue; Noguchi, Hiroko; Takahashi, Nobuaki; Kim, Yoshiharu; Matsuoka, Yutaka | Not applicable based on title |
| Salivary cortisol responses and session ratings of perceived exertion to a rugby match and fatigue test | 2017 | Caetano J˙nior, P C; Castilho, M L; Raniero, L | Not applicable based on title |
| The role of agonistic striving in the association between cortisol and high blood pressure | 2017 | Ewart, Craig K; Elder, Gavin J; Jorgensen, Randall S; Fitzgerald, Sheila T | Not applicable based on title |
| Is salivary cortisol moderating the relationship between salivary testosterone and hand-grip strength in healthy men? | 2017 | Crewther, Blair T; Thomas, Andrew G; Stewart-Williams, Steve; Kilduff, Liam P; Cook, Christian J | Not applicable based on title |
| Effect of competition on salivary cortisol, immunoglobulin A, and upper respiratory tract infections in elite young soccer players | 2012 | Mortatti, Arnaldo L; Moreira, Alexandre; Aoki, Marcelo S; Crewther, Blair T; Castagna, Carlo; de Arruda, Ademir F S; Filho, JosÈ M | Not applicable based on title |
| The role of stress hormones in the relationship between resting blood pressure and coagulation activity | 2006 | Wirtz, Petra H; Ehlert, Ulrike; Emini, Luljeta; R¸dis¸li, Katharina; Groessbauer, Sara; Mausbach, Brent T; von K‰nel, Roland | Not applicable based on title |
| Positive affect moderates the relationship between salivary testosterone and a health behavior composite in university females | 2020 | Martin, Luci A; Ter-Petrosyan, Mariam | Not applicable based on title |
| Salivary endocrine response following a maximal incremental cycling protocol with local vibration | 2020 | Jemni, MonËm; Marina, Michel; Delextrat, Anne; Tanner, Amy; Basset, Fabien A; Gu, Yaodong; Hu, Qiuli; Zhou, Huiyu; Mkaouer, Bessem; Konukman, Ferman | Not applicable based on title |
| Upper respiratory tract symptoms and salivary immunoglobulin A of elite female gymnasts: a full year longitudinal field study | 2020 | Dumortier, Jasmien; Bishop, Nicolette C; Vogelaers, Dirk; Boone, Jan; Delesie, Liesbeth; Tobback, Els; Mariman, An; Bourgois, Jan G | Not applicable based on title |
| Effect of Multi-Strain Probiotic Supplementation on URTI Symptoms and Cytokine Production by Monocytes after a Marathon Race: A Randomized, Double-Blind, Placebo Study | 2021 | Tavares-Silva, Edgar; Caris, Aline Venticinque; Santos, Samile Amorin; Ravacci, Graziela Rosa; Thomatieli-Santos, Ronaldo Vagner | Not applicable based on title |
| Sex-related Differences in Stress Reactivity and Cingulum White Matter | 2021 | Wheelock, M D; Goodman, A M; Harnett, N G; Wood, K H; Mrug, S; Granger, D A; Knight, D C | Not applicable based on title |
| Salivary hormones, IgA, and performance during intense training and tapering in judo athletes | 2013 | Papacosta, Elena; Gleeson, Michael; Nassis, George P | Not applicable based on title |
| Predicting emotional responses to potentially traumatic events from pre-exposure waking cortisol levels: a longitudinal study of police and firefighters | 2005 | Pineles, Suzanne L; Rasmusson, Ann M; Yehuda, Rachel; Lasko, Natasha B; Macklin, Michael L; Pitman, Roger K; Orr, Scott P | Not applicable based on title |
| Effect of pre-season training phase on anthropometric, hormonal and fitness parameters in young soccer players | 2019 | Perroni, Fabrizio; Fittipaldi, Simona; Falcioni, Lavinia; Ghizzoni, Lucia; Borrione, Paolo; Vetrano, Mario; Del Vescovo, Riccardo; Migliaccio, Silvia; Guidetti, Laura; Baldari, Carlo | Not applicable based on title |
| Session RPE and salivary immune-endocrine responses to simulated and official basketball matches in elite young male athletes | 2012 | Moreira, A; Crewther, B; Freitas, C G; Arruda, A F S; Costa, E C; Aoki, M S | Not applicable based on title |
| A Brief Body-Mind-Spirit Group Therapy for Chinese Medicine Stagnation Syndrome: A Randomized Controlled Trial | 2018 | Ng, Siu-Man; Leng, Lingli; Ho, Rainbow T H; Zhang, Zhangjin; Wang, Qi | Not applicable based on title |
| Resting salivary levels of IgA and cortisol are significantly affected during intensive resistance training periods in elite male weightlifters | 2012 | Tsai, Min-Lung; Li, Tzai-Li; Chou, Li-Wei; Chang, Chen-Kang; Huang, Shu-Yi; Fang, Shih-Hua | Not applicable based on title |
| Rapid noninvasive measurement of hormones in transdermal exudate and saliva | 2002 | Cook, Christian J | Not applicable based on title |
| Working hours, sleep, salivary cortisol, fatigue and neuro-behavior during Mars analog mission: five crews study | 2012 | Rai, Balwant; Foing, Bernard H; Kaur, Jasdeep | Not applicable based on title |
| Intensified training increases salivary free light chains in trained cyclists: Indication that training volume increases oral inflammation | 2018 | Heaney, Jennifer L J; Killer, Sophie C; Svendsen, Ida S; Gleeson, Michael; Campbell, John P | Not applicable based on title |
| Upper†respiratory symptoms (URS) and salivary responses across a season in youth soccer players: A useful and non-invasive approach associated to URS susceptibility and occurrence in young athletes | 2020 | Lopes, Renata Fiedler; Minuzzi, Luciele Guerra; Figueiredo, AntÛnio JosÈ; GonÁalves, Carlos; Tessitore, Antonio; Capranica, Laura; Teixeira, Ana Maria; Rama, Luis | Not applicable based on title |
| Salivary and plasma cortisol and testosterone responses to interval and tempo runs and a bodyweight-only circuit session in endurance-trained men | 2005 | Tanner, Amy Vivien; Nielsen, Birthe Vejby; Allgrove, Judith | Not applicable based on title |
| Assessing the association of university stress and physiological reactivity with decision-making among students | 2020 | Kuhnell, Raymond; Whitwell, Zoe; Arnold, Steven; Kingsley, Michael I C; Hale, Matthew W; Wahrendorf, Morten; Dragano, Nico; Wright, Bradley J | Not applicable based on title |
| Feasibility of autism-focused public speech training using a simple virtual audience for autism spectrum disorder | 2020 | Kumazaki, Hirokazu; Muramatsu, Taro; Kobayashi, Kazuki; Watanabe, Tetsuyou; Terada, Kazunori; Higashida, Haruhiro; Yuhi, Teruko; Mimura, Masaru; Kikuchi, Mitsuru | Not applicable based on title |
| Effects of antenatal hypnosis on maternal salivary cortisol during childbirth and six weeks postpartum-A randomized controlled trial | 2020 | Werner, Anette; Wu, Chunsen; Zachariae, Robert; Nohr, Ellen A; Uldbjerg, Niels; Hansen, ≈se Marie | Not applicable based on title |
| Effect of a simulated tactical occupation task on physiological strain index, stress and inflammation | 2020 | Morris, Cody E; Winchester, Lee J; Jackson, Andrew J; Tomes, Ariel S; Neal, Wesley A; Wilcoxen, Damon M; Chander, Harish; Arnett, Scott W | Not applicable based on title |
| Combining proactive transcranial stimulation and cardiac biofeedback to substantially manage harmful stress effects | 2021 | Schlatter, Sophie; Guillot, Aymeric; Schmidt, Laura; Mura, Mathilde; Trama, Robin; Di Rienzo, Franck; Lilot, Marc; Debarnot, Ursula | Not applicable based on title |
| The Positive Modulation Effect of a 6-Week Consumption of an Anthocyanin-Rich Mulberry Milk on Working Memory, Cholinergic, and Monoaminergic Functions in Healthy Working-Age Adults | 2021 | Thukham-Mee, Wipawee; Wattanathorn, Jintanaporn; Paholpak, Pongsatorn; Ransikachi, Poonsri; Piyavhatkul, Nawanant | Not applicable based on title |
| Refinement of saliva microRNA biomarkers for sports-related concussion | 2021 | Hicks, Steven D; Onks, Cayce; Kim, Raymond Y; Zhen, Kevin J; Loeffert, Jayson; Loeffert, Andrea C; Olympia, Robert P; Fedorchak, Gregory; DeVita, Samantha; Gagnon, Zofia; McLoughlin, Callan; Madeira, Miguel M; Zuckerman, Scott L; Lee, Timothy; Heller, Matthew; Monteith, Chuck; Campbell, Thomas R; Neville, Christopher; Fengler, Elise; Dretsch, Michael N | Not applicable based on title |
| Response of salivary biomarkers to an empathy triggering film sequence-a pilot study | 2021 | Zenzmaier, Christoph; Janssen, Jessie; Zulmin, Christoph; ÷sterreicher, Philipp; Heinrich, Lea; Tucek, Gerhard; Perkhofer, Susanne | Not applicable based on title |
| Polymorphonuclear leucocyte phagocytic function, ≥¥ T-lymphocytes and testosterone as separate stress-responsive markers of prolonged, high-intensity training programs | 2021 | Leal, Diogo V; Standing, Ariane S I; Furmanski, Anna L; Hough, John | Not applicable based on title |
| Effect of Acute Sleep Hygiene on Salivary Cortisol Level Following A Late Night Soccer-Specific Training Session | 2020 | Bonato, Matteo; Merati, Giampiero; La Torre, Antonio; Saresella, Marina; Marvetano, Ivana; Banfi, Giuseppe; Vitale, Jacopo A | Not applicable based on title |
| The biobehavioral impacts of sexual violence: Findings from an acute repeat survivor of vaginal rape | 2021 | Anderson, Katherine M; Ghosh, Mimi; Karris, Maile Y; Capozzi, Eleanor; Granger, Douglas A; Stockman, Jamila K | Not applicable based on title |
| How to alleviate the agony of providing negative feedback: Emotion regulation strategies affect hormonal stress responses to a managerial task | 2021 | Burk, Christian L; Wiese, Bettina S | Not applicable based on title |
| The psycho-hormonal influence of anaerobic fatigue on semi-professional female soccer players | 2017 | Broodryk, AdÈle; Pienaar, Cindy; Edwards, David; Sparks, Martinique | Not applicable based on title |
| Salivary cortisol in top-level professional soccer players | 2009 | Moreira, Alexandre; Arsati, Franco; de Oliveira Lima Arsati, Ynara Bosco; da Silva, Danilo Augusto; de Ara˙jo, Vera Cavalcanti | Not applicable based on title |
| Acute and delayed effects of high intensity interval resistance training organization on cortisol and testosterone production | 2016 | Di Blasio, Andrea; Izzicupo, Pascal; Tacconi, Laura; Di Santo, Serena; Leogrande, Marina; Bucci, Ines; Ripari, Patrizio; Di Baldassarre, Angela; Napolitano, Giorgio | Not applicable based on title |
| Home versus away competition: effect on psychophysiological variables in elite rugby union | 2015 | Cunniffe, Brian; Morgan, Kevin A; Baker, Julien S; Cardinale, Marco; Davies, Bruce | Not applicable based on title |
| The effects of exposure to an acute naturalistic stressor on working memory, state anxiety and salivary cortisol concentrations | 2008 | Robinson, S J; S¸nram-Lea, S I; Leach, J; Owen-Lynch, P J | Not applicable based on title |
| Long-term impact of role stress and cognitive rumination upon morning and evening saliva cortisol secretion | 2011 | Rydstedt, Leif W; Cropley, Mark; Devereux, Jason | Not applicable based on title |
| Physical and physiological demands and hormonal responses in basketball small-sided games with different tactical tasks and training regimes | 2019 | Sansone, P; Tessitore, A; Paulauskas, H; Lukonaitiene, I; Tschan, H; Pliauga, V; Conte, D | Not applicable based on title |
| Acute stress affects implicit but not explicit motor imagery: A pilot study | 2020 | Schlatter, Sophie; Guillot, Aymeric; Faes, Camille; Saruco, Elodie; Collet, Christian; Di Rienzo, Franck; Debarnot, Ursula | Not applicable based on title |
| The demand control model and circadian saliva cortisol variations in a Swedish population based sample (The PART study) | 2006 | Alderling, Magnus; Theorell, Tˆres; de la Torre, BartolomÈ; Lundberg, Ingvar | Not applicable based on title |
| Psychophysiological and cognitive effects of strawberry plants on people in isolated environments | 2005 | Li, Zhao-Ming; Liu, Hui; Zhang, Wen-Zhu; Liu, Hong | Not applicable based on title |
| Mucosal immunity and upper respiratory tract symptoms in recreational endurance runners | 2016 | Ihalainen, Johanna K; Schumann, Moritz; H‰kkinen, Keijo; Mero, Antti A | Not applicable based on title |
| The impact of acute mental stress on brachial artery flow-mediated dilation in women diagnosed with depression | 2019 | D'Urzo, Katrina A; La Rocque, Cherie L; Williams, Jennifer S; Stuckless, Troy J R; King, Trevor J; Plotnick, Meghan D; Gurd, Brendon J; Harkness, Kate L; Pyke, Kyra E | Not applicable based on title |
| Neuromuscular performance of elite rugby union players and relationships with salivary hormones | 2009 | Crewther, Blair T; Lowe, Tim; Weatherby, Robert P; Gill, Nicholas; Keogh, Justin | Not applicable based on title |
| Heart-Rate Variability in Elite Synchronized Swimmers | 2019 | Solana-Tramunt, MÚnica; Morales, Jose; Busc‡, Bernat; Carbonell, Marina; RodrÌguez-Zamora, Lara | Not applicable based on title |
| Yogic breathing when compared to attention control reduces the levels of pro-inflammatory biomarkers in saliva: a pilot randomized controlled trial | 2016 | Twal, Waleed O; Wahlquist, Amy E; Balasubramanian, Sundaravadivel | Not applicable based on title |
| Circadian variation of salivary immunoglobin A, alpha-amylase activity and mood in response to repeated double-poling sprints in hypoxia | 2016 | Born, Dennis-Peter; Faiss, Raphael; Willis, Sarah Jean; Strahler, Jana; Millet, Gregoire P; Holmberg, Hans-Christer; Sperlich, Billy | Not applicable based on title |
| Elevated activity of the sympathetic nervous system is related to diminished practice effects in memory: A pilot study | 2005 | Pagen, Linda H G; Smeets, Tom; Schmiedek, Lisa; Yassa, Michael A; Verhey, Frans R J; Jacobs, Heidi I L | Not applicable based on title |
| The impact of a competitive learning environment on hormonal and emotional stress responses and skill acquisition and expression in a medical student domain | 2019 | Cook, Christian J; Crewther, Blair T | Not applicable based on title |
| Visuo-spatial path learning, stress, and cortisol secretion following military cadets' first parachute jump: the effect of increasing task complexity | 2011 | Taverniers, John; Smeets, Tom; Lo Bue, Salvatore; Syroit, Jef; Van Ruysseveldt, Joris; Pattyn, Nathalie; von Grumbkow, Jasper | Not applicable based on title |
| Preliminary evidence that exercise dependence is associated with blunted cardiac and cortisol reactions to acute psychological stress | 2011 | Heaney, Jennifer L J; Ginty, Annie T; Carroll, Douglas; Phillips, Anna C | Not applicable based on title |
| Individual resting-state frontocingular functional connectivity predicts the intermittent theta burst stimulation response to stress in healthy female volunteers | 2020 | de Wandel, Linde; Pulopulos, Matias M; Labanauskas, Vytautas; de Witte, Sara; Vanderhasselt, Marie-Anne; Baeken, Chris | Not applicable based on title |
| Decreased cortisol response to awakening is associated with cognitive vulnerability to depression in a nonclinical sample of young adults | 2007 | Kuehner, Christine; Holzhauer, Susanne; Huffziger, Silke | Not applicable based on title |
| Mucosal immune responses during court training in elite tetraplegic athletes | 2012 | Leicht, C A; Bishop, N C; Goosey-Tolfrey, V L | Not applicable based on title |
| Effect of Cold Shower on Recovery From High-Intensity Cycling in the Heat | 2019 | Ajjimaporn, Amornpan; Chaunchaiyakul, Rungchai; Pitsamai, Sirikun; Widjaja, Waree | Not applicable based on title |
| Acute stress improves concentration performance | 2020 | Degroote, Cathy; Schwaninger, Adrian; Heimgartner, Nadja; Hedinger, Patrik; Ehlert, Ulrike; Wirtz, Petra H | Not applicable based on title |
| Effects of acute psychosocial stress exposure on endocrine and affective reactivity in college students differing in the 5-HTTLPR genotype and trait neuroticism | 2011 | Verschoor, Ellen; Markus, C Rob | Not applicable based on title |
| Effects of pilates exercise on salivary secretory immunoglobulin A levels in older women | 2005 | Hwang, Yoonyoung; Park, Jonghoon; Lim, Kiwon | Not applicable based on title |
| Extending the recovery window: Effects of trait rumination on subsequent evening cortisol following a laboratory performance stressor | 2015 | Zoccola, Peggy M; Dickerson, Sally S | Not applicable based on title |
| Different contribution of interleukin-6 and cortisol activity to total plasma fibrin concentration and to acute mental stress-induced fibrin formation | 2005 | von K‰nel, Roland; Kudielka, Brigitte M; Hanebuth, Dirk; Preckel, Daniel; Fischer, Joachim E | Not applicable based on title |
| Massage after exercise--responses of immunologic and endocrine markers: a randomized single-blind placebo-controlled study | 2009 | Arroyo-Morales, Manuel; Olea, Nicolas; RuÌz, ConcepciÛn; del Castilo, Juan de Dios Luna; MartÌnez, Manuel; Lorenzo, Carmen; DÌaz-RodrÌguez, Lourdes | Not applicable based on title |
| Prolonged performance-related neuroendocrine activation and perseverative cognition in low- and high-anxious university music students | 2018 | Gomez, Patrick; Nielsen, Carole; Studer, Regina K; Hildebrandt, Horst; Klumb, Petra L; Nater, Urs M; Wild, Pascal; Danuser, Brigitta | Not applicable based on title |
| Work stress and hair cortisol levels among workers in a Bangladeshi ready-made garment factory - Results from a cross-sectional study | 2014 | Steinisch, Maria; Yusuf, Rita; Li, Jian; Stalder, Tobias; Bosch, Jos A; Rahman, Omar; Str¸mpell, Christian; Ashraf, Hasan; Fischer, Joachim E; Loerbroks, Adrian | Not applicable based on title |
| Causal attribution and psychobiological response to competition in young men | 2017 | Salvador, Alicia; Costa, Raquel; Hidalgo, Vanesa; Gonz·lez-Bono, Esperanza | Not applicable based on title |
| Relationship between midweek training measures of testosterone and cortisol concentrations and game outcome in professional rugby union matches | 2014 | Gaviglio, Christopher M; Cook, Christian J | Included |
| Salivary cortisol levels in athletes and nonathletes: a systematic review | 2014 | Cevada, T; Vasques, P E; Moraes, H; Deslandes, A | Not applicable based on title |
| Salivary cortisol and ±-amylase responses to repeated bouts of downhill running | 2014 | Mckune, Andrew J; Bach, Christopher W; Semple, Stuart J; Dyer, Barry J | Not applicable based on title |
| Associations between digit ratio (2D4D), mood, and autonomic stress response in healthy men | 2019 | Walther, A; Breidenstein, J; Bˆsch, M; Sefidan, S; Ehlert, U; Annen, H; Wyss, T; La Marca, R | Not applicable based on title |
| Psychophysiological relationships between a multi-component self-report measure of mood, stress and behavioural signs and symptoms, and physiological stress responses during a simulated firefighting deployment | 2016 | Wolkow, Alexander; Aisbett, Brad; Ferguson, Sally A; Reynolds, John; Main, Luana C | Not applicable based on title |
| The stress of competition dissociates neural and cortisol homeostasis in elite athletes | 2003 | Iellamo, F; Pigozzi, F; Parisi, A; Di Salvo, V; Vago, T; Norbiato, G; Lucini, D; Pagani, M | Not applicable based on title |
| Effect of acute nitrate supplementation on neurovascular coupling and cognitive performance in hypoxia | 2016 | Lefferts, Wesley K; Hughes, William E; White, Corey N; Brutsaert, Tom D; Heffernan, Kevin S | Not applicable based on title |
| A Functional Near-Infrared Spectroscopy Study on the Cortical Haemodynamic Responses During the Maastricht Acute Stress Test | 2019 | Schaal, N K; Hepp, P; Schweda, A; Wolf, O T; Krampe, C | Not applicable based on title |
| Case Study: Nutritional and Lifestyle Support to Reduce Infection Incidence in an International-Standard Premier League Soccer Player | 2016 | Ranchordas, Mayur K; Bannock, Laurent; Robinson, Scott L | Not applicable based on title |
| The impact of sport related stressors on immunity and illness risk in team-sport athletes | 2018 | Keaney, Lauren C; Kilding, Andrew E; Merien, Fabrice; Dulson, Deborah K | Not applicable based on title |
| Salivary cytokine response in the aftermath of stress: An emotion regulation perspective | 2017 | Newton, Tamara L; Fernandez-Botran, Rafael; Lyle, Keith B; Szabo, Yvette Z; Miller, James J; Warnecke, Ashlee J | Not applicable based on title |
| Successful voluntary recruitment of cognitive control under acute stress | 2017 | Plessow, Franziska; Schade, Susann; Kirschbaum, Clemens; Fischer, Rico | Not applicable based on title |
| Salivary steroids hormones, well-being, and physical performance during an intensification training period followed by a tapering period in youth rhythmic gymnasts | 2017 | Antualpa, Kizzy; Aoki, Marcelo Saldanha; Moreira, Alexandre | Not applicable based on title |
| Effect of naproxen on the hypothalamic-pituitary-adrenal axis in healthy volunteers | 2009 | Eijsbouts, Agnes M M; Kempers, Marlies J E; Kramer, Renske S A; Hopman, Maria T E; van den Hoogen, Frank H J; Laan, Ronald F J M; Hermus, Ad R M M; Sweep, Fred C G J; van de Putte, Leo B A | Not applicable based on title |
| Skill acquisition and stress adaptations following laparoscopic surgery training and detraining in novice surgeons | 2016 | Crewther, Blair T; Shetty, Kunal; Jarchi, Delaram; Selvadurai, Shaun; Cook, Christian J; Leff, Daniel R; Darzi, Ara; Yang, Guang-Zhong | Not applicable based on title |
| Acute Physiological Responses to an Intensity-And Time-Under-Tension-Equated Single- vs Multiple-Set Resistance Training Bout in Trained Men | 2018 | Cintineo, Harry P; Freidenreich, Daniel J; Blaine, Caitlin M; Cardaci, Thomas D; Pellegrino, Joseph K; Arent, Shawn M | Not applicable based on title |
| Hyperactivity of the Sympatho-Adrenomedullary System Without Any Modification of the Hypothalamic-Pituitary-Adrenal Axis After Food Restriction Among High-Level Weightlifters | 2018 | Durguerian, Alexandre; Filaire, Edith; Drogou, Catherine; Sauvet, Fabien; Bougard, ClÈment; Chennaoui, Mounir | Not applicable based on title |
| The effect of antecedent exercise on the acute stress response and subsequent food consumption: a preliminary investigation | 2021 | Leow, Shina; Beer, Natalya J; Dimmock, James A; Jackson, Ben; Alderson, Jacqueline A; Clarke, Michael W; Guelfi, Kym J | Not applicable based on title |
| Patterns of cortisol reactivity to laboratory stress | 2004 | Roy, Mark P | Not applicable based on title |
| Triathletes Lose Their Advantageous Pain Modulation under Acute Psychosocial Stress | 2017 | Geva, Nirit; Pruessner, Jens; Defrin, Ruth | Not applicable based on title |
| A Multi-Factorial Assessment of Elite Paratriathletes' Response to Two Weeks of Intensified Training | 2019 | Stephenson, Ben T; Leicht, Christof A; Tolfrey, Keith; Goosey-Tolfrey, Victoria L | Not applicable based on title |
| A single bout of hard RPE-based cycling exercise increases salivary alpha-amylase | 2019 | Weiss, Lauren R; Venezia, Andrew C; Smith, J Carson | Not applicable based on title |
| Effects of protein versus carbohydrate supplementation on markers of immune response in master triathletes: A randomized controlled trial | 2019 | Naclerio, Fernando; Larumbe-Zabala, Eneko; Seijo, Marcos; Ashrafi, Nadia; Nielsen, Birthe V; Earnest, Conrad P | Not applicable based on title |
| Salivary chromogranin-A as a marker of psychological stress during a cognitive test battery in humans | 2006 | Kanamaru, Yoshiki; Kikukawa, Azusa; Shimamura, Keiichi | Not applicable based on title |
| Modulation of neuroendocrine response and non-verbal behavior during psychosocial stress in healthy volunteers by the glutamate release-inhibiting drug lamotrigine | 2004 | Makatsori, Aikaterini; Duncko, Roman; Moncek, Fedor; Loder, Ingrid; Katina, Stanislav; Jezova, Daniela | Not applicable based on title |
| Performance indicators during international rugby union matches are influenced by a combination of physiological and contextual variables | 2020 | Crewther, Blair T; Potts, Neill; Kilduff, Liam P; Drawer, Scott; Cook, Christian J | Included |
| Altered attentional control strategies but spared executive functioning in chronic cannabis users | 2017 | Nusbaum, Amy T; Whitney, Paul; Cuttler, Carrie; Spradlin, Alexander; Hinson, John M; McLaughlin, Ryan J | Not applicable based on title |
| The catecholamine neurotransmitter precursor tyrosine increases anger during exposure to severe psychological stress | 2015 | Lieberman, Harris R; Thompson, Lauren A; Caruso, Christina M; Niro, Philip J; Mahoney, Caroline R; McClung, James P; Caron, Gregory R | Not applicable based on title |
| Prenatal maternal distress seems to be associated with the infant's temperament and motor development: an explorative study | 2017 | Haselbeck, Christin; Niederberger, Uwe; Kulle, Alexandra; Wache, Katharina; Brauner, Ellen; Gutermuth, Merlin; Holterhus, Paul-Martin; Gerber, Wolf-Dieter; Siniatchkin, Michael | Not applicable based on title |
| Effects of acute exercise on salivary free insulin-like growth factor 1 and interleukin 10 in sportsmen | 2016 | Lasisi, Taye J; Adeniyi, Ade F | Not applicable based on title |
| Effects of chronic khat use on cardiovascular, adrenocortical, and psychological responses to stress in men and women | 2013 | al'Absi, Mustafa; Khalil, Najat Sayem; Al Habori, Molham; Hoffman, Richard; Fujiwara, Koji; Wittmers, Lorentz | Not applicable based on title |
| Inhibitory control failures and blunted cortisol response to psychosocial stress in amphetamine consumers after 6 months of abstinence | 2019 | Benitez-LÛpez, Yermein; Redolar-Ripoll, Diego; Ruvalcaba-Delgadillo, Yaveth; J·uregui-Huerta, Fernando | Not applicable based on title |
| Heart rate variability, salivary cortisol and competitive state anxiety responses during pre-competition and pre-training moments | 2019 | Souza, Renato A; Beltran, Oscar A B; Zapata, Diana M; Silva, Elis‚ngela; Freitas, Wagner Z; Junior, Rubens V; da Silva, Fabiano F; Higino, Wonder P | Not applicable based on title |
| Immunoendocrine responses of male spinal cord injured athletes to 1-hour self-paced exercise: pilot study | 2005 | Allgrove, Judith E; Chapman, Mark; Christides, Tatiana; Smith, Paul M | Not applicable based on title |
| Acute exercise-induced enhancement of fear inhibition is moderated by BDNF Val66Met polymorphism | 2019 | Keyan, Dharani; Bryant, Richard A | Not applicable based on title |
| Variability in nitrate-reducing oral bacteria and nitric oxide metabolites in biological fluids following dietary nitrate administration: An assessment of the critical difference | 2019 | Liddle, Luke; Burleigh, Mia C; Monaghan, Chris; Muggeridge, David J; Sculthorpe, Nicholas; Pedlar, Charles R; Butcher, John; Henriquez, Fiona L; Easton, Chris | Not applicable based on title |
| High-Dose Probiotic Supplementation Containing Lactobacillus casei for 7 Days Does Not Enhance Salivary Antimicrobial Protein Responses to Exertional Heat Stress Compared With Placebo | 2016 | Gill, Samantha Kirsty; Teixeira, Ana Maria; Rosado, Fatima; Cox, Martin; Costa, Ricardo Jose | Not applicable based on title |
| Fitness level impacts salivary antimicrobial protein responses to a single bout of cycling exercise | 2015 | Kunz, Hawley; Bishop, Nicolette C; Spielmann, Guillaume; Pistillo, Mira; Reed, Justin; Ograjsek, Teja; Park, Yoonjung; Mehta, Satish K; Pierson, Duane L; Simpson, Richard J | Not applicable based on title |
| Acute high-intensity exercise test in soccer athletes affects salivary biochemical markers | 2018 | Rodrigues de Araujo, VinÌcius; Lisboa, PatrÌcia; Boaventura, Gabriel; Caramez, Fabiele; Pires, Luciane; Oliveira, Elaine; Moura, Egberto; Casimiro-Lopes, Gustavo | Not applicable based on title |
| The effect of the visual exercise environment on the response to psychological stress: a pilot study | 2020 | Wood, Carly; Flynn, M; Law, R; Naufahu, J; Smyth, N | Not applicable based on title |
| Attentional bias modification in reducing test anxiety vulnerability: a randomized controlled trial | 2018 | Cai, Wenpeng; Pan, Yu; Chai, Huangyangzi; Cui, Yi; Yan, Jin; Dong, Wei; Deng, Guanghui | Not applicable based on title |
| Enhanced emotional empathy after psychosocial stress in young healthy men | 2015 | Wolf, Oliver T; Schulte, Judith M; Drimalla, Hanna; Hamacher-Dang, Tanja C; Knoch, Daria; Dziobek, Isabel | Not applicable based on title |
| The Acute Effects of Different Forms of Suspension Push-Ups on Oxygen Consumption, Salivary Testosterone and Cortisol and Isometric Strength | 2018 | Bellar, David; Etheredge, Cory; Judge, Lawrence W | No acute stressor |
| Positive adaptation of HPA axis function in women during 44 weeks of infantry-based military training | 2019 | Gifford, R M; O'Leary, T J; Double, R L; Wardle, S L; Wilson, K; Boyle, L D; Homer, N Z M; Kirschbaum, C; Greeves, J P; Woods, D R; Reynolds, R M | Not applicable based on title |
| Stress-induced cytokine responses and central adiposity in young women | 2008 | Brydon, L; Wright, C E; O'Donnell, K; Zachary, I; Wardle, J; Steptoe, A | Not applicable based on title |
| Monitoring internal training load and salivary immune-endocrine responses during an annual judo training periodization | 2017 | Agostinho, Marcus F; Moreira, Alexandre; Julio, Ursula F; Marcolino, Gilvan S; Antunes, Barbara M M; Lira, Fabio S; Franchini, Emerson | Not applicable based on title |
| Reducing racial/ethnic tobacco cessation disparities via cognitive behavioral therapy: Design of a dualsite randomized controlled trial | 2018 | Webb Hooper, Monica; Lee, David J; Simmons, Vani N; Brandon, Karen O; Antoni, Michael H; Unrod, Marina; Asfar, Taghrid; Correa, John B; Koru-Sengul, Tulay; Brandon, Thomas H | Not applicable based on title |
| The influence of a motivational climate intervention on participants' salivary cortisol and psychological responses | 2013 | Hogue, Candace M; Fry, Mary D; Fry, Andrew C; Pressman, Sarah D | Not applicable based on title |
| The workout responses of salivary-free testosterone and cortisol concentrations and their association with the subsequent competition outcomes in professional rugby league | 2013 | Crewther, Blair T; Sanctuary, Colin E; Kilduff, Liam P; Carruthers, Jamie S; Gaviglio, Chris M; Cook, Christian J | Included |
| Salivary cortisol and testosterone responses to high-intensity cycling before and after an 11-day intensified training period | 2013 | Hough, John; Corney, Robert; Kouris, Antonios; Gleeson, Michael | Not applicable based on title |
| Do waking salivary cortisol levels correlate with anesthesiologist's job involvement? | 2012 | Klein, Moti; Weksler, Natan; Gidron, Yori; Heldman, Eliyahu; Gurski, Eugen; Smith, Otto Robert F; Gurman, Gabriel M | Not applicable based on title |
| The combined influence of fat consumption and repeated mental stress on brachial artery flow-mediated dilatation: a preliminary study | 2014 | Poitras, Veronica J; Slattery, David J; Levac, Brendan M; Fergus, Stevenson; Gurd, Brendon J; Pyke, Kyra E | Not applicable based on title |
| Prolonged sleep deprivation and continuous exercise: effects on melatonin, tympanic temperature, and cognitive function | 2014 | Davis, Greggory R; Etheredge, Corey E; Marcus, Lena; Bellar, David | Not applicable based on title |
| Stress induced cortisol release and schizotypy | 2018 | Walter, Emma E; Fernandez, Francesca; Snelling, Mollie; Barkus, Emma | Not applicable based on title |
| Effects of paraprobiotic Lactobacillus paracasei MCC1849 supplementation on symptoms of the common cold and mood states in healthy adults | 2018 | Murata, M; Kondo, J; Iwabuchi, N; Takahashi, S; Yamauchi, K; Abe, F; Miura, K | Not applicable based on title |
| Saliva as a non-invasive tool for monitoring oxidative stress in swimmers athletes performing a VO2max cycle ergometer test | 2020 | Biagini, Denise; Lomonaco, Tommaso; Ghimenti, Silvia; Fusi, Jonathan; Cerri, Eugenio; De Angelis, Francesca; Bellagambi, Francesca Giuseppa; Oger, Camille; Galano, Jean Marie; Bramanti, Emilia; Franzoni, Ferdinando; Fuoco, Roger; Di Francesco, Fabio | Not applicable based on title |
| Effects of CYP1A2 and ADORA2A genotypes on the ergogenic response to caffeine in professional handball players | 2020 | MuÒoz, Alejandro; LÛpez-Samanes, ¡lvaro; Aguilar-Navarro, Mill·n; Varillas-Delgado, David; Rivilla-GarcÌa, Jes˙s; Moreno-PÈrez, VÌctor; Del Coso, Juan | Not applicable based on title |
| Prevalence and risk indicators of non-carious cervical lesions in male footballers | 2020 | Medeiros, Tamea Lacerda Monteiro; Mutran, Sheila Cristina Almeida Neves; Espinosa, Daybelis Gonz·lez; do Carmo Freitas Faial, Kelson; Pinheiro, Helder Henrique Costa; D'Almeida Couto, Roberta Souza | Not applicable based on title |
| Impact of Experimentally Induced Cognitive Dietary Restraint on Eating Behavior Traits, Appetite Sensations, and Markers of Stress during Energy Restriction in Overweight/Obese Women | 2018 | Morin, Isabelle; BÈgin, Catherine; Maltais-GiguËre, Julie; BÈdard, Alexandra; Tchernof, AndrÈ; Lemieux, Simone | Not applicable based on title |
| Variation of salivary iga during weight loss period before a competition among university judo players | 2019 | Hiraoka, Hiroaki; Hanaoka, Yukichi; Jesmin, Subrina; Kimura, Fuminori; Matsuish, Yujiro; Shimizu, Kazuhiro; Watanabe, Koichi | Not applicable based on title |
| Changes in Stress and Appetite Responses in Male Power-Trained Athletes during Intensive Training Camp | 2017 | Oshima, Satomi; Takehata, Chisato; Sasahara, Ikuko; Lee, Eunjae; Akama, Takao; Taguchi, Motoko | Not applicable based on title |
| Stress lowers the detection threshold for foul-smelling 2-mercaptoethanol | 2005 | Pacharra, Marlene; Sch‰per, Michael; Kleinbeck, Stefan; Blaszkewicz, Meinolf; Wolf, Oliver T; van Thriel, Christoph | Not applicable based on title |
| Emotions, immunity and sport: Winner and loser athlete's profile of fighting sport | 2015 | Pesce, Mirko; Fratta, Irene La; Ialenti, Valentina; Patruno, Antonia; Ferrone, Alessio; Franceschelli, Sara; Rizzuto, Alessia; Tatangelo, Raffaella; Campagna, Giovanna; Speranza, Lorenza; Felaco, Mario; Grilli, Alfredo | Not applicable based on title |
| The influence of 10 min of the Johrei healing method on laboratory stress | 2006 | Laidlaw, Tannis M; Naito, Akira; Dwivedi, Prabudha; Hansi, Navjyot K; Henderson, Don C; Gruzelier, John H | Not applicable based on title |
| The cortisol response to hypobaric hypoxia at rest and post-exercise | 2012 | Woods, D R; Davison, A; Stacey, M; Smith, C; Hooper, T; Neely, D; Turner, S; Peaston, R; Mellor, A | Included |
| Acute short-term mental stress does not influence salivary flow rate dynamics | 2012 | Naumova, Ella A; Sandulescu, Tudor; Al Khatib, Philipp; Thie, Michael; Lee, Wing-Kee; Zimmer, Stefan; Arnold, Wolfgang H | Not applicable based on title |
| Modifying a negative interpretation bias for ambiguous social scenarios that depict the risk of rejection in women with anorexia nervosa | 2018 | Turton, Robert; Cardi, Valentina; Treasure, Janet; Hirsch, Colette R | Not applicable based on title |
| Cookie or clementine? Psychophysiological stress reactivity and recovery after eating healthy and unhealthy comfort foods | 2019 | Finch, Laura E; Cummings, Jenna R; Tomiyama, A Janet | Not applicable based on title |
| Habitual and acute exercise effects on salivary biomarkers in response to psychosocial stress | 2019 | Wunsch, Kathrin; Wurst, Ramona; von Dawans, Bernadette; Strahler, Jana; Kasten, Nadine; Fuchs, Reinhard | Not applicable based on title |
| Stress hormone levels in saliva after shogi competition are modified by stress coping strategies | 2011 | Hasegawa-Ohira, Masako; Toda, Masahiro; Morimoto, Kanehisa | Not applicable based on title |
| Changes in creatine kinase, lactate dehydrogenase and aspartate aminotransferase in saliva samples after an intense exercise: a pilot study | 2018 | Barranco, Tomas; Tvarijonaviciute, Asta; Tecles, Fernando; Carrillo, Jose M; S·nchez-Resalt, Cristina; Jimenez-Reyes, Pedro; Rubio, Monica; GarcÌa-BalletbÛ, Monserrat; CerÛn, Jose J; Cugat, Ramon | Not applicable based on title |
| Frequency of bullying at work, physiological response, and mental health | 2011 | Hansen, ≈se Marie; Hogh, Annie; Persson, Roger | Not applicable based on title |
| Psychometric and neurobiological assessment of resilience in a non-clinical sample of adults | 2013 | Petros, Natalia; Opacka-Juffry, Jolanta; Huber, Jˆrg H | Not applicable based on title |
| Essential Oil of Japanese Cedar (Cryptomeria japonica) Wood Increases Salivary Dehydroepiandrosterone Sulfate Levels after Monotonous Work | 2017 | Matsubara, Eri; Tsunetsugu, Yuko; Ohira, Tatsuro; Sugiyama, Masaki | Not applicable based on title |
| Immediate and long-term effects of meditation on acute stress reactivity, cognitive functions, and intelligence | 2012 | Singh, Yogesh; Sharma, Ratna; Talwar, Anjana | Not applicable based on title |
| Preconditioning Strategy in Rugby-7s Players: Beneficial or Detrimental? | 2019 | Marrier, Bruno; Durguerian, Alexandre; Robineau, Julien; Chennaoui, Mounir; Sauvet, Fabien; Servonnet, AurÈlie; Piscione, Julien; Mathieu, Bertrand; Peeters, Alexis; Lacome, Mathieu; Morin, Jean-Benoit; Le Meur, Yann | Not applicable based on title |
| The role played by the interaction between genetic factors and attachment in the stress response in infancy | 2009 | Frigerio, Alessandra; Ceppi, Elisa; Rusconi, Marianna; Giorda, Roberto; Raggi, Maria Elisabetta; Fearon, Pasco | Not applicable based on title |
| Effects of prior light exposure on early evening performance, subjective sleepiness, and hormonal secretion | 2012 | M¸nch, Mirjam; Linhart, Friedrich; Borisuit, Apiparn; Jaeggi, Susanne M; Scartezzini, Jean-Louis | Not applicable based on title |
| Social constraints, genetic vulnerability, and mental health following collective stress | 2011 | Holman, E Alison; Lucas-Thompson, Rachel G; Lu, Tammy | Not applicable based on title |
| Fluid Restriction Decreases Solid Food Consumption Post-Exercise | 2019 | PÈrez-Luco, Cristian; DÌaz-Castro, Francisco; Jorquera, Carlos; Troncoso, Rodrigo; Zbinden-Foncea, Hermann; Johannsen, Neil M; Castro-Sepulveda, Mauricio | Not applicable based on title |
| Salivary cortisol increases after bariatric surgery in women | 2011 | Valentine, A R; Raff, H; Liu, H; Ballesteros, M; Rose, J M; Jossart, G H; Cirangle, P; Bravata, D M | Not applicable based on title |
| Assisting couples to develop healthy relationships: effects of couples relationship education on cortisol | 2011 | Ditzen, Beate; Hahlweg, Kurt; Fehm-Wolfsdorf, Gabriele; Baucom, Don | Not applicable based on title |
| Pre-experience of social exclusion suppresses cortisol response to psychosocial stress in women but not in men | 2010 | Weik, Ulrike; Maroof, Patrick; Zˆller, C‰cilia; Deinzer, Renate | Not applicable based on title |
| Morning based strength training improves afternoon physical performance in rugby union players | 2014 | Cook, Christian J; Kilduff, Liam P; Crewther, Blair T; Beaven, Martyn; West, Daniel J | Not applicable based on title |
| Influence of chewing time on salivary stress markers | 2014 | Tasaka, Akinori; Takeuchi, Kai; Sasaki, Hiromitsu; Yoshii, Takayuki; Soeda, Ryohei; Ueda, Takayuki; Sakurai, Kaoru | Not applicable based on title |
| A pilot evaluation on a stress management programme using a combined approach of cognitive behavioural therapy (CBT) and complementary and alternative medicine (CAM) for elementary school teachers | 2015 | Tsang, Hector W H; Cheung, W M; Chan, Alan H L; Fung, Kelvin M T; Leung, Ada Y; Au, Doreen W H | Not applicable based on title |
| Association of methamphetamine use and restrictive interventions in an acute adult inpatient mental health unit: A retrospective cohort study | 2017 | McKenna, Brian; McEvedy, Samantha; Kelly, Kathleen; Long, Bec; Anderson, Jess; Dalzell, Elaine; Maguire, Tessa; Tacey, Mark; Furness, Trentham | Not applicable based on title |
| Hormonal responses to physical and cognitive stress in a school setting | 2010 | Budde, Henning; Pietrassyk-Kendziorra, Sascha; Bohm, Sebastian; Voelcker-Rehage, Claudia | Not applicable based on title |
| Effect of acute exercise on the levels of salivary cortisol, tumor necrosis factor-alpha and nitric oxide | 2010 | Rahman, Zamzy A; Abdullah, Nizam; Singh, Rabindarjeet; Sosroseno, Wihaskoro | Not applicable based on title |
| Effect of hypoxia on cerebrovascular and cognitive function during moderate intensity exercise | 2016 | Lefferts, Wesley K; Babcock, Matthew C; Tiss, Matthew J; Ives, Stephen J; White, Corey N; Brutsaert, Tom D; Heffernan, Kevin S | Not applicable based on title |
| Clinical EFT (emotional freedom techniques) improves multiple physiological markers of health | 2005 | Bach, Donna; Groesbeck, Gary; Stapleton, Peta; Sims, Rebecca; Blickheuser, Katharina; Church, Dawson | Not applicable based on title |
| Effects of carbohydrate beverage ingestion on the salivary IgA response to intermittent exercise in the heat | 2011 | Sari-Sarraf, V; Doran, D A; Clarke, N D; Atkinson, G; Reilly, T | Not applicable based on title |
| Dose effect of caffeine on testosterone and cortisol responses to resistance exercise | 2008 | Beaven, C Martin; Hopkins, Will G; Hansen, Kier T; Wood, Mathew R; Cronin, John B; Lowe, Timothy E | Not applicable based on title |
| Salivary testosterone and cortisol responses in professional rugby players after four resistance exercise protocols | 2008 | Beaven, C Martyn; Gill, Nicholas D; Cook, Christian J | Not applicable based on title |
| The salivary testosterone and cortisol response to three loading schemes | 2008 | Crewther, Blair; Cronin, John; Keogh, Justin; Cook, Christian | Not applicable based on title |
| Anti-Stress, Behavioural and Magnetoencephalography Effects of an L-Theanine-Based Nutrient Drink: A Randomised, Double-Blind, Placebo-Controlled, Crossover Trial | 2016 | White, David J; de Klerk, Suzanne; Woods, William; Gondalia, Shakuntla; Noonan, Chris; Scholey, Andrew B | Not applicable based on title |
| Variations in anticipatory cognitive stress appraisal and differential proinflammatory cytokine expression in response to acute stress | 2007 | Wirtz, Petra H; von K‰nel, Roland; Emini, Luljeta; Suter, Tobias; Fontana, Adriano; Ehlert, Ulrike | Not applicable based on title |
| Neuromuscular and hormonal responses to a single session of whole body vibration exercise in healthy young men | 2007 | Erskine, Julie; Smillie, Ian; Leiper, John; Ball, Derek; Cardinale, Marco | Included |
| Motivation, stress, anxiety, and cortisol responses in elite paragliders | 2007 | Filaire, Edith; Alix, Deborah; Rouveix, Matthieu; Le Scanff, Christine | Not applicable based on title |
| Cortisol and stress responses during a game and practice in female collegiate soccer players | 2007 | Haneishi, Kanae; Fry, Andrew C; Moore, Christopher A; Schilling, Brian K; Li, Yuhua; Fry, Mary D | Not applicable based on title |
| Effects of intraoperative breaks on mental and somatic operator fatigue: a randomized clinical trial | 2011 | Engelmann, Carsten; Schneider, Mischa; Kirschbaum, Clemens; Grote, Gudela; Dingemann, Jens; Schoof, Stefan; Ure, Benno M | Not applicable based on title |
| Effects of glucocorticoid and noradrenergic activity on spatial learning and spatial memory in healthy young adults | 2019 | Chae, Woo Ri; Metz, Sophie; Weise, Jeanette; Nowacki, Jan; Piber, Dominique; Mueller, Sven C; Wingenfeld, Katja; Otte, Christian | Not applicable based on title |
| The Effects of a 36-Hour Mixed Task Ultraendurance Race on Mucosal Immunity Markers and Pulmonary Function | 2017 | Bellar, David; Murphy, Kellie A; Aithal, Ritvik; Davis, Greggory R; Piper, Tim | Included |
| Relationships among training stress, mood and dehydroepiandrosterone sulphate/cortisol ratio in female cyclists | 2006 | Bouget, MikaÎl; Rouveix, Mathieu; Michaux, Odile; Pequignot, Jean-Marc; Filaire, Edith | Not applicable based on title |
| Long-Haul Northeast Travel Disrupts Sleep and Induces Perceived Fatigue in Endurance Athletes | 2018 | Stevens, Christopher J; Thornton, Heidi R; Fowler, Peter M; Esh, Christopher; Taylor, Lee | Not applicable based on title |
| Post-awakening cortisol secretion during basic military training | 2006 | Clow, A; Edwards, S; Owen, G; Evans, G; Evans, P; Hucklebridge, F; Casey, A | Not applicable based on title |
| Tobacco usage interacts with postdisaster psychopathology on circadian salivary cortisol | 2006 | Olff, Miranda; Meewisse, Marie-Louise; Kleber, Rolf J; van der Velden, Peter G; Drogendijk, Annelieke N; van Amsterdam, Jan G C; Opperhuizen, Antoon; Gersons, Berthold P R | Not applicable based on title |
| Influence of the way of reporting alpha-Amylase values in saliva in different naturalistic situations: A pilot study | 2017 | Contreras-Aguilar, MarÌa Dolores; Escribano, Dami·n; MartÌnez-Subiela, Silvia; MartÌnez-MirÛ, Silvia; Rubio, MÛnica; Tvarijonaviciute, Asta; Tecles, Fernando; CerÛn, Jose J | Not applicable based on title |
| Effects of high-intensity isokinetic exercise on salivary cortisol in athletes with different training schedules: relationships to serum cortisol and lactate | 2005 | Paccotti, P; Minetto, M; Terzolo, M; Ventura, M; Ganzit, G P; Borrione, P; Termine, A; Angeli, A | Not applicable based on title |
| The relationship between salivary biomarkers and state-trait anxiety inventory score under mental arithmetic stress: a pilot study | 2005 | Noto, Yuka; Sato, Tetsumi; Kudo, Mihoko; Kurata, Kiyoshi; Hirota, Kazuyoshi | Not applicable based on title |
| The influence of perceived control and locus of control on the cortisol and subjective responses to stress | 2004 | Bollini, Annie M; Walker, Elaine F; Hamann, Stephan; Kestler, Lisa | Not applicable based on title |
| Immune and hormonal changes following intense military training | 2003 | Gomez-Merino, Danielle; Chennaoui, Mounir; Burnat, Pascal; Drogou, Catherine; Guezennec, Charles Yannick | Included |
| Effects of chronic academic stress on mental state and expression of glucocorticoid receptor ± and ≤ isoforms in healthy Japanese medical students | 2011 | Kurokawa, Ken; Tanahashi, Toshihito; Murata, Akiho; Akaike, Yoko; Katsuura, Sakurako; Nishida, Kensei; Masuda, Kiyoshi; Kuwano, Yuki; Kawai, Tomoko; Rokutan, Kazuhito | Not applicable based on title |
| High-Intensity Training and Salivary Immunoglobulin A Responses in Professional Top-Level Soccer Players: Effect of Training Intensity | 2016 | Owen, Adam L; Wong, Del P; Dunlop, Gordon; Groussard, Carole; Kebsi, Wiem; Dellal, Alexandre; Morgans, Ryland; Zouhal, Hassane | Not applicable based on title |
| Neurobehavioral effects of exposure to propionic acid revisited-Does psychosocial stress interfere with distractive effects in volunteers? | 2016 | Pacharra, Marlene; Sch‰per, Michael; Kleinbeck, Stefan; Blaszkewicz, Meinolf; Golka, Klaus; van Thriel, Christoph | Not applicable based on title |
| The man I once knew: grief and inflammation in female partners of veterans with traumatic brain injury | 2016 | Saban, Karen L; Mathews, Herbert L; Collins, Eileen G; Hogan, Nancy S; Tell, Dina; Bryant, Fred B; Pape, Theresa Louise Bender; Griffin, Joan M; Janusek, Linda Witek | Not applicable based on title |
| Age at first intercourse is inversely related to female cortisol stress reactivity | 2002 | Brody, Stuart | Not applicable based on title |
| Cortisol, DHEA, performance and training in elite swimmers | 2002 | Chatard, J-C; Atlaoui, D; Lac, G; Duclos, M; Hooper, S; Mackinnon, L | Not applicable based on title |
| Relationship between serum, saliva and urinary cortisol and its implication during recovery from training | 2002 | Neary, J P; Malbon, L; McKenzie, D C | Not applicable based on title |
| Psychosocial stress affects the acquisition of cerebellar-dependent sensorimotor adaptation | 2018 | Gheorghe, Delia A; PanouillËres, Muriel T N; Walsh, Nicholas D | Not applicable based on title |
| Examining stress: an investigation of stress, mood and exercise in medical students | 2005 | O'Flynn, J; Dinan, T G; Kelly, J R | Not applicable based on title |
| Effects of chewing gum on cognitive function, mood and physiology in stressed and non-stressed volunteers | 2010 | Smith, Andrew | Not applicable based on title |
| Salivary antimicrobial peptides (LL-37 and alpha-defensins HNP1-3), antimicrobial and IgA responses to prolonged exercise | 2009 | Davison, Glen; Allgrove, Judith; Gleeson, Michael | Not applicable based on title |
| Influence of chewing force on salivary stress markers as indicator of mental stress | 2012 | Soeda, R; Tasaka, A; Sakurai, K | Not applicable based on title |
| The cortisol awakening response and resilience in elite swimmers | 2016 | Meggs, J; Golby, J; Mallett, C J; Gucciardi, D F; Polman, R C J | Not applicable based on title |
| Salivary estradiol, interleukin-6 production, and the relationship to substrate metabolism during exercise in females | 2011 | Ives, Stephen J; Blegen, Mark; Coughlin, Mary A; Redmond, Jan; Matthews, Tracey; Paolone, Vincent | Not applicable based on title |
| Comparison of pain, cortisol levels, and psychological distress in women undergoing surgical termination of pregnancy under local anaesthesia versus intravenous sedation | 2007 | Suliman, Sharain; Ericksen, Todd; Labuschgne, Peter; de Wit, Renee; Stein, Dan J; Seedat, Soraya | Not applicable based on title |
| Effects of acute psychological stress on athletic performance in elite male swimmers | 2019 | Rano, Jacqueline; FridÈn, Cecilia; Eek, Frida | Not applicable based on title |
| Circadian phenotype impacts the brain's resting-state functional connectivity, attentional performance, and sleepiness | 2019 | Facer-Childs, Elise R; Campos, Brunno M; Middleton, Benita; Skene, Debra J; Bagshaw, Andrew P | Not applicable based on title |
| Hand-held Colorimetry Sensor Platform for Determining Salivary ±-Amylase Activity and Its Applications for Stress Assessment | 2019 | Hsiao, Hsien-Yi; Chen, Richie L C; Chou, Chih-Chi; Cheng, Tzong-Jih | Not applicable based on title |
| Caffeine and stress alter salivary alpha-amylase activity in young men | 2010 | Klein, Laura C; Bennett, Jeanette M; Whetzel, Courtney A; Granger, Douglas A; Ritter, Frank E | Not applicable based on title |
| The protective impact of a mental skills training session and motivational priming on participantspsychophysiological responses to performance stress | 2019 | Hogue, Candace M. | Not applicable based on title |
| No effect of a 30-h period of sleep deprivation on leukocyte trafficking, neutrophil degranulation and saliva IgA responses to exercise | 2009 | Ricardo, J S Costa; Cartner, Louise; Oliver, Samuel J; Laing, Stewart J; Walters, Robert; Bilzon, James L J; Walsh, Neil P | age |
| The influence of hydration status during prolonged endurance exercise on salivary antimicrobial proteins | 2015 | Killer, Sophie C; Svendsen, Ida S; Gleeson, Michael | Not applicable based on title |
| Performance evaluation of a salivary amylase biosensor for stress assessment in military field research | 2016 | Peng, Henry T; Savage, Erin; Vartanian, Oshin; Smith, Shane; Rhind, Shawn G; Tenn, Catherine; Bjamason, Stephen | Not applicable based on title |
| Salivary testosterone and cortisol responses to four different rugby training exercise protocols | 2015 | Gaviglio, Christopher M; Osborne, Mark; Kelly, Vincent G; Kilduff, Liam P; Cook, Christian J | Not applicable based on title |
| Stress intensifies demands on response selection during action cascading processes | 2014 | Yildiz, Ali; Wolf, Oliver T; Beste, Christian | Not applicable based on title |
| Short-term effects of a randomized controlled worksite relaxation intervention in Greece | 2005 | Alexopoulos, Evangelos C; Zisi, Marilena; Manola, Georgia; Darviri, Christina | Not applicable based on title |
| The relationship between shift work, sleep, and cognition in career emergency physicians | 2012 | Machi, Mari S; Staum, Matthew; Callaway, Clifton W; Moore, Charity; Jeong, Kwonho; Suyama, Joe; Patterson, P Daniel; Hostler, David | Included |
| Immediate Effects of Traditional Thai Massage on Psychological Stress as Indicated by Salivary Alpha-Amylase Levels in Healthy Persons | 2015 | Sripongngam, Thanarat; Eungpinichpong, Wichai; Sirivongs, Dhavee; Kanpittaya, Jaturat; Tangvoraphonkchai, Kamonwan; Chanaboon, Sutin | Not applicable based on title |
| Association between coffee consumption and markers of inflammation and cardiovascular function during mental stress | 2006 | Hamer, Mark; Williams, Emily D; Vuononvirta, Raisa; Gibson, E Leigh; Steptoe, Andrew | Not applicable based on title |
| Acute consolidation stress enhances reality monitoring in healthy young adults | 2008 | Smeets, T; Sijstermans, K; Gijsen, C; Peters, M; Jelicic, M; Merckelbach, H | Not applicable based on title |
| Effects of competition, exercise, and mental stress on secretory immunity | 2005 | Ring, Christopher; Carroll, Douglas; Hoving, Johan; Ormerod, John; Harrison, Lesley K; Drayson, Mark | Not applicable based on title |
| Quantitative Assessment for Self-Tracking of Acute Stress Based on Triangulation Principle in a Wearable Sensor System | 2019 | Wu, Wanqing; Pirbhulal, Sandeep; Zhang, Heye; Mukhopadhyay, Subhas Chandra | Not applicable based on title |
| Glucocorticoid response to stress induction prior to learning is negatively related to subsequent motor memory consolidation | 2019 | Dolfen, Nina; King, Bradley R; Schwabe, Lars; Swinnen, Stephan; Albouy, Genevieve | Not applicable based on title |
| Effects of Chronic Cold-Water Immersion in Elite Rugby Players | 2019 | Tavares, Francisco; Beaven, Martyn; Teles, J˙lia; Baker, Dane; Healey, Phil; Smith, Tiaki B; Driller, Matthew | Not applicable based on title |
| Enactive phenomenological approach to the trier social stress test: A mixed methods point of view | 2019 | Vors, Olivier; Cury, FranÁois; Marqueste, Tanguy; Mascret, Nicolas | Not applicable based on title |
| Positive verbal feedback about task performance is related with adaptive physiological responses: An experimental study of the effort-reward imbalance stress model | 2019 | Brooks, Richard P; Jones, Monica T; Hale, Matthew W; Lunau, Thorsten; Dragano, Nico; Wright, Bradley J | Not applicable based on title |
| Ecological salivary cortisol specimen collection--part 1: methodological consideration of yield, error, and effects of sampling decisions in a perinatal mental health study | 2008 | Seng, Julia S; King, Anthony P; Gabriel, Cynthia; Reed, Caroline D; Sperlich, Mickey; Dunbar, Sara; Fraker, Emily; Ronis, David L | Not applicable based on title |
| Physical demands and salivary immunoglobulin A responses of elite Australian rules football athletes to match play | 2015 | Coad, Sam; Gray, Bon; Wehbe, George; McLellan, Christopher | Not applicable based on title |
| Prematch salivary secretory immunoglobulin a in soccer players from the 2014 World Cup qualifying campaign | 2015 | Morgans, Ryland; Owen, Adam; Doran, Dominic; Drust, Barry; Morton, James P | Not applicable based on title |
| Sleep-wake cycles and cognitive functioning in schizophrenia | 2011 | Bromundt, Vivien; Kˆster, Matthias; Georgiev-Kill, Angela; Opwis, Klaus; Wirz-Justice, Anna; Stoppe, Gabriela; Cajochen, Christian | Not applicable based on title |
| Living high-training low altitude training: effects on mucosal immunity | 2005 | Tiollier, E; Schmitt, L; Burnat, P; Fouillot, J-P; Robach, P; Filaire, E; Guezennec, Cy; Richalet, J-P | Not applicable based on title |
| Salivary IgA response to prolonged exercise in a hot environment in trained cyclists | 2005 | Laing, S J; Gwynne, D; Blackwell, J; Williams, M; Walters, R; Walsh, N P | Not applicable based on title |
| Intense training: mucosal immunity and incidence of respiratory infections | 2005 | Tiollier, E; Gomez-Merino, D; Burnat, P; Jouanin, J-C; Bourrilhon, C; Filaire, E; Guezennec, C Y; Chennaoui, M | Not applicable based on title |
| Salivary mitochondrial DNA copy number is associated with exercise ventilatory efficiency | 2017 | Chen, Yang; Hill, Helene Z; Lange, Gudrun; Falvo, Michael J | Included |
| Salivary VIP concentrations are elevated in humans after acute stress | 2013 | Ventre, Giovanni; Colonna, Caitlin; Smith, Jennifer; Alfano, Denise; Moldow, Roberta | Not applicable based on title |
| An increase in salivary interleukin-6 level following acute psychosocial stress and its biological correlates in healthy young adults | 2013 | Izawa, Shuhei; Sugaya, Nagisa; Kimura, Kenta; Ogawa, Namiko; Yamada, Kosuke C; Shirotsuki, Kentaro; Mikami, Ikuyo; Hirata, Kanako; Nagano, Yuichiro; Nomura, Shinobu | Not applicable based on title |
| Low vigorous physical activity is associated with increased adrenocortical reactivity to psychosocial stress in students with high stress perceptions | 2017 | Gerber, Markus; Ludyga, Sebastian; M¸cke, Manuel; Colledge, Flora; Brand, Serge; P¸hse, Uwe | Not applicable based on title |
| Daily stress magnifies the association between cognitive decline and everyday memory problems: an integration of longitudinal and diary methods | 2014 | Rickenbach, Elizabeth Hahn; Almeida, David M; Seeman, Teresa E; Lachman, Margie E | Not applicable based on title |
| Mucosal immune responses to treadmill exercise in elite wheelchair athletes | 2011 | Leicht, Christof Andreas; Bishop, Nicolette Claire; Goosey-Tolfrey, Victoria Louise | Not applicable based on title |
| Salivary hormonal responses and performance changes during 15 weeks of mixed aerobic and weight training in elite junior wrestlers | 2012 | Passelergue, Philippe A; Lac, GÈrard | Not applicable based on title |
| Salivary SIgA responses to acute moderate-vigorous exercise in monophasic oral contraceptive users | 2015 | Hayashida, Harumi; Dolan, Nicola J; Hounsome, Charlotte; Alajmi, Nawal; Bishop, Nicolette C | Not applicable based on title |
| Biomarkers of physical activity and exercise | 2015 | Palacios, Gonzalo; Pedrero-Chamizo, Raquel; Palacios, Nieves; Maroto-S·nchez, Beatriz; Aznar, Susana; Gonz·lez-Gross, Marcela; EXERNET Study Group | Not applicable based on title |
| Weight loss strategies, stress, and cognitive function: supervised versus unsupervised dieting | 2005 | Green, Michael W; Elliman, Nicola A; Kretsch, Mary J | Not applicable based on title |
| Evaluation of an integrated system of wearable physiological sensors for stress monitoring in working environments by using biological markers | 2018 | Betti, Stefano; Lova, Raffaele Molino; Rovini, Erika; Acerbi, Giorgia; Santarelli, Luca; Cabiati, Manuela; Del Ry, Silvia; Cavallo, Filippo | Not applicable based on title |
| The alerting effect of the wake maintenance zone during 40 hours of sleep deprivation | 2018 | Zeeuw, Jan de; Wisniewski, Sophia; Papakonstantinou, Alexandra; Bes, Frederik; Wahnschaffe, Amely; Zaleska, Mandy; Kunz, Dieter; M¸nch, Mirjam | Not applicable based on title |
| Self-reported emotion regulation difficulties are associated with mood but not with the biological stress response to thin ideal exposure | 2018 | Humbel, Nadine; Messerli-B¸rgy, Nadine; Schuck, Kathrin; Wyssen, Andrea; Garcia-Burgos, David; Biedert, Esther; Lennertz, Julia; Meyer, Andrea H; Whinyates, Katherina; Isenschmid, Bettina; Milos, Gabriella; Trier, Stephan; Adolph, Dirk; Cwik, Jan; Margraf, J¸rgen; Assion, Hans-Jˆrg; Teismann, Tobias; Ueberberg, Bianca; Juckel, Georg; M¸ller, Judith; Klauke, Benedikt; Schneider, Silvia; Munsch, Simone | Not applicable based on title |
| The effect of speed, power and strength training, and a group motivational presentation on physiological markers of athlete readiness: A case study in professional rugby | 2018 | Serpell, Benjamin G; Strahorn, Joshua; Colomer, Carmen; McKune, Andrew; Cook, Christian; Pumpa, Kate | No acute stressor |
| Black tea aroma inhibited increase of salivary chromogranin-A after arithmetic tasks | 2018 | Yoto, Ai; Fukui, Natsuki; Kaneda, Chisa; Torita, Shoko; Goto, Keiichi; Nanjo, Fumio; Yokogoshi, Hidehiko | Not applicable based on title |
| Late-night exercise affects the autonomic nervous system activity but not the hypothalamo-pituitary-adrenal axis in the next morning | 2018 | UÁar, Cihat; ÷zgˆÁer, Tuba; Yildiz, Sedat | Not applicable based on title |
| Inhalation of Japanese cedar (Cryptomeria japonica) wood odor causes psychological relaxation after monotonous work among female participants | 2005 | Matsubara, Eri; Ohira, Tatsuro | Not applicable based on title |
| Assessment of hydration biomarkers including salivary osmolality during passive and active dehydration | 2013 | MuÒoz, C X; Johnson, E C; Demartini, J K; Huggins, R A; McKenzie, A L; Casa, D J; Maresh, C M; Armstrong, L E | Included |
| Dynamic changes in saliva after acute mental stress | 2014 | Naumova, Ella A; Sandulescu, Tudor; Bochnig, Clemens; Al Khatib, Philipp; Lee, Wing-Kee; Zimmer, Stefan; Arnold, Wolfgang H | Not applicable based on title |
| The metabolic, hormonal, biochemical, and neuromuscular function responses to a backward sled drag training session | 2014 | West, Daniel J; Cunningham, Daniel J; Finn, Charlotte V; Scott, Phillip M; Crewther, Blair T; Cook, Christian J; Kilduff, Liam P | Not applicable based on title |
| Clinical decision-making augmented by simulation training: neural correlates demonstrated by functional imaging: a pilot study | 2014 | Goon, S S H; Stamatakis, E A; Adapa, R M; Kasahara, M; Bishop, S; Wood, D F; Wheeler, D W; Menon, D K; Gupta, A K | Not applicable based on title |
| Accelerometry and salivary cortisol response during Air Force Special Tactics Officer selection | 2013 | Cuddy, John S; Reinert, Andrew R; Hailes, Walter S; Slivka, Dustin R; Ruby, Brent C | Not applicable based on title |
| Use of personalized ventilation for improving health, comfort, and performance at high room temperature and humidity | 2013 | Melikov, A K; Skwarczynski, M A; Kaczmarczyk, J; Zabecky, J | Not applicable based on title |
| A friendly version of the trier social stress test does not activate the HPA axis in healthy men and women | 2013 | Wiemers, Uta S; Schoofs, Daniela; Wolf, Oliver T | Not applicable based on title |
| Detection of orexin A neuropeptide in biological fluids using a zinc oxide field effect transistor | 2013 | Hagen, Joshua; Lyon, Wanda; Chushak, Yaroslav; Tomczak, Melanie; Naik, Rajesh; Stone, Morley; Kelley-Loughnane, Nancy | Not applicable based on title |
| Environmental stress, oxytocin receptor gene (OXTR) polymorphism, and mental health following collective stress | 2013 | Lucas-Thompson, Rachel G; Holman, E Alison | Not applicable based on title |
| Call-associated acute fatigue in surgical residents--subjective perception or objective fact? A cross-sectional observational study to examine the influence of fatigue on surgical performance | 2012 | Schlosser, Katja; Maschuw, Katja; Kupietz, Eva; Weyers, Peter; Schneider, Ralph; Rothmund, Matthias; Hassan, Iyad; Bartsch, Detlef Klaus | Not applicable based on title |
| Psychosocial determinants of diurnal alpha-amylase among healthy Quebec workers | 2016 | Marchand, Alain; Juster, Robert-Paul; Lupien, Sonia J; Durand, Pierre | Not applicable based on title |
| Salivary lymphocyte responses following acute anaerobic exercise in a cool environment | 2017 | Carlson, Lara A; Lawrence, Michael A; LeCavalier, Kaylee; Koch, Alexander J | Not applicable based on title |
| Relationship of sleep alterations with perimenopausal and postmenopausal symptoms | 2014 | Moreno-FrÌas, Carmen; Figueroa-Vega, NictÈ; Malacara, Juan Manuel | Not applicable based on title |
| Stimulated saliva aminotransaminase alteration after experiencing acute hypoxia training | 2014 | Mominzadeh, Mahmud; Mirzaii-Dizgah, Iraj; Mirzaii-Dizgah, Mohammad-Reza; Mirzaii-Dizgah, Mohammad-Hossein | Performance not measured |
| Moderate acute exercise (70% VO2 peak) induces TGF-≤, ±-amylase and IgA in saliva during recovery | 2014 | Rosa, L; Teixeira, Aas; Lira, Fs; Tufik, S; Mello, Mt; Santos, Rvt | Not applicable based on title |
| Acute apnea swimming: metabolic responses and performance | 2014 | Guimard, Alexandre; Prieur, Fabrice; Zorgati, Houssem; Morin, David; Lasne, FranÁoise; Collomp, Katia | Not applicable based on title |
| Salivary IL-21 and IgA responses to a competitive match in elite basketball players | 2013 | Moreira, A; Bacurau, R F P; Napimoga, M H; Arruda, A F S; Freitas, C G; Drago, G; Aoki, M S | Not applicable based on title |
| Breakfast high in whey protein or carbohydrates improves coping with workload in healthy subjects | 2013 | Sihvola, Nora; Korpela, Riitta; Henelius, Andreas; Holm, Anu; Huotilainen, Minna; M¸ller, Kiti; Poussa, Tuija; Pettersson, Kati; Turpeinen, Anu; Peuhkuri, Katri | Not applicable based on title |
| Salivary antimicrobial protein responses during multistage ultramarathon competition conducted in hot environmental conditions | 2013 | Gill, Samantha Kirsty; Teixeira, Ana Maria; Rama, Luis; Rosado, F·tima; Hankey, Joanne; Scheer, Volker; Robson-Ansley, Paula; Costa, Ricardo Jose Soares | Not applicable based on title |
| Effect of Magnolia officinalis and Phellodendron amurense (ReloraÆ) on cortisol and psychological mood state in moderately stressed subjects | 2013 | Talbott, Shawn M; Talbott, Julie A; Pugh, Mike | Not applicable based on title |
| The impact of acute mental stress on brachial artery flow-mediated dilation differs when shear stress is elevated by reactive hyperemia versus handgrip exercise | 2013 | Szijgyarto, Ingrid C; King, Trevor J; Ku, Jennifer; Poitras, Veronica J; Gurd, Brendon J; Pyke, Kyra E | Not applicable based on title |
| Influence of carbohydrate ingestion on salivary immunoglobulin A following resistance exercise | 2013 | Carlson, Lara A; Kenefick, Robert W; Koch, Alexander J | Not applicable based on title |
| Flow injection spectrophotometric analysis of human salivary ±-amylase activity using an enzyme degradation of starch-iodine complexes in flow channel and its application to human stress testing | 2005 | Ohtomo, Takao; Igarashi, Shukuro; Takagai, Yoshitaka | Not applicable based on title |
| Correlation between salivary alpha-amylase, anxiety, and game records in the archery competition | 2016 | Lim, In Soo | Not applicable based on title |
| Hostility and telomere shortening among US military veterans: Results from the National Health and Resilience in Veterans Study | 2016 | Watkins, Laura E; Harpaz-Rotem, Ilan; Sippel, Lauren M; Krystal, John H; Southwick, Steven M; Pietrzak, Robert H | Not applicable based on title |
| Psychophysiological stress in tennis players during the first single match of a tournament | 2009 | Filaire, Edith; Alix, Deborah; Ferrand, Claude; Verger, Michel | Not applicable based on title |
| Rejection sensitivity relates to hypocortisolism and depressed mood state in young women | 2008 | Tops, Mattie; Riese, HarriÎtte; Oldehinkel, Albertine J; Rijsdijk, Fr¸hling V; Ormel, Johan | Not applicable based on title |
| Salivary immunoglobulin A and upper respiratory symptoms during 5 months of training in elite tetraplegic athletes | 2012 | Leicht, Christof A; Bishop, Nicolette C; Paulson, Thomas A W; Griggs, Katy E; Goosey-Tolfrey, Victoria L | Not applicable based on title |
| Mechanical, hormonal and psychological effects of a non-failure short-term strength training program in young tennis players | 2015 | Sarabia, Jose Manuel; Fernandez-Fernandez, Jaime; Juan-Recio, Casto; Hern·ndez-DavÛ, Hector; Urb·n, Tom·s; Moya, Manuel | Not applicable based on title |
| Dehydration decreases saliva antimicrobial proteins important for mucosal immunity | 2012 | Fortes, Matthew B; Diment, Bethany C; Di Felice, Umberto; Walsh, Neil P | Not applicable based on title |
| Pre-competition hormonal and psychological levels of elite hockey players: relationship to the "home advantage" | 2006 | CarrÈ, Justin; Muir, Cameron; Belanger, Joey; Putnam, Susan K | Not applicable based on title |
| Salivary steroids at rest and after a training load in young male athletes: relationship with chronological age and pubertal development | 2006 | Di Luigi, L; Baldari, C; Gallotta, M C; Perroni, F; Romanelli, F; Lenzi, A; Guidetti, L | Not applicable based on title |
| Physiological and mechanical response to soccer-specific intermittent activity and steady-state activity | 2006 | Greig, Matt P; McNaughton, Lars R; Lovell, Ric J | Not applicable based on title |
| Saliva DHEAS changes in patients suffering from psychopathological disorders arising from bullying at work | 2012 | Lac, GÈrard; Dutheil, FrÈdÈric; Brousse, Georges; Triboulet-Kelly, CÈline; Chamoux, Alain | Not applicable based on title |
| Biopsychological markers of distress in informal caregivers | 2004 | Davis, Linda Lindsey; Weaver, Michael; Zamrini, Edward; Stevens, Alan; Kang, Duck-Hee; Parker, C Richard | Not applicable based on title |
| Back massage therapy promotes psychological relaxation and an increase in salivary chromogranin A release | 2010 | Noto, Yuka; Kudo, Mihoko; Hirota, Kazuyoshi | Not applicable based on title |
| Caffeinated chewing gum increases repeated sprint performance and augments increases in testosterone in competitive cyclists | 2010 | Paton, Carl D; Lowe, Timothy; Irvine, Athena | Not applicable based on title |
| Salivary immunoglobulin A responses in professional top-level futsal players | 2011 | Moreira, Alexandre; Arsati, Franco; de Oliveira Lima-Arsati, Ynara Bosco; de Freitas, Camila Gobo; de Ara˙jo, Vera Cavalcanti | Not applicable based on title |
| Stress reactions to cognitively demanding tasks and open-plan office noise | 2009 | Kristiansen, Jesper; Mathiesen, Line; Nielsen, Pernille Kofoed; Hansen, Ase Marie; Shibuya, Hitomi; Petersen, Helga Munch; Lund, S¯ren Peter; Skotte, J¯rgen; J¯rgensen, Marie Birk; S¯gaard, Karen | Not applicable based on title |
| Relationship between salivary Chromogranin-A and stress induced by simulated monotonous driving | 2009 | Yamakoshi, Takehiro; Park, Sang-Bum; Jang, Won-Cheoul; Kim, Kyungho; Yamakoshi, Yasuhiro; Hirose, Hajime | Not applicable based on title |
| Better not to deal with two tasks at the same time when stressed? Acute psychosocial stress reduces task shielding in dual-task performance | 2012 | Plessow, Franziska; Schade, Susann; Kirschbaum, Clemens; Fischer, Rico | Not applicable based on title |
| Psychological and physiological response of students to different types of stress management programs | 2012 | Iglesias, Silvia L; Azzara, Sergio; Argibay, Juan Carlos; Arnaiz, MarÌa Lores; de Valle Carpineta, MarÌa; Granchetti, Hugo; Lagomarsino, Eduardo | Not applicable based on title |
| Improvement in physiological and psychological parameters after 6 months of yoga practice | 2012 | Rocha, K K F; Ribeiro, A M; Rocha, K C F; Sousa, M B C; Albuquerque, F S; Ribeiro, S; Silva, R H | Not applicable based on title |
| Effects of transcutaneous electrical nerve stimulation on motion sickness induced by rotary chair: a crossover study | 2012 | Chu, Hsin; Li, Min-Hui; Juan, Szu-Hsuan; Chiou, Wen-Yao | Not applicable based on title |
| Stress related changes during TeamGym competition | 2016 | DE Pero, Roberta; Cibelli, Giuseppe; Cortis, Cristina; Sbriccoli, Paola; Capranica, Laura; Piacentini, Maria F | Not applicable based on title |
| Salivary chromogranin A, but not ±-amylase, correlates with cardiovascular parameters during high-intensity exercise | 2011 | Gallina, Sabina; Di Mauro, Michele; D'Amico, Maria Angela; D'Angelo, Emanuele; Sablone, Andrea; Di Fonso, Alessia; Bascelli, Adriana; Izzicupo, Pascal; Di Baldassarre, Angela | Not applicable based on title |
| High-altitude headache: the effects of real vs sham oxygen administration | 2015 | Benedetti, Fabrizio; Durando, Jennifer; Giudetti, Lucia; Pampallona, Alan; Vighetti, Sergio | Not applicable based on title |
| Acute and chronic effects of tantric yoga practice on distress index | 2015 | Batista, Janir Coutinho; Souza, AglÈcio Luiz; Ferreira, Heloisa Aparecida; Canova, Fernando; Grassi-Kassisse, Dora Maria | Not applicable based on title |
| Effect of acupuncture on salivary immunoglobulin A after a bout of intense exercise | 2010 | Matsubara, Yuichi; Shimizu, Kazuhiro; Tanimura, Yuko; Miyamoto, Toshikazu; Akimoto, Takayuki; Kono, Ichiro | Not applicable based on title |
| Elevated plasma interleukin-6 levels in trained male triathletes following an acute period of intense interval training | 2007 | Robson-Ansley, Paula J; Blannin, Andrew; Gleeson, Michael | Not applicable based on title |
| Acute salivary hormone responses to complex exercise bouts | 2011 | Beaven, C Martyn; Gill, Nicholas D; Ingram, John R; Hopkins, Will G | Not applicable based on title |
| [Evaluation of a pilot health promotion and stress management program for Pharmacy and Biochemistry students and professionals] | 2014 | Iglesias, S L; Granchetti, H; Azzara, S; Carpineta, M; Pappalardo, M; Argibay, J C; Lagomarsino, E | Not applicable based on title |
| Effects among healthy subjects of the duration of regularly practicing a guided imagery program | 2005 | Watanabe, Eri; Fukuda, Sanae; Shirakawa, Taro | Not applicable based on title |
| The immediate effects of 10-minute relaxation training on salivary immunoglobulin A (s-IgA) and mood state for Japanese female medical co-workers | 2007 | Taniguchi, Toshiyo; Hirokawa, Kumi; Tsuchiya, Masao; Kawakami, Norito | Not applicable based on title |
| Sex differences in response to cognitive stress during a fatiguing contraction | 2009 | Yoon, Tejin; Keller, Manda L; De-Lap, Bonnie Schlinder; Harkins, April; Lepers, Romuald; Hunter, Sandra K | Undefined stressor in criteria |
| Deficits in behavioral inhibition predict treatment engagement in prison inmates | 2009 | Fishbein, Diana; Sheppard, Monica; Hyde, Christopher; Hubal, Robert; Newlin, David; Serin, Ralph; Chrousos, George; Alesci, Salvatore | Not applicable based on title |
| Mucosal immunity and illness incidence in elite rugby union players across a season | 2011 | Cunniffe, Brian; Griffiths, Hywel; Proctor, Wayne; Davies, Bruce; Baker, Julien S; Jones, Ken P | Not applicable based on title |
| The effects of carbohydrate supplementation during repeated bouts of prolonged exercise on saliva flow rate and immunoglobulin A | 2005 | Li, Tzai-Li; Gleeson, Michael | Not applicable based on title |
| A very low level of magnetic field exposure does not affect a participant's mental fatigue and stress as much as VDT work | 2005 | Ishihara, Itsuko; Ikushima, Miharu; Horikawa, Junko; Haraga, Miki; Kawamoto, Rieko; Murase, Chiharu; Tashiro, Taku; Tsutsui, Yasuhiro; Kawashima, Masatoshi; Kasai, Hiroshi; Yamazaki, Sayumi; Majima, Yukie; Kurokawa, Yoshika | Not applicable based on title |
| Effects of 21 days of intensified training on markers of overtraining | 2010 | Slivka, Dustin R; Hailes, Walther S; Cuddy, John S; Ruby, Brent C | Included |
| Psychophysiological stress responses during training and competition in young female competitive tennis players | 2015 | Fernandez-Fernandez, J; Boullosa, D A; Sanz-Rivas, D; Abreu, L; Filaire, E; Mendez-Villanueva, A | Not applicable based on title |
| The effect of single and repeated bouts of prolonged cycling and circadian variation on saliva flow rate, immunoglobulin A and alpha-amylase responses | 2004 | Li, Tzai-Li; Gleeson, Michael | Not applicable based on title |
| Mucosal immunity modulated by integrative meditation in a dose-dependent fashion | 2010 | Fan, Yaxin; Tang, Yi-Yuan; Ma, Yinghua; Posner, Michael I | Not applicable based on title |
| Influence of timing of postexercise carbohydrate-protein ingestion on selected immune indices | 2009 | Costa, Ricardo J S; Oliver, Samuel J; Laing, Stewart J; Waiters, Robert; Bilzon, James L J; Walsh, Neil P | Not applicable based on title |
| Self-Knowledge Dim-Out: Stress Impairs Metacognitive Accuracy | 2015 | Reyes, Gabriel; Silva, Jaime R; Jaramillo, Karina; Rehbein, Lucio; Sackur, JÈrÙme | Not applicable based on title |
| Whole-Body Cryostimulation Limits Overreaching in Elite Synchronized Swimmers | 2015 | Schaal, Karine; LE Meur, Yann; Louis, Julien; Filliard, Jean-Robert; Hellard, Philippe; Casazza, Gretchen; Hausswirth, Christophe | Not applicable based on title |
| Exercise, but not acute sleep loss, increases salivary antimicrobial protein secretion | 2015 | Gillum, Trevor L; Kuennen, Matthew R; Castillo, Micaela N; Williams, Nicole L; Jordan-Patterson, Alex T | Not applicable based on title |
| Sleep and the endogenous melatonin rhythm of high arctic residents during the summer and winter | 2015 | Paul, Michel A; Love, Ryan J; Hawton, Andrea; Arendt, Josephine | Not applicable based on title |
| Biopsychosocial influence on shoulder pain: risk subgroups translated across preclinical and clinical prospective cohorts | 2015 | George, Steven Z; Wallace, Margaret R; Wu, Samuel S; Moser, Michael W; Wright, Thomas W; Farmer, Kevin W; Borsa, Paul A; Parr, Jeffrey J; Greenfield, Warren H; Dai, Yunfeng; Li, Hua; Fillingim, Roger B | Not applicable based on title |
| The contributions of arousal and self-focused attention to avoidance in social anxiety | 2005 | Grisham, Jessica R; King, Bradley J; Makkar, Steve R; Felmingham, Kim L | Not applicable based on title |
| The impact of a 17-day training period for an international championship on mucosal immune parameters in top-level basketball players and staff members | 2008 | Moreira, Alexandre; Arsati, Franco; Cury, PatrÌcia Ramos; Franciscon, ClÛvis; Simıes, Antonio Carlos; de Oliveira, Paulo Roberto; de Ara˙jo, Vera Cavalcanti | Not applicable based on title |
| Salivary IgA as a risk factor for upper respiratory infections in elite professional athletes | 2008 | Neville, Vernon; Gleeson, Michael; Folland, Jonathan P | Not applicable based on title |
| Saliva pH as a biomarker of exam stress and a predictor of exam performance | 2014 | Cohen, Miri; Khalaila, Rabia | Not applicable based on title |
| Tennis, incidence of URTI and salivary IgA | 2003 | Novas, A M P; Rowbottom, D G; Jenkins, D G | Not applicable based on title |
| Anger and psychobiological changes during smoking abstinence and in response to acute stress: prediction of smoking relapse | 2007 | al'Absi, Mustafa; Carr, Steven B; Bongard, Stephan | Not applicable based on title |
| A common polymorphism in the mineralocorticoid receptor modulates stress responsiveness | 2006 | DeRijk, Roel H; W¸st, Stefan; Meijer, Onno C; Zennaro, Maria-Christina; Federenko, Ilona S; Hellhammer, Dirk H; Giacchetti, Gilberta; Vreugdenhil, Erno; Zitman, Frans G; de Kloet, E Ronald | Not applicable based on title |
| Physiological dynamic apnea responses in relation to apnea capacity in triathletes | 2017 | Guimard, Alexandre; Zorgati, Houssem; Brulaire, Sylvain; Amiot, Virgile; Prieur, Fabrice; Collomp, Katia | Not applicable based on title |
| Dynamic changes in salivary cortisol and secretory immunoglobulin A response to acute stress | 2009 | Fan, Yaxin; Tang, Yiyuan; Lu, Qilin; Feng, Shigang; Yu, Qingbao; Sui, Danni; Zhao, Qingbai; Ma, Yinghua; Li, Song | Not applicable based on title |
| Lactate, cortisol and testosterone values from high-speed resistive exercise workouts done by women | 2014 | Caruso, J.F.; Urquhart, M.A.; Barbosa, A.G.; Giebel, R.M.; Learmonth, L.A.; Potter, W.T. | Not applicable based on title |
| Thermoregulatory vs event sweating--comparison of clinical methodologies, physiology and results | 2014 | Biehle-Hulette, S J; Krailler, J M; Elstun, L T; Bentz, S; Benzing, K W; Spruell, R D; Hellhammer, J; Swaile, D F | Not applicable based on title |
| Intention retrieval and deactivation following an acute psychosocial stressor | 2013 | Walser, Moritz; Fischer, Rico; Goschke, Thomas; Kirschbaum, Clemens; Plessow, Franziska | Not applicable based on title |
| Salivary biomarkers of physical fatigue as markers of sleep deprivation | 2013 | Michael, Darren J; Valle, Bianca; Cox, Jennifer; Kalns, John E; Fogt, Donovan L | Included |
| Impact of aerobic exercise intensity on craving and reactivity to smoking cues | 2013 | Janse Van Rensburg, Kate; Elibero, Andrea; Kilpatrick, Marcus; Drobes, David J | Not applicable based on title |
| Dietary nucleotide improves markers of immune response to strenuous exercise under a cold environment | 2013 | Riera, Joan; Pons, Victoria; Martinez-Puig, Daniel; Chetrit, Carlos; Tur, Josep A; Pons, Antoni; Drobnic, Franchek | Not applicable based on title |
| Effects of strongman training on salivary testosterone levels in a sample of trained men | 2013 | Ghigiarelli, Jamie J; Sell, Katie M; Raddock, Jessica M; Taveras, Kurt | Not applicable based on title |
| The effects of an acute dose of Rhodiola rosea on endurance exercise performance | 2013 | Noreen, Eric E; Buckley, James G; Lewis, Stephanie L; Brandauer, Josef; Stuempfle, Kristin J | Not applicable based on title |
| Can decision-making skills affect responses to psychological stress in healthy women? | 2012 | Santos-Ruiz, Ana; Garcia-Rios, M Carmen; Fernandez-Sanchez, JosÈ Carlos; Perez-Garcia, Miguel; MuÒoz-GarcÌa, Miguel Angel; Peralta-Ramirez, Maria Isabel | Not applicable based on title |
| Calf training for loading onto vehicle at weaning | 2012 | Fukasawa, Michiru | Not applicable based on title |
| Natural sports supplement formulation for physical endurance: a randomized, double-blind, placebo-controlled study | 2017 | Gopi, Sreeraj; Jacob, Joby; Varma, Karthik; Amalraj, Augustine; Sreeraj, T. R.; Kunnumakkara, Ajaikumar B.; Divya, Chandradhara | Not applicable based on title |
| Taiji practice attenuates psychobiological stress reactivity--a randomized controlled trial in healthy subjects | 2012 | Nedeljkovic, Marko; Ausfeld-Hafter, Brigitte; Streitberger, Konrad; Seiler, Roland; Wirtz, Petra H | Not applicable based on title |
| Oral administration of Cimicifuga racemosa extract attenuates psychological and physiological stress responses | 2012 | Nadaoka, Isao; Yasue, Masaaki; Kitagawa, Yasushi; Koga, Yoshihiko | Not applicable based on title |
| Maternal factors regulating preterm infants' responses to pain and stress while in maternal kangaroo care | 2012 | Castral, ThaÌla CorrÍa; Warnock, Fay Fathalee; Ribeiro, Laiane Medeiros; de Vasconcelos, Maria Gorete Lucena; Leite, Adriana Moraes; Scochi, Carmen Gracinda Silvan | Not applicable based on title |
| Monitoring internal load parameters during simulated and official basketball matches | 2012 | Moreira, Alexandre; McGuigan, Michael R; Arruda, Ademir F s; Freitas, Camila G; Aoki, Marcelo S | Not applicable based on title |
| The acute potentiating effects of back squats on athlete performance | 2011 | Crewther, Blair T; Kilduff, Liam P; Cook, Christian J; Middleton, Matt K; Bunce, Paul J; Yang, Guang-Zhong | Not applicable based on title |
| Higher frontal EEG synchronization in young women with major depression: a marker for increased homeostatic sleep pressure? | 2011 | Birchler-Pedross, Angelina; Frey, Sylvia; Chellappa, Sarah Laxhmi; Gˆtz, Thomas; Brunner, Patrick; Knoblauch, Vera; Wirz-Justice, Anna; Cajochen, Christian | Not applicable based on title |
| Immune responses to exercising in a cold environment | 2011 | LaVoy, Emily C P; McFarlin, Brian K; Simpson, Richard J | Not applicable based on title |
| Positional demands of professional rugby | 2015 | Lindsay, Angus; Draper, Nick; Lewis, John; Gieseg, Steven P; Gill, Nicholas | Not applicable based on title |
| Aerobic exercise reduced oxidative stress in saliva of persons with Down syndrome | 2005 | Zambrano, Jean C; Marquina, RamÛn; Sulbar·n, Nancy; RodrÌguez-Malaver, Antonio J; Reyes, Rafael A | Not applicable based on title |
| Innate immune responses to a single session of sprint interval training | 2011 | Davison, Glen | Not applicable based on title |
| Signs of overload after an intensified training | 2011 | Bresciani, G; Cuevas, M J; Molinero, O; Almar, M; Suay, F; Salvador, A; de Paz, J A; Marquez, S; Gonz·lez-Gallego, J | Not applicable based on title |
| Detailed assessment of the hemodynamic response to psychosocial stress using real-time MRI | 2011 | Jones, Alexander; Steeden, Jennifer A; Pruessner, Jens C; Deanfield, John E; Taylor, Andrew M; Muthurangu, Vivek | Not applicable based on title |
| Stress-related hormonal and psychological changes to official youth Taekwondo competitions | 2011 | Chiodo, S; Tessitore, A; Cortis, C; Cibelli, G; Lupo, C; Ammendolia, A; De Rosas, M; Capranica, L | Not applicable based on title |
| Stress management interventions in the workplace improve stress reactivity: a randomised controlled trial | 2011 | Limm, Heribert; G¸ndel, Harald; Heinm¸ller, Mechthild; Marten-Mittag, Birgitt; Nater, Urs M; Siegrist, Johannes; Angerer, Peter | Not applicable based on title |
| Psychophysiological effects of preperformance massage before isokinetic exercise | 2011 | Arroyo-Morales, Manuel; Fern·ndez-Lao, Carolina; Ariza-GarcÌa, Angelica; Toro-Velasco, Cristina; Winters, Marinus; DÌaz-RodrÌguez, Lourdes; Cantarero-Villanueva, Irene; Huijbregts, Peter; Fern·ndez-De-las-PeÒas, Cesar | Not applicable based on title |
| [Specific trait and state anxiety's roles in emergence and maintenance of attentional biases associated with anxiety: Inventories and investigation tracks] | 2009 | Bardel, M-H; Colombel, F | Not applicable based on title |
| Effects of quercetin and EGCG on mitochondrial biogenesis and immunity | 2009 | Nieman, David C; Henson, Dru A; Maxwell, Kendra R; Williams, Ashley S; McAnulty, Steven R; Jin, Fuxia; Shanely, R Andrew; Lines, Thomas C | Not applicable based on title |
| Negative affect and chemical intolerance as risk factors for building-related symptoms: a controlled exposure study | 2008 | Fiedler, Nancy; Kelly-McNeil, Kathie; Ohman-Strickland, Pamela; Zhang, Junfeng; Ottenweller, John; Kipen, Howard M | Not applicable based on title |
| Higher overcommitment to work is associated with lower norepinephrine secretion before and after acute psychosocial stress in men | 2008 | Wirtz, Petra H; Siegrist, Johannes; Rimmele, Ulrike; Ehlert, Ulrike | Not applicable based on title |
| Psychophysiologic responses of invasive cardiologists in an academic catheterization laboratory | 2006 | Detling, Nicole; Smith, Aynsley; Nishimura, Rick; Keller, Shelly; Martinez, Matthew; Young, William; Holmes, David | Not applicable based on title |
| Temporal variation of acute stress responses in sympathetic nervous and immune systems | 2005 | Kimura, Kenta; Isowa, Tokiko; Ohira, Hideki; Murashima, Seikou | Not applicable based on title |
| Biological and psychological responses to two rapid shiftwork schedules | 2004 | Lac, G; Chamoux, A | Chronic/Prolonged stressor |
| Saliva parameters as potential indices of hydration status during acute dehydration | 2004 | Walsh, Neil P; Laing, Stewart J; Oliver, Samuel J; Montague, Joanna C; Walters, Robert; Bilzon, James L J | Included |
| Psychophysiological effects of nicotine abstinence and behavioral challenges in habitual smokers | 2002 | al'Absi, Mustafa; Amunrud, Todd; Wittmers, Lorentz E | Not applicable based on title |
| The risk of being shot at: Stress, cortisol secretion, and their impact on memory and perceived learning during reality-based practice for armed officers | 2011 | Taverniers, John; Smeets, Tom; Van Ruysseveldt, Joris; Syroit, Jef; von Grumbkow, Jasper | Not applicable based on title |
| Social-cognitive predictors of hypothalamic-pituitary-adrenal reactivity to interpersonal conflict in emerging adult couples | 2006 | Laurent, Heidemarie K.; Powers, Sally I. | Not applicable based on title |
| Diurnal variation in the salivary melatonin responses to exercise: relation to exercise-mediated tachycardia | 2011 | Marrin, K; Drust, B; Gregson, W; Morris, C J; Chester, N; Atkinson, G | Not applicable based on title |
| Efficacy of abbreviated progressive muscle relaxation in a high-stress college sample | 2012 | Dolbier, Christyn L.; Rush, Taylor E. | Not applicable based on title |
| Changes in the salivary biomarkers induced by an effort test | 2010 | de Oliveira, V N; Bessa, A; Lamounier, R P M S; de Santana, M G; de Mello, M T; Espindola, F S | Not applicable based on title |
| The timing-dependent effects of stress-induced cortisol release on evaluative conditioning | 2020 | Halbeisen, Georg; Buttlar, Benjamin; Kamp, Siri-Maria; Walther, Eva | Not applicable based on title |
| Effects of acute aerobic and resistance exercise on cognitive function and salivary cortisol responses | 2019 | Wang, Chun-Chih; Alderman, Brandon; Wu, Chih-Han; Chi, Lin; Chen, Su-Ru; Chu, I-Hua; Chang, Yu-Kai | Not applicable based on title |
| The creative thinking cognitive process influenced by acute stress in humans: an electroencephalography study | 2019 | Wang, Xuewei; Duan, Haijun; Kan, Yuecui; Wang, Botao; Qi, Senqing; Hu, Weiping | Not applicable based on title |
| Interactive effects of testosterone and cortisol on hippocampal volume and episodic memory in middle-aged men | 2018 | Panizzon, Matthew S; Hauger, Richard L; Xian, Hong; Jacobson, Kristen; Lyons, Michael J; Franz, Carol E; Kremen, William S | Not applicable based on title |
| Conditioning cortisol in healthy young women - A randomized controlled trial | 2021 | Tekampe, Judith; van Middendorp, HenriÎt; Biermasz, Nienke R; Sweep, Fred C G J; Meijer, Onno C; Pelsma, Iris C M; Pereira, Alberto M; Hermus, Ad R M M; Evers, Andrea W M | Not applicable based on title |
| Influences of increased training volume on exercise performance, physiological and psychological parameters | 2016 | Kageta, Tomoyo; Tsuchiya, Yoshifumi; Morishima, Takuma; Hasegawa, Yuta; Sasaki, Hiroto; Goto, Kazushige | Not applicable based on title |
| Trait rumination and response to negative evaluative lab-induced stress: neuroendocrine, affective, and cognitive outcomes | 2019 | Vrshek-Schallhorn, Suzanne; Velkoff, Elizabeth A; Zinbarg, Richard E | Not applicable based on title |
| Dopaminergic and noradrenergic modulation of stress-induced alterations in brain activation associated with goal-directed behaviour | 2021 | van Ruitenbeek, Peter; Quaedflieg, Conny Wem; Hernaus, Dennis; Hartogsveld, Bart; Smeets, Tom | Not applicable based on title |
| Human nail cortisol as a retrospective biomarker of chronic stress: A systematic review | 2021 | Phillips, Riana; Kraeuter, Ann-Katrin; McDermott, Brett; Lupien, Sonia; Sarnyai, Zolt·n | Not applicable based on title |
| Efficacy of edible bird's nest on cognitive functions in experimental animal models: A systematic review | 2021 | Ismail, Maznah; Alsalahi, Abdulsamad; Aljaberi, Musheer Abdulwahid; Ibrahim, Ramlah Mohamad; Bakar, Faizah Abu; Ideris, Aini | Not applicable based on title |
| Imbalance Between Salivary Cortisol and DHEA Responses Is Associated with Social Cost and Self-perception to Social Evaluative Threat in Japanese Healthy Young Adults | 2020 | Shirotsuki, Kentaro; Izawa, Shuhei; Sugaya, Nagisa; Kimura, Kenta; Ogawa, Namiko; Yamada, Kosuke Chris; Nagano, Yuichiro | Not applicable based on title |
| Acute hypobaric-hypoxia challenge and salivary cortisol and DHEA-S in healthy male subjects | 2012 | Pontremolesi, Sara; Biselli, Roberto; Ciniglio Appiani, Giuseppe; D'Amelio, Raffaele; Simona, Simeoni; Patacchioli, Francesca Romana | Included |
| Dexamethasone-suppressed Salivary Cortisol and Pain Sensitivity in Female Twins | 2017 | Godfrey, Kathryn M; Herbert, Matthew; Strachan, Eric; Mostoufi, Sheeva; Crofford, Leslie J; Buchwald, Dedra; Poeschla, Brian; Succop, Annemarie; Afari, Niloofar | Not applicable based on title |
| Chewing unflavored gum does not reduce cortisol levels during a cognitive task but increases the response of the sympathetic nervous system | 2016 | Walker, Jessica; Hosiner, Andreas; Kergoat, Sophie; Walker, Joel M; Somoza, Veronika | Not applicable based on title |
| Maximal strength and cortisol responses to psyching-up during the squat exercise | 2005 | McGuigan, Michael R; Ghiagiarelli, Jamie; Tod, David | Not applicable based on title |
| Influence of acute stress on response inhibition in healthy men: An ERP study | 2017 | Dierolf, Angelika Margarete; Fechtner, Julia; Bˆhnke, Robina; Wolf, Oliver T; Naumann, Ewald | Not applicable based on title |
| Listening to motivational music during warming-up attenuates the negative effects of partial sleep deprivation on cognitive and short-term maximal performance: Effect of time of day | 2021 | Khemila, Syrine; Abedelmalek, Salma; Romdhani, Mohamed; Souissi, Amine; Chtourou, Hamdi; Souissi, Nizar | Not applicable based on title |
| Medical students preferring a surgical or non-surgical elective differ in their emotional and hormonal responses to a psychological stressor | 2020 | Crewther, Blair T; Cook, Christian J | Not applicable based on title |
| Relationship between alexithymia, alexithymia factors and salivary cortisol in men exposed to a social stress test | 2008 | de Timary, Philippe; Roy, Emmanuel; Luminet, Olivier; FillÈe, Catherine; Mikolajczak, MoÔra | Not applicable based on title |
| [Increased cortisol levels, frostbite and effects on the muscles and skeleton during extreme polar conditions] | 2003 | Steine, Kjetil; R¯seth, Arne G; Sandbaek, Gunnar; Murison, Robert; Slagsvold, Carl-Erik; Keller, Anne; Falch, Jan A | Not applicable based on title |
| Differences between the physiologic and psychologic effects of aromatherapy body treatment | 2008 | Takeda, Hitomi; Tsujita, Junzo; Kaya, Mitsuharu; Takemura, Masanori; Oku, Yoshitaka | Not applicable based on title |
| Psychophysiological stress response during competition between elite and non-elite Korean junior golfers | 2009 | Kim, K J; Chung, J W; Park, S; Shin, J T | Not applicable based on title |
| Differential impact of the first and second wave of a stress response on subsequent fear conditioning in healthy men | 2013 | Antov, Martin I; Wˆlk, Christoph; Stockhorst, Ursula | Not applicable based on title |
| Less immune activation following social stress in rural vs urban participants raised with regular or no animal contact, respectively | 2018 | Bˆbel, Till S; Hackl, Sascha B; Langgartner, Dominik; Jarczok, Marc N; Rohleder, Nicolas; Rook, Graham A; Lowry, Christopher A; G¸ndel, Harald; Waller, Christiane; Reber, Stefan O | Not applicable based on title |
| Tyrosine supplementation mitigates working memory decrements during cold exposure | 2007 | Mahoney, Caroline R; Castellani, John; Kramer, F Matthew; Young, Andrew; Lieberman, Harris R | Not applicable based on title |
| Latent inhibition of rotation chair-induced nausea in healthy male and female volunteers | 2005 | Klosterhalfen, Sibylle; Kellermann, Sandra; Stockhorst, Ursula; Wolf, Jutta; Kirschbaum, Clemens; Hall, Geoffrey; Enck, Paul | Not applicable based on title |
| Effects of Probiotic Supplementation on Immune and Inflammatory Markers in Athletes: A Meta-Analysis of Randomized Clinical Trials | 2022 | Guo, Yi-Ting; Peng, Yu-Ching; Yen, Hsin-Yen; Wu, Jeng-Cheng; Hou, Wen-Hsuan | Not applicable based on title |
| High indoor CO2 concentrations in an office environment increases the transcutaneous CO2 level and sleepiness during cognitive work | 2005 | Vehvil‰inen, Tommi; Lindholm, Harri; Rintam‰ki, Hannu; P‰‰kkˆnen, Rauno; Hirvonen, Ari; Niemi, Olli; Vinha, Juha | Not applicable based on title |
| Noninvasive evaluation of the chronic influence of local air velocity from an air conditioner using salivary cortisol and skin caspase-14 as biomarkers of psychosomatic and environmental stress | 2005 | Yamaguchi, M; Nishimiya, H | Not applicable based on title |
| Public speaking in front of an unreceptive audience increases implicit power motivation and its endocrine arousal signature | 2015 | Wiemers, Uta S; Schultheiss, Oliver C; Wolf, Oliver T | Not applicable based on title |
| Type of self-talk matters: Its effects on perceived exertion, cardiorespiratory, and cortisol responses during an iso-metabolic endurance exercise | 2022 | Basset, Fabien A; Kelly, Liam P; Hohl, Rodrigo; Kaushal, Navin | Not applicable based on title |
| Influence of long-term exposure to an air-conditioned environment on the diurnal cortisol rhythm | 2006 | Ueno, Tomoko; Ohnaka, Tadakatsu | Not applicable based on title |
| Cortisol, testosterone and mood state variation during an official female football competition | 2016 | Casanova, Natalina; Palmeira-DE-Oliveira, Ana; Pereira, Ana; CrisÛstomo, LuÌs; Travassos, Bruno; Costa, Aldo M | Not applicable based on title |
| Reliability of salivary cortisol and testosterone to a high-intensity cycling protocol to highlight overtraining | 2021 | Hough, John; Leal, Diogo; Scott, Gemma; Taylor, Lee; Townsend, Dominic; Gleeson, Michael | Not applicable based on title |
| Cortisol awakening response in the airborne rescue service | 2022 | Braun, D; Frank, M; Theiler, L; Petrowski, K | Not applicable based on title |
| The impact of prior day sleep and physical activity on the cortisol awakening response | 2021 | Anderson, Travis; Corneau, Gail; Wideman, Laurie; Eddington, Kari; Vrshek-Schallhorn, Suzanne | Not applicable based on title |
| Weekly Fluctuations in Salivary Hormone Responses and Their Relationships With Load and Well-Being in Semiprofessional, Male Basketball Players During a Congested In-Season Phase | 2022 | Kamarauskas, Paulius; Lukonaitien, Inga; Scanlan, Aaron T; Ferioli, Davide; Paulauskas, Henrikas; Conte, Daniele | chronic/Prolonged stressor |
| Transient changes in frontal alpha asymmetry as a measure of emotional and physical distress during sleep | 2011 | Flo, Elisabeth; Steine, Iris; BlÂgstad, Tone; Gr¯nli, Janne; Pallesen, StÂle; Portas, Chiara M | Not applicable based on title |
| Electrostimulation's enhancement of recovery during a rugby preseason | 2013 | Beaven, C Martyn; Cook, Christian; Gray, David; Downes, Paul; Murphy, Ian; Drawer, Scott; Ingram, John R; Kilduff, Liam P; Gill, Nicholas | Not applicable based on title |
| Cortisol release, heart rate and heart rate variability in the horse and its rider: different responses to training and performance | 2013 | von Lewinski, Mareike; Biau, Sophie; Erber, Regina; Ille, Natascha; Aurich, Jˆrg; Faure, Jean-Michel; Mˆstl, Erich; Aurich, Christine | Not applicable based on title |
| Mind/body techniques for physiological and psychological stress reduction: stress management via Tai Chi training - a pilot study | 2007 | Esch, Tobias; Duckstein, Jorg; Welke, Justus; Braun, Vittoria | Not applicable based on title |
| Effect of high-fidelity simulation on alpha-amylase activity and concentrations of secretory immunoglobulin class A, cortisol, and testosterone among medical students | 2021 | Bialka, Szymon; Copik, Maja; Ubych, Adam; Marciniak, RadosBaw; Smereka, Jacek; Szarpak, Lukasz; Misiolek, Hanna | Not applicable based on title |
| Correlation between salivary biochemical stress indicators and psychological indicators | 2018 | Crnkovi, Danijel; Peco, Mirna; Gelo, Josipa | Not applicable based on title |
| The effects of suppressing the biological stress systems on social threat-assessment following acute stress | 2020 | Ali, Nida; Cooperman, Cory; Nitschke, Jonas P; Baldwin, Mark W; Pruessner, Jens C | Not applicable based on title |
| Exercise-Induced Salivary Hormone Responses to High-Intensity, Self-Paced Running | 2021 | Leal, Diogo V; Taylor, Lee; Hough, John | Not applicable based on title |
| Effects of a work-related stress model based mental health promotion program on job stress, stress reactions and coping profiles of women workers: a control groups study | 2020 | Ornek, Ozlem Koseoglu; Esin, Melek Nihal | Not applicable based on title |
| Cortisol, chronotype, and coping styles as determinants of tolerance of nursing staff to rotating shift work | 2021 | Minelli, Andrea; Di Palma, Michael; Rocchi, Marco Bruno Luigi; Ponzio, Elisa; Barbadoro, Pamela; Bracci, Massimo; Pelusi, Gilda; Prospero, Emilia | Not applicable based on title |
| ≤-Eudesmol, an Oxygenized Sesquiterpene, Reduces the Increase in Saliva 3-Methoxy-4-Hydroxyphenylglycol After the "Trier Social Stress Test" in Healthy Humans: A Randomized, Double-Blind, Placebo-Controlled Cross-Over Study | 2018 | Ohara, Kazuaki; Misaizu, Akane; Kaneko, Yuji; Fukuda, Takafumi; Miyake, Mika; Miura, Yutaka; Okamura, Hisayoshi; Yajima, Jumpei; Tsuda, Akira | Not applicable based on title |
| Digit ratio (2D:4D) and salivary testosterone, oestradiol and cortisol levels under challenge: Evidence for prenatal effects on adult endocrine responses | 2015 | Crewther, Blair; Cook, Christian; Kilduff, Liam; Manning, John | Not applicable based on title |
| Stress related changes during a half marathon in master endurance athletes | 2015 | Piacentini, M F; Minganti, C; Ferragina, A; Ammendolia, A; Capranica, L; Cibelli, G | Not applicable based on title |
| Association between hormonal status, stress, recovery, and motivation of paralympic swimmers | 2020 | Rosa, Jo„o Paulo Pereira; Silva, Andressa; Rodrigues, Dayane Ferreira; Menslin, Rui; Ara˙jo, Leonardo Tomasello; Vital, Roberto; Tufik, Sergio; Stieler, Eduardo; de Mello, Marco Tulio | Not applicable based on title |
| Household illness is the strongest predictor of upper respiratory tract symptom risk in elite rugby union players | 2021 | Keaney, Lauren C; Kilding, Andrew E; Merien, Fabrice; Shaw, David M; Borotkanics, Robert; Dulson, Deborah K | Not applicable based on title |
| Lactobacilli reduce chemokine IL-8 production in response to TNF-± and Salmonella challenge of Caco-2 cells | 2013 | Ren, Da-Yong; Li, Chang; Qin, Yan-Qing; Yin, Rong-Lan; Du, Shou-Wen; Ye, Fei; Liu, Hong-Feng; Wang, Mao-Peng; Sun, Yang; Li, Xiao; Tian, Ming-Yao; Jin, Ning-Yi | Not applicable based on title |
| No Effect of a Ketone Monoester on Markers of Stress and Performance in a Live-Burn Search and Rescue in Firefighters | 2022 | Waldman, Hunter S; Bryant, Andrea R; Shepherd, Brandon D; Egan, Brendan; McAllister, Matthew J | Not applicable based on title |
| Immunological responses to overreaching in cyclists | 2003 | Halson, Shona L; Lancaster, Graeme I; Jeukendrup, Asker E; Gleeson, Michael | Not applicable based on title |
| Four Days of Blueberry Powder Supplementation Lowers the Blood Lactate Response to Running But Has No Effect on Time-Trial Performance | 2019 | Brandenburg, Jason P; Giles, Luisa V | Not applicable based on title |
| Prospective memory under acute stress: The role of (output) monitoring and ongoing-task demands | 2019 | Mˆschl, Marcus; Walser, Moritz; Surrey, Caroline; Miller, Robert | Not applicable based on title |
| Effects of operational assessment of the 4:4 and 4:4/6:6 watch systems on sleepiness, fatigue, and stress responses during patrolling on a navy missile patrol boat | 2022 | Myllyl‰, Mikko; Kyrˆl‰inen, Heikki; Ojanen, Tommi; Ruohola, Juha-Petri; Heinonen, Olli J; Vahlberg, Tero; Parkkola, Kai I | Not applicable based on title |
| Altitude, exercise and immune function | 2005 | Mazzeo, Robert S | Not applicable based on title |
| Effect of the combination of ginseng, oriental bezoar and glycyrrhiza on autonomic nervous activity and immune system under mental arithmetic stress | 2008 | Zheng, Aisong; Moritani, Toshio | Not applicable based on title |
| Cannabinoid polymorphisms interact with plasma endocannabinoid levels to predict fear extinction learning | 2021 | Ney, Luke J; Matthews, Allison; Hsu, Chia-Ming Ken; Zuj, Daniel V; Nicholson, Emma; Steward, Trevor; Nichols, David; Graham, Bronwyn; Harrison, Ben; Bruno, Raimondo; Felmingham, Kim | Not applicable based on title |
| Psychological state during pregnancy is associated with sleep quality: preliminary findings from MY-CARE cohort study | 2021 | Teoh, Ai Ni; Kaur, Satvinder; Mohd Shukri, Nurul Husna; Shafie, Siti Raihanah; Ahmad Bustami, Normina; Takahashi, Masaki; Lim, Pei Jean; Shibata, Shigenobu | Not applicable based on title |
| Effects of aerobic exercise on uric acid, total antioxidant activity, oxidative stress, and nitric oxide in human saliva | 2005 | Gonz·lez, David; Marquina, RamÛn; RondÛn, Norelis; Rodriguez-Malaver, Antonio J; Reyes, Rafael | Not applicable based on title |
| The role of oxytocin signaling in depression and suicidality in returning war veterans | 2021 | Warrener, Corinne D; Valentin, Edward M; Gallin, Camilla; Richey, Lynnet; Ross, Deanna B; Hood, Chelsea J; Lori, Adriana; Cubells, Joseph; Rauch, Sheila A M; Rilling, James K | Not applicable based on title |
| Salivary Alpha-Amylase and Behavior Reaction in Acute Stress and the Impact of Tridimensional Personality | 2005 | Ma, Lei; Wan, Jing; Shen, Xiaoyan | Not applicable based on title |
| Exploring the relationship between meaningful conditioned pain modulation and stress system reactivity in healthy adults following exposure to the cold pressor task | 2022 | Lukacs, Michael J; Melling, C W James; Walton, David M | Not applicable based on title |
| Loss of nocturnal dipping pattern of skin sympathetic nerve activity during and following an extended-duration work shift in residents in training | 2021 | Chen, Mu; Sun, Jian; Chen, Tai-Zhong; Xu, Dong-Zhu; Wan, Juyi; Wang, Qunshan; Li, Yi-Gang | Not applicable based on title |
| Predictors of upper respiratory tract symptom risk: Differences between elite rugby union and league players | 2021 | Keaney, Lauren Catherine; Kilding, Andrew E; Merien, Fabrice; Shaw, David M; Borotkanics, Robert J; Cupples, Balin; Dulson, Deborah K | Not applicable based on title |
| Salivary Hsp72 does not track exercise stress and caffeine-stimulated plasma Hsp72 responses in humans | 2011 | Fortes, Matthew B; Whitham, Martin | Not applicable based on title |
| Evaluation of the effectiveness of malaria vector control measures in urban settings of Dakar by a specific anopheles salivary biomarker | 2013 | Drame, Papa Makhtar; Diallo, Abdoulaye; Poinsignon, Anne; Boussari, Olayide; Dos Santos, Stephanie; Machault, Vanessa; Lalou, Richard; Cornelie, Sylvie; LeHesran, Jean-Yves; Remoue, Franck | Not applicable based on title |
| Timing of Preexercise Partial-Body Cryotherapy Exposure to Promote Jump Performance | 2022 | Partridge, Emily M; Cooke, Julie; McKune, Andrew J; Pyne, David B | Not applicable based on title |
| Mobile phones and head tumours The discrepancies in cause-effect relationships in the epidemiological studies - how do they arise? | 2011 | Levis, Angelo G; Minicuci, Nadia; Ricci, Paolo; Gennaro, Valerio; Garbisa, Spiridione | Not applicable based on title |
| Acute physiological responses to strongman training compared to traditional strength training | 2016 | Harris, Nigel K; Woulfe, Colm J; Wood, Matthew R; Dulson, Deborah K; Gluchowski, Ashley K; Keogh, Justin B | Not applicable based on title |
| A pilot study: the relationship between salivary MCP-1 and IgA, and exercise performance in long-distance runners and sprinters | 2022 | Uchida, Masataka; Suga, Tadashi; Terada, Masafumi; Isaka, Tadao | Not applicable based on title |
| The effect of preseason training on mucosal immunity in male basketball players | 2011 | Azarbayjani, M; Nikbakht, H; Rasaee, M J | Not applicable based on title |
| Impact of intense training and rapid weight changes on salivary parameters in elite female Taekwondo athletes | 2011 | Tsai, M-L; Ko, M-H; Chang, C-K; Chou, K-M; Fang, S-H | Not applicable based on title |
| Menstrual phase and the vascular response to acute resistance exercise | 2018 | Augustine, Jacqueline A; Nunemacher, Kayla N; Heffernan, Kevin S | Not applicable based on title |
| Testosterone and dihydrotestosterone changes in male and female athletes relative to training status | 2021 | Cook, Christian J; Crewther, Blair T; Kilduff, Liam P; Agnew, Linda L; Fourie, Phillip; Serpell, Benjamin G | Not applicable based on title |
| Use of stress-hormone levels and habitat selection to assess functional connectivity of a landscape for an amphibian | 2012 | Janin, AgnËs; LÈna, Jean-Paul; Deblois, Sandrine; Joly, Pierre | Not applicable based on title |
| Improvement of sprint performance in wheelchair sportsmen with caffeine supplementation | 2016 | Graham-Paulson, Terri S; Perret, Claudio; Watson, Phil; Goosey-Tolfrey, Victoria L | Not applicable based on title |
| Different placebos, different mechanisms, different outcomes: lessons for clinical trials | 2015 | Benedetti, Fabrizio; Dogue, Sara | Not applicable based on title |
| Genetic variation in catechol-O-methyltransferase is associated with individual differences in conditioned pain modulation in healthy subjects | 2021 | Korczeniewska, Olga A; Kuo, Fengshen; Huang, Ching-Yu; Nasri-Heir, Cibele; Khan, Junad; Benoliel, Rafael; Hirschberg, Craig; Eliav, Eli; Diehl, Scott R | Not applicable based on title |
| Experimental use of an acrolein-based primer as collagen cross-linker for dentine bonding | 2005 | Maravic, Tatjana; Breschi, Lorenzo; Comba, Allegra; Cunha, Sandra Ribeiro; Angeloni, Valeria; Nucci, Cesare; Hebling, Josimeri; Pashley, David; Tay, Franklin; Mazzoni, Annalisa | Not applicable based on title |
| Salivary free insulin-like growth factor-i levels: effects of an acute physical exercise in athletes | 2009 | Antonelli, G; Gatti, R; Prearo, M; De Palo, E F | Not applicable based on title |
| Salivary nitric oxide and alpha-amylase as indexes of training intensity and load | 2013 | Diaz, M M; Bocanegra, O L; Teixeira, R R; Soares, S S; Espindola, F S | Not applicable based on title |
| Cytotoxicity of elastomeric power chains in artificial saliva: an in vitro study | 2015 | Halimi, Abdelali; Azeroual, Mohamed-Faouzi; Eddimani, Latifa; Natiq, Abdelhafid; Bakri, Youssef; Zaoui, Fatima | Not applicable based on title |
| Oral neutrophil responses to acute prolonged exercise may not be representative of blood neutrophil responses | 2015 | Davison, Glen; Jones, Arwel Wyn | Not applicable based on title |
| The effect of exercise on innate mucosal immunity | 2010 | West, N P; Pyne, D B; Kyd, J M; Renshaw, G M; Fricker, P A; Cripps, A W | Not applicable based on title |
| Effects of Tomato Juice Intake on Salivary 8-Oxo-dG Levels as Oxidative Stress Biomarker after Extensive Physical Exercise | 2020 | Pour Khavari, Ali; Haghdoost, Siamak | Not applicable based on title |
| Technical nuances of exposing rat common carotid arteries for practicing microsurgical anastomosis | 2018 | Tayebi Meybodi, Ali; Aklinski, Joseph; Gandhi, Sirin; Lawton, Michael T; Preul, Mark C | Not applicable based on title |
| A noninvasive and qualitative bioluminescent assay for express diagnostics of athletes' responses to physical exertion | 2021 | Kratasyuk, Valentina A; Stepanova, Lyudmila V; Ranjan, Rajeev; Sutormin, Oleg S; Pande, Shubhra; Zhukova, Galina V; Miller, Olga M; Maznyak, Natalya V; Kolenchukova, Oksana A | Not applicable based on title |
| Exercise and the hypothalamo-pituitary-adrenal axis | 2005 | Duclos, Martine; Tabarin, Antoine | Not applicable based on title |
| Spinal cord injury: known and possible influences on the immune response to exercise | 2005 | Leicht, Christof; Goosey-Tolfrey, Vicky; Bishop, Nicolette | Not applicable based on title |
| Alteration in salivary cortisol and interleukin-6 levels during two different intensities of acute aerobic exercise | 2022 | HUNTULA, SOONTARAPORN; PUNSAWAD, CHUCHARD; LALERT, LADDAWAN | Not applicable based on title |
| EFFECT OF HIGH-INTENSITY STRENGTH EXERCISE ON COGNITIVE PERFORMANCE / EFECTO DE UN EJERCICIO DE FUERZA DE ALTA INTENSIDAD SOBRE EL RENDIMIENTO COGNIVITO | 2021 | Bermejo, J. L.; Marco-AhullÛ, A.; Ribeiro do Couto, B.; Monfort-Torres, G.; Pardo, A. | Not applicable based on title |
| A Multifactorial Assessment of Elite Paratriathletes' Response to 2 Weeks of Intensified Training | 2019 | Stephenson, Ben T.; Leicht, Christof A.; Tolfrey, Keith; Goosey-Tolfrey, Victoria L. | Not applicable based on title |
| Salivary markers of stress and physical activities: a systematic review / Marcadores salivares de estrÈs y actividades fÌsicas: una revisiÛn sistem·tica | 2021 | Vasconcellos de Souza, I.; Pereira Ribeiro, L. C.; Andrade Dantas, K. B.; Martin Dantas, E. H. | Not applicable based on title |
| Physical fitness modulates mucosal immunity and acceleration capacity during a short-term training period in elite youth basketball players | 2020 | Valvassori, R.; Saldanha Aoki, M.; Conte, D.; Drago, G.; Moreira, A. | Not applicable based on title |
| Psychosomatic conditions in university students related to melatonin biological rhythm and cortisol morning levels | 2020 | Nagane, Mitsuo; Oyama, Yoshinori; Suge, Rie; Watanabe, Shu-Ichi | Not applicable based on title |
| Pre and post-competition cortisol in athletes from the brazilian confederation of aquatic sports | 2018 | SILVA, GLAUBER CASTELO BRANCO; JUNIOR, JOS… ROBERTO ANDRADE DO NASCIMENTO; CORTEZ, ANT‘NIO CARLOS LEAL; DI MASI, FABRIZIO; DANTAS, EST…LIO HENRIQUE MARTIN; DE MELO, GISLANE FERREIRA | Not applicable based on title |
| The Effect of Physical And Mental Stress on the Heart Rate, Cortisol and Lactate Concentrations in Rock Climbers | 2018 | Magiera, Artur; Roczniok, Robert; Sadowska-Krpa, Ewa; Kempa, Katarzyna; Placek, Oskar; Mostowik, Aleksandra | Included |
| Cortisol and Anxiety Responses in Female Collegiate Golfers during Qualifying and Competition Play | 2019 | Madrigal, Leilani A.; Wilson, Patrick B. | Not applicable based on title |
| Salivary Cortisol Analysis In Collegiate Female Lacrosse Athletes | 2021 | Figueroa, Yvette L.; Carter, Jenna L.; Mathews, Stephanie L.; Bunn, Jennifer A. | age |
| Changes in Salivary Hormones Concentration during the Preparation and Competition Period in Olympic Weightlifters | 2017 | Kov· , Milan; Laczo, Eugen; Vajda, Matej; Cihov·, Iveta; Babkov·, Jaroslava | Not applicable based on title |
| Effects of Water Immersion on Mucosal Immune Defense after Acute Resistance Exercise | 2019 | Hironaga ITO, ; Nobuhiko EDA, ; Hideya NAKATSUKA, ; Norihide YAMASHITA, ; Takao AKAMA, | Not applicable based on title |
| Workload and cortisol levels in helicopter combat pilots during simulated flights / Carga mental y niveles de cortisol en pilotos de helicÛptero de combate en vuelos simulados | 2016 | GarcÌa-Mas, A.; Ortega, E.; Ponseti, J.; de Teresa, C.; C·rdenas, D. | Not applicable based on title |
| Cortisol and Alpha-amylase changes during an Ultra-Running Event | 2017 | DENEEN, WHITNEY P.; JONES, ALEXIS B. | Not applicable based on title |
| Water cutting, stress hormones and salivary immunoalobulins in wrestlers | 2011 | Cieslak, Thomas; Muir, Cameron; Corbett, Lauren; Ludwa, Izabella A.; Klentrou, Panagiota | Not applicable based on title |
| Weekly Salivary Biomarkers across a Season for Elite Men Collegiate Basketball Players | 2018 | ANDRE, MATTHEW J.; FRY, ANDREW C.; LUEBBERS, PAUL E.; HUDY, ANDREA; DIETZ, PARTRICIA R.; CAIN, GLENN J. | Not applicable based on title |
| Effects of Probiotic (Bifidobacterium longum 35624) Supplementation on Exercise Performance, Immune Modulation, and Cognitive Outlook in Division I Female Swimmers | 2018 | Carbuhn, Aaron F.; Reynolds, Shelby M.; Campbell, Clark W.; Bradford, Luke A.; Deckert, Jake A.; Kreutzer, Andreas; Fry, Andrew C. | Not applicable based on title |
| Evaluation of an Australian combat ration pack as a sole nutrition source during 23 days of military adventurous training in the tropics | 2003 | Booth C, ; Coad R, ; Roberts W, | Not applicable based on title |
| Effects of prolonged running in the heat and cool environments on selected physiological parameters and salivary lysozyme responses | 2017 | Ibrahim, Nur S.; Chen, Chee K.; Ayub, Ayunizma; Muhamad, Ayu S. | Not applicable based on title |
| Influence of exercise time of day on salivary melatonin responses | 2019 | Carlson, Lara A; Pobocik, Kaylee M; Lawrence, Michael A; Brazeau, Daniel A; Koch, Alexander J | Not applicable based on title |
| Sex-Based Effects on Immune Changes Induced by a Maximal Incremental Exercise Test in Well-Trained Swimmers | 2014 | Morgado, Jose P.; Monteiro, Cristina P.; Matias, Catarina N.; Alves, Francisco; Pessoa, Pedro; Reis, Joana; Martins, F·tima; Seixas, Teresa; Laires, Maria J. | Not applicable based on title |
| Psychobiologic responses to 4 days of increased training and recovery in cyclists / Reponses psychobiologiques a quatre jours d ' entrainement augmente et a la recuperation chez des cyclistes | 2002 | Filaire, E.; Legrand, B.; Bret, K.; Sagnol, M.; Cottet-Emard, J.M.; Pequignot, J.M. | age |
| The resting salivary antimicrobial proteins and cortisol concentration in wrestlers during 12-week training | 2015 | Trochimiak, Teresa; H¸bner-Wozniak, El\|bieta; Tomaszewski, PaweB | Not applicable based on title |
| Effects of caffeine, sleep loss, and stress on cognitive performance and mood during US Navy SEAL training | 2002 | Lieberman, Harris R.; Tharion, William J.; Shukitt-Hale, Barbara; Speckman, Karen L.; Tulley, Richard | Not applicable based on title |
| Relationship between biological markers and psychological states in elite basketball players across a competitive season | 2012 | Robazza, Claudio; Gallina, Sabina; D'Amico, Maria Angela; Izzicupo, Pascal; Bascelli, Adriana; Di Fonso, Alessia; Mazzaufo, Claudio; Capobianco, Andrea; Di Baldassarre, Angela | Not applicable based on title |
| Effects of a Simulated Altitude Device on Endurance Performance and Mucosal Immunity | 2014 | Blazek, Alisa D.; Anderson, Paul J.; Brichler, Jennifer G.; Slawinski, Michelle K.; Rose, Mark T.; Kirby, Timothy E.; Swain, Carmen B. | Performance not measured |
| Effect of a Mental Training Program on Salivary Cortisol in Volleyball Players | 2014 | Weigert Coelho, Ricardo; Kuczynski, Katia Maria; Paes, Mayara Juliana; de Lima Greboggy, DÍnis; Bertoldo dos Santos, Priscilla; Souza Rosa, Ana Paula Dalazuana; Facco Stefanello, Joice Mara | Not applicable based on title |
| Salivary pH increases after jump exercises in hypoxia | 2014 | Juli‡-S·nchez, S.; ¡lvarez-Herms, J.; Gatterer, H.; Burtscher, M.; PagËs, T.; Viscor, G. | Included |
| Immunological responses to overreaching in cyclists / Reponses immunologiques a une periode de surentrainement chez des cyclistes | 2003 | Halson, S.L.; Lancaster, G.I.; Jeukendrup, A.E.; Gleeson, M. | Not applicable based on title |
| Nutritional strategies to counter stress to the immune system in athletes, with special reference to football | 2006 | Nieman, David C; Bishop, Nicolette C | Not applicable based on title |
| Evidence of a Non-Linear Dose-Response Relationship between Training Load and Stress Markers in Elite Female Futsal Players | 2014 | Milanez, Vinicius F.; Ramos, Solange P.; Okuno, Nilo M.; Boullosa, Daniel A.; Nakamura, Fabio Y. | Not applicable based on title |
| Testosterone and Cortisol Responses to Five High-Intensity Functional Training Competition Workouts in Recreationally Active Adults | 2018 | Mangine, Gerald T.; Van Dusseldorp, Trisha A.; Feito, Yuri; Holmes, Alyssa J.; Serafini, Paul R.; Box, Allyson G.; Gonzalez, Adam M. | Not applicable based on title |
| Gender differences in the pre-competition temporal patterning of anxiety and hormonal responses | 2004 | Thatcher, J.; Thatcher, R.; Dorling, D. | Not applicable based on title |
| Salivary alpha-amylase, heart rate and heart-rate variability in response to an experimental model of competitive stress in volleyball players | 2014 | PETROV, L. A.; BOZHILOV, G.; ALEXANDROVA, A. V.; MUGANDANI, S. C.; DJAROVA, T. G. | Not applicable based on title |
| Psychophysiological Effects of Competitive Stress on Swimming Coaches | 2004 | Loupos, Dimitris; Tsalis, Giorgos; Barkoukis, Vassilis; Semoglou, Kleoniki; Mougios, Vassilis | Not applicable based on title |
| Planned Missing Data Designs for Spline Growth Models in Salivary Cortisol Research | 2013 | Hogue, CandaceM.; Pornprasertmanit, Sunthud; Fry, MaryD.; Rhemtulla, Mijke; Little, ToddD. | Not applicable based on title |
| The effect of exercise mode and intensity of sub-maximal physical activities on salivary testosterone to cortisol ratio and ±-amylase in young active males | 2011 | AZARBAYJANI, MOHAMMAD ALI; FATOLAHI, HOSEYN; RASAEE, MOHAMMAD JAVAD; PEERI, MAGHSOD; BABAEI, ROHOLAH | Not applicable based on title |
| Stress recovery during an ocean boat race | 2004 | Gunnarsson, Lars-Gunnar; B‰ck, Hans; Jones, Ian; Olsson, Tommy | Not applicable based on title |
| The Influence of High Intensity Interval Training on the Salivary Cortisol Response to a Psychological Stressor and Mood State in Non-Sedentary College Students | 2013 | Ormsbee, Michael J.; Kinsey, Amber W.; Minwook Chong, ; Friedman, Heather S.; Dodge, Tonya; Fehling, Patricia C. | Not applicable based on title |
| Psychophysiological Stress in Under-17 Soccer Players | 2014 | Dos Santos, Priscilla Bertoldo; Kuczynski, Katia Maria; do Amaral Machado, Thais; Vecchi Osiecki, Ana Cl·udia; Facco Stefanello, Joice Mara | Not applicable based on title |
| The effects of a nucleotide supplement on the immune and metabolic response to short term, high intensity exercise performance in trained male subjects | 2007 | McNaughton, L.; Bentley, D.; Koeppel, P. | Not applicable based on title |
| Evaluation of salivary cortisol as a biomarker of self-reported mental stress in field studies | 2004 | Hjortskov, Nis; Garde, Anne Helene; ÿrbÊk, Palle; Hansen, ≈se Marie | Not applicable based on title |
| MEASUREMENT OF HEART RATE VARIABILITY AND SALIVARY CORTISOL LEVELS IN BEGINNER SCUBA DIVERS | 2011 | Coetzee, Nicoleen | Not applicable based on title |
| MUCOSAL IgA RESPONSE TO INTENSE INTERMITTENT EXERCISE IN HEALTHY MALE AND FEMALE ADULTS | 2004 | Engels, Hermann-J.; Fahlman, Marianne M.; Morgan, Amy L.; Formolo, Lance R. | Not applicable based on title |
| Cortisol, DHEA, performance and training in elite swimmers / Cortisol, DHEA, performance et entrainement chez des nageurs de haut niveau | 2002 | Chatard, J.C.; Atlaoui, D.; Lac, G.; Duclos, M.; Hooper, S.; Mackinnon, L. | Not applicable based on title |
| RESPONSES OF SALIVA TESTOSTERONE, CORTISOL, AND TESTOSTERONE-TO-CORTISOL RATIO TO A TRIATHLON IN YOUNG AND MIDDLE-AGED MALES | 2005 | Chang, Chen-Kang; Tseng, H.F.; Tan, H.F.; Hsuuw, Y.D.; Lee-Hsieh, J. | Not applicable based on title |
| Anti-stress effect of yoga-type breathing: modification of salivary cortisol, heart rate and blood pressure following a step-climbing exercise | 2002 | Monnazzi, Paola; Leri, Oriana; Guizzardi, Liliana; Mattioli, Domenico; Patacchioli, F. R. | Not applicable based on title |
| Saliva as a Marker of Competition Stress in Male Handball Players | 2008 | ObmiDski, Zbigniew; Eliasz, Jerzy; Rojek, Jolanta | Not applicable based on title |
| Tennis, incidence of URTI and salivary IgA / Frequence des affections respiratoires des voies superieures et de l ' immunoglobine secretoire, en tennis | 2003 | Novas, A.M.P.; Rowbottom, D.G.; Jenkins, D.G. | Not applicable based on title |
| THE ACUTE RESPONSE AND RECOVERY TIME-COURSE OF AUTONOMIC AND PERFORMANCE PARAMETERS FOLLOWING VARIOUS TRAINING INTENSITIES IN HIGHLY-TRAINED ROWERS | 2017 | Holt, Ana C.; Plews, Daniel J.; Oberlin-Brown, Katherine T.; Merien, Fabrice; Kilding, Andrew E. | Not applicable based on title |
| Salivary prekallikrein output during the ranger training-induced stress | 2004 | Tagashira, Sumito; Yamaguchi, Keiji; Matsunaga, Tsunenori; Toda, Kazuo; Hayashi, Yoshihiko | Not applicable based on title |
| Psychophysiological responses in the pre-competition period in elite soccer players | 2008 | Alix-Sy, DÈborah; Le Scanff, Christine; Filaire, Edith | Not applicable based on title |
| ORAL ADMINISTRATION OF THE PROBIOTIC LACTOBACILLUS FERMENTUM VRI-003 AND MUCOSAL IMMUNITY IN ENDURANCE ATHLETES | 2008 |  | Not applicable based on title |
| Application of acute pre-exercise partial-body cryotherapy promotes jump performance, salivary ±-amylase and athlete readiness | 2022 | Partridge, Emily M; Cooke, Julie; McKune, Andrew J; Pyne, David B | Not applicable based on title |
| Maturity-Associated Variations in Resistance Exercise-Induced Hormonal Responses in Young Male Athletes | 2022 | Sekine, Yuta; Hirose, Norikazu | Not applicable based on title |
| Stress Effects: A Study of Salivary Cortisol Levels in Third-year Medical Students | 2011 | Tseng, Teresa; Iosif, Ana-Maria; Seritan, Andreea L. | Not applicable based on title |
| The shape of stress: the use of frequent sampling to measure temporal variation in S-IgA levels during acute stress | 2007 | Benham, Grant | Not applicable based on title |
| Change in stress levels following mindfulness-based stress reduction in a therapeutic community | 2003 | Marcus MT, ; Fine PM, ; Moeller FG, ; Khan MM, ; Pitts K, ; Swank PR, ; Liehr P, | Not applicable based on title |
| Cardiorespiratory fitness and oxidative stress: effect of acute maximal aerobic exercise in children and adolescents | 2011 | Benitez-Sillero, J D; Perez-Navero, J L; Tasset, I; Guillen-Del Castillo, M; Gil-Campos, M; Tunez, I | Not applicable based on title |
| A longitudinal study of changes in salivary sIgA during pregnancy and the puerperal period, focusing on differences between vaginal birth and cesarean section | 2008 | Shitami C, ; Tamaru M, ; Takenaka K, ; Tanaka Y, | Not applicable based on title |
| Undergoing venipuncture in healthcare education: the psycho-biological effect on students | 2008 | Sarid O, ; Anson O, ; Schwartz D, ; Yaari A, | Not applicable based on title |
| Effectiveness of relaxation technique at the workplace evaluated by autonomic nervous activity and psychological mood | 2011 | Oshima, Norihito; Numao, Shinji; Chinzei, Mieko | Not applicable based on title |
| Monitoring stress tolerance and occurrences of upper respiratory illness in basketball players by means of psychometric tools and salivary biomarkers | 2011 | Moreira, Alexandre; Arsati, Franco; de Oliveira Lima-Arsati, Ynara Bosco; Simıes, Antonio Carlos; de Ara˙jo, Vera Cavalcanti | Not applicable based on title |
| Association between endocrine markers, accumulated workload, and fitness parameters during a season in elite young soccer players | 2021 | Nobari, Hadi; Mainer-Pardos, Elena; Adsuar, JosÈ Carmelo; Franco-GarcÌa, Juan Manuel; Rojo-Ramos, Jorge; Cossio-BolaÒos, Marco Antonio; Urzua Alul, Luis; PÈrez-GÛmez, Jorge | Not applicable based on title |
| Muscle damage, endocrine, and immune marker response to a soccer match | 2012 | Thorpe, Robin; Sunderland, Caroline | Not applicable based on title |
| The interplay between plasma hormonal concentrations, physical fitness, workload and mood state changes to periods of congested match play in professional soccer players | 2020 | Saidi, Karim; Ben Abderrahman, Abderraouf; Boullosa, Daniel; Dupont, GrÈgory; Hackney, Anthony C; Bideau, Benoit; Pavillon, Thomas; Granacher, Urs; Zouhal, Hassane | Not applicable based on title |
| Effect of a congested match schedule on immune-endocrine responses, technical performance and session-RPE in elite youth soccer players | 2016 | Moreira, Alexandre; Bradley, Paul; Carling, Christopher; Arruda, Ademir Felipe Schultz; Spigolon, Leandro M P; Franciscon, Clovis; Aoki, Marcelo Saldanha | Not applicable based on title |
| Alteration of immune function in women collegiate soccer players and college students | 2004 | Putlur, Praveen; Foster, Carl; Miskowski, Jennifer A; Kane, Melissa K; Burton, Sara E; Scheett, Timothy P; McGuigan, Michael R | Not applicable based on title |
| The effects of successive soccer matches on the internal match load, stress tolerance, salivary cortisol and jumping performance in youth soccer players | 2021 | Pinto, Julio Cesar Barbosa de Lima; de Oliveira, Romerito SÛstenes Canuto; Galv„o-Coelho, Nicole Leite; de Almeida, Raissa NÛbrega; Moreira, Alexandre; Mortatti, Arnaldo Luis | Not applicable based on title |
| The effects of sleep extension on sleep, performance, immunity and physical stress in rugby players | 2005 | Swinbourne, R; Miller, J; Smart, D; Dulson, DK; Gill, N | Not applicable based on title |
| Effects of dawn simulation on markers of sleep inertia and post-waking performance in humans | 2014 | Thompson, Andrew; Jones, Helen; Gregson, Warren; Atkinson, Greg | Not applicable based on title |
| Saliva flow rate, total protein concentration and osmolality as potential markers of whole body hydration status during progressive acute dehydration in humans | 2005 | Walsh, NP; Montague, JC; Callow, N; Rowlands, AV | Included |
| Stress Biomarkers, Mood States, and Sleep during a Major Competition: "Success" and "Failure" Athlete's Profile of High-Level Swimmers | 2016 | Chennaoui, Mounir; Bougard, ClÈment; Drogou, Catherine; Langrume, Christophe; Miller, Christian; Gomez-Merino, Danielle; Vergnoux, FrÈdÈric | Not applicable based on title |
| Saliva indices track hypohydration during 48 h of fluid restriction or combined fluid and energy restriction | 2005 | Oliver, SJ; Laing, SJ; Wilson, S; Bilzon, JLJ; ..., | Not applicable based on title |
| Effect of long-haul transmeridian travel on recovery and performance in international level swimmers | 2022 | Rossiter, Antonia; Comyns, Thomas M.; Powell, Cormac; Nevill, Alan M.; Warrington, Giles D. | Not applicable based on title |
| Circadian effects on performance and effort in collegiate swimmers | 2005 | Anderson, A; Murray, G; Herlihy, M; Weiss, C; ..., | Not applicable based on title |
| The effect of basketball matches on salivary markers: A systematic review | 2005 | Kamarauskas, P; Conte, D | Not applicable based on title |
| Competition stage influences perceived performance but does not affect rating of perceived exertion and salivary neuro-endocrine-immune markers in elite young basketball players | 2018 | Arruda, Ademir Felipe Schultz de; Aoki, Marcelo Saldanha; Paludo, Ana Carolina; Drago, Gustavo; Moreira, Alexandre | Not applicable based on title |
| Effect of lavender aroma on salivary endocrinological stress markers | 2005 | Toda, M; Morimoto, K | Not applicable based on title |
| Training Load and Player Monitoring in High-Level Football: Current Practice and Perceptions | 2016 | Akenhead, Richard; Nassis, George P | Not applicable based on title |
| Salivary molecules as indicators of hydration status | 2005 | STEFANELLI, ML; BUTTI, G; IRIART, M; ..., | Included |
| Understanding the fatigue-recovery cycle in team sport athletes | 2005 | Hogarth, LW; Burkett, BJ; ..., | Not applicable based on title |
| Investigation of the relationship between training load, monitoring markers of recovery, injury, and illness in elite team sports | 2005 | Tiernan, C | Not applicable based on title |
| Sustained sleep restriction reduces resistance exercise quality and quantity in females | 2022 | Knowles, Olivia E; Drinkwater, Eric J; Roberts, Spencer S H; Alexander, Sarah E; Abbott, Gavin; Garnham, Andrew; Lamon, SÈverine; Aisbett, Brad | Chronic/Prolonged stressor |
| Recovery in Soccer | 2012 | NÈdÈlec, Mathieu; McCall, Alan; Carling, Chris; Legall, Franck; Berthoin, Serge; Dupont, Gregory | Not applicable based on title |
| Effects of long-haul travel on recovery and performance in elite athletes A systematic review | 2005 | Rossiter, A; Warrington, GD; ..., | Not applicable based on title |
| Hydration biomarkers during daily life: recent advances and future potential | 2005 | Armstrong, LE | Not applicable based on title |
| Focus: The Science of Stress: Virtual Reality Based Active Shooter Training Drill Increases Salivary and Subjective Markers of Stress | 2005 | McAllister, MJ; Martaindale, MH; ..., | Not applicable based on title |
| The effect of sex hormones on sleep spindles and cognitive performance | 2005 | Hˆdlmoser, K; Herzog, S; ..., | Not applicable based on title |
| Quantitative Estimation of Total Body Water Loss During Physical Exercise | 2005 | Ring, M | Not applicable based on title |
| Effect of varied recovery interventions on markers of psychophysiological stress in professional rugby union | 2015 | Lindsay, Angus; Lewis, John; Gill, Nicholas; Gieseg, Steven P; Draper, Nick | Not applicable based on title |
| Quantitative Sch‰tzung des Verlustes an Gesamtkˆrperwasser w‰hrend kˆrperlicher Bet‰tigung | 2005 | Ring, M | Not applicable based on title |
| Hydration Status, Salivary Biomarkers of Stress, Body Composition, and Performance in Response to a 4-Week Pre-Season Training Cycle in Division 1 Basketball & | 2005 | Ayscue, J | Not applicable based on title |
| Monitoring training load (Stuart Cormack and Aaron Coutts) | 2005 | Joyce, D; Lewindon, D | Not applicable based on title |
| A novel role of probiotics in improving host defence of elite rugby union athlete: A double blind randomised controlled trial | 2019 | Pumpa, Kate L; McKune, Andrew J; Harnett, Joanna | Not applicable based on title |
| Neuromuscular, endocrine, and perceptual recovery after a youth American football game | 2005 | Davis, JK; Wolfe, AS; Basham, SA; ..., | Not applicable based on title |
| Active shooter training drill increases blood and salivary markers of stress | 2020 | McAllister, Matthew J; Martaindale, M Hunter; RenterÌa, Liliana I | Not applicable based on title |
| SPECIFIC INDICATORS OF DEHYDRATION IN COMBAT SPORTS | 2005 | Trivi, T | None peer reviewed article |
| Associations between attention and implicit associative learning in healthy adults: the role of cortisol and salivary alpha-amylase responses to an acute stressor | 2005 | Becker, L; Rohleder, N | Not applicable based on title |
| HUMAN PHYSIOLOGY AND PSYCHOLOGY FACTORS: 100 B ILEWG EURO-MOON-MARS MISSION | 2005 | Rai, B; Kaur, J; Foing, BH | Not applicable based on title |
| 88 The impact of workload and training status on salivary antimicrobial proteins following acute exercise | 2005 | Kunz, HE; Spielmann, G; Pistillo, M; Reed, J; ..., | Undefined stressor in criteria |
| Masticatory performance alters stress relief effect of gum chewing | 2015 | Nishigawa, Keisuke; Suzuki, Yoshitaka; Matsuka, Yoshizo | Not applicable based on title |
| & immersion performed on successive days on physical performance, muscle damage, and inflammatory, hormonal, and oxidative stress markers in volleyball players | 2005 | Freitas, VH de; Ramos, SP; Bara-Filho, MG; ..., | Not applicable based on title |
| From darkness to light: non-visual light effects can be modulated by optimizing light spectrum during nighttime and daytime | 2005 | Zeeuw, JL de | Not applicable based on title |
| The Effect of Several Small Time-zone Transitions on the Timing of Salivary Melatonin Onset | 2005 | Roach, GD; Fletcher, AA; Rodgers, M; Dawson, D | Not applicable based on title |
| Might salivary lysozyme be an indicator of prolonged intense training load in athletes? A preliminary study in adolescent male gymnasts | 2005 | V·Ha, P; JuYÌkov·, J; Bernacikov·, M; `ev Ìk, R; ..., | Not applicable based on title |
| More stress for the eastward travelling student athlete: a preliminary analysis | 2005 | Atala­, O; Gotshalk, LA; Queen, L; ..., | Not applicable based on title |
| Mood and cortisol responses following tryptophan-rich hydrolyzed protein and acute stress in healthy subjects with high and low cognitive reactivity to depression | 2009 | Firk, Christine; Markus, C Rob | Not applicable based on title |
| Sleep duration and timing in obsessive-compulsive disorder (OCD): evidence for circadian phase delay | 2020 | Coles, M E; Schubert, J; Stewart, E; Sharkey, K M; Deak, M | Not applicable based on title |
| Inadequate Sample Sizes in Studies of Athletic Performance at the 2012 ACSM Annual Meeting | 2005 | Pyne, DB; Hopkins, WG; Martin, DT | Not applicable based on title |
| Exogenous cortisol causes a shift from deliberative to intuitive thinking | 2016 | Margittai, Zsofia; Nave, Gideon; Strombach, Tina; van Wingerden, Marijn; Schwabe, Lars; Kalenscher, Tobias | Not applicable based on title |
| Exploring the short-term effects of low volume resistance exercise: Can resistance exercise priming improve subsequent markers of athletic performance? | 2005 | Harrison, P | Not applicable based on title |
| Load monitoring in elite paralympic athletes: implications for training and recovery | 2005 | Sinnott-O'Connor, C | Not applicable based on title |
| 0233 GABA: A Neural Marker of Resilience to Psychomotor Vigilance Impairment during Sleep Deprivation | 2005 | Satterfield, BC; Silveri, MM; Alkozei, A; Raikes, AC; ..., | Not applicable based on title |
| Nasal issues in athletes | 2013 | Navarro, Rodolfo R; Romero, Leigh; Williams, Kwani | Not applicable based on title |
| Salivary nitrite content, cognition and power in Mixed Martial Arts fighters after rapid weight loss: a case study | 2005 | CamarÁo, NF; Neto, IVS; Nascimento, DC; ..., | Not applicable based on title |
| Computational approaches for individual circadian phase prediction in field settings | 2020 | Stone, Julia E.; Postnova, Svetlana; Sletten, Tracey L.; Rajaratnam, Shantha M.W.; Phillips, Andrew J.K. | Not applicable based on title |
| Personal info | 2005 | HODOSY, J | Not applicable based on title |
| Limitations of salivary osmolality as a marker of hydration status | 2005 | Ely, BR; Cheuvront, SN; Kenefick, RW; Sawka, MN | Not applicable based on title |
| The relationship between lower-limb strength and match-related muscle damage in elite level professional European soccer players | 2015 | Owen, Adam; Dunlop, Gordon; Rouissi, Mehdi; Chtara, Moktar; Paul, Darren; Zouhal, Hassane; Wong, Del P | Not applicable based on title |
| New genetic model for predicting phenotype traits in sports | 2005 | Massidda, M; Scorcu, M; ..., | Not applicable based on title |
| Identifying Metabolites as Markers of Fatigue in Athletes | 2005 | George, CD | Not applicable based on title |
| Basal mild dehydration increase salivary cortisol after a friendly match in young elite soccer players | 2018 | Castro-Sepulveda, Mauricio; Ramirez-Campillo, Rodrigo; Abad-Colil, Felipe; Monje, Camila; PeÒailillo, Luis; Cancino, Jorge; Zbinden-Foncea, Hermann | age |
| Evidence and application of athlete monitoring programs in national collegiate athletic association women's soccer: a narrative review | 2005 | Ishida, A; Bazyler, CD; Sayers, AL; ..., | Not applicable based on title |
| Escape from a diving submarine simulator: Impacts of mindfulness differences on physio-biological responses and cognitive performances | 2005 | Trousselard, M | Not applicable based on title |
| CHANGES IN PSY CHOLOGICAL AND BIOCHEMICAL INDICES OF STRESS ACROSS A COMPETITIVE SEASON IN AGE-GROUP SWIMMERS | 2005 | Kerr, G; VanHeest, JL; Rodgers, CD | Not applicable based on title |
| Significant strength gains observed in rugby players after specific resistance exercise protocols based on individual salivary testosterone responses | 2005 | Beaven, CM; Cook, CJ; Gill, ND | Not applicable based on title |
| Science of Rugby | 2014 | Twist, Craig; Highton, Jamie | Not applicable based on title |
| Does caffeine ingestion influence mucosal immunity and performance in males during intermittent exercise in the heat? | 2005 | Marwah, A | Not applicable based on title |
| Evaluation of bathing with and without acute bouts of exercise at salivary levels of ±-amylase and IgA in men | 2005 | Harada, H; Yoshikawa, T; Yasuda, N | Not applicable based on title |
| Sleep loss during military training reduces testosterone in US army Rangers: a Two-Study series | 2005 | Mantua, J; Naylor, JA; Ritland, BM; ..., | Included |
| A year on the international space station: Implementing a long-duration biomedical research mission | 2005 | Charles, JB; Pietrzyk, RA | Not applicable based on title |
| Winter-summer difference in post-awakening salivary ±-amylase and sleepiness depending on sleep and melatonin | 2021 | Danilenko, Konstantin V; Kobelev, Evgenii; Zhanaeva, Svetlana Y; Aftanas, Lyubomir I | Not applicable based on title |
| Effects of a competitive wrestling season on body composition, endocrine markers, and anaerobic exercise performance in NCAA collegiate wrestlers | 2013 | Ratamess, Nicholas A; Hoffman, Jay R; Kraemer, William J; Ross, Ryan E; Tranchina, Christopher P; Rashti, Stefanie L; Kelly, Neil A; Vingren, Jakob L; Kang, Jie; Faigenbaum, Avery D | Not applicable based on title |
| Changes in Performance and Morning-Measured Responses in Sport Rock Climbers | 2019 | Magiera, Artur; Roczniok, Robert; Sadowska-Krpa, Ewa; Kempa, Katarzyna | Included |
| Associations between Saliva Alpha-Amylase, Heart Rate Variability, Saliva Cortisol and Cognitive Performance in Individuals at Ultra High-Risk for Psychosis | 2022 | Almstrup, FB; Nordentoft, M; Jensen, MA; Kristiansen, J; ..., | Not applicable based on title |
| Training load, physical performance, biochemical markers, and psychological stress during a short preparatory period in brazilian elite male volleyball players | 2019 | Horta, Thiago A G; Bara Filho, MaurÌcio G; Coimbra, Danilo R; Miranda, Renato; Werneck, Francisco Z | Not applicable based on title |
| Sleep and Performance during a Preseason in Elite Rugby Union Athletes | 2021 | Teece, Angus R; Argus, Christos K; Gill, Nicholas; Beaven, Martyn; Dunican, Ian C; Driller, Matthew W | Not applicable based on title |
| Time restricted feeding reduces inflammation and cortisol response to a firegrounds test in professional firefighters | 2021 | McAllister, Matthew J; Gonzalez, Andrew E; Waldman, Hunter S | Not applicable based on title |
| Postmatch recovery of physical performance and biochemical markers in team ball sports: a systematic review | 2018 | Doeven, Steven H; Brink, Michel S; Kosse, Silke J; Lemmink, Koen A P M | review |
| Salivary oxytocin, cognitive anxiety and self-confidence in pre-competition athletes | 2021 | La Fratta, Irene; Franceschelli, Sara; Speranza, Lorenza; Patruno, Antonia; Michetti, Carlo; D'Ercole, Paolo; Ballerini, Patrizia; Grilli, Alfredo; Pesce, Mirko | Not applicable based on title |
| Cervical transcutaneous vagal nerve stimulation (ctVNS) improves human cognitive performance under sleep deprivation stress | 2021 | McIntire, Lindsey K; McKinley, R Andy; Goodyear, Chuck; McIntire, John P; Brown, Rebecca D | Not applicable based on title |
| Recovery and Well-being in Sport and Exercise | 2021 | Coutts, Aaron J.; Crowcroft, Stephen; Kempton, Tom | Not applicable based on title |
| The Effects of Acute Stress on Multiple Object Tracking | 2005 | Almeida, CA | Not applicable based on title |
| Salivary antioxidants status following progressive aerobic exercise: what are the differences between waterpipe smokers and non-smokers? | 2005 | Arazi, H; Taati, B; Sajedi, F Rafati; Suzuki, K | Not applicable based on title |
| The acute effects of twenty-four hours of sleep loss on the performance of nationalcaliber male collegiate weightlifters | 2005 | Blumert, PA; Crum, AJ; Ernsting, M; Volek, JS; ..., | Not applicable based on title |
| Acute stress enhances the sensitivity for facial emotions: a signal detection approach | 2019 | Domes, Gregor; Zimmer, Patrick | Not applicable based on title |
| Up in the Air: Evidence of Dehydration Risk and Long-Haul Flight on Athletic Performance | 2020 | Zubac, Damir; Buoite Stella, Alex; Morrison, Shawnda A | Not applicable based on title |
| Changes in salivary levels of creatine kinase, lactate dehydrogenase, and aspartate aminotransferase after playing rugby sevens: the influence of gender | 2005 | Fern·ndez, ¡ Gonz·lez; OrtÌ, JE de la Rubia; ..., | Not applicable based on title |
| Sport analytics platform for athletic readiness assessment | 2005 | Moatamed, B; Darabi, S; Gwak, M; ..., | Not applicable based on title |
| Intercollegiate cross country competition: effects of warm-up and racing on salivary levels of cortisol and testosterone | 2005 | Casto, KV; Elliott, C; Edwards, DA | Not applicable based on title |
| Standardized Measures for Managing and Reducing Behavioral Health and Performance Risks | 2005 | Williams, TJ | Not applicable based on title |
| Time Course of Performance Changes and Fatigue Markers During Training for the Ironman Triathlon | 2005 | Joiner, AJ | Not applicable based on title |
| The effect of the arm swing on the ground and in the water on the heart rate of boating athletes | 2005 | Churproong, S | Not applicable based on title |
| Oxidative stress, muscle and liver cell damage in professional soccer players during a 2-game week schedule | 2018 | Viana-Gomes, D.; Rosa, F.L.L.; Mello, R.; Paz, G.A.; Miranda, H.; Salerno, V.P. | Included |
| Monitoring Athletic Training Status Through Autonomic Heart Rate Regulation: A Systematic Review and Meta-Analysis | 2016 | Bellenger, Clint R; Fuller, Joel T; Thomson, Rebecca L; Davison, Kade; Robertson, Eileen Y; Buckley, Jonathan D | Not applicable based on title |
| Salivary Hormone Assays | 2022 | Becker, L; Rohleder, N; Schultheiss, O | Not applicable based on title |
| Effect of competition on stress salivary biomarkers in elite and amateur female adolescent inline skaters | 2019 | Dehghan, F.; Khodaei, F.; Afshar, L.; Shojaei, F.K.; Poorhakimi, E.; Soori, R.; Fatolahi, H.; Azarbayjani, M.A. | Not applicable based on title |
| High prevalence of dehydration and inadequate nutritional knowledge among university and club level athletes | 2017 | Magee, Pamela Jane; Gallagher, Alison M; McCormack, Jacqueline M | Not applicable based on title |
| Identifying readiness to train: When to push and when to pull | 2005 | Turner, AN; Bishop, C; Springham, M; ..., | Not applicable based on title |
| Association between Cold Face Test-induced vagal inhibition and cortisol response to acute stress | 2011 | La Marca, Roberto; Waldvogel, Patricia; Thˆrn, Hanna; Tripod, MÈlanie; Wirtz, Petra H; Pruessner, Jens C; Ehlert, Ulrike | Not applicable based on title |
| Skin temperature changes of under-20 soccer players after two consecutive matches | 2017 | de Andrade Fernandes, Alex; Pimenta, Eduardo MendonÁa; Moreira, Danilo Gomes; Sillero-Quintana, Manuel; Marins, Jo„o Carlos Bouzas; Morandi, Rodrigo Figueiredo; Kanope, Tane; Garcia, Emerson Silami | Not applicable based on title |
| Effects of a moderate evening alcohol dose II: performance | 2007 | Rupp, Tracy L; Acebo, Christine; Seifer, Ronald; Carskadon, Mary A | Not applicable based on title |
| Changes in markers of stress, recovery, training load and performance during a women's Division I field hockey season | 2005 | Conway, SP | Not applicable based on title |
| Morningness eveningness and intelligence among high-achieving US students: Night owls have higher GMAT scores than early morning types in a top-ranked MBA program | 2014 | Piffer, Davide; Ponzi, Davide; Sapienza, Paola; Zingales, Luigi; Maestripieri, Dario | Not applicable based on title |
| The serotonin transporter gene variants modulate acute stress-induced hippocampus and dorsomedial prefrontal cortex activity during memory retrieval | 2019 | Li, Shijia; Tang, Jun; Gao, Yan; Thiel, Christiane M; Wolf, Oliver T | Not applicable based on title |
| Tech for talent [Olympic Games - technology] | 2021 | Andrews, C. | Not applicable based on title |
| The night before night shift: Chronotype impacts total sleep and rapid eye movement sleep during a strategically delayed sleep | 2023 | Reiter, Andrew M; Roach, Gregory D; Sargent, Charli | Not applicable based on title |
| The Effect of Monophasic Oral Contraceptives on Muscle Strength and Markers of Recovery After Exercise-Induced Muscle Damage: A Systematic Review | 2022 | Glenner-Frandsen, Astrid; With, Cecilie; Gunnarsson, Thomas P; Hostrup, Morten | Not applicable based on title |
| Cytokine responses and math performance: The role of stereotype threat and anxiety reappraisals | 2005 | John-Henderson, NA; Rheinschmidt, ML; ..., | Not applicable based on title |
| Associations among dehydration, testosterone and stress hormones in terms of body weight loss before competition | 2005 | Yildirim, I | Not applicable based on title |
| Biochemical and endocrine responses to impact and collision during elite rugby league match play | 2005 | McLellan, CP; Lovell, DI; Gass, GC | Not applicable based on title |
| Effects of a 3-month endurance event on physical performance and body composition: the G2 trans-Greenland expedition | 2005 | Frykman, PN; Harman, EA; Opstad, PK; Hoyt, RW; ..., | Not applicable based on title |
| Monitoring for overreaching in rugby league players | 2007 | Coutts, Aaron J; Reaburn, Peter; Piva, Terrence J; Rowsell, Greg J | Not applicable based on title |
| Physiological arousal of military personnel prior to separate bouts of competition | 2005 | Record, S | None peer reviewed article |
| Cryotherapy, Hydrotherapy and other training recovery modalities: Effects on hormones' balance and performance | 2005 | Jemni, M; Bianco, A; Fazlani, T | Not applicable based on title |
| Competition Calendar and Strength-Aerobic Training Sessions Interaction: The Brainteaser of Coach to Manage Training Schedule in Elite Team Sport Athletes & | 2005 | Morin, S; Ahmaidi, S; LeprÍtre, PM | Not applicable based on title |
| Performance, sleep and circadian phase during a week of simulated night work | 2005 | Lamond, N; DORRIAN, J; ROACH, GD; ..., | Not applicable based on title |
| The Science of Training - Soccer | 2006 | Reilly, Thomas | Not applicable based on title |
| Effect of long haul travel on maximal sprint performance and diurnal variations in elite skeleton athletes | 2007 | Bullock, Nicola; Martin, David T; Ross, Angus; Rosemond, Doug; Marino, Frank E | Not applicable based on title |
| Cognitive benefit and cost of acute stress is differentially modulated by individual brain state | 2017 | Kohn, Nils; Hermans, Erno J; Fern·ndez, GuillÈn | Not applicable based on title |
| Periodisation: tailoring training based on the menstrual cycle may work in theory but can they be used in practice? | 2020 | Julian, Ross; Sargent, Debby | Not applicable based on title |
| Predicting hydration status using machine learning models from physiological and sweat biomarkers during endurance exercise: A single case study | 2022 | Wang, Shu; Lafaye, Celine; Saubade, Mathieu; Besson, Cyril; Margarit-Taule, Josep Maria; Gremeaux, Vincent; Liu, Shih-Chii | Not applicable based on title |
| HRP Increment 45/46 Overview | 2005 | Kinder, K; Lead, I; Miller, G; Lead, LMIO | Not applicable based on title |
| Non-Invasive Physiological Monitoring for Physical Exertion and Fatigue Assessment in Military Personnel: A Systematic Review | 2021 | Bustos, Denisse; Guedes, Joana C; Vaz, M·rio P; Pombo, Eduardo; Fernandes, Ricardo J; Costa, JosÈ Torres; Baptista, Jo„o Santos | review |
| Effects of short-term normobaric hypoxia on haematology, muscle phenotypes and physical performance in highly trained athletes | 2006 | Basset, Fabien A; Joanisse, Denis R; Boivin, FrÈdÈric; St-Onge, JosÈe; Billaut, FranÁois; DorÈ, Jean; Chouinard, Richard; Falgairette, Guy; Richard, Denis; Boulay, Marcel R | Not applicable based on title |
| Monitoring training loads in professional basketball players engaged in a periodized training program | 2017 | Aoki, Marcelo S; Ronda, Lorena T; Marcelino, Pablo R; Drago, Gustavo; Carling, Chris; Bradley, Paul S; Moreira, Alexandre | Not applicable based on title |
| Cold Water Immersion Enhanced Athletes' Wellness and 10-m Short Sprint Performance 24-h After a Simulated Mixed Martial Arts Combat | 2018 | Tabben, Montassar; Ihsan, Mohammed; Ghoul, Nihel; Coquart, Jeremy; Chaouachi, Anis; Chaabene, Helmi; Tourny, Claire; Chamari, Karim | Not applicable based on title |
| Physiological, cognitive, and expectancy effects of aromatherapy following acute stress | 2005 | Fonareva, I | Not applicable based on title |
| C-Reactive Protein Serum Levels as an Internal Load Indicator of Sprints in Competitive Football Matches | 2019 | Jatene, Pedro; Dos Santos, Gustavo Silva; Portella, Daniel Leite | Not applicable based on title |
| Resistance exercise on two consecutive days induces cortisol, CK, IgA responses in active young males | 2005 | Arazi, H; Eghbali, E; Suzuki, K; Mahdavi, M | Not applicable based on title |
| HRP Increment 41/42 Overview | 2005 | Schwanbeck, N; Miller, G; Thaxton, J; Kershner, C | Not applicable based on title |
| Physiological and Psychological Stress Markers in Concussed Athletes from Injury to Post-return to Play | 2005 | Senthinathan, A | Not applicable based on title |
| Effects of external counterpulsation therapy on acute recovery in elite Australian rugby league players | 2005 | Roberts, L; James, L; Caia, J; Wellington, B; ..., | Not applicable based on title |
| Salivary cortisol is highly correlated with training intensity in English Premier League players | 2005 | Dunbar, J; Rosen, B; Gimpel, M; ..., | Not applicable based on title |
| Physical response to a simulated period of soccer-specific fixture congestion | 2005 | Page, RM; Marrin, K; Brogden, CM; ..., | Not applicable based on title |
| Anti-stress effect of theanine on students during pharmacy practice: positive correlation among salivary ±-amylase activity, trait anxiety and subjective stress | 2013 | Unno, Keiko; Tanida, Naoki; Ishii, Naoto; Yamamoto, Hiroyuki; Iguchi, Kazuaki; Hoshino, Minoru; Takeda, Atsushi; Ozawa, Hayato; Ohkubo, Tsutomu; Juneja, Lekh Raj; Yamada, Hiroshi | Not applicable based on title |
| Hydration level monitoring using embedded piezoresistive microcantilever sensors | 2005 | Gunter, RL; Delinger, WD; Porter, TL; Stewart, R; ..., | Not applicable based on title |
| Acute and chronic over-exertion: do depressed immune responses provide useful markers? | 1998 | Shephard, R J; Shek, P N | Not applicable based on title |
| Around the world in 16 days: the effect of long-distance transmeridian travel on the sleep habits and behaviours of a professional Super Rugby team | 2021 | Smithies, Tim D; Eastwood, Peter R; Walsh, Jennifer; Murray, Kevin; Markwick, Will; Dunican, Ian C | Not applicable based on title |
| Timing light treatment for eastward and westward travel preparation | 2009 | Paul, Michel A; Miller, James C; Love, Ryan J; Lieberman, Harris; Blazeski, Sofi; Arendt, Josephine | Not applicable based on title |
| Hydration status of Air Force military basic trainees after implementation of the back-mounted hydration system | 2005 | Fogt, DL; Brosch, LC; Dacey, DC; Kalns, JE; ..., | Included |
| Psychophysiological effects of precompetition anxiety on basketball performance | 2005 | Alexander, D | Not applicable based on title |
| Personalized stress monitoring: a smartphone-enabled system for quantification of salivary cortisol | 2018 | Rey, Elizabeth; Jain, Aadhar; Abdullah, Saeed; Choudhury, Tanzeem; Erickson, David | Not applicable based on title |
| Salivary cortisol and immunoglobulin A responses to simulated and official Jiu-Jitsu matches | 2005 | Moreira, A; Franchini, E; Freitas, CG de; ..., | Not applicable based on title |
| The effects of hydration on cognitive performance during a simulated wildfire suppression shift in temperate and hot conditions | 2005 | Cvirn, MA; Dorrian, J; Smith, BP; Vincent, GE; Jay, SM; ..., | No salivary markers |
| Acceptance Date: June 14, 2018 | 2005 | Tavares, F; Beaven, M; Teles, J; Baker, D; ..., | Not applicable based on title |
| Physiological stress markers during breath-hold diving and SCUBA diving | 2019 | Marlinge, Marion; Coulange, Mathieu; Fitzpatrick, Richard C; Delacroix, Romain; Gabarre, Alexie; LainÈ, Nicolas; Cautela, Jennifer; Louge, Pierre; Boussuges, Alain; Rostain, Jean-Claude; Guieu, RÈgis; Joulia, Fabrice C | Not applicable based on title |
| Lowest Perceived Exertion In The Late Morning Due To Effects Of The Endogenous Circadian System: 798 Board# 59 May 30 2: 00 PM-3: 30 PM | 2005 | Thosar, SS; Herzig, MX; Roberts, SA; Berman, AM; ..., | Not applicable based on title |
| D4 3 Concept for Physiological Measurement Suite for Stress Assessment | 2020 | UHEI, DL; Giessing, L; Frenkel, MO | Not applicable based on title |
| The Effectiveness of Mindfulness-Based Cognitive-Behavioral Strategy Training on Cognitive Emotion Regulation Strategies and Salivary Cortisol Levels in & | 2005 | Samadi, H; Maleki, B; Sohbatiha, M | Not applicable based on title |
| Fatigue biomarker index: an objective salivary measure of fatigue level | 2012 | Michael, Darren J; Daugherty, Sheena; Santos, Adrienne; Ruby, Brent C; Kalns, John E | Included |
| S53 The Effects of a Prebiotic Supplementation on Reading and Cognitive Performance in Elementary School Children: A Randomised Placebo-Controlled Study | 2005 | Baiao, R; Capitao, L; Baek, H; ..., | Not applicable based on title |
| Longitudinal follow-up of biochemical markers of fatigue throughout a sporting season in young elite rugby players | 2005 | Alaphilippe, A; Mandigout, S; Ratel, S; ..., | Not applicable based on title |
| Repeated low-dose caffeine ingestion during a night of total sleep deprivation improves endurance performance and cognitive function in young recreational runners: A randomized, double-blind, placebo-controlled study | 2022 | Khcharem, Amir; Souissi, Wajdi; Masmoudi, Liwa; Sahnoun, Zouheir | Not applicable based on title |
| The Relation of Psychometric Scales, Cortisol Concentration in Athletic Performance in Team Sports: A Systematic Review | 2005 | Torres, VBC; Domingos-Gomes, JR; ..., | review |
| Oxidative stress and motion sickness in one crew during competitive offshore sailing | 2005 | Giacon, TA; Bosco, G; Vezzoli, A; Dellanoce, C; ..., | Not applicable based on title |
| Acute Psychosocial Stress Increases Cognitive-Effort Avoidance | 2021 | Bogdanov, Mario; Nitschke, Jonas P; LoParco, Sophia; Bartz, Jennifer A; Otto, A Ross | Not applicable based on title |
| The effects of spectral tuning of evening ambient light on melatonin suppression, alertness and sleep | 2017 | Rahman, Shadab A; St Hilaire, Melissa A; Lockley, Steven W | Not applicable based on title |
| Optimal Hydration Biomarkers: Consideration of Daily Activities | 2005 | McKenzie, CXMAL; Armstrong, LE | Included |
| Hydration assessment of athletes | 2005 | Cheuvront, SN; Sawka, MN | Not applicable based on title |
| An experimental study of adolescent sleep restriction during a simulated school week: changes in phase, sleep staging, performance and sleepiness | 2017 | Agostini, Alex; Carskadon, Mary A; Dorrian, Jillian; Coussens, Scott; Short, Michelle A | Not applicable based on title |
| Athlete burnout: What we know, what we could know, and how we can find out more | 2005 | Eklund, RC; DeFreese, JD | Not applicable based on title |
| Genetics and Genomics in Sports | 2005 | MIYAMOTO-MIKAMI, ERI; FUKU, N | Not applicable based on title |
| The Human Research Program Suite of Integrated One-Year Mission Experiments: Description and Integration | 2005 | Shelhamer, M; Oubre, C | Not applicable based on title |
| The science and practice of middle and long distance running | 2021 | Homer, Mark R.; Pedlar, Charles R. | Not applicable based on title |
| Hormonal and performance responses to a peak-taper cycle in collegiate throwers | 2005 | Kern, AJ | Not applicable based on title |
| Salivary Cortisol Analysis In Collegiate Female Lacrosse Athletes: 120 | 2005 | Figueroa, YL; Carter, JL; Mathews, SL; Bunn, JA | Not applicable based on title |
| Nocturnal sustained attention during sleep deprivation can be predicted by specific periods of subjective daytime alertness in normal young humans | 2006 | Taillard, Jacques; Moore, Nicholas; Claustrat, Bruno; Coste, Olivier; Bioulac, Bernard; Philip, Pierre | Not applicable based on title |
| Stress appraisals influence athletic performance and psychophysiological response during 161 km cycling time trials | 2005 | Thompson, MA; Toner, J; Perry, JL; Burke, R; ..., | Not applicable based on title |
| Physiological and Perceptual Recovery-Stress Responses to an Elite Netball Tournament | 2005 | Russell, S; Simpson, MJ; Evans, AG; ..., | Not applicable based on title |
| Salivary hormone response to maximal exercise at two time points during the day | 2005 | Hayes, LD; Grace, FM; LonKilgore, J; ..., | Not applicable based on title |
| Effects of prolonged running under different environmental conditions onselected physiological markers, muscle damage and time trial performance inrecreational athletes | 2005 | Suhaimi, MZB; Chen, CK; ..., | No salivary markers |
| The Integration of Salivary Immunoglobulin A by the Repetitive Stressful Task | 2005 | Nomura, S; Mizuno, T; Nozawa, A; ..., | Not applicable based on title |
| Effects of Eurycoma longifolia Jack supplementation on eccentric leg press exercise-induced muscle damage in rugby players | 2005 | Zakaria, A; Washif, J; Lim, B; Nosaka, K | Not applicable based on title |
| Proton density weighted laryngeal MRI in systemically dehydrated rats | 2005 | Oleson, S; Lu, KH; Liu, Z; Durkes, AC; ..., | Not applicable based on title |
| Acute Fatigue Responses to Occupational Training in Military Personnel: A Systematic Review and Meta-Analysis | 2023 | Heilbronn, Brian; Doma, Kenji; Sinclair, Wade; Connor, Jonathan; Irvine-Brown, Lachlan; Leicht, Anthony | Review |
| Planning for Sports Ultimate Performance | 2005 | Manilal, KP | Not applicable based on title |
| How positive affect buffers stress responses | 2005 | Steenbergen, H van; Bruijn, ERA de; ..., | Not applicable based on title |
| Biochemical Response Comparisons of a Competitive Microcycle Vs Congested Fixture Periods in Elite Level European Champions League Soccer Players | 2005 | Owen, AL; Djaoui, L; Dellal, A; Ates, O; ..., | Not applicable based on title |
| Live and/or sleep high:train low, using normobaric hypoxia | 2008 | Richalet, J-P; Gore, C J | Not applicable based on title |
| Decreased sympathetic cardiovascular influences and hormone-physiological changes in response to Covid-19-related adaptations under different learning environments | 2022 | Gellisch, Morris; Wolf, Oliver T; Minkley, Nina; Kirchner, Wolfgang H; Br¸ne, Martin; Brand-Saberi, Beate | Not applicable based on title |
| 0231 The Impact of At-Home Actigraphy on Performance and Sleepiness in the Lab over 62 Hours of Total Sleep Deprivation | 2005 | Carlsson, KE; Bessey, AF; Skeiky, L; Prindle, NE; ..., | Not applicable based on title |
| Investigation of the relationship between salivary | 2005 | Tiernan, C | Not applicable based on title |
| Comparing performance during morning vs afternoon training sessions in intercollegiate basketball players | 2017 | Heishman, Aaron D; Curtis, Michael A; Saliba, Ethan N; Hornett, Robert J; Malin, Steven K; Weltman, Arthur L | Not applicable based on title |
| Monitoring training and performance in athletes | 2005 | McGuigan, M | Not applicable based on title |
| Sex differences in the physical performance, physiological, and psycho-cognitive responses to military operational stress | 2022 | Conkright, William R; O'Leary, Thomas J; Wardle, Sophie L; Greeves, Julie P; Beckner, Meaghan E; Nindl, Bradley C | review |
| Cortisol, reaction time test and health among offshore shift workers | 2010 | Harris, Anette; Waage, Siri; Ursin, Holger; Hansen, Ase Marie; Bjorvatn, Bj¯rn; Eriksen, Hege R | Chronic/Prolonged stressor |
| The usefulness of performing biochemical tests in the saliva of kickboxing athletes in the dynamic of training | 2019 | Volodchenko, Oleksandr Anatolievich; Podrigalo, Leonid Vladimirovich; Iermakov, Sergii Sidorovich; {ychowska, MaBgorzata Teresa; JagieBBo, WBadysBaw | Not applicable based on title |
| Effects of exercise in hot and humid conditions and bovine colostrum on salivary immune markers | 2020 | McKenna, Zachary; Berkemeier, Quint; Gorini, Felipe; Kuennen, Matthew; Naylor, Ashley; Kleint, Austin; Gillum, Trevor | Not applicable based on title |
| The relationship between the cortisol awakening response, mood states, and performance | 2005 | DÌaz, MM; Bocanegra, OL; Teixeira, RR; ..., | Not applicable based on title |
| The effect of 40 h constant wakefulness on task-switching efficiency | 2009 | Bratzke, Daniel; Rolke, Bettina; Steinborn, Michael B; Ulrich, Rolf | Not applicable based on title |
| Melatonin and cortisol assessment of circadian shifts in astronauts before flight | 1995 | Whitson, P A; Putcha, L; Chen, Y M; Baker, E | Not applicable based on title |
| Salivary Testosterone and Cortisol Responses to Maximal Exercises in Tunisian Soccer Players | 2021 | Bouazizi, M | Not applicable based on title |
[truncated: 20,427 more chars]
