# Supplementary material for: Salivary biomarkers of tactical athlete readiness: A systematic review
Source: PLoS One. 2025 Apr 29;20(4):e0321223. doi: 10.1371/journal.pone.0321223 (PMC12040155; doi:10.1371/journal.pone.0321223)
Supplement: S3 Table — (DOCX) [file pone.0321223.s003.docx]

**S3 Table.** Characteristics and key findings of included studies

| **Author, Year,**  **Country,**  **Title** | **Participants** | **Study Design** | **Study Description** | **Salivary Biomarkers & Collection Method** | **Salivary Biomarker Quantification Method** | **Acute Stressors** | **Paired Measures of Stress/Performance** | **Quantified Change in Biomarker(s)** | **Key Findings** |
| --- | --- | --- | --- | --- | --- | --- | --- | --- | --- |
| Cook et al.  (2011)  Country: UK  “Skill Execution and Sleep Deprivation: Effects of Acute Caffeine or Creatine Supplementation - a Randomized Placebo-controlled Trial” | Profile: Elite rugby players  n= 10  Age (y): 20 ± 0.5  Sex: 10 M | Randomized (crossover) | Players completed 10 trials of a simple rugby passing skill test. For 5 of these trials, participants had between 7-9 hours of sleep, while in the other 5 trials they had 3-5 hours of sleep. | Cortisol, testosterone  Passive drool | Samples were analyzed in duplicate using commercial kits (Salimetrics, PA, USA). Minimum detection limit for testosterone was 2 pg/ml with intra- and interassay coefficients of variation (CV) of 1.2-12.7%. Minimum detection limit for cortisol was 0.3 ng/ml with intra- and inter-assay CV of 2.6-9.8%. | Sleep deprivation (3-5 hours of sleep) | Rugby ball passing skill with dominant and nondominant hand | Cortisol: ↑ 33%  Testosterone ↔  *Graph estimate | Significant differences in rugby passing skill were found between sleep deprived and non-sleep deprived conditions for the placebo (non-supplemented) group. However, cortisol was only slightly elevated in the sleep deprived group compared to the non-sleep deprived, with the difference not reaching statistical significance. Testosterone levels were not affected by sleep status in the placebo group. |
| Chennaoui et al.  (2009)  Country: France  “Effects of Ramadan Fasting on Physical Performance and Metabolic, Hormonal, and Inflammatory Parameters in Middle-Distance Runners” | Profile: Middle distance athletes  n= 8  Age (y): 25 ± 1.3  Sex: Not reported | Cohort | Middle distance athletes performed a max aerobic velocity (MAV) 5 days before and on days 7 and 21 of Ramadan fasting (RF). Additionally, saliva samples were taken before and after MAV tests. | Cortisol, testosterone, T:C  Not reported | Salivary cortisol concentrations were assayed in duplicate by radioimmunoassay using commercial kits (DiaSorin, Anthony, France) with a limit of sensitivity of 0.28 nmol.L^-1^. Salivary testosterone cocentrations were assayed in duplicate by enzyme-linked immunoassay (ELISA), using Salimetrics Salivary Testosterone Enzyme Immunoassay Kit, with a sensitivity of 0.05 ng·mL^-1^. CV not listed. | Partial sleep deprivation, decrease in caloric intake | Maximal aerobic velocity (MAV) | MAV pre/post:  Cortisol: ↑ 65% (day 7)  Testosterone: ↑ 91% (average 3 days)  T/C: ↑ 105 % (day 5)  Pre/post RF:  Cortisol: ↑ 33%  Testosterone: ↓ 5% | MAV performance decreased at day 7 and 21 of fasting relative to baseline. Within test days, testosterone significantly increased from pre- to post MAV. Cortisol and T:C only significantly increased post-MAV on day 7 and baseline, respectively. No differences in hormonal concentrations were observed across the fasting period (between baseline, day 7 and day 21). Fasting and partial sleep-deprivation may affect hormonal response to physical fatigue as evidenced by greater increase in cortisol response mid-fasting relative to baseline. |
| Donald et al.  (2017)  Country: Ireland  “Acute Effects of 24-h Sleep Deprivation on Salivary Cortisol and Testosterone Concentrations and Testosterone to Cortisol Ratio Following Supplementation With Caffeine or Placebo” | Profile: Amateur competitive athletes who actively participate in a competitive sport on a seasonal basis  n= 11  Age (y): 24 ± 6  Sex: 11 M | Randomized (crossover) | Athletes participated in 3 test days, 1 without sleep deprivation, 1 with sleep deprivation, and 1 with sleep deprivation with caffeine supplementation. Testing included a modified Hoff test and Yo-Yo test. Each test day was separated by at least 1 week. | Cortisol, testosterone, T:C  Passive Drool | Samples were assayed for cortisol and testosterone concentrations using ELISA kits (Salimetrics, PA, USA). Each test used 25 µL for singlet determination. Cortisol assay had a lower limit sensitivity of 0.007 µg.dL^-1^ and mean intra- and inter-CV of 6.8 and 15%, respectively. The testosterone assay had a lower limit sensitivity of 3.7 pg.mL^-1^ and mean intra- and inter-CV of 6.5 and less than 15% respectively. | 24-hour sleep deprivation | 2 aerobic tests: a modified Hoff and Yo-Yo | Cortisol: ↑ 100% (Placebo after Yo-Yo test)  Testosterone: ↔  T/C: ↓ 30% (Placebo after Yo-Yo) | No significant differences were found for aerobic test times across states. However, cortisol levels were higher for the sleep deprived control arm (placebo) compared to the non-sleep deprived and sleep-deprived with caffeine supplementation both pre and post modified Hoff test and post Yo-Yo. Testosterone and T:C were lower in the sleep-deprivation group compared to non- sleep deprivation group before and after modified Hoff test and Yo-Yo. |
| Lieberman et al.  (2005)  Country: US  “Severe Decrements in Cognition Function and Mood Induced by Sleep Loss, Heat, Dehydration, and Undernutrition During Simulated Combat” | Profile: US Army elite light infantry unit  n= 31  Age (y): 31.6 ± 0.4  Sex: 31 M | Cohort | Cognitive and physiological markers of stress were evaluated before, during, and after an intense training exercise conducted for 53 hours in the heat. | Cortisol, testosterone, melatonin | Cortisol, testosterone, and melatonin were determined with standard radioimmunoassay procedures. Cortisol was assessed with a kit from Incstar Corporation (sensitivity and CV not provided). Testosterone was assessed with an assay from Diagnostic Products (sensitivity and CV not provided). Melatonin was assessed via ELISA kit from Alpco (sensitivity and CV not provided). | Intense exercise with load carriage during a 53-hour period that included sleep deprivation, dehydration, and extreme ambient temperature (peak 31°C) | Body weight, cognitive performance, and mood assessment | Cortisol: ↓ 18%, ↑ 76% (AM, PM) Testosterone: ↓ 44%, ↓ 23% (AM, PM) Melatonin ↔ | Post-training, cognitive measures, mood state, and body weight and hydrations status had all significantly declined. Additionally, cortisol increased significantly from before to after training while testosterone decreased. Stressors inherent in military training like severe sleep loss disrupts the normal diurnal rhythm of cortisol release, resulting in elevated cortisol levels throughout the day as well as suppressed testosterone. |
| Machi et al.  (2012)  Country: US  “The Relationship Between Shift Work, Sleep, and Cognition in Career Emergency Physicians” | Profile: Emergency physicians  n= 13  Age (y): 38.2 ± 7.0 (31–52)  Sex: 9 M: 4 F | Cohort | Emergency department physicians were tested before and after day/night shifts using several cognitive tests. In addition, saliva was collected before and after shifts. | Cortisol, melatonin  Not reported | Cortisol levels were assayed by enzyme immunoassay (Salimetrics, PA, USA). Melatonin levels were assayed by ELISA (Direct Saliva Melatonin ELISA Kit, Buhlman, Switzerland). Sensitivity and CV not listed. | Sleep deprivation due to overnight shifts | Cognitive tests including the Paced Auditory Serial Addition Test, the University of Southern California Repeatable Episodic Memory Test, the Trail Making Test, and the Stroop Color-Word Test | Cortisol: ↓28% morning peak (night shift compared to day shift)  Melatonin: ↔  *Graph estimate | Physicians reported lower sleep-quality scores than the general public and cognitive measures were significantly decreased after shifts. Cortisol remained elevated after the night shift, where normal diurnal rhythm would result in lower cortisol release in the morning. Additionally, evening cortisol levels showed a trend to be higher in night-shift workers compared to day shift. No effect of shift or time was found for melatonin. |
| Mantua et al.  (2020)  Country: US  “Sleep Loss During Military Training Reduces Testosterone in the US. Army Rangers: A Two-Study Series” | Profile: US army rangers from paratrooper and ground assault preparations  n= Study 1 n = 76; Study 2 n = 44  Age (y): Study 1 25.95 ± 4.00  Study 2 24.00 ± 3.14  Sex: 120 M | Cohort | Saliva samples were collected during before and after routine Army Ranger training missions | Testosterone  Passive drool | Testosterone was quantified in duplicate using Salimetrics Salivary Testosterone ELISA kit. Sensitivity and CV not listed. | Acute sleep deprivation - single night nocturnal training | None | Testosterone: ↓ 28.0%, ↓ 25.4% (study 1, study 2) | Testosterone was 28% and 25.4% lower, respectively, after two training missions (90% lower than comparative historical data for similar populations). These data support prior findings that sleep deprivation can reduce testosterone levels. |
| Michael et al.  (2013)  Country: US  “Salivary Biomarkers of Physical Fatigue as Markers of Sleep Deprivation” | Profile: college students, ROTC cadet trainees, and recreational (non-varsity) athletes  n= 30  Age (y): 18-35  Sex: 30 M | Randomized (control) | Participants were randomized into control and sleep-deprived groups. Both groups were monitored over 48 hours with measures taken every 3 hours. The sleep-deprived group were not permitted to sleep over the test period. | Fatigue Biomarker Index (FBI) - ratio of two salivary peptides that correlate with fatigue.  Passive drool | Protein content was quantified using a bicinchoninic acid (BCA) assay, and peptides were labeled with light and heavy isotopes of acetic anhydride to tag free amines, enabling identification and quantification through liquid chromatography-mass spectrometry. | 48-hour sleep deprivation | Profile of Mood States (POMS) and Stroop Color-Conflict Test | FBI: ↓ x10000 (log10: 3 to -1) peak sleep deprived compared to control following the first night | Significant differences were found for Stroop performance and FBI after the first overnight period relative to baseline with worse Stroop performance and higher FBI levels throughout the day after sleep-deprivation. Receiver-operator characteristic (ROC) analysis suggested that salivary biomarkers of physical fatigue can identify sleep-deprived patients with reasonable specificity and selectivity (area under curve, 92%). |
| Pajcin et al.  (2017)  Country: AU  “Decreased Salivary Alpha-amylase Levels are Associated with Performance Deﬁcits During Sleep Loss” | Profile: Young adults  n= 12  Age (y): 22.4 ± 2.5  Sex: 7 M: 5 F | Quasi-experimental | Participants were sleep deprived for 50 hours and had performance measures and saliva samples taken 8 times per day over 2 days. | Salivary alpha-amylase (sAA)  Stimulated | Saliva samples were assayed using a sAA specific kinetic enxyme assay kit (Salimetrics, PA, USA) with a sensitivity of 0.4 U/ml. CV not listed. | 50-hour sleep deprivation | Performance on driving simulation and Psychomotor vigilance test (PVT) | alpha- amylase: ↓ x300 (log10 4.5 to 2)* | PVT and simulated driving performance was significantly worse after sleep deprivation compared to rested. AA values demonstrated a marked diurnal profile similar to performance and were associated with performance, with higher AA levels associated with better performance and vice versa. |
| Serpell et al.  (2019)  Country: UK  “Sleep and Salivary Testosterone and Cortisol During a Short Preseason Camp: A Study in Professional Rugby Union” | Profile: Professional rugby players  n= 19  Age (y): 26.4 y ± 3.9  Sex: 19 M | Cohort | Rugby players were monitored for 8 days around a 4-day training camp. Sleep-onset, latency, sleep duration, efficiency, and waking time were measured via wrist actigraphy. Saliva samples were taken on waking and 45 minutes after waking. | Cortisol, testosterone, T:C  Passive drool | Cortisol and testosterone were assayed using commercial ELISA kits (Salimetrics, PA, USA). Testosterone sensitivity was 3.67 pmol/L with intra-assay and inter-assay CV of <5.4% and <6.0%, respectively. Cortisol assay sensitivity was 3.00 nmol/L with intra-assay and inter-assay CV of <4.8% and <5.2%, respectively. | Sleep quality | Self-perceived muscle soreness | Cortisol: ↑ 100%, day 1-2 waking. ↓ 33% day 3 to day 4  Testosterone: ↔  T/C ratio: ↓ 33% day 1 to day 2  *Graph estimate | Significant differences were found for sleep efficiency and waking time across camp days, with a small increase in efficiency between day 2 and 3 and earlier wake times on days 1 to 3 compared to baseline. On waking, cortisol significantly increased across the camp from day 1, while T:C significantly decreased across camp days from day 1. With little change in sleep quality, and therefore, a lack of expected sleep deprivation, it is likely that the rise in cortisol with and between days seen in this study was in part related to anticipation of the day ahead. A weak inverse correlation was observed between either C or T:C upon waking and self-perceived muscle soreness |
| Summers et al.  (2021)  Country: AU  “The Acute Readiness Monitoring Scale: Assessing Predictive and Concurrent Validation” | Profile: Young adults  n= 30  Age (y): 23 ± 4  Sex: 12 M: 18 F | Quasi-experimental | After a 5-day baseline assessment, participants completed a 24-hour sleep-deprivation protocol. | Cortisol  Passive drool | Cortisol was quantified in duplicateusing commercial ELISA kits (Salimetrics, PA, USA). Sensitivity was not listed but intra- and inter-assay CV was 3.44% and 6.24%, respectively. | 24-hour sleep deprivation | Acute Readiness Monitoring Scale (ARMS), Psychomotor vigilance test (PVT), heart rate variability (HRV), National Aeronautics and Space Administration Task Load Index (NASA-TLX) | Cortisol awakening resposnse: ↓ ~40%  *Graph estimate | Participants reported increased sleepiness throughout the night of the sleep deprivation protocol as well as significant reductions in reaction time and readiness and reported increased workload scores. Additionally, lower awakening response in cortisol was observed post sleep-deprivation with lower scores on the ARMS readiness subscales being significantly associated with lower cortisol awakening responses. |
| Taylor et al.  (2017)  Country: US  “Anabolic Hormone Profiles in Elite Military Men: Robust Associations with Age, Stress, and Fatigue” | Profile: Active-duty military members of the elite Navy Sea, Air, and Land (SEAL) community  n= 57  Age (y): 33.4 ±7.3  Sex: 57 M | Cohort | Salivary anabolic hormone profiles (5 samples per day for 2 days) of active-duty soldiers were measured during the following conditions: blood pressure, sleep, perceived stress, fatigue. | DHEA, testosterone  Unstimulated | DHEA and testosterone were assayed in duplicate using commercially available immunoassays (Salimetrics, PA, USA). Sensitivity for DHEA was 5 pg/mL and 1 pg/mL for testosterone. Intra- and inter-assay CV for both assays were <10% and <15%, respectively. | Military duty | Sleep efficiency, subjective fatigue score, and perceived stress | Fatigue:  DHEAS: ↓ (quantity not provided)  Testosterone: ↓ 30 % (quartiles 1 and 4 difference)  Perceived stress:  Testosterone: ↓ (quantity not provided) | Testosterone and DHEAS were not significantly associated with sleep efficiency, however, lower levels of both hormones were associated with greater reported fatigue while testosterone was negatively associated with perceived stress. |
| Xu et al.  (2018)  Country: China  “Discovery and Identification of Fatigue-related Biomarkers in Human Saliva” | Profile: Emergency Physicians  n= 47  Age (y): 27-41  Sex: 36 M: 12 F | Cohort | Saliva samples were collected before and after >18 hour shifts from emergency room physicians split into 2 groups (fatigued and non-fatigued) based on collected electroencephalogram (EEG) data. | Identified salivary proteins  Stimulated | Proteins from saliva samples in this study were quantified using the BCA Protein Assay Kit, following acetone precipitation to isolate proteins. The concentration of the dissolved proteins was then measured, and a fixed amount of protein (100 µg) was used for downstream processing, including labeling with tandem mass tags for quantitative proteomics analysis. | Fatigue via emergency room shift work | Presence of fatigue waves in EEG signal and Piper Fatigue Scale (PFS) score | 30 proteins were identified to differentiate (as a group) fatigue from none-fatigue, each protein had different content changes individually | 12 of the 47 physicians were defined as fatigued using EEG theta waves. 767 total proteins were identified from the sample. 34 proteins were significantly associated with fatigue. Using diagnostic tests, 8 proteins were identified that high diagnostic value were used to establish discriminant equations of fatigue with an overall correct diagnostic rate of 95.7%. Fatigue proteins identified included immune, inflammatory factor, metabolic and tumor related. |
| Bellar et al.  (2017)  Country: US  “The Effects of a 36-hour Mixed Task Ultra-Endurance Race on Mucosal Immunity Markers and Pulmonary Function” | Profile: Ultra-endurance runners  n= 13  Age (y): 34.5 ± 5.2  Sex: 12 M: 12 F | Cohort | Salivary markers as well as pulmonary function were assessed for participants of a mixed task ultra-endurance event. Saliva samples were taken before the race and after completion or dropout. | Salivary flow rate, immunoglobin-A (sIgA), and sAA  Passive drool | sAA and sIgA was quantified via a kinetic enzyme assay and indirect competitive immunoassay, respectively (Salimetrics, PA, USA). Samples were analyzed in duplicate and average CV was <5%. Sensitivity not listed. | Physiological fatigue and sleep-deprivation due to ultra-endurance event comprising of 36 hours of aerobic and strength events | Pulmonary function test (peak flow rate) and event completion or dropout. | IgA: ↓33% (finishers), ↑ 45% (non-finishers) IgA type 1: ↓29% (finishers), ↔ (non-finishers)  Flow rate: ↓20% (finishers), ↑ 50% (non-finishers) Alpha-amylase: ↔  *Graph estimate | Event finishers showed a trend towards increased peak flow rate after the event. Salivary flow rate also trended towards being reduced in finishers but was significantly increased in non-finishers post event. Expression of salivary IgA was significantly decreased in finishers but increased in non-finishers. No change was found in AA. |
| Bonato et al.  (2020)  Country: Italy  “Effect of High-Intensity Interval Training Versus Small-sided Games Training on Sleep and Salivary Cortisol Level” | Profile: None professional soccer players  n= 30  Age (y): HIIT: 23 (6), SSG: 25 (7)  Sex: 30 M | Randomized (parallel group) | Salivary cortisol was collected before and after the activities of participants randomized into either high-intensity interval training (HIIT) or small-sided games (SSGs) groups. Additionally, sleep parameters were measured in all participants 2 nights before and after the activity. | Cortisol  Not reported | A salivary cortisol kit (DKO020; DiaMetra, Italy) based on a competitive immunoenzymatically colorimetric method was used for quantitative determination of cortisol. Intra- and inter-assay CV were both <14%. Sensitivity was not listed. | Fatigue and muscle damage induced by HIIT or SSGs activities | Sleep parameters and exercise intensity as measured by heart rate (HR) | Cortisol: ↑ 300%, ↑ 50% (HIIT, SSG)  *Graph estimate | The intensity of exercise for all HIIT bouts and SSGs were >90% predicted HR max. A greater reduction in sleep quality was found after HIIT compared to SSGs, while salivary cortisol significantly increased after both training sessions with a greater increase post HIIT compared to SSG. Additionally, the cortisol awakening response was significantly increased post HIIT session. |
| Chen et al.  (2017)  Country: US  “Salivary Mitochondrial DNA Copy Number is Associated with Exercise Ventilatory Efficiency” | Profile: Young adults  n= 15  Age (y): 32.2 ± 7.1  Sex: 14 M: 1 F | Quasi-experimental | All participants performed a cardio-pulmonary exercise challenge for 8 minutes with 4-6 minutes at 80-90% max heart rate (HR) during which gas exchange was measured. Saliva was collected prior to the test. | Salivary Mitochondrial DNA Copy Number (mtDNAcn)  Not reported | Mitochondrial DNA (mtDNA) from saliva samples was quantified using real-time polymerase chain reaction (RT-PCR). DNA was extracted from saliva collected in DNA collection kits, purified, and digested before being quantified via UV spectrophotometry. The mtDNA copy number was then calculated using plasmid calibration standards and analyzed using RT-PCR with specific primers targeting a mitochondrial gene region. | Physiological fatigue via cardiopulmonary exercise protocol. | Ventilatory efficiency (VE/V̇co2) and oxygen consumption (V̇o2·kg^−1^). | N/A: No post exercise sample collected. | Greater mtDNAcn was associated with lower VE/V̇co2 but not V̇o2·kg−1 after adjustment for physical activity. These results suggest that greater mitochondrial content is associated with more efficient ventilation, i.e., individuals with greater mitochondrial content have a greater capacity for oxidative phosphorylation, and therefore, reduced reliance on anaerobic metabolism. |
| Crewther et al.  (2013)  Country: UK  “The Workout Responses of Salivary-free Testosterone and Cortisol Concentrations and Their Association with the Subsequent Competition Outcomes in Professional Rugby League” | Profile: Professional rugby players  n= 13  Age (y): 23.3 ± 3.5  Sex: 13 M | Cohort | Participants were followed over a 7-week period at the start of the competitive season. Saliva was measured before and after mid-week resistance training exercise. | Cortisol, testosterone  Passive drool | Samples were analyzed in duplicate using commercial kits (Salimetrics, PA, USA). Minimum detection limit for testosterone was 6.1 pg·ml^-1^ with inter-assay CV <12%. Minimum detection limit for cortisol was 0.12 ng·ml^-1^ with an inter-assay CV <7%. | Resistance load workouts (part of training program) | Competitive record as well as outcome metrics including points ranking, points differential ranking and coach ranking | Cortisol: ↓ 23% / 26% pre to post workout (wins/losses)  Testosterone: ↑ 31% / 3% pre to post workout (wins/losses) | Elevated testosterone levels after mid-week workout were associated with game wins, but no significant difference before and after mid-week training was observed before losses. Free testosterone was also moderately related to coach ranking, points differential ranking and points ranking. Cortisol decrease was found pre and post mid-week training before both wins and losses with no significant difference between the two outcomes. |
| Crewther et al.  (2016)  Country: UK  “Salivary Testosterone Responses to a Physical and Psychological Stimulus and Subsequent Effects on Physical Performance in Healthy Adults” | Profile: Healthy university students  n= 26  age (y): men = 24.5 ± 5.3, women = 23.5 ± 3.9)  Sex: 12 M: 14 F | Randomize (crossover) | Participants completed 3 sessions in a randomized, crossover design: exercise with cycling sprints, viewing an aggressive video, and a control session. Salivary testosterone, hand-grip strength (HGS) and countermovement jump (CMJ) power were measured pre and post each session. | Testosterone  Passive drool | Samples were analyzed in duplicate using commercial kits (Salimetrics, PA, USA). Minimum detection limit was 6.1 pg·ml^-1^ with inter-assay CV <12%. | Sprint cycling | Physical performance testing which included HGS and CMJ peak power | Testosterone: ↑ 20%, ↑ 15% (men, women) | Testosterone was not significantly increased pre to post session for any intervention in men but was after sprint cycling and watching an aggressive video in women, with the sprint cycling resulting in a greater increase in testosterone than the video. Although not significant, a brief bout of sprint cycling elevated testosterone in men as well. |
| Crewther et al.  (2018)  Country: UK  “Can Salivary Testosterone and Cortisol Reactivity to a Mid-week Stress Test Discriminate a Match Outcome During International Rugby Union Competition?” | Profile: Professional rugby players  n= 33  Age (y): 27.9 ± 3.2  Sex: 33 M | Cohort | Participants completed a standardized stress test 3-4 days before 7 international rugby matches. On test days, saliva samples were measured in the morning, pre-test, and post-test. | Cortisol, testosterone  Passive drool | Samples were analyzed in duplicate using commercial kits (Salimetrics, PA, USA). Testosterone assay had a minimum detection limit of 6.1 pg·ml^-1^ while cortisol was 0.12 ng·ml^-1^. Inter-assay CV was between 5.2% and 10.1%. | Shuttle run test performance | Match outcome and coach rated player performance | Cortisol: ↑ 44% morning-pretest (wins)  ↓ ~53% (losses)  Testosterone: ↓ ~40% (wins and losses) | Morning testosterone and cortisol concentrations were lower before wins than losses and the morning to pre-test cortisol increase was significantly larger before wins but lower before losses. Regression analyses identified change from morning to pre-test testosterone and cortisol and change between pre and post-test testosterone as significant measures of match outcome with the final model having a classification accuracy of 72.2%. |
| Erskine et al.  (2007)  Country: UK  “Neuromuscular and Hormonal Responses to a Single Session of Whole Body Vibration Exercise in Healthy Young Men” | Profile: healthy, young adult males  n= 7  Age (y): 22.3 ± 2.7  Sex: 7 M | Randomized (crossover) | Participants performed 10 sets of half-squat isometric exercises for 1-min. Two trials were conducted either with whole body vibration (WBV) or without (control). Saliva samples were collected before, immediately after, 1, 2, and 24 hours after treatment. | Testosterone, cortisol, T:C  Unstimulated | Cortisol and testosterone were quantified were analyzed in duplicate using commercial enzyme immunoassay kits (Salimetrics, PA, USA). Average intra-assay CV for testosterone was 3.3% and 6.7% for high and low levels of concentration (pg·ml^-1^) while average inter-assay CV was 5.1% and 9.6%. Average intra-assay CV for cortisol was 3.88% and 7.12% for high and low levels concentration (μg 100 ml^-1^) while average inter-assay CV was 6.69% and 6.88%. | Isometric squat holds with and without WBV | Maximum isometric unilateral leg extension torque and rate of torque development | Testosterone: ↓ 10%, ↔ (WBV, Control)  Cortisol: ↑ 15%, ↓ 15% (WBV, control)  T:C: ↓ 22%, ↑ 40% (WBV, control)  *Estimates from graph for pre/post change | No significant effect for time was found in the control group for maximum extension torque or rate of torque development. Additionally, no significant differences were found for cortisol or testosterone across time. However, a trend was found for increased cortisol after WBV treatment. The imposed stimulus of isometric squat holds may not have been sufficient to trigger a hormonal response. |
| Gaviglio and Cook  (2014)  Country: UK  “Relationship Between Midweek Training Measures of Testosterone and Cortisol Concentrations and Game Outcome in Professional Rugby Union Matches” | Profile: Professional rugby players  n= 22  Age (y): 27.8 ± 4.0  Sex: 22 M | Cohort | Participants were assessed for salivary testosterone and cortisol concentrations before and after midweek training sessions over 6 weeks. | Cortisol, testosterone, T:C  Passive drool | Testosterone and cortisol were quantified in duplicate via commercial ELISA kits (Salimetrics, PA, USA). CV for both testosterone and cortisol were <10%. Sensitivity not listed. | Mid-week skill-based rugby training sessions | Match outcome | Cortisol: ↔  Testosterone: ↑ 30% / 20% pre to post skill test (wins/losses)  T/C ratio: ↑ 40% / 7% pre to post skill test(wins/losses)  *Graph estimate | A significant increase in testosterone after mid-week skill training sessions was seen before wins, however, cortisol did not show significant changes before wins. Additionally, a significant increase in T:C pre- to post training sessions was found before wins while the T:C was lower before wins than compared with before losses. |
| Gomez-Merino et al.  (2003)  Country: France  “Immune and Hormonal Changes Following Intense Military Training” | Profile: French military cadet school  n= 26  Age (y): 21 ± 2  Sex: 26 M | Cohort | Military cadets were followed over a 4-week training program. Saliva samples were taken before and after the duration of the training protocol. | Salivary immunoglobin A (sIgA), interleukin 6 (IL-6), prolactin, DHEA, testosterone, adrenocorticotrophic hormone (ACTH), cortisol  Passive drool | sIgA concentrations were determined by a modified nephelometric method (IGALC, Immunochemstriy IMMAGE system, Beckman Coulter, Roissy, France) with limit of sensitivity of 18 mg·L^-1^. Salivary protein concentrations were determined using a colorimetric method for clinical use in CSF and urine (urinary/CSF protein, AU600 Application, Olympus System Reagent, Rungis, France) with a maximal linearity of 2,000 mg·L^-1^. | 3-week endurance training followed by 1 week of combat training | None | IgA: ↓25%  Mean protein concentration: ↓15% | sIgA, testosterone, and DHEA were all significantly reduced pre- to post training. Additionally, sIgA was significantly increased post-training suggesting a suppression of the mucosal immune system associated with a release of proinflammatory cytokines. |
| González-Hernández et al.  (2022)  Country: Spain  “Response of Muscle Damage Markers to an Accentuated Eccentric Training Protocol: Do Serum and Saliva Measurements Agree? | Profile: resistance-trained sport science students  n= 16  Age (y): 26.6 ± 4.8 (M)  22.7 ± 1.4 (F)  Sex: 10 M: 6 F | Quasi-experimental | Participants performed accentuated eccentric loading (AEL) protocol of 8 sets of 10 reps squats against 120% estimated 1-repetition maximum. Salivary and serum muscle damage markers were measured before and 24 and 96 hours post training. | Creatine kinase (CK), aspartate aminotransferase (AST), and lactate dehydrogenase (LDH)  Passive drool | CK, AST, and LDH were measured with commercial kits adapted for saliva samples and performed in an automated biochemistry analyzer (Olympus A400, Beckman Coulter, Roissy, France). Details on specific assays used not listed. | AEL squat training protocol. | Serum muscle damage markers. | CK: ↑ 100% (post 96 hours)  AST: ↑ 18% (post 96 hours)  LDH: ↑ 9% (post 96 hours) | In serum, CK and LDH were significantly elevated 24 and 96 hours post training. However, in saliva significant increases in CK and LDH were only found 96 hours post, while the values measured in saliva were lower compared to those measured in serum. Bland-Altman plots showed low levels of agreement between serum and saliva measurements of biomarkers. Markers of muscle damage all increased from pre- to post training but lack of agreement between serum and saliva samples suggests that markers from different cannot be assumed to be found in similar concentrations. |
| Magiera et al.  (2018)  Country: Poland  “The Effect of Physical and Mental Stress on the Heart Rate, Cortisol and Lactate Concentrations in Rock Climbers” | Profile: Rock climbers with 1-6 years’ experience  n= 12  Age (y): 23 ± 4.6  Sex: 7 M: 5 F | Quasi-experimental | Climbers performed a variety of climbing routes testing various demands (physical, mental, fatigue). Saliva samples were taken in the morning, before warm-up, before climbing, immediately after descent, and 15 minutes after descent. | Cortisol, and blood lactate  Not reported | Cortisol was determined commercial ELISA kit (SLV-2930, DRG Instrments GmbH, Germany). Sensitivity and CV not listed. | Rock climbing with various difficulty levels. | Heart rate (HR) and blood lactate | Cortisol: ↑ 8%, ↑ 30% (easy route, difficult route)  *levels were within normal range at all times | HR was elevated from baseline during all climbing conditions but was most elevated during ‘difficult routes’. Blood lactate increased during the difficult route with rest, while cortisol increased pre- to post the easy climb, the repeated difficult climbs, and the difficult route with lead climb. Climbing tasks increased cortisol levels, even during ‘easy’ climbs suggesting that anticipation of a stressful event may also increase cortisol. |
| Magiera et al.  (2019)  Country: Poland  “Changes in Performance and Morning‐Measured Responses in Sport Rock Climbers” | Profile: Rock climbers (intermediate performance level, 1-3 years’ experience)  n= 6  Age (y): 26 ± 2.58  Sex: 6 M | Cohort | Participants took part in a 2-week training camp split into two phases that included 8 days of climbing routes and 4 rest days with difficulty level increased week to week (phases). Saliva samples were collected each morning. | Cortisol, testosterone, T:C  Not reported | Cortisol was determined commercial ELISA kit (SLV-2930, DRG Instrments GmbH, Germany). Sensitivity and CV not listed. | Rock climbing camp | Maximal voluntary contraction (MVC) hand grip strength, Self-perceived fatigue, HRV, and HR | Cortisol: ↑ (day 14)  Testosterone:↔  T/C: ↓ 42% (day 14)  *Graph estimate | Supine HR and HRV were significantly greater in phase 1 of the camp compared to pre-camp, with no differences in measures between phase 1 and 2. MVC significantly decreased from phase 1 to phase 2. Cortisol and testosterone were significantly decreased in both phases compared to pre-phase but tended to increase from phase 1 to 2. T/C was not significantly different across phases. Findings suggest that previous day’s workload elicits changes in hormonal biomarkers. |
| McLean et al.  (2010)  Country: AU  “Neuromuscular, Endocrine, and Perceptual Fatigue Responses During Different Length Between-Match Microcycles in Professional Rugby League Players” | Profile: Professional rugby players  n= 12  Age (y): 24.3 ± 3.6  Sex: 12 M | Cohort | Participants were followed during a 26-week season split into microcycles. Salivary markers as well as performance measures were taken pre-match and at different days after match to assess recovery. | Cortisol, testosterone, T:C  Passive drool | Cortisol and testosterone were determined in duplicate by ELISA assay (Salimetrics, PA, USA). CV as a percent of the assays were 7.3% and 2.4% for testosterone and cortisol, respectively. Sensitivity not listed. | Rugby gameplay and training | Countermovement jump (CMJ) performance (flight time and relative power), self-reported fatigue, sleepiness, muscle soreness, and mood | Cortisol: ↔  Testosterone: ↔ | CMJ performance was reduced post-match in all microcycles. Similar to CMJ, players’ fatigue, muscle soreness, and overall well-being was lower following each match. However, testosterone and T:C showed no significant changes within or between each microcycle along with high variation in individual response. There were also large variations in cortisol response, but a small post-match reduction was found 24-48 hours post-match followed by an increase up to 4 days later. |
| Merrigan et al.  (2021)  Country: US  “Acute Inflammatory, Cortisol, and Soreness Responses to Supramaximal Accentuated Eccentric Loading” | Profile: Resistance trained men  n= 21  Age (y): 24.0 ± 4.2  Sex: 21 M | Randomized (crossover) | Participants completed accentuated eccentric loading (AEL) and traditional resistance training (TRA) protocols in a random order separated by 24 hours. Salivary cortisol was assessed at baseline and 0, 15, 30, and 60 minutes post-exercise. | Cortisol  Not reported | Cortisol was determined via ELISA (Salimetrics, PA, USA) with intra- and inter-assay CV of 4.38% and 6.74%, respectively. Sensitivity not reported. | Supramaximal AEL and TRA squats | Quadriceps muscle thickness and echo intensity, self-assessment of soreness and fatigue | Cortisol: ↓ ~40% (graph estimate) | Echo intensity but no muscle thickness increased post-exercise after both protocols while muscle soreness was increased after AEL exercise only. Cortisol was significantly decreased 1-hour post-exercise compared to baseline. Reductions in cortisol were attributed to low-intensity exercise protocol. |
| Michael et al.  (2012)  Country: US  “Fatigue Biomarker Index: An Objective Salivary Measure of Fatigue Level” | Profile: Recreationally trained cyclists  n= 9  Age (y): 28.1 ± 11.0  Sex: 9 M | Randomized (crossover) | Participants completed two 10-hour exercise programs in random order separated by 7 days. During each protocol participants received either carbohydrate drinks or placebo. | Ratio of peptide pair with concentrations that changed in opposite directions with fatigue level, described as ‘fatigue biomarker index’ (FBI).  Passive drool | Proteins and peptides were quantified using a bicinchoninic acid (BCA) assay (Pierce, IL, USA). Samples were labeled with isotopic variants of acetic anhydride for mass-specific tagging, and liquid chromatography-mass spectrometry (LC-MS) was used to analyze and quantify the peptides. Specific peptide intensities were calculated to create a fatigue biomarker index (FBI) for monitoring fatigue levels. | Exercise at 70% maximum ventilatory threshold for 10-hour session | Self-reported rating of perceived exertion (RPE). | FBI: ↓ x1000 CHO, ↓ x1000 Placebo (log10: 1.5 to -1.5, log10: 1 to -2) | Significant increases in RPE were experienced after both exercise protocols. A ratio of ion intensities for two novel peptides, derived from acidic and basic proline-rich proteins was identified as an objective marker for fatigue evaluation. |
| Rutherfurd-Markwick et al.  (2017)  Country: NZ  “Salivary Diagnostic Markers in Males and Females During Rest and Exercise” | Profile: Recreationally active  n= 20  Age (y): 27.4 ± 5.9  Sex: 8 M: 12 F | Randomized (crossover) | Participants performed both 60 minutes cycling at 70% max power and 60 minutes rest protocol (sitting with no activity). The trials were separated by 3-7 days. Saliva was collected at 15 and 45 minutes into the activity. | Flow rate, osmolality, sodium (Na+), potassium (K+), chloride (Cl−), secretory immunoglobulin A (sIgA), alpha amylase (sAA) activity, and cortisol.  Unstimulated | Flow rate was calculated on the assumption that salivary density was 1 g·mL^-1^. Osmolality was measured using a freezing point depression osmometer (Osmomat 030, Berlin, Germany). Electrolyte levels were measures using an Easy-Ltye analyser (Medica Corporation, MA, USA). sIgA was determined by ELISA using Tina Quant kits (Roche Diagnostics, Germany). sAA activity was measures using Infinty Amylase Liquid stable reagent (Thermoscientific, Worthing, UK). Cortisol was determined by radioimmunoassay (IBL International GMBH, Hamburg, Germany). Sensitivity and CV not listed. | Exercise at 70% peak power over 60 minutes of cycling | RPE and HR | flow rate: ↑ 14 %, ↔ (males, females)  Osmolality: ↔, ↑ 17 % (males, females)  Na+: ↔, ↔ (males, females)  K+: ↔, ↑ 15 % (males, females)  Cl−: ↔, ↓ 15 % (males, females)  Secretory IgA: ↔, ↑ 25 % (males, females)  α-amylase activity: ↔, ↑ 66 % (males, females)  Cortisol:↔, ↔ (males, females)  *Graph estimate | HR was higher during exercise for both sexes with females having a higher average HR during exercise but not at rest with a similar pattern observed with RPE. No change was observed for Na+, while a trend towards increased K+ and decreased Cl- was observed after exercise for females only. No change was found in sIgA in response to exercise, while AA increased post-exercise for females but not for males. A trend for increased cortisol post exercise was noted for females but not for males. |
| Slivka et al.  (2010)  Country: US  “Effects of 21 Days of Intensified Training on Markers of Overtraining” | Profile: triathlon and cyclists  n= 8  age (y): 24 ± 1  Sex: 8 M | Quasi-experimental | Participants completed a 21- day intensified cycling training protocol. Saliva samples, 1-hour trial time performance, HR response, and mood were collected throughout the program. V02 max was measured before and after the 21-day program. | Testosterone, cortisol, T:C, sIgA  Passive drool | Testosterone, cortisol and sIgA were measured in duplicate using a competitive immunoassay (Salimetrics, PA, USA). Absolute secretory sIgA were corrected for salivary flow rate. CV for all assays were <10%. Sensitivity not listed. | 21-day bicycle tour (approximately 169 ± 4 km per day). | Average power (W) during 1-hour performance cycling trials, V02 max, HR response, and profile of mood states (POMS) | Testosterone: ↓ 0% - 33% (pre/post across different days)  Cortisol: ↓ 33% - 60% (pre/post across different days)  Secretory IgA: ↓ 14% (morning day 21 to day 1)  T:C: ↑ 0- 400% (pre/post across different days) | No difference in average time trial power (W) or HR recovery was found across the program and no difference in max aerobic capacity (V02 max) was found pre- to post. Vigor, as measured by POMS, decreased from day 1 to 4 and remained lower throughout the program. No change in sIgA was found in absolute or relative terms from day, however, cortisol and testosterone both decreased from pre- to post daily exercise, while T:C increased from pre-to post daily exercise with no difference in the change of any of the three markers pre- to post exercise found across the program. |
| Sparkes et al.  (2020)  Country: UK  “The Effect of Training Order on Neuromuscular, Endocrine and Mood Response to Small-Sided Games and Resistance Training Sessions Over a 24- Hour Period” | Profile: semi-professional soccer players  n= 14  Age (y): 22.1 ± 3.1  Sex: 14 M | Randomized (crossover) | Participants performed either small-sided games (SSG) followed by resistance training (RES) 2 hours later or (SSG + RES) or the opposite (RES + SSG). Two days later all participants had their order of exercises reversed. Countermovement jump (CMJ), mood, and saliva were collected pre, during, and after both protocols. | Testosterone, cortisol, T:C  Passive drool | Testosterone and cortisol were measured in duplicate via commercial immunoassay kits (Salimetrics, PA, USA). Minimum detection limit for testosterone was 6.1 pg·mL with and inter-assay CV of 5.8%. Cortisol had a detection limit of 0.12 ng·mL with an inter-assay CV of 5.5%. | Combination of small-sided games and resistance training | CMJ and RPE | Testosterone: ↓ 20%, ↓ 13% (SSG+RES, RES+SSG)  Cortisol: ↓ 45%, ↓ 45% (SSG+RES, RES+SSG)  T:C: ↑ 75%, ↑ 60% (SSG+RES, RES+SSG)  *Graph estimate | CMJ significantly decreased from baseline during RES + SSG but not during SSG + RES while mood score improved during SSG + RES but not vice versa. Testosterone and cortisol were significantly decreased while T:C was increased two hours into both protocols with no differences in changes between order of exercises. |
| Springham et al.  (2021)  Country: AU  “Salivary Immunoendocrine and Self-report Monitoring Profiles across an Elite-Level Professional Football Season” | Profile: Professional football players  n= 18  Age (y): 24 ± 3.8  Sex: 18 M | Cohort | Saliva samples were collected following recovery days across a complete 6-week and 8 5-week in-season mesocycles. Athlete self-reported measures (ASRM) of fatigue were also recorded. | Cortisol, testosterone, T:C, AA sIgA  Not reported | sIgA and cortisol were determined using lateral flow immunochromatographic (LFI; SOMA Bioscience, UK) test strips. CV wsa reported as 9.4% and 6.8% for sIgA and cortisol, respectively. sAA and testosterone were meaures by ELISA kits (EIA; SOMA BioScience, UK). Intra- and inter-assay CV for sAA and testosterone was reported as 4.71% and 11.4%; and 7.94% and 9.4%, respectively. | Competitive football season | RPE scale, self-reported measures of fatigue (ASRM), sleep quality, muscle soreness, stress level, and mood (ASRM) | Cortisol: ↓ 42 %  Testosterone: ↑ 5%  Alpha-amylase: ↔  IgA: ↑ 10% (↑ 28%, and ↑ 48% in highest workload mesocycles)  T:C: ↑ 50%  * average of 9 mesocycles compared to baseline. | Workload, as measured by CR-10 scale, was elevated in 5 out of 8 mesocycles compared to baseline. Perceived measures of fatigue were lower than preseason after mesocycle 4 and stayed depressed for the remainder of the season. Significant quadratic relationships were found between sIgA and perceived fatigue, testosterone and perceived fatigue, sleep quality, muscle soreness and ASRM total. Linear relationships were found between cortisol and perceived fatigue and sleep quality, and T:C and perceived fatigue and sleep quality. |
| Viana-Gomes et al.  (2018)  Country: Brazil  “Oxidative Stress, Muscle and Liver Cell Damage in Professional Soccer Players During a 2-Game Week Schedule” | Profile: professional soccer players  n= 8  Age (y): 27.2 ± 5.5  Sex: 8 M | Cohort | Blood and saliva were collected from professional soccer players at the start of a championship tournament. Samples were collected at baseline, post-game 1, 48hours post-game 1, post-game 2, 24 hours post-game 2 and 48 hours post-game 2. | Oxidative stress and antioxidant capacity markers were measured including total antioxidant capacity (TAC), uric acid (UA), thiobarbituric acid-reactive substances (TBARS).  Unstimulated | UA was measured in duplicate using a commercial kit (Bioclin). TAC was measured using the 2,2-diphenyl-1-picrylhydrazyl (DPPH) assay. Lipid peroxidation was quantified by measuring TBARS via spectrophotometry. | Two competitive soccer games during a low intensity training week | Plasma biomarkers of exercise intensity and muscle damage including blood lactate, creatine kinase (CK), aspartate transaminase (AST), alanine transaminase (ALT), γ-glutamyl transferase (GGT). | TAC: ↔  UA: ↓ 40% (48 hours post game 2)  TBARS: ↑ 47.6%, ↑ 62% (post game 1 and 2) | Blood lactate was significantly increased from baseline after games 1 and 2. Additionally, plasma CK increased compared to baseline at all time points. AST and ALT were only significantly elevated 48-hours after game 2, while GGT was greater post game 2 and 24 and 48-hours post game 2.  Out of the salivary biomarkers only TBARS was significantly elevated across time points, increasing immediately after each game indicating an increase in the level of lipid peroxidation post-game. |
| McKetney et al.  (2022)  Country: US  “Proteomic and Metabolomic Profiling of Acute and Chronic Stress Events Associated with Military Exercises” | Profile: US Army infantry members  n= 30  Age (y): 24.2  Sex: 26 M: 4 F | Cohort | Saliva samples were collected over 10 days split into 3 sections, ‘Battle-ready’ (baseline), ‘Mission’ (military training), and ‘Recovery’ (post-test). Multi-omic analysis was performed to develop predictive models of acute and chronic stress in the warfighter. | Identified proteins and metabolites following multi-omic analysis.  Passive drool | Proteins in saliva samples were quantified using a bottom-up proteomics approach. After saliva was collected and stored, proteins were denatured with guanidine hydrochloride, reduced, alkylated, and digested with endoprotease Lys-C and trypsin. The digested peptides were desalted, separated using liquid chromatography, and analyzed via high-resolution mass spectrometry. Protein abundances were quantified using label-free quantification (LFQ) methods, and the data were normalized and analyzed with bioinformatics tools. | Acute stressor: 2 instances of live firefights  Chronic stressor: 72-hour combat simulating training mission | None | Protein clusters were identified based on significant change in abundance. Fold changes were provided in aggregated graphs with p values/folds.  For the 9 discriminatory proteins/metabolites, log2 of label free quantification intensities graph is available in supplementary materials | More than 300 proteins and 100 compound features (metabolites) were related to mission stress. Combination models of the top 5 most discriminant proteins and top 5 most discriminant metabolites of mission initiation yielded predictive values of 83% and 96%, respectively. |
| Akazawa et al.  (2019)  Country: Japan  “Effect of Sleep Efficiency on Salivary Metabolite Profile and Cognitive Function During Exercise in Volleyball Athletes” | Profile: Competitive college level volleyball players  n= 12  Age (y): 20± 0.3  Sex: 12 F | Quasi-experimental | Participants had saliva samples collected before the performance of a cognitive task (Stroop test) at rest and during exercise (8 minutes bouts of light and heavy intensity cycling) after measurement of sleep quality during the prior week. Outcome measures were compared across ‘lesser’ and ‘better’ sleep quality groups depending on sleep data scores. | A range of identified metabolites in which differences were detected between lesser and better sleep quality groups  Stimulated | Metabolites were quantified using capillary electrophoresis and time-of-flight mass spectrometry (CE-TOFMS). Saliva samples were collected, centrifuged to remove proteins and macromolecules, and passed through a 5 kDa cutoff filter. The filtrate was analyzed using CE-TOFMS to identify and quantify metabolites based on their mass-to-charge ratios, migration times, and peak areas. | Sleep efficiency (lesser vs greater), exercise (light vs intense) | Cognitive ability (Stroop test scores). | Lysine: ↑ 400%  Proline: ↑ 33%  Tyrosine: ↑ 50%  Ornithine: ↑ 400%  Citrulline: ↑ 66%  Arginine: ↑ 60%  2-oxoglutaric acid: ↑ 33%  Myo-inositol 3-phosphate: ↓ 70%  Caffeine: ↑ 400%  Urea: ↓ 50%  *Graph estimate. Change in better sleep relevant to the poor sleep group | Sleep efficiency was greater, and sleep latency, and arousal time was significantly shorter in the better quality sleep group compared to lesser quality group.  Several metabolites were significantly different between sleep status groups including greater levels of lysine, proline, tyrosine, ornithine, citrulline, 2-oxoglutatic acid, and caffeine in the better sleep group. Lysine, tyrosine, citrulline, and arginine were also significantly correlated with sleep efficiency and citrulline levels were correlated with Stroop response time during heavy exercise. Results suggest that sleep quality may be associated with glucose and ammonia metabolism. |
| Crewther et al.  (2020)  Country: UK  “Performance Indicators During International Rugby Union matches are Influenced by a Combination of Physiological and Contextual Variables” | Profile: Professional rugby players  n= 29  Age (y): 28.8 ± 2.8 (13 ‘back’ position)  27.8 ± 3.4 (16 ‘forward’ position)  Sex: 29 M | Cohort | Participants were followed across 8 matches to determine the relationship of game performance with physiological and contextual variables. Saliva samples were collected during the morning of each game day. | Cortisol, testosterone  Passive drool | Testosterone and cortisol were assayed in duplicate using ELISA kits (Salimetrics, PA, USA). The kits had a sensitivity limit of 1pg/ml for testosterone and 0.07 ng/nl for cortisol. Inter-assay CV was <10% for both testosterone and cortisol. | Training and gamed induced fatigue | Coach and player ratings of performance and quantitative game statistics including offloads, turnovers, runs with ball in hand, tackles, passes, and defenders beaten | Quantities not reported | Morning cortisol but not testosterone level was positively related to coach and player ratings of performance. Cortisol was also associated with game metrics (less turnovers and passes). However, sleep and mood state were the most consistent predictors of game performance. |
| Fogt et al.  (2009)  Country: US  “Hydration Status of Air Force Military Basic Trainees After Implementation of the Back-Mounted Hydration System” | Profile: Air Force trainees  n= 78 (Back mounted (BM): 40; Regular canteen (SI): 38)  Age (y): BM: 19.6 ±2.7; SI: 19.3 ±1.9  Sex: 78 M | Randomized (parallel group) | Participants were randomly assigned into back-mounted or standard issue hydration systems. Saliva samples were collected daily over 5 weeks in the morning and before dinner. | Saliva osmolality, total protein concentration  Unstimulated | Osmolality was determined using a freezing point depression osmometer (Model 3 MO, Advanced Instruments, MA, USA). Total protein was measured on a serial aliquot of saliva supernatant using a colormetric assay (Sigma-Aldrich Corp., MO, USA). | 5-week military training | Total body water (TBW) assessed with bioelectrical impedance device | Osmolality: ↑ 25% (over 5 weeks)  Total protein: ↓ 20% (over 5 weeks) | Saliva osmolality and total protein decreased from morning to afternoon for both hydration systems, but fluctuation was not statistically significant. |
| Heydari et al.  (2022)  Country: Iran  “Effect of Competition on Salivary α-amylase in Taekwondo Athletes” | Profile: Taekwondo athletes  n= 20  Age (y): 18-25  Sex: 20 M | Cohort | Saliva samples were collected 1-week before, 15 minutes before, immediately after, 30 minutes after, and 24-hours after a Taekwondo competition. | Alpha-amylase  Stimulated | sAA activity was measured with kinetic chlorometric method using a kit with reported accuracy of 2.5 units per milliliter. | Competition induced fatigue and stress | Self-reported stress level (via questionnaire) and competition outcome | alpha amylase: ↑ 55%, ↑ 127%, ↑ 42%, ↓ 82% (Relative to baseline 1 week before : 15 min. before competition, immediately after, 30 min. after, 1 day after) | Alpha-amylase was significantly different across time points, increasing from one-week before the competition to a peak immediately after the competition, after which it returned to baseline levels 24-hours post. Additionally, a positive correlation was observed between alpha-amylase and self-reported stress. |
| Tait et al.  (2022)  Country: Australia  “Impact of Military Training Stress on Hormone Response and Recovery” | Profile: Army Combat Engineer Initial Employment Training (IET) course soldiers  n= 30  Age (y): 22.7 ± 3.8  Sex: 27 M: 3 F | Cohort | Participants were tracked over a 16-day period which included an 8-day military training exercise during which saliva samples were collected at waking, 30 min post- waking, | Cortisol, testosterone  Unstimulated | Testosterone and cortisol were analyzed in duplicate using ELISA kits (IBL International, Hamburg, Germany). Intra- and inter-assay CV was <10% and <20%, respectively for both assays. Sensitivity was not reported. | Training induces fatigue and reduced sleep | Self-reported stress and recovery | Cortisol: ↓ 18%, ↑ 76% (AM, PM)  Testosterone: ↓ 44%, ↓ 23% (AM, PM)  Melatonin ↔ | Cortisol levels were lower in the morning after total sleep deprivation. Testosterone levels reduced but recovered within 4 days. Testosterone/cortisol level at bedtime did not recover to pre training by the end of the protocol (3 day recovery period) |
| Taylor et al.  (2007)  Country: US  “Stressful Military Training: Endocrine Reactivity, Performance, and Psychological Impact” | Profile: Active-duty navy trainees  n= 19  Age (y): 21.5 ± 1.7  Sex: 19 M | Cohort | Saliva samples were taken from Navy trainees at baseline (unstressed) and during survival and captivity training. | Cortisol, dehydroepiandrosterone sulfate (DHEAS)  Stimulated | Cortisol was measured using a Coat-A-Count radioummunoassay kit (Diagnostic Products Corporation, CA, USA) with a sensitivity of 0.7-55.2 nmol·L^-1^ and intra- and inter-assay CV of 3-7% and 5%, respectively. DHEAS was measured using a radioimmunoassay kit (Diagnostic Systems Laboratories, Inc., TX, USA) with a sensitivity of 0.1-30 ng·ml^-1^ and intra- and inter-assay CV of 5-7% and 6%, respectively. | Survival, Evasion, Resistance, and Escape Training | Survival target skills | Cortisol: ↓ 69% / 37% (waking /30 minutes post waking)  Testosterone: ↓ 28%-38% (at different days relative to baseline)  T/C ratio: ↓ 42%-73% (relative to different days) | Cortisol and DHEAS were elevated during captivity training compared to baseline. Performance during high-intensity captivity training was inversely related to DHEAS-cortisol ratio. Conversely, performance during low-intensity captivity training was positively related to DHEAS-cortisol ratio. |
| Julià-Sánchez et al.  (2013)  Country: Spain/Austria  “Salivary pH Increases After Jump Exercises in Hypoxia” | Profile: Active young adults  n= 8  Age (y): 33.62 ± 4.07  Sex: Not reported | Randomized (crossover) | Saliva was collected from 8 participants before and after performing intense exercise at 3 different simulated altitudes: 550m, 2500m, and 4000m. Salivary pH was assessed, as well as post workout blood lactate and oxygen saturation | Salivary pH  Passive drool | Salivary pH was measured using a digital pH-meter (Lutron PH-220, Tapei, Taiwan). | Simulated Altitude (hypoxia) under intense exercise (6 sets of 15 s jumping) | Blood lactate, HR, and arterial oxygen saturation | Salivary pH: ↑ ~0.3* | Blood lactate and heart rate were significantly increased post-exercise, however, no difference in that change was found across groups. In comparison, oxygen saturation showed greater change in the hypoxia groups compared to normoxia. Salivary pH significantly increased after exercising at 2500m and 4000m but not 550m, but not during normoxia condition. A significant negative correlation was found between salivary pH and arterial oxygen for hypoxia conditions but not during normoxia. |
| Pontremolesi et al.  (2012)  Country: Italy  “Acute Hypobaric-Hypoxia Challenge and Salivary Cortisol and DHEA-S in Healthy Male Subjects” | Profile: Military aviators (experimental),  flight physiology instructors (control)  n= 20 (8 control)  Age (y): 37 ± 7 (exp.)  39 ± 5 (control)  Sex: 20 M | Randomized (parallel group) | Participants experienced altitude (7620 m) induced hypoxia in a simulation chamber for 5 minutes. A control group received the same treatment but with oxygen masks. Salivary samples were taken for 3 days (before, trial day, post-trial) every 2 hours from 08:30 to 20:30. | Salivary cortisol and dehydroepiandrosterone sulfate (DHEAS)  Not reported | Cortisol and DHEAS were assayed with commercial immunoenzymatic kits (Diametra, Milan, Italy). Cortisol had a minimum detectable concentration of cortisol of 0.5 ng·ml^-1^ and intra- and inter-assay CV of <10% and <7%, respectively. DHEAS had a minimal detectable concentration of 25 pg·ml^-1^ , also with an intra- and inter-assay CV of <10% and <7%, respectively. | Hypoxia (altitude induced) | None | Cortisol: ↑ 100%, ↑ 700% (AM, PM)  DHEA-s: ↑ 300%, ↑ 200% (AM, PM)  DHEA-s/Cortisol: ↑130%, ↓69% (AM, PM)  *Graph estimate | Cortisol and DHEA-S levels were significantly elevated during altitude-induced hypoxia. No difference in hormone concentrations were found the day after in either group compared to baseline. The DHEA-S to cortisol ratio did not change during induced hypoxia indicating stress resilience. |
| Tsunekawa et al.  (2022)  Country: Japan  “Differences in Stress Response Between Two Altitudes Assessed by Salivary Cortisol Levels Within Circadian Rhythms in Long‑Distance Runners” | Profile: Elite long-distance runners  n= 12  Age (y): 23.5 (19.5–26.0)  Sex: 12 F | Quasi-experimental | Saliva was collected from 12 elite runners during a high-altitude training camp and a low-altitude training camp. | Cortisol  Stimulated | Cortisol was measured via electrochemiluminescence immunoassay (ECLIA) using the Elecsys Cortisol II on the Cobas 8000 system (Roche Diagnostics, Japan). Intra- and inter-assay CV were 4.1% and 4.6%, respectively. Sensitivity was not reported. | Exercise (running) at high and low altitude | None | Cortisol: ↑ 110 %  Dehydroepiandrosterone-sulfate (DHEA-S): ↑ 90 % | Basal salivary levels before dinner were significantly higher at the high-altitude camp compared to the low. Additionally, the rate of change in the salivary cortisol concentrations on day 2 was higher in the high altitude camp compared to low. |
| Woods et al.  (2012)  Country: UK  “The Cortisol Response to Hypobaric Hypoxia at Rest and Post-Exercise” | Profile: Expedition trekkers  n= 55 total (15 had data available both at rest and post-exercise)  Age (y): 35 ± 8 (22–51)  Sex: 26 M | Cohort | Salivary cortisol was measured at rest and at >5000m after a 6-h trek. | Cortisol  Passive drool | Cortisol was measured via high sensitivity ELISA kit (Salimetrics Europe, Suffolk, UK). Sensitivity was reported as 0.57 nmol/l and Inter-assay CV was 4.5%-8.2%.s | Exercise at high-altitude | None | Cortisol: ↑ 80% and 70% morning exercise change (high and low altitude on day 2)  Cortisol: ↑ 270% and 100% afternoon exercise (high and low altitude on day 2)  Cortisol evening dinner level: ↑ 220 % day 2 high altitude relative to low altitude | Both morning (at rest) and afternoon (post-exercise) cortisol levels significantly rose from baseline to peak ascent. However, a suppression effect on cortisol was found with altitude as levels initially dropped with ascent until an altitude of >4270m where it significantly increased. This was attributed to the fact that resting baseline cortisol levels were recorded at 1300m (base camp). |
| Meléndez-Gallardo et al.  (2022)  Country: Uruguay  “Salivary Molecules as Indicators of Hydration Status” | Profile: Young adults  n= 10  Age (y): 25.5 ± 3.7  Sex: 10 M | Quasi-experimental | Subjects ran for 120 minutes on a treadmill at 60% VO2 max. Exercise was broken into 8 intervals of 15 minutes of activity and 8 minutes rest. Saliva samples were collected during each rest period. | Alpha-amylase, K+, Cl^–^, cortisol, total protein, and osmolality  Unstimulated | sAA was quantified using a colorimetric enzymatic method with the substrate 4.6 ethylidene-p-nitrophenyl-α, D-maltoheptaosoid, analyzed on a Roche Integra 800 system​ (Roche Diagnostics, Mannheim, Germany). Osmolality was analyzed using a vapor pressure osmometer (Vapro, UT, USA). Potassium and protein were measured by Cobas Integra 800 (Roche Diagnostics, Mannheim, Germany). Chloride was analyzed with a chloridemeter (CM20, Kreienbaum, Germany). Cortisol concentrations were measured by liquid chromatography-tandem mass spectrometry. | Exercise induced dehydration | Body mass loss | Cortisol: ↑ 350% at rest after ascent.  Cortisol: ↓ 43% after exercise from 3400 to 4270 m, ↑ 125% after exercise and ascent to 5150 m.  *Graph estimate | Total body water loss reached levels of moderate dehydration by the end of the exercise protocol (2.5-5.5% of BM). Cortisol and alpha-amylase were significantly increased only after losses of total body water of ~2% or greater. Osmolality and K+ concentrations were significantly different only after participants reached moderate hydration also. Cl^–^ concentrations showed significant differences only after mild dehydration. |
| Muñoz et al.  (2014)  Country: US  “Optimal Hydration Biomarkers: Consideration of Daily Activities” | Profile: Recreationally active young adults  n= 23  Age (y): 22 ± 3  Sex: 23 M | Quasi-experimental | Participants completed 2 experimental protocols in a hot environment (36°C, 50% humidity): passive dehydration (PAS), and active dehydration (ACT). With each % loss in body mass, saliva samples were taken. | Salivary osmolality (V_osm_)  Not reported | **Osmolality** was measured using a vapor pressure osmometer, however, specific device details were not provided. | Passive dehydration (sitting for 5 hours) and active dehydration (cycling at 68% age-predicted max heart rate for 5 hours) in hot environment | Body mass loss,  blood serum osmolality, salivary osmolality, urine osmolality, urine, and urine specific gravity | Alpha-amylase: ↑ 100%  K+: ↑ 85%  Cl-: ↑ 700%  Cortisol: ↑ 500%  Total protein: ↑ 500%  Saliva osmolality: ↑ 133%  *Graph estimate, 0 to 3% body mass loss | Salivary and serum markers were more accurate to predict dehydration during ACT while urine markers were more accurate during PAS. Salivary osmolality most accurately predicted a dehydrated state due to exercise (≤2% body mass loss) with 86% sensitivity and 91% specificity. |
| Muñoz et al.  (2013)  Country: US  “Assessment of Hydration Biomarkers Including Salivary Osmolality During Passive and Active Dehydration” | Profile: Recreationally active young adults  n= 23  Age (y): 22 ± 3 years  Sex: 23 M | Quasi-experimental | Participants completed 2 experimental protocols in a hot environment (36°C, 50% humidity): passive dehydration (PAS), and active dehydration (ACT). With each % loss in body mass, saliva samples were taken. | Salivary osmolality  Passive drool | Osmolality was measured via freezing point depression osmometer (Advanced Instruments, MA, USA) | Passive dehydration (sitting for 5 hours) and active dehydration (cycling at self-selected moderate pace for 5 hours) in hot environment | Body mass loss,  blood serum osmolality (S_osm_), urine osmolality (U_osm_), volume (U_vol_), and specific gravity (U_sg_) | Osmolality: ↑ 9%, ↑ 38% (passive, active dehydration at -1% body mass loss)  ↑ 100% (active, at -3% body mass loss)  *Graph estimate | Total average body mass loss during PAS and ACT was -1.4% and -4.1%, respectively. Significant changes in S_osm_, V_osm_, U_osm_ (increase), and U_vol_ (decrease) were found with progressive loss in body mass due to dehydration, however, change was greater during ACT than PAS. Salivary osmolality  showed greater accuracy in detecting dehydration (≤2% body mass loss), closely followed by blood serum osmolality (S_osm_). |
| Walsh et al.  (2004)  Country: UK  “Saliva Parameters as Potential Indices of Hydration Status During Acute Dehydration” | Profile: Young adults  n= 15  Age (y): 23 ± 3  Sex: 15 M | Randomized (crossover) | Participants performed 2 exercise dehydration trials until body mass loss was 3%. The first trial did not allow fluid intake (NFI) while the second did allow fluid intake (FI). | Saliva flow rate, osmolality, and total protein concentration  Unstimulated | Salivary flow rate was determined by dividing volume of saliva by collection time. Salivary osmolality was measured using a freezing point depression osmometer (Model 3MO, Advanced Instruments, MA, USA). Total protein concentration was analyzed using a spectrophometer with a commercially available kit (Kit No. 610, Sigma, Poole, UK). | Acute dehydration (3% loss in body mass) | Body mass change, salivary, urine and serum markers | Vosm: ↑ 9% passive dehydration, ↑ 41% active dehydration (1% body mass change for both) | Saliva osmolality rate and total protein increased while saliva flow rate decreased in the NFI group. No change was experienced in the FI group. In both groups, catecholamines increased, suggesting that the change in saliva variables was due to dehydration and not sympathetic activity. |
| Walsh et al.  (2004)  Country: UK  “Saliva Flow Rate, Total Protein Concentration and Osmolality as Potential Markers of Whole Body Hydration Status During Progressive Acute Dehydration in Humans” | Profile: Young adults  n= 12  Age (y): 21 ± 1  Sex: 12 M | Quasi-experimental | Participants cycled at 60% V02 max in an environmental chamber (30°C, 70% humidity) until progressive body mass loss of 1%, 2%, and 3%. After exercise, participants drank a carbohydrate electrolyte solution equal to 150% body mass loss within 1 hour. Saliva samples were collected at pre-exercise, 1%, 2%, and 3% body mass loss, and then at 75, 135, and 195 min post-exercise. | Saliva flow rate, osmolality, and total protein concentration  Unstimulated | Salivary flow rate was determined by dividing volume of saliva by collection time. Salivary osmolality was measured using a freezing point depression osmometer (Model 3MO, Advanced Instruments, MA, USA). Total protein concentration was analyzed using a spectrophometer with a commercially available kit (Kit No. 610, Sigma, Poole, UK). | Acute dehydration (up to 3% loss in body mass) | Body mass loss | Flow rate: ↓ 62%  Osmolality: ↑ 110%  Total protein: ↑ 203% | Saliva flow rate decreased, and total protein concentration and osmolality increased during dehydration. Saliva flow rate was significantly reduced from baseline by 2% loss in body mass while total protein concentration and osmolality were significantly higher than baseline by 1% loss in body mass. Additionally, total protein concentration and osmolality were strongly correlated with % body mass loss during dehydration. |
| Walsh et al.  (2002)  Country: UK  “Salivary IgA Response to Prolonged Exercise in a Cold Environment in Trained Cyclists” | Profile: Competitive cyclists  n= 15  Age (y): 29 ± 2  Sex: 15 M | Randomized (cross-over) | Participants cycled at 70% VO_2_ max for 2 hours in an environmental chamber across two conditions: cold (-6.4°C) and control (19.8°C). Saliva samples were collected before, after, and 2 hours after exercise. | Salivary IgA, osmolality, and flow rate  Unstimulated | Salivary flow rate was determined by dividing volume of saliva by collection time. Salivary osmolality was measured using a freezing point depression osmometer (Model 3MO, Advanced Instruments, MA, USA). sIgA was measured using a sandwhich-type ELISA method (Sigma, Poole, UK). CV of analytical method was 8.1% and 2.0% for sIgA and osmolality, respectively. | Exercise in cold and normal (control) temperature | None | Flow rate: ↓ 60%  Osmolality: ↑ 100%  Total protein: ↑ 133%  *Graph estimate | Both saliva rate and s-IgA significantly decreased post-exercise. At post-exercise, s-IgA concentration was significantly higher in the control trial compared to cold. In comparison, saliva osmolality was significantly higher post-exercise, while it was lower in the cold trial than control. Overall, the response to exercise was the same in both cold and control trials for salivary flow rate and s-IgA. |
|  |  |  |  |  |  |  |  |  |  |

**Abbreviations:** sAA, salivary alpha amylase; ACT, active dehydration; ACTH, adrenocorticotrophic hormone; ALT, alanine transaminase; ARMS, acute readiness monitoring scale; AST, aspartate aminotransferase; AEL, accentuated eccentric loading; ASRM, athlete self-reported measures; CK, creatine kinase; CMJ, countermovement jump; DHEA, dehydroepiandrosterone; DHEAS, dehydroepiandrosterone sulfate; EEG, electroencephalogram; F, female; FBI, fatigue biomarker index; GGT, γ-glutamyl transferase; HIIT, high-intensity interval training; HGS, hand-grip strength; HR, heart rate; HRV, heart rate variability; sIgA, salivary immunoglobulin A; IL-6, interleukin 6; LDH, lactate dehydrogenase; M, male; MAV, maximal aerobic velocity; MVC, maximum voluntary contraction; mtDNAcn, mitochondrial DNA copy number; NASA-TLX, national aeronautics and space administration task load index; PASAT, paced auditory serial addition test; PFS, piper fatigue scale; POMS, profile of mood states; PVT, psychomotor vigilance test; RF, Ramadan fasting; RPE, rating of perceived exertion; ROC, receiver operating curve; sIgA, secretory immunoglobulin A; S_osm_, blood serum osmolality; SSG, small-sided games; TAC, total antioxidant capacity; TBARS, thiobarbituric acid-reactive substances; TBW, total body water; T:C, testosterone to cortisol ratio; TMT, trail making test; TRA, traditional resistance training; REMT, repeatable episodic memory test; RES, resistance training; UA, uric acid; UK, United Kingdom; US, United States; U_osm_, urine osmolality; U_sg_, urine specific gravity; U_vol_, urine volume; V_osm_, Salivary osmolality; WBV, whole body vibration; y, years.
