# Supplementary material for: Salivary biomarkers of tactical athlete readiness: A systematic review
Source: PLoS One. 2025 Apr 29;20(4):e0321223. doi: 10.1371/journal.pone.0321223 (PMC12040155; doi:10.1371/journal.pone.0321223)
Supplement: Attachment — Submitted filename: 20250221 Response Letter.pdf [file pone.0321223.s007.pdf]

GEORGE MASON UNIVERSITY | School of Kinesiology

February 21, 2025

Dr. Castillo-Navarrete and reviewers,

We are pleased to re-submit the following manuscript titled: “Salivary biomarkers of tactical athlete readiness: A systematic review”. With our resubmission we are providing a revised manuscript with changes noted in track changes, a clean verision of the revised manuscript and this response letter. Point by point responses to comments are provided below. We would like to express our appreciation to each of the reviewers and the editor for their time.

Please review your reference list to ensure that it is complete and correct. If you have cited papers that have been retracted, please include the rationale for doing so in the manuscript text, or remove these references and replace them with relevant current references. Any changes to the reference list should be mentioned in the rebuttal letter that accompanies your revised manuscript. If you need to cite a retracted article, indicate the article’s retracted status in the References list and also include a citation and full reference for the retraction notice.
Response: We have reviewed the reference list and made sure that the references are correct and no retracted articles were cited.

Additional Editor Comments:
After reviewing the changes and the reviewers’ comments, we appreciate the effort you have put into improving the paper. The study addresses a relevant topic, and the revisions have significantly strengthened the manuscript.
Response: Thank you for your positive feedback and recognition of the effort put into the revisions. We appreciate your guidance in strengthening the manuscript throughout the review process.

To further refine the work, we recommend minor revisions. These include clarifying and streamlining the discussion, incorporating additional references on biomarkers such as oxytocin, muscle enzymes, uric acid, and proteomics, and briefly addressing the role of serum biomarkers and their potential correlations with salivary studies. Providing more details on assays and proteomic methods, as well as discussing the impact of race on biomarker outcomes, would also enhance the manuscript.
Response: We appreciate the further feedback on our manuscript. To address these points we made further edits to the discussion to improve the structure and flow from paragraph to paragraph. In some cases edits were made to remove any redundant statements.

A few edits were made to address the role of serum biomarkers section of the discussion. The edits were made to highlight their comparative strengths and limitations alongside salivary biomarkers.

Minor edits regarding the assays and proteomic methods were made to results, discussion and supplementary table 3 to ensure consistent language across these components of the paper.

Lastly the additional references were incorporated to the discussion:

González Fernández, Á., de la Rubia Ortí, J. E., Franco-Martinez, L., Ceron, J. J., Mariscal, G., & Barrios, C. (2020). Changes in salivary levels of creatine kinase, lactate dehydrogenase, and aspartate aminotransferase after playing rugby sevens: the influence of gender. *International journal of environmental research and public health*, *17*(21), 8165.

Barranco, T., Tvarijonaviciute, A., Tecles, F., Carrillo, J. M., Sánchez-Resalt, C., Jimenez-Reyes, P., ... & Cugat, R. (2017). Changes in creatine kinase, lactate dehydrogenase and aspartate aminotransferase in saliva samples after an intense exercise: a pilot study. *The Journal of sports medicine and physical fitness*, *58*(6), 910-916.

González-Hernández, J. M., Franco, L., Colomer-Poveda, D., Martinez-Subiela, S., Cugat, R., Cerón, J. J., ... & Tvarijonaviciute, A. (2019). Influence of sampling conditions, salivary flow, and total protein content in uric acid measurements in saliva. *Antioxidants*, *8*(9), 389.

Bishop NC, Gleeson M. Acute and chronic effects of exercise on markers of mucosal immunity. Frontiers in Bioscience. 2009;14(2):4444–56.

González Fernández, Á., de la Rubia Ortí, J. E., Franco-Martinez, L., Ceron, J. J., Mariscal, G., & Barrios, C. (2020). Changes in salivary levels of creatine kinase, lactate dehydrogenase, and aspartate aminotransferase after playing rugby sevens: the influence of gender. *International journal of environmental research and public health*, *17*(21), 8165.

Franco-Martínez, L., González-Hernández, J. M., Horvatić, A., Guillemin, N., Cerón, J. J., Martínez-Subiela, S., ... & Reyes, P. J. (2020). Differences on salivary proteome at rest and in response to an acute exercise in men and women: a pilot study. *Journal of proteomics*, *214*, 103629.

Regards,

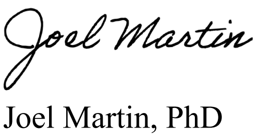

Joel Martin, PhD
George Mason University
Associate Professor, Kinesiology
